# A BioBricks Metabolic Engineering Platform for the Biosynthesis of Anthracyclinones in *Streptomyces coelicolor*

**DOI:** 10.1021/acssynbio.2c00498

**Published:** 2022-11-15

**Authors:** Rongbin Wang, Jennifer Nguyen, Jacob Hecht, Nora Schwartz, Katelyn V. Brown, Larissa V. Ponomareva, Magdalena Niemczura, Dino van Dissel, Gilles P. van Wezel, Jon S. Thorson, Mikko Metsä-Ketelä, Khaled A. Shaaban, S. Eric Nybo

**Affiliations:** † Department of Life Technologies, University of Turku, FIN-20014 Turku, Finland; ‡ Department of Pharmaceutical Sciences, College of Pharmacy, Ferris State University, Big Rapids, Michigan 49307, United States; ^§^Center for Pharmaceutical Research and Innovation, ^∥^Department of Pharmaceutical Sciences, College of Pharmacy, University of Kentucky, Lexington, Kentucky 40536, United States; ⊥ Institute of Biology, Leiden University, Sylviusweg 72, 2333 BE Leiden, The Netherlands; # Department of Biotechnology and Nanomedicine, SINTEF AS, P.O. Box 4760 Torgarden, NO-7465 Trondheim, Norway

**Keywords:** BioBricks, synthetic biology, natural product biosynthesis, anthracyclinones, *Streptomyces coelicolor*, anticancer

## Abstract

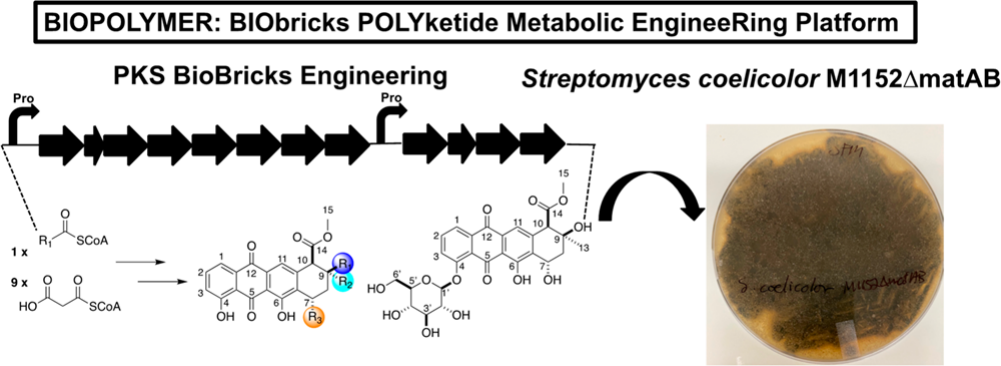

Actinomycetes produce a variety of clinically indispensable molecules, such as antineoplastic anthracyclines. However, the actinomycetes are hindered in their further development as genetically engineered hosts for the synthesis of new anthracycline analogues due to their slow growth kinetics associated with their mycelial life cycle and the lack of a comprehensive genetic toolbox for combinatorial biosynthesis. In this report, we tackled both issues via the development of the BIOPOLYMER (BIOBricks POLYketide Metabolic EngineeRing) toolbox: a comprehensive synthetic biology toolbox consisting of engineered strains, promoters, vectors, and biosynthetic genes for the synthesis of anthracyclinones. An improved derivative of the production host *Streptomyces coelicolor* M1152 was created by deleting the *matAB* gene cluster that specifies extracellular poly-β-1,6-*N*-acetylglucosamine (PNAG). This resulted in a loss of mycelial aggregation, with improved biomass accumulation and anthracyclinone production. We then leveraged BIOPOLYMER to engineer four distinct anthracyclinone pathways, identifying optimal combinations of promoters, genes, and vectors to produce aklavinone, 9-*epi*-aklavinone, auramycinone, and nogalamycinone at titers between 15–20 mg/L. Optimization of nogalamycinone production strains resulted in titers of 103 mg/L. We structurally characterized six anthracyclinone products from fermentations, including new compounds 9,10-*seco*-7-deoxy-nogalamycinone and 4-*O*-β-d-glucosyl-nogalamycinone. Lastly, we tested the antiproliferative activity of the anthracyclinones in a mammalian cancer cell viability assay, in which nogalamycinone, auramycinone, and aklavinone exhibited moderate cytotoxicity against several cancer cell lines. We envision that BIOPOLYMER will serve as a foundational platform technology for the synthesis of designer anthracycline analogues.

## Introduction

**Figure 1:**
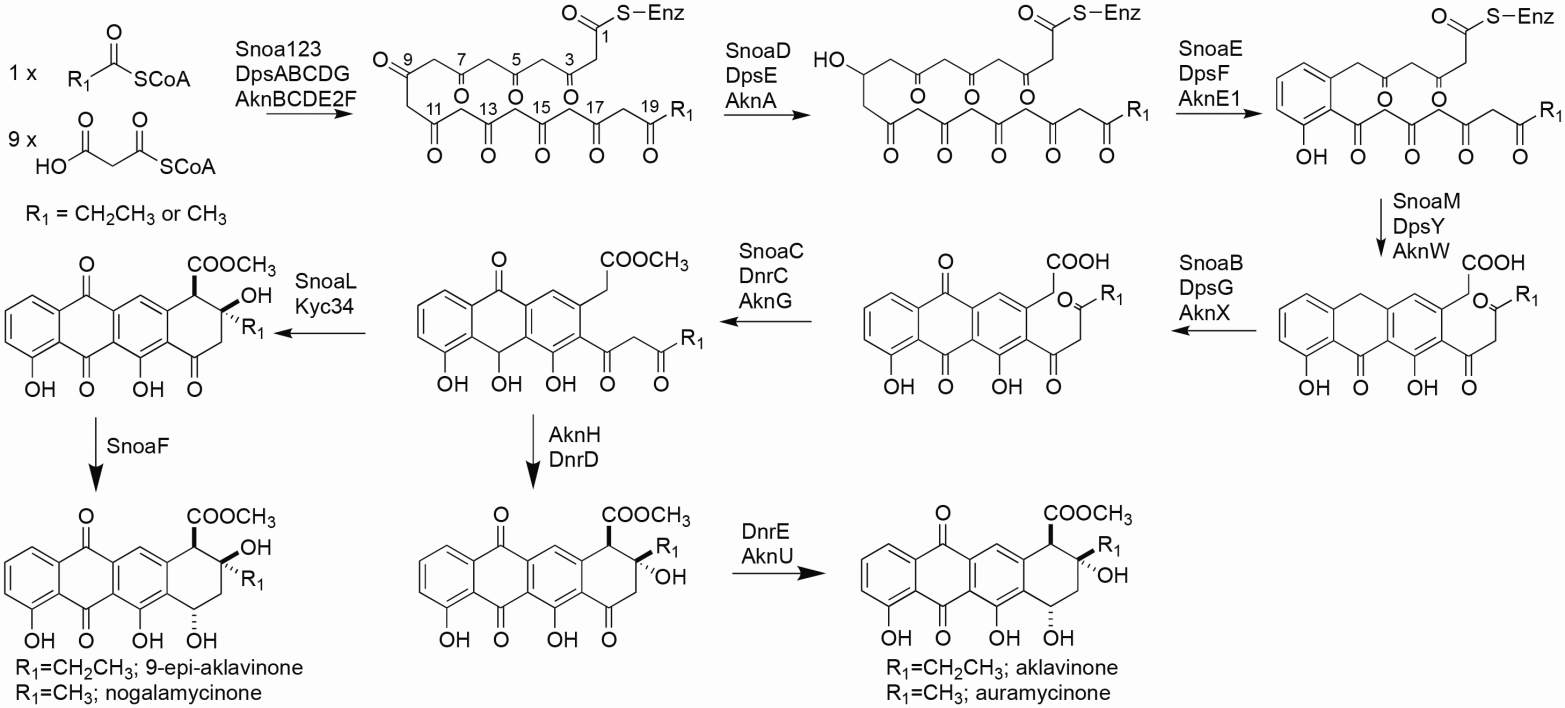
Biosynthesis of anthracyclinones. One acetyl-CoA or propionyl-CoA starter unit is condensed with nine malonyl-CoA extender units via iterative Claisen condensation reactions via the minimal PKS to form an enzyme-tethered decaketide intermediate. The polyketide ketoreductase reduces the 9-ketone to a hydroxyl group followed by C7–C12 first-ring aromatization, C5–C14 s-ring cyclization, and C3–C16 third-ring cyclization by the second-third ring cyclase. C-12 oxidation is catalyzed by an anthraquinol monooxygenase, followed by *O*-methylation to afford a tricyclic anthraquinone methyl ester. The key tricyclic intermediate undergoes fourth-ring cyclization via two distinct routes: SnoaL/Kyc34 cyclizes the fourth ring to afford a 9(*S*)-anthracyclinone and AknH/DnrD cyclizes the fourth ring to afford a 9(*R*)-anthracyclinone. The final step is C-7 ketoreduction by ketoreductases to afford C-21 anthracyclinones 9-*epi*-aklavinone or aklavinone and C-20 anthracyclinones nogalamycinone and auramycinone.

Anthracyclines are clinically important natural products for the treatment of human cancers.^1^ Anthracyclines influence DNA and chromatin structure via two distinct mechanisms: histone eviction from open chromosomal regions and the inhibition of topoisomerase II, resulting in the formation of double-stranded DNA breaks.^2^ Recently, several anthracycline biosynthetic pathways have been characterized, which provide genetic tools for the metabolic engineering of new anthracycline derivatives. The nogalamycin, aclacinomycin, and daunorubicin polyketide pathways are canonical examples of anthracycline biosynthesis and have been studied in detail over the past few decades.^3−7^ For example, anthracyclinones are C_20_ (e.g., nogalamycin) or C_21_ (e.g., daunorubicin or aclacinomycin) aromatic polyketides that are synthesized by minimal polyketide synthase (minPKS) composed of a ketoacyl synthase (KSa, AknB/DpsA/Snoa1), chain length factor (KSb, AknC/DpsB/Snoa2), and an acyl carrier protein (ACP, AknD/DpsG/Snoa3) to form an enzyme-linked poly-β-ketothioester intermediate (Figure 1). The AknBCDE2F or DpsABCDG minPKS complex condenses one propionyl-CoA starter unit to nine malonyl-CoA extender units to form a C-21 intermediate,^8^ whereas the Snoa123 minPKS condenses one acetyl-CoA and nine malonyl-CoA extender units to form a C-20 polyketide. The poly-β-ketothioester is reduced at 9-position by a PKS-associated ketoreductase (KR, AknA/DpsE/SnoaD), and then is aromatized via C7–C12 cyclization (ARO, AknE/DpsF/SnoaE), C5–C14 and C3–C16 cyclization via second-third ring cyclase (CYC, AknW/DpsY/SnoaM),^9^ and C-12 oxidation via an anthraquinol oxygenase (OXY, AknX/DpsG/SnoaB)^10^ to afford a tricyclic anthraquinone intermediate (Figure 1). The tricyclic anthracyclinone is *O*-methylated to form nogalonic acid methyl ester (NAME, SnoaC)^11^ or aklanonic acid methyl ester (AAME, AknG/DnrC),^12^ respectively, followed by fourth-ring cyclization to a 9(*S*)-configured anthracyclinone (Kyc34/SnoaL) and subsequent C7-ketoreduction (SnoaF) to afford nogalamycinone or 9-*epi-*aklavinone^13,14^ (Figure 1). Alternatively, the tricyclic intermediate can undergo fourth-ring cyclization to a 9(*R*)-configured anthracyclinone (AknH/DnrD) and 7-ketoreduction (AknU/DnrE) to yield aklavinone or auramycinone (Figure 1).^15^ Inspired by the biosynthetic logic of the anthracycline PKS system, the motivation for the present work was to develop an expanded metabolic engineering toolset to improve access to these important natural products and to generate new anthracycline analogues via microbial synthesis.

Synthetic biology is a discipline that has been defined as the “engineering-driven building of increasingly complex biological entities for novel applications”.^16^ Indeed, the field of synthetic biology has resulted in new genetic systems and organisms useful for filling in critical gaps in the pharmaceutical, biofuels, and cosmetics industries.^17^ Synthetic biology boasts the development of a variety of genetic tools, including chassis hosts, promoters, terminators, genes, and vectors useful for reprogramming model organisms. Synthetic biology is also a promising discipline for increasing access to indispensable natural products, such as polyketides.^18^ Synthetic biology has also contributed new tools for the refinement of idiosyncratic model organisms, such as *Streptomyces* spp., that are difficult to transform or feature morphological limitations, such as the formation of mycelial clumps during submerged liquid fermentation.^19,20^*Streptomyces* produce valuable bioactive molecules, such as anthracyclines and tetracyclines. *Streptomyces* spp. exhibit a highly complex life cycle, including sporulation, branching, fragmentation, and adhesion that is regulated and correlated with specialized metabolism, which limits their use as industrial hosts. Recently, the *matAB* gene cluster was discovered to encode novel glycosyltransferases responsible for the synthesis of extracellular poly-β-1,6-*N*-acetylglucosamine (PNAG) that leads to cellular clumping.^21^ Van Dissel et al. inactivated the *matAB* gene cluster in *Streptomyces coelicolor* M145 and characterized strains that lost the mycelial aggregation phenotype and exhibited improved biomass accumulation. Here, we developed the derivative strain *Streptomyces coelicolor* M1152Δ*matAB* as an improved cell factory for the metabolic engineering of minimal aromatic polyketides from type II PKS pathways.

**Figure 2:**
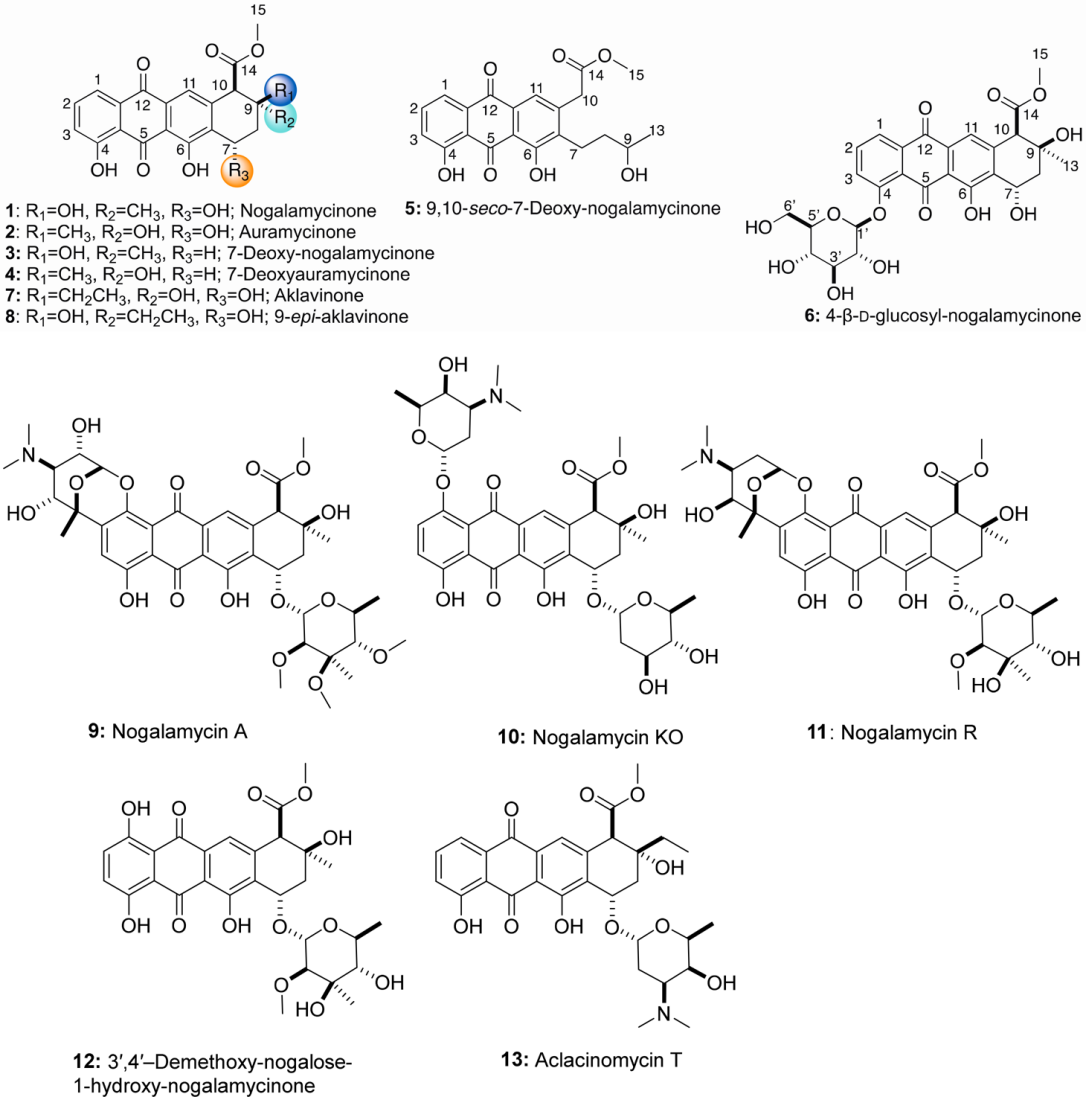
Structures of anthracyclin(on)es described in this work.

In this report, we developed a BioBricks toolkit of promoters, expression vectors, and engineered *Streptomyces* hosts for the metabolic engineering of anthracyclinones from type II PKS pathways. All genes were assembled according to the BioBricks [RFC10] standard. We designed minimal PKS (minPKS) cassettes based on the *snoa123* (C-20 nogalamycin), *aknBCDE2F* (C-21 aclacinomycin), *dpsABGCD* (C-21 doxorubicin), and *oxyABCD* (C-19N oxytetracycline) biosynthetic pathways. First, we compared the production titer resulting from expressing minPKS gene cassettes in different Streptomyces hosts: *Streptomyces lividans* K4–114, *Streptomyces coelicolor* M1146, M1152, and M1154, and *Streptomyces coelicolor* M1152Δ*matAB*. These experiments resulted in the production of the expected aromatic minimal polyketides. Second, we performed combinatorial biosynthesis experiments by coexpressing the minPKS gene cassettes with different combinations of KR/ARO/CYC/OXY genes to optimize metabolic flux toward tricyclic anthracyclinones. Third, we coexpressed different orthologs of *O*-methyltransferases, fourth ring cyclases, and 7-ketoreductases to generate the expected anthracyclinones. Finally, the optimum gene combinations were cloned onto one plasmid to determine the production yields in *S. coelicolor* M1152Δ*matAB*. This work resulted in the production of eight anthracyclinone analogues, including the new compounds 9,10-*seco*-7-deoxy-nogalamycinone and 4-β-d-glucosyl-nogalamycinone and the unexpected 7-deoxy analogues (Figure 2). Finally, the anticancer activity of the different anthracyclinone derivatives was assessed in a mammalian cell viability assay, which revealed that nogalamycinone, auramycinone, and aklavinone had moderate antiproliferative activity (<30 μM IC_50_) against several human cancer cell lines.

## Results and Discussion

### Engineering of the *snoa123* MinPKS into Different *Streptomyces* spp. Hosts

We first synthesized a codon-optimized version of the *Streptomyces nogalater* minimal polyketide synthase (*snoa123*) responsible for the synthesis of the nogalamycin C-20 poly-β-ketothioester (Figure 1). The codon optimization was based on the native codon preference for *S. coelicolor* (Genscript, OptimumGene). Previously, the involvement of *snoa123* in the synthesis of nogalamycin has been confirmed via heterologous expression studies and genetic complementation studies in *Streptomyces* spp. strains.^6,22^ The *snoa123* operon was designed based on principles from the BioBricks [RFC-10] standard: (1) the genes were decoupled from their native translational coupling and synthesized as individual ORFs; (2) the strong BBa_B0034 ribosome binding site was incorporated into the 5′-untranslated region (5′-AAAGAGGAGAAA-3′) to control the rate of translation initiation for each gene in the operon;^23,24^ (3) the genes were cloned to lack internal *Eco*RI, *PstI*, *SpeI*, and *XbaI* restriction sites, and if necessary, silent mutations were engineered in these restriction sites to make the genes compatible with the BioBricks [RFC-10] standard; (4) the genes were given BioBricks prefix (5′-GAATTCGCGGCCGCTTCTAGAG-3′) and suffix (5′-TACTAGTAGCGGCCGCTGCAG-3′) sequences to enable isocaudomer cloning^25^ (Table 1). To facilitate gene expression, we generated a BioBricks compatible version of the ϕC31-based integrating expression vector pSET152^26^ (e.g., pSET152BB) to allow for cloning into *Eco*RI*/PstI* sites and transformation into *S. coelicolor* via intergeneric conjugation (Table 1).^27^ The genes were expressed from the strong constitutive promoter kasOp*.^28,29^ The resulting pSET152BB derivative featured the *snoa123* fused to the kasOp* promoter and was transformed into three heterologous hosts: (1) *S. lividans* K4–114 (lacking the actinorhodin type II polyketide synthase gene cluster);^30^ (2) *S. coelicolor* M1146 (lacking the prodiginine, actinorhodin, coelimycin, and cryptic polyketide gene clusters);^31^ and (3) *S. coelicolor* M1152 (an RNA polymerase B up-regulated mutant of *S. coelicolor* M1146) (Table 2).

**Table 1:** Plasmids Constructed and/or Used in This Study

plasmid	genotype and relevant characteristics	reference
pSET152	*aac3(IV)*, *oriT*, *lacZα*, ΦC31int, *attP*, MCS	(26)
pSET152BB	BioBricks-compatible vector pSET152; *aac3(IV)*^*R*^, *oriT*, ϕC31int, *attP*, MCS	(24)
pENSV1	BioBricks-compatible vector; *aadA*^*R*^, *bla*^*R*^, *oriT*, ϕSV1int, *attP*	(24)
pENTG1	BioBricks-compatible vector; *vph*^*R*^, *bla*^*R*^, *oriT*, ϕTG1int, *attP*	(24)
pOSV808	BioBricks-compatible vector; *hph*^*R*^, *oriT*, VWBint, *attP*, *amilCFP*	(32)
pSnogaori	pKC505-based *E. coli*–*Streptomyces* shuttle vector harboring 30.26 kb fragment of nogalamycin biosynthetic gene cluster; *aac(3)IV*, SCP2* ori, pBR322 *ori*, λ cos sites	Accession No. OM832358,^33^
pSET154BB	pSET152BB with strong 5′-*tt-sbi-A* terminator and 3′-*fd* phage terminator	This study,^8,9^
pENSV3	pENSV1 with strong 5′- ECK120010818-term and 3′- ECK120029600-spy-term terminators	This study,^10^
pENTG3	pENTG1 with strong 5′- L3S2P21-term and 3′- L3S3P41-term terminators	This study,^10^
pSET-S1	wild-type *snoa123* fused to the medium ermE*p promoter; cloned in pSET152BB vector	This study
pSET-S2	wild-type *snoa123* fused to the strong kasOp* promoter; cloned in pSET152BB vector	This study
pSET-S3	codon-optimized *snoa123* fused to the strong kasOp* promoter; cloned in pSET152BB vector	This study
pSET-A1	wild-type *aknBCDE2F* fused to the medium ermE*p promoter; cloned in pSET152BB vector	This study
pSET-A2	wild-type *aknBCDE2F* fused to the strong kasOp* promoter; cloned in pSET152BB vector	This study
pSET-D1	wild-type *dpsABCDG* fused to the medium ermE*p promoter; cloned in pSET152BB vector	This study
pSET-D2	wild-type *dpsABCDG* fused to the medium kasOp* promoter; cloned in pSET152BB vector	This study
pSET-O2	wild-type *oxyABCD* fused to the strong kasOp* promoter; cloned in pSET152BB	This study
pSET-S2S5	kasOp*-snoa123+kasOp*-snoaDEMB cloned in pSET152BB	This study
pSET-D1S5	ermE*p-dpsABCDG+kasOp*-snoaDEMB cloned in pSET152BB	This study
pSET-D2S5	kasOp*-dpsABCDG+kasOp*-snoaDEMB cloned in pSET152BB	This study
pSET-A1S5	ermE*p-aknBCDE2F+kasOp*-snoaDEMB cloned in pSET152BB	This study
pSET-A1S5	kasOp*-aknBCDE2F+kasOp*-snoaDEMB cloned in pSET152BB	This study
pSET-D1D5	ermE*p-dpsABCDG+kasOp*-dpsEFYdnrG cloned in pSET152BB	This study
pSET-D2D5	kasOp*-dpsABCDG+kasOp*-dpsEFYdnrG cloned in pSET152BB	This study
pSET-A1D5	ermE*p-aknBCDE2F+kasOp*-dpsEFYdnrG cloned in pSET152BB	This study
pSET-A2D5	kasOp* -aknBCDE2F+kasOp*-dpsEFYdnrG cloned in pSET152BB	This study
pSET-D1A5	ermE*p-dpsABCDG+kasOp*-aknAE1WX cloned in pSET152BB	This study
pSET-D2A5	kasOp*-dpsABCDG+kasOp*-aknAE1WX cloned in pSET152BB	This study
pSET-A1A5	ermE*p-aknBCDE2F+kasOp*-aknAE1WX cloned in pSET152BB	This study
pSET-A2A5	kasOp*-aknBCDE2F+kasOp*-aknAE1WX cloned in pSET152BB	This study
pSV-S4	kasOp*-snoaDEM cloned in pENSV1	This study
pSV-S5	kasOp*-snoaDEMB cloned in pENSV1	This study
pSV-A4	kasOp*-aknAE1W cloned in pENSV1	This study
pSV-A5	kasOp*-aknAE1WX cloned in pENSV1	This study
pTG-S6	*sp44-snoaLCF* cloned in pENTG1	This study
pTG-S7	*sp44-snoaC+kyc34+snoaF* cloned in pENTG1	This study
pTG-A6	*sp44-aknGHU* cloned in pENTG1	This study
pTG-D6	*sp44-dnrCDE* cloned in pENTG1	This study
pEN10001	kasOp*-*aknBCDE2F*+kasOp*-*aknAE1WX*+*sp44*-*aknGHU* cloned into pSET154BB	This study
pEN10002	kasOp*-*aknBCDE2F*+kasOp*-*aknAE1WX*+*sp44*-*snoaC+kyc34+snoaF* cloned into pSET154BB	This study
pEN10003	kasOp*-snoa123+kasOp*-snoaDEMB+sp44-aknGHU cloned into pSET154BB	This study
pEN10004	kasOp*-snoa123+kasOp*-snoaDEMB+sp44-snoaC+kyc34+snoaF	This study
pRW10000	codon-optimized kasOp*-snoa123+kasOp*-aknAE1WX+sp44-snoaLCF	This study

**Table 2:** Bacterial Strains Used in This Study

strain	genotype or comments	source or reference
*Escherichia coli* JM109	F′ *traD36 proA+B+lacIq* Δ(*lacZ*)M15/ Δ(*lac-proAB*) *gln V44 e14- gyrA96 recA1 relA1 endA1 thi hsdR17*	Promega
*Escherichia coli* ET12567/(pUZ8002)	*dam- dcm- hsdM hsdS hsdR cat tet*; carrying plasmid pUZ8002	(34)
*Streptomyces lividans* K4–114	*pro-2 str-6 SLP2- SLP3- act::ermE*	(30)
*Streptomyces coelicolor* M1146	SCP1- SCP2- Δ*act* Δ*red* Δ*cpk* Δ*cda*	(31)
*Streptomyces coelicolor* M1152	SCP1- SCP2- Δ*act* Δ*red* Δ*cpk* Δ*cda rpoB*(C1298T)	(31)
*Streptomyces coelicolor* M1152Δ*matAB*	SCP1- SCP2- Δ*act* Δ*red* Δ*cpk* Δ*cda rpoB*(C1298T) Δ*sco2961–2962*	This study,^19^

**Figure 3:**
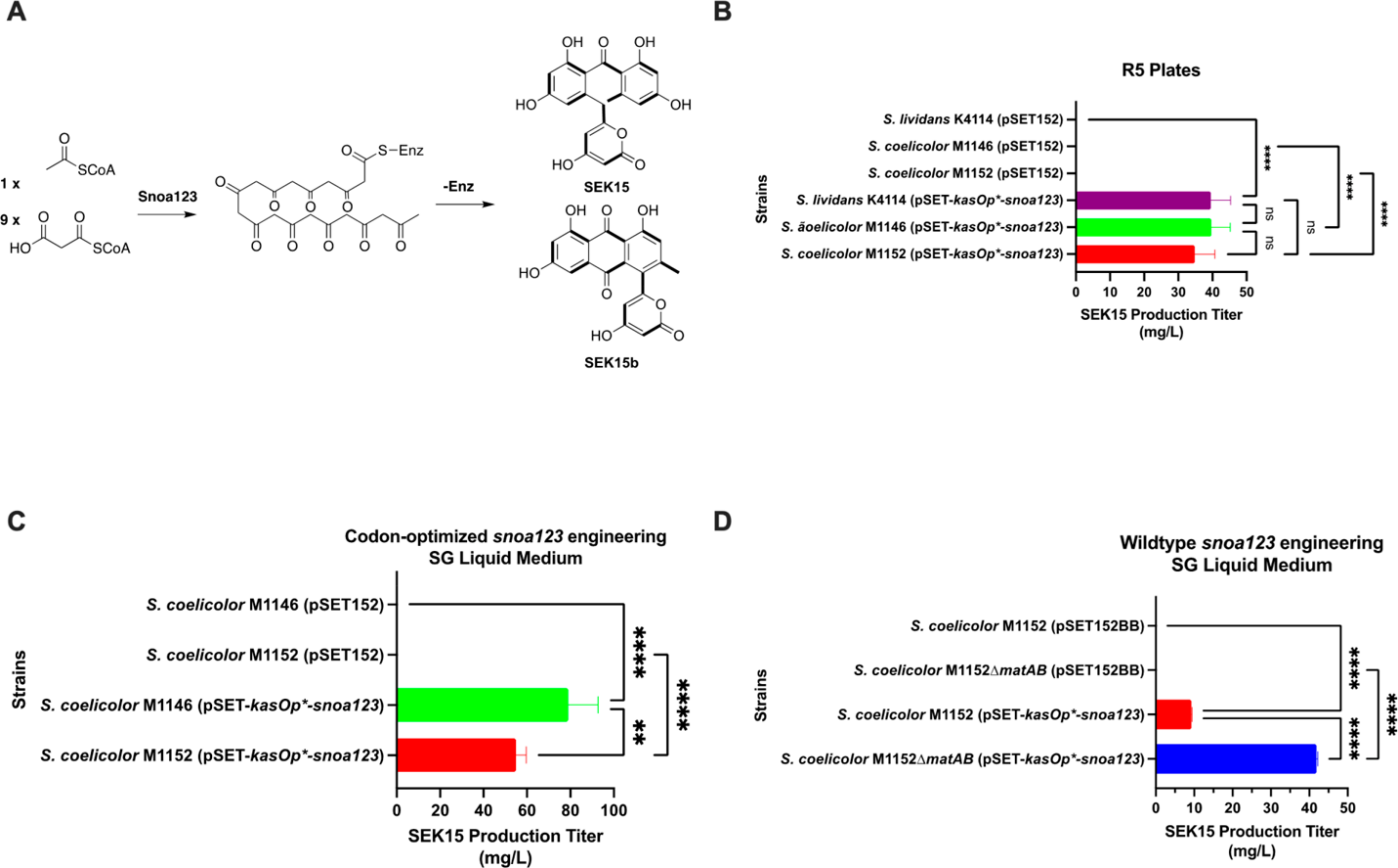
Metabolic engineering of SEK15. (A) Scheme for the biosynthesis of SEK15 via the Snoa123 minPKS. (B) Production titers of SEK15 on R5 solid agar plates from the heterologous expression of codon-optimized *snoa123* in *S. lividans* K4–114, *S. coelicolor* M1146, and *S. coelicolor* M1152. (C) Production titers of SEK15 in SG liquid media shake flask experiments from the heterologous expression of codon-optimized *snoa123* in *S. coelicolor* M1146 and *S. coelicolor* M1152. (D) Production titers of SEK15 in SG liquid media shake flask experiments from the heterologous expression of wild-type *snoa123* in *S. coelicolor* M1152 and *S. coelicolor* M1152Δ*matAB*. Experiments were carried out in double triplicate and the error bars reflect the standard deviation. ANOVA was carried out to determine the statistical significance between strains. The statistically significant comparisons are reflected with asterisks. The statistical significance of observed results was established with a *p* < 0.05. * indicates *p* ≤ 0.05, ** indicates *p* ≤ 0.01, *** indicates *p* ≤ 0.001, and **** indicates *p* ≤ 0.0001.

The heterologous expression of the codon-optimized *snoa123* operon resulted in significant production of yellow-orange pigments on SFM agar plates. Each of the recombinant strains was plated in triplicate on R5 agar plates for 5 days and then extracted to analyze the production of polyketides (Figure 3). Each strain produced copious amounts of known polyketides SEK15 (e.g., C_20_H_16_O_8_, calc. *m*/*z* = 385.0923 [M + H]^+^; found *m*/*z* = 385.0910 [M + H]^+^) and SEK15b (e.g., C_20_H_12_O_8_, calc. *m*/*z* = 381.0610 [M + H]^+^; found *m*/*z* = 381.0595 [M + H]^+^) as determined by HRESI-MS analysis (Supporting Information Figures S11 and S12). *S. lividans* K4–114 also produced large quantities of undesired prodiginines, based on HRESI-MS total ion counts, and therefore this strain was excluded from further experimentation. The production titer of SEK15 was strain-dependent and media-dependent. On R5 agar plates, the strains transformed with empty pSET152BB vector produced no detectable SEK15; however, *S. lividans* K4–114 expressing the construct produced 39.6 mg/L SEK15, *S. coelicolor* M1146 produced 39.7 mg/L SEK15, and *S. coelicolor* M1152 produced 34.8 mg/L SEK15 (Figure 3). In SG liquid media, *S. coelicolor* M1146 and M1152 strains transformed with empty pSET152BB produced no SEK15, whereas *S. coelicolor* M1152 expressing the *snoa123* operon produced 55 mg/L SEK15 and *S. coelicolor* M1146 produced the highest SEK15 titer at 79 mg/L (Figure 3).

### Engineering of *aknBCDE2F*, *dpsABCDG*, and *oxyABCD* MinPKS Operons into *S. coelicolor*

To compare the biosynthetic capacity of *S. coelicolor* M1152 and the *S. coelicolor* M1152Δ*matAB* mutant, heterologous expression experiments were conducted to produce minimally cyclized aromatic polyketides using propionyl-CoA, acetyl-CoA, or malonamyl-CoA starter units. We anticipated that the strains resulting from these experiments would serve as a proof-of-concept for the use of *S. coelicolor* M1152Δ*matAB* as a production host for pathway engineering of anthracyclines. The original *aknBCDE2F* gene annotations from *Streptomyces galilaeus* ATCC 31615 (Accession No. AF257324.2) were revised using next-generation sequencing (Supporting Information, Table S1). Next-generation sequencing of pSnogaori, which encodes the majority of the nogalamycin biosynthetic pathway, identified several mutations in the original annotations of *snoaD*, *snoaE*, *snoaM*, and *snoaB* genes. The revised sequence was deposited in the NCBI database (Accession No. OM832358). Despite several attempts to express codon-optimized versions of the *aknBCDE2F*, *dpsABCDG*, and *oxyABCD* minPKS operons, attempts to produce the expected minimal aromatic polyketides were unsuccessful. These attempts also included the preservation of the native translational coupling and ribosome binding site of the KSα-KSβ subunits, which Liu et al. previously demonstrated to be indispensable for the expression of the *alpABC* and *whiE–III–IV–V* type II PKS subunits in *E. coli*.^35^ As a result, we focused further construct development on the expression of wild-type versions of the *snoa123*, *aknBCDE2F*, *dpsABCDG*, and *oxyABCD* minPKS operons.^36^ The expression of *aknBCDE2F* and *dpsABCDG* operons was expected to produce the C-21 polyketide UWM7 and the *oxyABCD* operon was expected to produce the amidated polyketide WJ85.^37,38^ We cloned the *aknBCDE2F*, *snoa123*, and *oxyABCD* genes under the control of the intermediate strength ermE*p promoter or the strong kasOp* promoter and spliced these cassettes into pSET152BB.^28,39^ The resulting plasmids were transformed into *S. coelicolor* M1152 and *S. coelicolor* M1152Δ*matAB* via intergeneric conjugation. *S. coelicolor* M1152/pSET152BB and *S. coelicolor* M1152Δ*matAB*/pSET152BB were included in the analysis as negative controls.

Expression of the wild-type *snoa123* minPKS in *S. coelicolor* M1152 resulted in the production of 9.2 mg/L SEK15, *S. coelicolor* M1152ΔmatAB harboring the same construct produced 3-fold more SEK15 at 41.8 mg/L (*p* < 0.0001) (Figure 3). The codon-optimized version of *snoa123* was more productive than the wild-type version of *snoa123* (e.g., 79 mg/L), which indicates that codon-optimization likely enhances translation of the Snoa123 minPKS complex and results in greater metabolic flux toward SEK15 via mass action. We previously observed a similar result when comparing wild-type and codon-optimized versions of the valerena-1,10-diene synthase, *VoTPS1*, in *E. coli*.^40^ Expression of the codon-optimized version resulted in a 3-fold greater production titer of valerena-1,10-diene than the wild-type version. Additionally, this result demonstrates that the dispersed growth phenotype of *S. coelicolor* M1152Δ*matAB* greatly enhanced polyketide production.

We are only beginning to unravel the mechanisms that control morphogenesis of streptomycetes in submerged cultures. Productivity of streptomycetes in industrial fermentation depends on the mycelial morphology in a product-dependent manner; in other words, less favorable growth conditions may have to be accepted to obtain good productivity.^41−42^ Considering that it is hard to predict how production responds to changes in growth and morphology, we need to have more tools at our disposal to change the growth characteristics and thus optimize the chance of success. Overexpression of the cell division activator SsgA increases fragmentation of streptomycetes, which leads to faster growth. A strain of *S. coelicolor* overexpressing SsgA produced large amounts of prodigionines, but hardly any actinorhodin.^43^ However, SsgA affects the intracellular architecture, making the hyphae less robust. Deletion of *matAB* prevents the production of poly-*N*-acetylglucosamine (PNAG), an EPS that “glues” the hyphae together, promoting pellet formation.^19,21^ Deletion of *matAB* in *S. coelicolor* M1152 significantly resulted in a dispersed growth phenotype (data not shown) and led to better productivity. The more PNAG is produced the larger the pellets, and thus the technology may be widely applicable.

**Figure 4:**
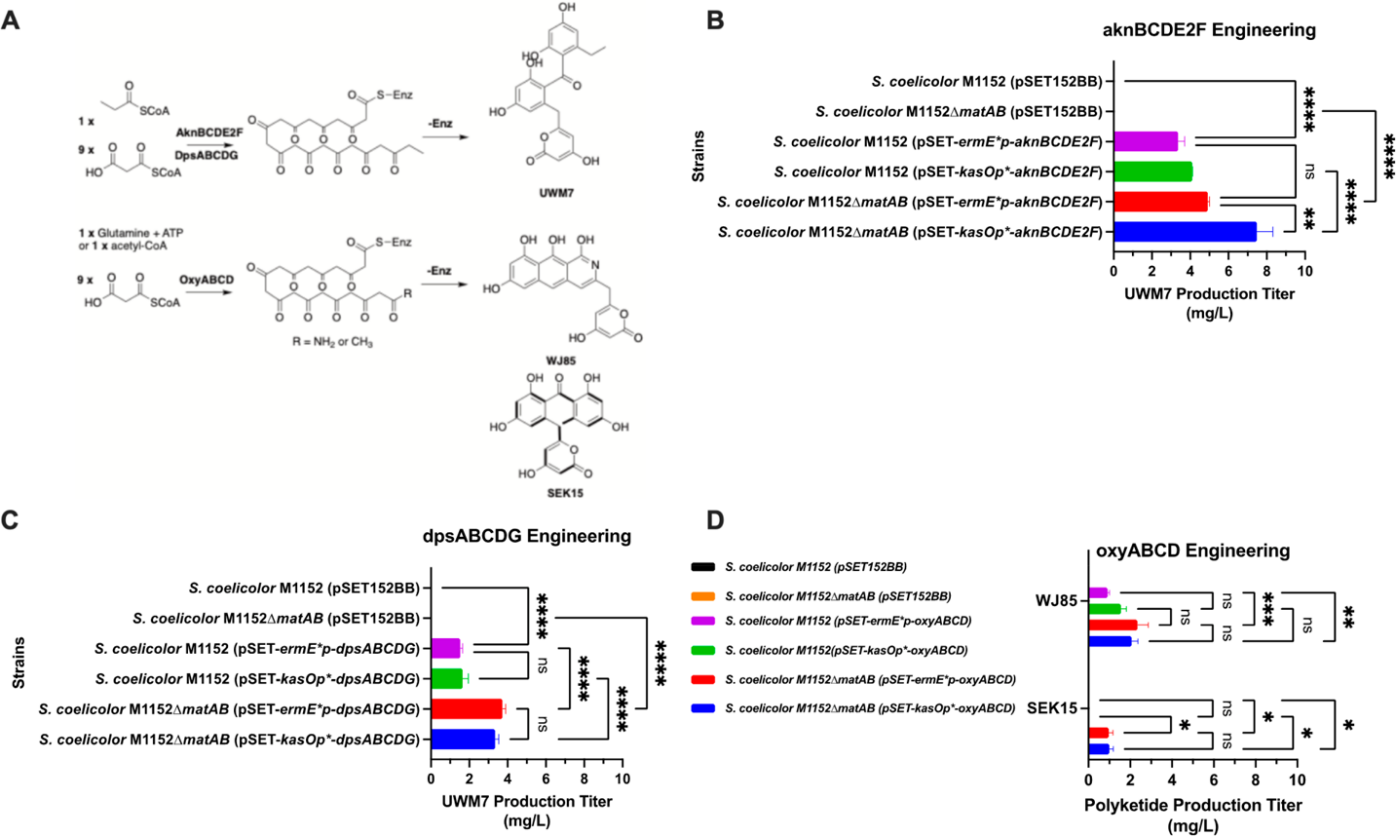
Engineering of UWM7 and WJ85 minimal aromatic polyketides. (A) Biosynthesis scheme for the synthesis of UWM7 from the DpsABCDG/AknBCDE2F minPKS and WJ85 from the OxyABCD minPKS. (B) Production titers of UWM7 resulting from the engineering of pSET-ermE*p-aknBCDE2F and pSET-kasOp*-aknBCDE2F in *S. coelicolor* M1152 and M1152Δ*matAB*. (C) Production titers of UWM7 resulting from the engineering of pSET-ermE*p-dpsABCDG and pSET-kasOp*-dpsABCDG in *S. coelicolor* M1152 and M1152Δ*matAB*. (D) Production titers of SEK15 and WJ85 resulting from the engineering of pSET-ermE*p-oxyABCD and pSET-kasOp*-oxyABCD in *S. coelicolor* M1152 and M1152Δ*matAB*. ANOVA was carried out to determine the statistical significance between strains. The statistically significant comparisons are reflected with asterisks. The statistical significance of observed results was established with a *p* < 0.05. * indicates *p* ≤ 0.05, ** indicates *p* ≤ 0.01, *** indicates *p* ≤ 0.001, and **** indicates *p* ≤ 0.0001.

Engineering of the *aknBCDE2F* and *dpsABCDG* operons in *S. coelicolor* M1152Δ*matAB* and *S. coelicolor* M1152 resulted in the expected production of UWM7 as the major metabolite at production titers of 1–7.5 mg/L in SG shake flask experiments (Figure 4). UWM7 exhibited a retention time of 8.42 min with a UV maximum of 290 nm and the expected mass in ESI+ mode [M + H]^+^ = 399 *m*/*z*, as previously described (Supporting Information, Figure S13).^38^ In each case, the corresponding *S. coelicolor* M1152Δ*matAB* resulted in a statistically significant increase in UWM7 production titers as compared to *S. coelicolor* M1152 (*p* ≤ 0.01), and the *aknBCDE2F* operon was twice as productive as the *dpsABCDG* operon. The best construct featured the kasOp* promoter fused to the *aknBCDE2F* operon (7.5 mg/L UWM7), which resulted in 33% greater UWM7 as compared to the ermE*p promoter fused to the *aknBCDE2F* operon (*p* ≤ 0.05) (Figure 4).

Engineering of *oxyABCD* into the host strains resulted in the production of WJ85 as determined via HPLC-MS analysis (Supporting Information, Figure S14). WJ85 exhibited a retention time of 9.54 min with a UV maximum at 290 nm, and it exhibited a parental mass of [M + H]^+^ = 368 *m*/*z*, as previously described.^37^ The ermE*p-oxyABCD construct resulted in the production of 2.3 mg/L WJ85 in *S. coelicolor* M1152Δ*matAB*, as compared to the kasOp*-oxyABCD construct, which resulted in the production of 2.0 mg/L WJ85, although the difference between these two promoters was not statistically significant in this case (Figure 4). Due to the deletion of the Δ*matAB* operon, the *S. coelicolor* M1152Δ*matAB* strain accumulated 1 mg/L SEK15, which was not detected in *S. coelicolor* M1152. This result was interpreted to mean that *S. coelicolor* M1152*ΔmatAB* strain exhibited improved production characteristics for minimal aromatic polyketides. The differences in production titer of WJ85 and SEK15 between M1152 and M1152*ΔmatAB* were determined to be statistically significant based on ANOVA statistical analysis (Figure 4). Due to the low production yields of the *oxyABCD* operon, we did not pursue its use any further. In summation, we chose *S. coelicolor* M1152Δ*matAB* for further experiments to build out anthracyclinone pathways.

### Engineering of Ketoreductases, Aromatases, and Cyclases

**Figure 5:**
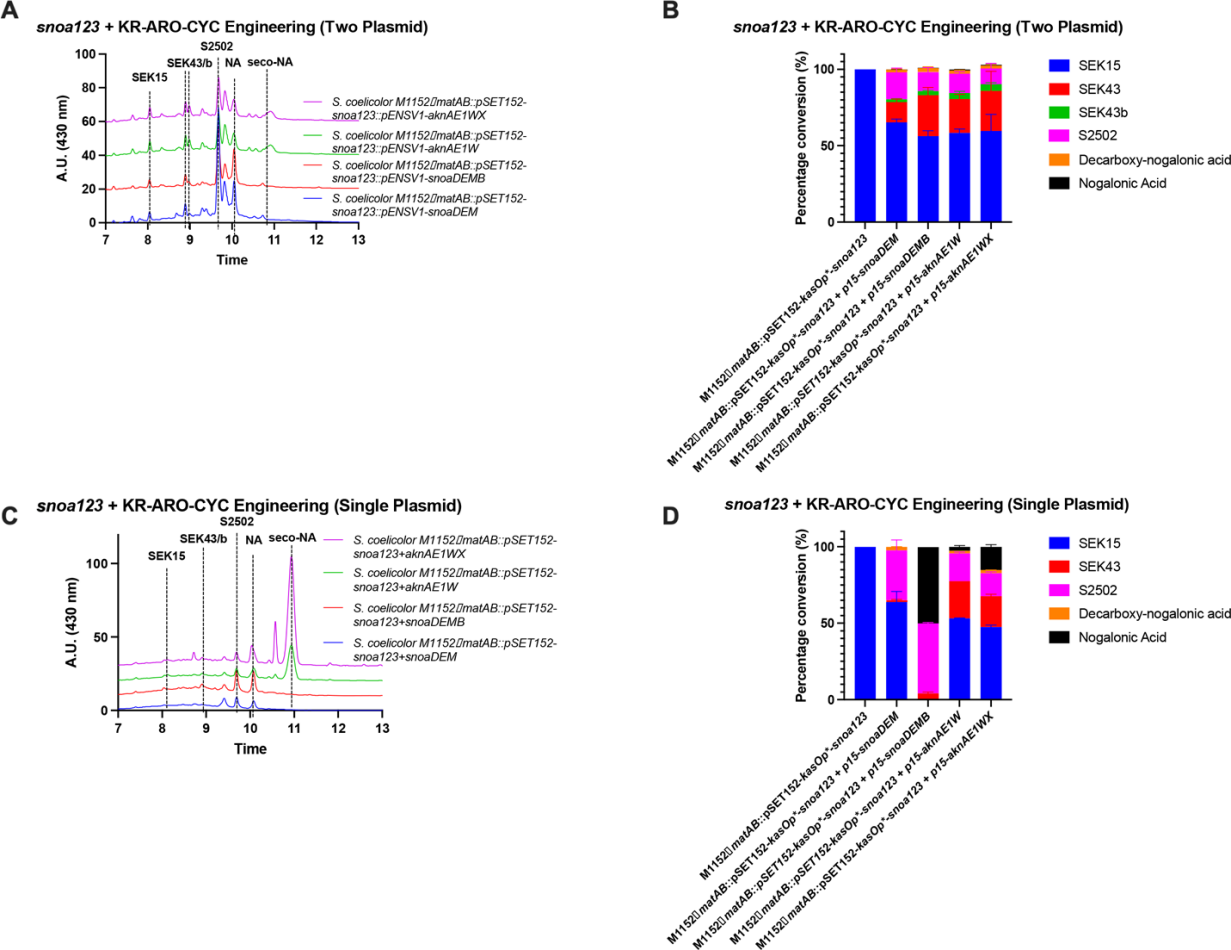
Engineering of KR/ARO/CYC cassettes with the *snoa123* minPKS. (A) Chromatograms of metabolites from two-plasmid *snoa123* and KR/ARO/CYC heterologous expression experiments. (B) Disposition of metabolites from two-plasmid *snoa123* and KR/ARO/CYC heterologous expression experiments. (C) Chromatograms of metabolites from one-plasmid *snoa123* and KR/ARO/CYC heterologous expression experiments. (D) Disposition of metabolites from one-plasmid *snoa123* and KR/ARO/CYC heterologous expression experiments.

We next engineered reducing ketoreductase (KR), aromatase (ARO), and cyclase (CYC) gene cassettes into *S. coelicolor* M1152Δ*matAB* to generate tricyclic anthracyclinones (Figure 5). Starting from the enzyme-tethered poly-β-ketothioester synthesized by Snoa123, the 3-oxoacyl-[acyl carrier protein] ketoreductase (SnoaD/AknA) reduces the poly-β-ketothioester at 9-position, followed by C7–C12 first ring cyclization and aromatization (SnoaE/AknE1), C5–C14 s ring cyclization and C3–C16 third ring cyclization by the second-third ring cyclase (SnoaM/AknW), and C-12 oxidation by the anthrone oxygenase (SnoaB/AknX) (Figure 1). Genetic cassettes for *aknAE1W*, *aknAE1WX*, *snoaDEM*, and *snoaDEMB* were codon-optimized and cloned as described above under the control of the strong *p15* promoter from *Streptomyces albus* and cloned into a separate integrating vector pENSV1 for two plasmid coexpression or spliced into the pSET152BB-kasOp*-snoa123 construct for expression of the entire cassette on a single plasmid.^24,42^ Heterologous expression of *snoa123+snoaDEM*, *snoa123+snoaDEMB*, *snoa123+aknAE1W*, and *snoa123+aknAE1WX* on two plasmids resulted in the production of SEK15, and C7–C12 cyclized metabolites SEK43 and SEK43b, C7–C12 and C9–C14 cyclized metabolite S2502, and tricyclic anthracyclinones nogalonic acid and its rearrangement product decarboxy-nogalonic acid, as expected (Figure 5, Supporting Information, Figure S10). These metabolites were confirmed based on a comparison to authenticated biosynthetic standards from *S. lividans* TK24/pSY21c.^11^ Percent conversion was calculated based on the integration of the peak areas at λ = 290 nm since the polyketides exhibit similar molar absorptivity coefficients at this wavelength (Figure 5).^11^ This finding supports the role of AknA as a ketoreductase, AknE1 as an ARO/CYC, AknW as a second and third-ring cyclase, and AknX as an anthrone oxygenase. Previously, Chung et al. demonstrated that the AknX anthrone oxygenase enhances the oxidation of emodin anthrone to emodin anthraquinone, although C-12 oxidation can also occur spontaneously.^10^ Expression of the C-12 anthraquinol oxidase increased the formation of correctly cyclized tricyclic anthracyclinone intermediate, which confirms its essential role in the oxidation of nogalonic acid. Coexpression of the *snoa123* minPKS genes with the KR/ARO/CYC cassettes on one plasmid resulted in higher metabolic flux toward nogalonic acid (Figure 5). One possible explanation for this was offered by Yang et al., who observed that expression of type II PKS genes on the same plasmid in *E. coli* resulted in higher production of carminic acid.^43^ Yang et al. postulated that colocalization of the minPKS genes and cyclase genes enhances the formation of the transient polyketide synthase complex, enhancing the production titer. Interestingly, the coexpression of *snoa123+aknAE1WX* on the same plasmid construct resulted in approximately 15% conversion to nogalonic acid. Expression of the *snoa123+snoaDEMB* construct resulted in a 50% conversion to nogalonic acid (Figure 5). These results demonstrate that the systematic testing of different orthologous gene combinations can provide additional insight into the compatibility of heterologous polyketide synthase components from related pathways. In addition, this approach can be used to provide biochemical evidence for gene products from uncharacterized pathways or for which only bioinformatic description is provided (e.g., AknA, AknE1, AknW).

**Figure 6:**
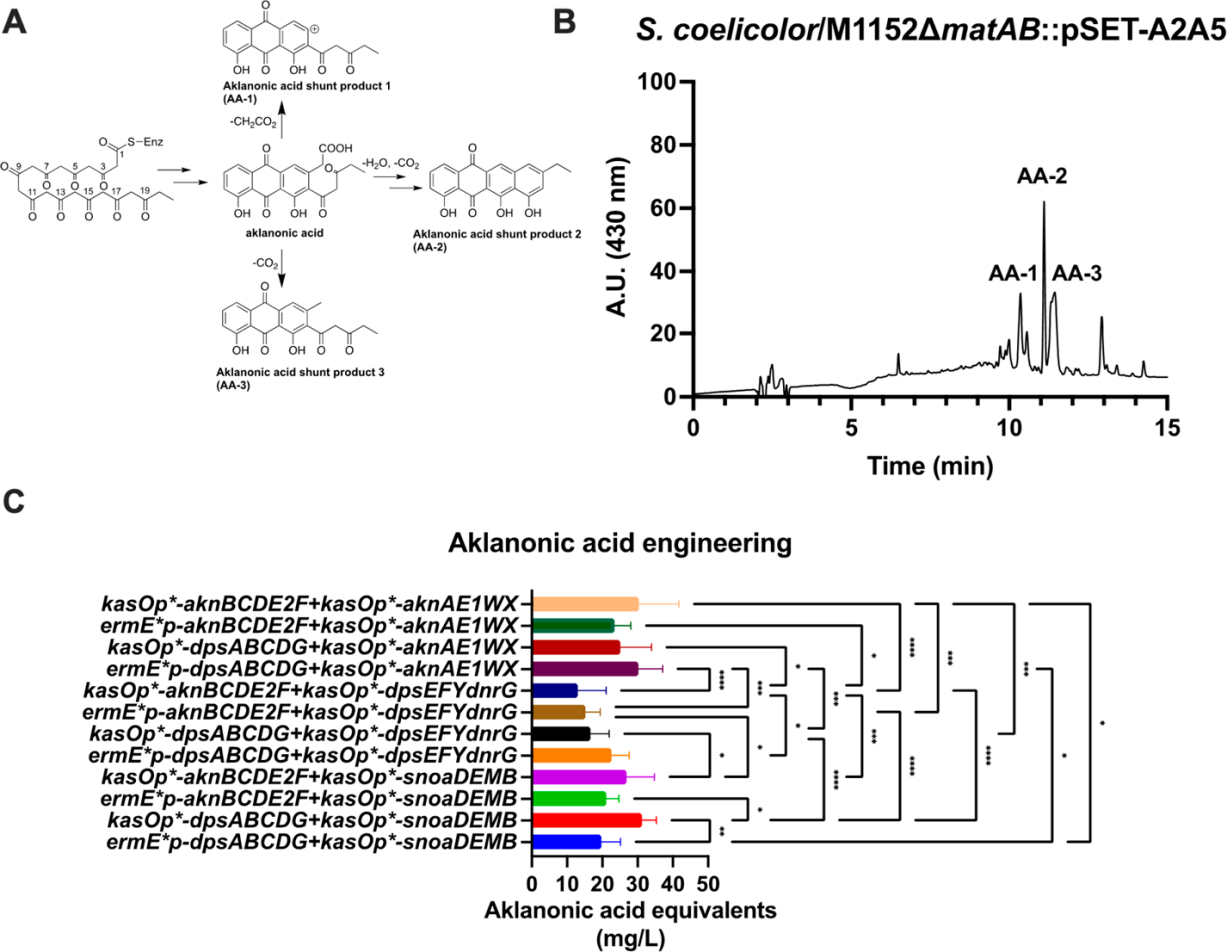
Engineering of aklanonic acid via combinatorial biosynthesis. (A) Scheme depicting degradation of aklanonic acid to its three detected shunt products (AA-1, AA-2, and AA-3). (B) Chromatogram of aklanonic acid degradation metabolites produced by a representative strain. (C) Aklanonic acid production titers resulting from full-factorial combinatorial biosynthesis of minPKS and KR/ARO/CYC/OXY genes. Error bars depict standard deviation. ANOVA was carried out to determine the statistical significance between strains. The statistically significant comparisons are reflected with asterisks. The statistical significance of observed results was established with a *p* < 0.05. * indicates *p* ≤ 0.05, ** indicates *p* ≤ 0.01, *** indicates *p* ≤ 0.001, and **** indicates *p* ≤ 0.0001.

We next decided to test the potential of BIOPOLYMER for combinatorial biosynthesis by carrying out a full factorial experiment for the biosynthesis of aklanonic acid. We designed this experiment by cloning combinations of the *aknBCDE2F* and *dpsABCDG* minPKS operons with the *aknAE1WX*, *snoaDEMB*, and *dpsEFY+dnrG* KR/ARO/CYC/OXY operons. The KR/ARO/CYC/OXY operons were all cloned under the control of the strong kasOp* promoter since the strong transcriptional regulation of tailoring genes correlates with improved cyclic product formation and diminished shunt product accumulation in the actinorhodin pathway.^44^ Expression of *aknBCDE2F* and *dpsABCDG* with KR/ARO/CYC/OXY genes resulted in the accumulation of three aklanonic acid-derived shunt products (AA-1, AA-2, and AA3), as previously described (Figure 6).^4^ Mass spectroscopy confirmed the production of the three degradation products: AA-1, [M – H]^−^ = 337 *m*/*z*; AA-2, [M – H]^−^ = 333 *m*/*z*; AA-3, [M – H]^−^ = 351 *m*/*z* (Supporting Information, Figure S85). All combinations assessed resulted in the production of aklanonic acid, which indicates that there is a significant biosynthetic collaboration between minPKS enzymes and ketoreductase/cyclase enzymes between the nogalamycin, daunorubicin, and aclacinomycin biosynthetic pathways (Figure 6). The production titers of aklanonic acid varied between 20 mg/L to 35 mg/L with the best combinations consisting of recombinant PKS systems *dpsABCDG+snoaDEMB* and *dpsABCDG+aknAE1WX* and the native *aknBCDE2F+aknAE1WX* pathway (Figure 6). We performed an analysis of variance (ANOVA) of all 12 strains to determine if the differences in production titer between the different strains were statistically significant (Figure 6). The ANOVA indicated that the observed results between many of the comparisons were statistically significant, which provides good evidence that the combinatorial biosynthesis of the minPKS and cyclase gene cassettes was responsible for the observed differences in production titer.

### Engineering of Nogalamycinone and Structural Characterization of Anthracyclinones

We next decided to complete the aglycone engineering pathway by introducing the nogalonic acid methyltransferase, fourth-ring cyclase, and 7-ketoreductase from the nogalamycin biosynthetic pathway (Figure 1). The resulting construct, pSET152BB-ermE*p-snoa123+kasOp*-aknAE1WX+sp44-snoaLCF was transformed into *S. coelicolor* M1152Δ*matAB* and assessed for production of nogalamycinone. The order of genes in the *snoaLCF* operon was determined to be important for the complete conversion of nogalonic acid to nogalamycinone. Initially, cloning of the *snoaC* before *snoaL* and subsequent expression of the resulting plasmid yielded incomplete conversion from nogalonic acid to nogalamycinone. The resulting strains produced six compounds that could be detected via HPLC-MS, including nogalamycinone (**1**) which could be confirmed via comparison to an authentic HPLC standard (Figure S18). Interestingly, a new early eluting product was also detected that corresponded to a glucosylated derivative of nogalamycinone, as determined by mass spectrometric analysis in ESI– mode ([M – H]^−^ = 559 *m*/*z*). The resulting strain was scaled up in a 5 L SG shake flask fermentation, extracted with 3 × 5 L of ethyl acetate, and fractionated via SiO_2_ column chromatography in chloroform:methanol systems on a Teledyne Combiflash 100 auto purification system. A second 2 L fermentation of the strain was carried out to isolate the unknown glucosylated nogalamycinone derivative. Compounds **1**–**6** were purified from additional prep-HPLC experiments.

The physicochemical properties of compounds **1**–**6** have been summarized in the experimental section (Supporting Information, Figures S16–S82, Tables S3 and S4). The chemical structures of the known anthracyclines nogalamycinone (**1**), auramycinone (**2**), 7-deoxy-nogalamycinone (**3**), and 7-deoxyauramycinone (**4**) were established by ^1^H NMR, ^13^C NMR, ^1^H,^1^H-COSY, ^1^H,^13^C-HMBC, and ^1^H,^1^H-NOESY NMR spectroscopic measurements (Supporting Information, Tables S3 and S4, Figures S16 and S17). Unexpectedly, both nogalamycinone and auramycinone were produced, which result from fourth ring cyclization reactions that generate the 9(*S*) and 9(*R*) products.^13,45^ Compounds **1** and **2** were produced in an approximately 2:1 ratio, as were their shunt products **3** and **4**, which indicates that nogalamycinone and 7-deoxynogalamycinone were the preferred enzymatic products in the strain.

It is generally accepted that the codon-optimization of genes is known to improve translational efficiency by substituting rare codons in an mRNA sequence for preferred codons with abundant tRNAs, which leads to an increase in the concentration of the protein.^46^ What is less understood is whether codon-optimization disrupts information that is present in wild-type mRNAs (e.g., rare codons that slow down the translation or that influence translation via binding to rRNA), which could impact protein conformation and function.^47^ Hu et al. verified 342 synonymous codon variants of the scFv antibody and observed widely varying production titers, solubility, and binding affinity (e.g., ranging from no binding affinity to 10^–8^ M), while all proteins encoded the same original amino acid sequence.^48^ Sander et al. demonstrated that synonymous codon changes in a fluorescent protein impacted the fluorescence and can alter the folded structure due to differences in the rate of translation and folding of the *N-* and *C-*termini.^49^ It could be hypothesized that the production of **3** and **4** by codon optimized SnoaL could be due to protein conformational differences from the wild-type SnoaL which impact the binding of NAME in the active site, but this must be examined further.

The formation of **3**, **4**, and **5** requires the elimination of the 7-hydroxyl group, possibly due to the presence of a promiscuous CytA-like enzyme that is present in *S. coelicolor*. Gui et al. have characterized CytA as a promiscuous anthracycline-inactivating enzyme that reduces the C-7 position for a broad variety of anthracyclines in the cytorhodin pathway according to an NADH-dependent mechanism.^50^

Compound **5** was obtained as a yellow solid and displayed UV–vis characteristics like metabolites **1**–**4**. The molecular formula of **5** was established as C_21_H_20_O_7_ based on (+)- and (−)-HRESIMS indicative of two additional protons as compared to compounds **3** and **4** (Supporting Information, Figure S64 and S65). Comparison of the ^1^H and ^13^C NMR of **5** and **3**/**4** (Supporting Information, Tables S3 and S4) revealed **5** to lack the C-9/C-10 bond connection and the methine proton signal at C-10 position (CH-10) in compounds **3**–**4** was replaced by CH_2_ at δ_H_ 3.83 (CH_2_-10). In addition, an additional oxygenated methine signal was observed at δ_H_ 3.80 (m, CH-9) in compound **5**. Furthermore, the singlet methyl signals in compounds **3**/**4** were replaced by a doublet methyl signal in **5** at δ_H_ 1.22 (d, 6.2). This was consistent with the presence of ^1^H–^1^H COSY correlations CH_3_-13/H-9, H-9/CH_2_-8, CH_2_-8/CH_2_-7 for **5** (Supporting Information, Figure S16). All the remaining 2D-NMR (^1^H,^1^H-COSY, HMBC, and NOESY) correlations are in full agreement with structure **5** (Supporting Information, Figures S63–S72). Based on the cumulative spectroscopic data, **5** differed solely from those of compounds **3**-**4** via their C-9/C-10 bond connection. Consistent with the cumulative 1D and 2D NMR data analysis, structure **5** was established as depicted in Figure 1 and named 9,10-*seco*-7-deoxy-nogalamycinone (**5**).

Compound **6** was isolated as a yellow solid and displayed UV–vis characteristics like anthracyclinones **1**–**4**. The molecular formula of **6** was established as C_27_H_28_O_13_ based on (+) and (−)-HRESIMS, with Δ*m*/*z* = 162 higher than those of **1**, indicative of the presence of an extra hexose moiety in **6** (Supporting Information, Figures S74 and S75). Based on the ^1^H NMR, ^13^C NMR, NOESY, and ^1^H,^1^H-COSY data, the carbohydrate was unambiguously assigned as d-glucose (Supporting Information, Table S5 and Figure S17). Compared to **1**, the ^13^C/^1^H/HSQC NMR of **6** (Supporting Information, Table S1–S3) highlighted the presence of additional signals for the *O*-glycoside moiety in compound **6**. Based on the ^1^H NMR, ^13^C NMR, NOESY, and ^1^H,^1^H-COSY data, the carbohydrate was unambiguously assigned as d-glucose (Supporting Information, Table S5, Figure S17 and Figures S76−S82). The connection of the sugar moiety at the 4-position was established based on the observed critical HMBC correlation from H-1′ (δ_H_ 5.12, d, *J* = 7.7 Hz) to C-4 (δ_C_ 160.2). All the remaining 2D-NMR (^1^H,^1^H-COSY, HMBC, and NOESY) correlations are in full agreement with structure **6** (Supporting Information, Figure S17 and Figures S76–S82). As a new natural product and closely related to **1**, compound **6** was designated as 4-β-d-glucosyl-nogalamycinone (**6**).

### Full Pathway Engineering of Anthracyclinones

**Figure 7:**
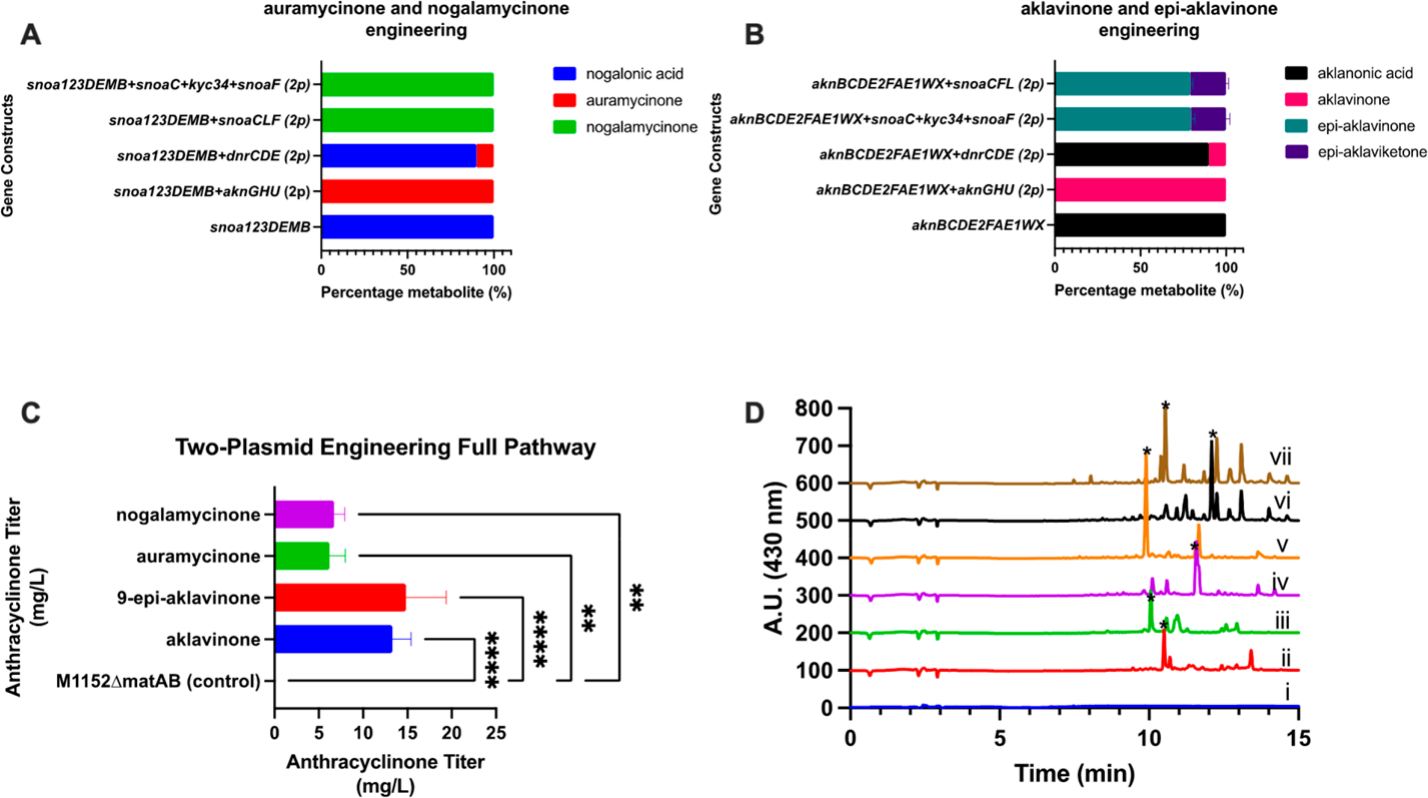
Full pathway engineering of anthracyclinone polyketide synthases. Engineering of methyltransferase, fourth-ring cyclase, and ketoreductase genes furnished anthracyclinone pathways in *S. coelicolor* M1152Δ*matAB*. (A) *S. coelicolor* M1152Δ*matAB*::pS2S5 was complemented with different constructs to biosynthesize different amounts of **1** and **2**. (B) *S. coelicolor* M1152Δ*matAB*::pA2A5 was complemented with different constructs to biosynthesize different amounts of **7** and **8**. (C) Production titer of anthracyclinones in strains expressing the entire pathway on two plasmids. Strains were grown in SG-TES liquid media for 5 days. Error bars reflect the SD of six replicates. Experimental groups were compared using a one-way ANOVA test to determine statistical significance. The statistical significance of observed results was established with a *p* < 0.05. * indicates *p* ≤ 0.05, ** indicates *p* ≤ 0.01, *** indicates *p* ≤ 0.001, and **** indicates *p* ≤ 0.0001. (D) HPLC chromatogram traces of different strains at 430 nm with major metabolites highlighted with an asterisk (*): (i) *S. coelicolor* M1152Δ*matAB*::pSET152; (ii) *S. coelicolor* M1152Δ*matAB*::A2A5 (aklanonic acid); (iii) *S. coelicolor* M1152Δ*matAB*::S2S5 (nogalonic acid); (iv) *S. coelicolor* M1152Δ*matAB*::S2S5A6 (auramycinone); (v) *S. coelicolor* M1152Δ*matAB*::S2S5S6 (nogalamycinone); (vi) *S. coelicolor* M1152Δ*matAB*::A2A5A6 (aklavinone); (vii) *S. coelicolor* M1152Δ*matAB*::A2A5S6 (9-*epi*-aklavinone).

We decided to complete the pathway engineering of other anthracyclinones, including auramycinone (**2**), aklavinone (**7**), and 9-*epi*-aklavinone (**8**). Fujiwara et al. isolated auramycinone as a C-20 anthracyclinone from *S. galilaeus* OBB-111, and aklavinone is a C-21 anthracyclinone that serves as the backbone for doxorubicin and aclacinomycin A.^51,52^ 9-*epi-*Aklavinone was reported as a hybrid anthracyclinone resulting from the heterologous expression of *snoaL* from the nogalamycin biosynthetic pathway in a mutant strain of *S. peucetius* M18.^5^ Using the strains *S. coelicolor* M1152Δ*matAB*::pS2S5 (nogalonic acid producer) and *S. coelicolor* M1152Δ*matAB*::pA2A5 (aklanonic acid producer) as hosts, we cloned different combinations of *O*-methyltransferase genes (*aknG*, *dnrC*, *snoaC*), fourth-ring cyclases (*dnrD*, *kyc34*, *aknH*), and 7-ketoreductases (*snoaF*, *dnrE*, *aknU*) under the control of the strong *sp44* promoter and cloned them into the TG1-actinophage integrating vector pENTG1.^24^ In brief, pENTG1 is a vector that encodes *bla* and *vph* for ampicillin and viomycin selection, respectively, and a codon-optimized version of the TG1 integrase and corresponding *attP* site for integration into the *S. coelicolor* TG1 *attB* chromosomal locus at a single copy.^53,54^ The resulting vectors were expected to result in production of 9*(S)*-configured anthracyclinones (e.g., pENTG1-*sp44-snoaC+kyc34+snoaF*, pENTG1-*sp44-snoCLF*) or 9(*R*)-configured anthracyclinones (e.g., pENTG1-*sp44-dnrCDE* and pENTG1-*sp44-aknGHU*). Coexpression of these vectors in either of the two strains resulted in the production of the expected anthracyclinones (Figure 7). To produce nogalamycinone, *snoaLCF* and *snoaC+kyc34+snoaF* both resulted in the complete conversion of nogalonic acid. This result also provides experimental proof that *kyc34* from the keyicin biosynthetic pathway encodes a NAME cyclase, like *snoaL*.^55^ Similarly, coexpression of *aknGHU* in *S. coelicolor* M1152Δ*matAB*::pS2S5 resulted in the complete conversion of nogalonic acid to **2**. For the production of **7**, when *S. coelicolor* M1152Δ*matAB*::pA2A5 was complemented with *aknGHU*, complete conversion of aklanonic acid was observed. Lastly, the complementation of the aklanonic acid producer with either *snoaLCF* or *snoaC+kyc34+snoaF* resulted in approximately 80% conversion to 9-*epi*-aklavinone. This experiment demonstrates that the BIOPOLYMER system can be used to produce all the naturally occurring reduced anthracyclinone analogues.

**Figure 8:**
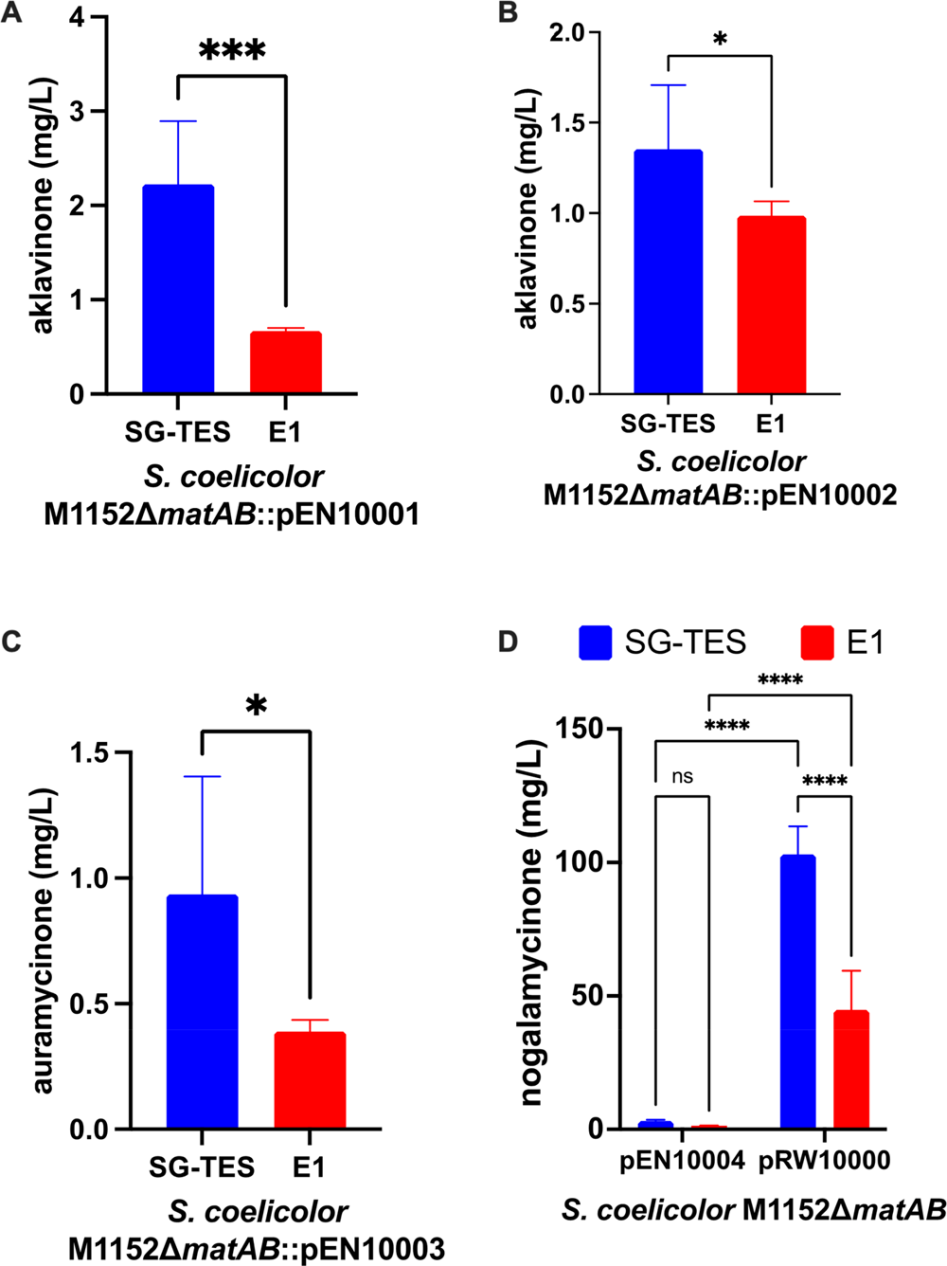
Production titers resulting from the expression of anthracyclinone pathways on a single plasmid. *S. coelicolor* M1152Δ*matAB* was transformed with vectors (A) pSET154BB-kasOp*-aknBCDE2F+kasOp*-aknAE1WX+sp44-aknGHU (pEN10001, aklavinone pathway), (B) pSET154BB-kasOp*-aknBCDE2F+kasOp*-aknAE1WX+sp44-snoaC+kyc34+snoaF (pEN10002, 9-*epi*-aklavinone pathway), (C) pSET154BB-kasOp*-snoa123+kasOp*-snoaDEMB+sp44-aknGHU (pEN10003, auramycinone pathway), or (D) pSET154BB-kasOp*-snoa123+kasOp*-snoaDEMB+sp44-snoaC+kyc34+snoaF (pEN10004, wild-type nogalamycinone pathway) or pSET152BB-kasOp*-snoa123+kasOp*-aknAE1WX + *sp44-snoaCLF* (pRW10000, codon-optimized nogalamycinone pathway) and grown in shake flasks of SG-TES or E1 liquid media for 5 days. Error bars reflect the standard deviation of six replicates. Post hoc Student’s *t* tests were performed to determine statistical significance. The statistical significance of observed results was established with a *p* < 0.05. * indicates *p* ≤ 0.05, ** indicates *p* ≤ 0.01, *** indicates *p* ≤ 0.001, and **** indicates *p* ≤ 0.0001.

Next, we cloned the aklavinone, 9-*epi*-aklavinone, auramycinone, and nogalamycinone pathways onto a single integrating plasmid. To accomplish this, a new version of pSET152 was cloned in which the strong synthetic *tt-sbi-A* transcriptional terminator from *Mycobacterium tuberculosis* and the strong *fd* phage terminator were inserted 5′ and 3′ of the BioBricks cloning sites,^56−58^ which was designated as pSET154BB (Supporting Information, Table S1). The resulting plasmids encoding aklavinone (e.g., pSET154BB-kasOp*-aknBCDE2F+kasOp*-aknAE1WX+sp44-aknGHU), auramycinone (pSET154BB-kasOp*-snoa123+kasOp*-snoaDEMB+sp44-aknGHU), and nogalamycinone (pSET154BB-kasOp*-snoa123+kasOp*-snoaDEMB+sp44-snoaC+kyc34+snoaF) were transformed into *S. coelicolor* M1152Δ*matAB* to quantify the production titers of the intended anthracyclinones. In addition, we also transformed in the nogalamycinone pathway plasmid pSET152BB-kasOp*-snoa123+kasOp*-aknAE1WX + *sp44-snoaCLF*, which was used to isolate compounds **1**–**6** for structure elucidation. The strains were fermented in SG-TES media and E1 media, which are two media used in our laboratories to produce polyketides. In the case of the first four plasmids for aklavinone, 9-*epi-*aklavinone, auramycinone, and nogalamycinone, the yields of anthracyclinones were lower than those obtained with the two-plasmid expression system (Figure 8). *S. coelicolor* M1152Δ*matAB* harboring these constructs produced a mean production titer of 2.22 mg/L aklavinone in SG-TES media and 0.67 mg/L in E1 media, 1.35 mg/L 9-*epi*-aklavinone in SG-TES media and 0.98 mg/L 9-*epi-*aklavinone in E1 media, 0.94 mg/L auramycinone in SG-TES media and 0.39 mg/L auramycinone in E1 media, and 3.22 mg/L nogalamycinone in SG-TES media and 1.32 mg/L nogalamycinone in E1 media. In contrast, the fully codon-optimized construct pSET152BB-kasOp*-snoa123+kasOp*-aknAE1WX+*sp44-snoaCLF* resulted in the production of approximately 33-fold higher levels of nogalamycinone at 103 mg/L in SG-TES media and 45 mg/L in E1 media E1 media. The yields of anthracyclinones indicate that the metabolic flux is controlled by the expression of the minimal polyketide synthase. This is especially highlighted by the greatly increased production titer of nogalamycinone resulting from the fully codon-optimized nogalamycinone construct, which could be explained by the enhanced translation of the Snoa123 minPKS complex. This result indicates that additional optimization of the *aknBCDE2F* minPKS cassette is still required. Several strategies could be pursued, including codon-optimization, additional promoter engineering, multiplexed site-specific genome engineering (MSGE) to increase the number of ϕC31 *attB* sites in the chromosome, and even engineering of the endogenous housekeeping sigma factor *σ*^*hrdB*^ to establish a self-sustaining production system (*StSS*).^59−61^ As determined by Student’s *t-*test, SG-TES media was better than E1 media to produce all four anthracyclinones in this strain (*p* ≤ 0.05).

### Human Cancer Cell Viability Assays

**Figure 9:**
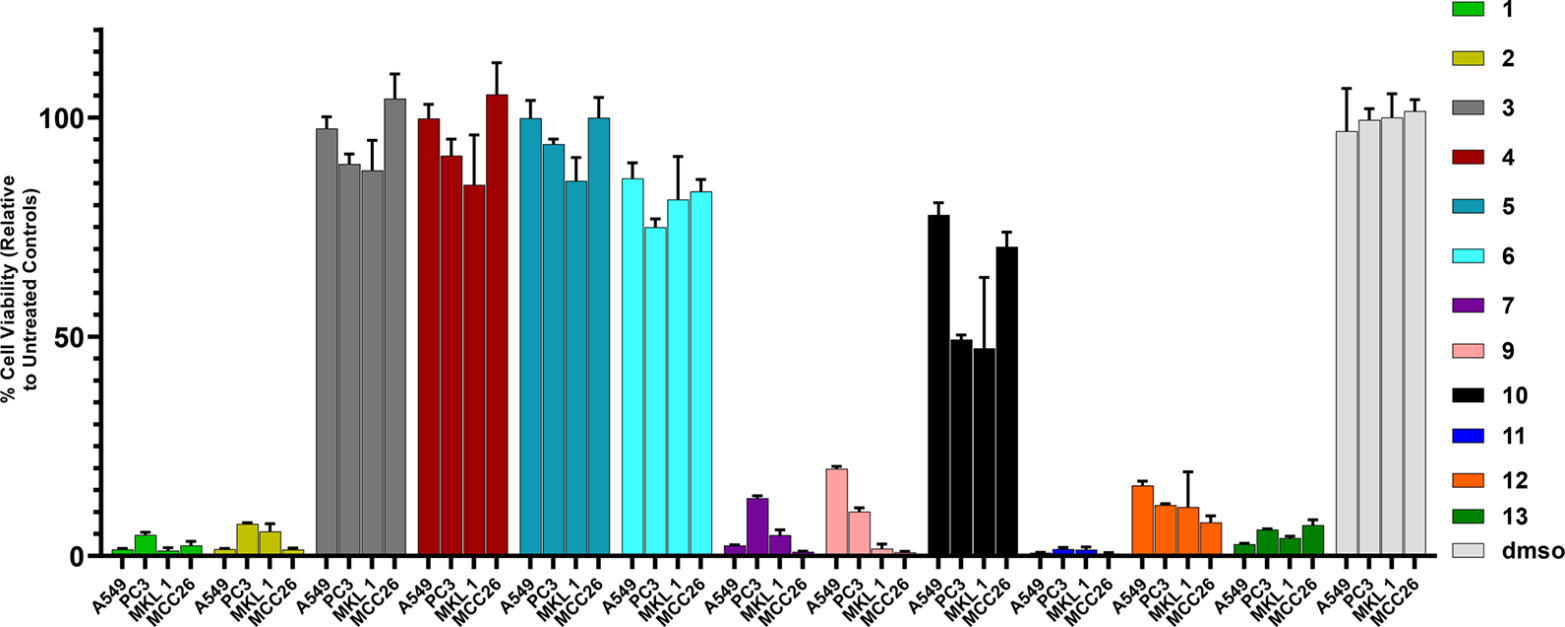
% Viability of A549 (non-small-cell lung) and PC3 (prostate) human cancer cell lines, and Merkel cells (MKL1 and MCC26) (after 72 h) at 80 μM concentration of compounds **1**–**7** and **9**–**13**.

Next, we assessed the antiproliferative activity of anthracyclinones **1**–**7** and compared the activity to anthracycline compounds previously isolated in our lab: nogalamycin A (**9**), nogalamycin KO (**10**), nogalamycin R (**11**), 3′,4′-demethoxy-nogalose-1-hydroxy-nogalamycinone (**12**), and aclacinomycin T (**13**).^33,62,63^ First, the compounds were assessed in a single dose (80 μM) viability assay against a representative set of human cancer cell lines [A549 (non-small-cell lung epithelial carcinoma), PC3 (prostate adenocarcinoma), and MKL1 (polyomavirus-positive Merkel carcinoma) and MCC26 (virus-negative Merkel carcinoma) (Figure 9)]. The most active compounds were anthracyclines nogalamycin A (**9**), nogalamycin R (**11**), 3′,4′-demethoxy-nogalose-1-hydroxy-nogalamycinone (**12**), and aclacinomycin T (**13**). This result was expected since the glycoside moiety of the anthracyclines is essential for binding to DNA and inhibition of DNA topoisomerases.^64^ Compounds **6** and **10** exhibited slight antiproliferative activity (**6**: 75–85% T/C; **10**: 50–75% T/C). In the single dose experiment **1**, **2**, and **7** exhibited antiproliferative activity (<10% T/C) comparable to previously reported compounds **9**, **11**, **12**, and **13**. Compounds **3**–**5** did not exhibit any activity in the assay.

The dose–response relationships of **1**, **2**, **6**, **7**, and **9**–**13** were established for A549 and PC3 cells and Merkel cells MKL1 and MCC26 (Supporting Information, Figure S83 and S84). The half-maximal inhibitory concentration values (IC_50_) were determined for the entire cancer cell panel (Table 3). Previously reported compound **9** was the most cytotoxic among the set tested, with IC_50_ values ranging from 21 to 95 nM. In contrast, compound **7** was the most active among the analogues derived from the current study, with IC_50_ values ranging from 8 to 26 μM. This is consistent with previous studies highlighting the contribution of anthracycline glycosylation to potency, selectivity, and ADMET.^65^

**Table 3:** Cytotoxic Activities of Compounds **1**–**13**^a^

	IC_50_ μM		IC_50_ μM	
compounds	A549	PC3	MKL1	MCC26
Nogalamycinone (**1**)	30.30 ± 2.30	18.60 ± 3.61	29.30 ± 10.30	36.50 ± 5.34
Auramycinone (**2**)	31.00 ± 0.96	13.90 ± 3.66	26.10 ± 1.16	30.40 ± 2.01
7-Deoxy-nogalamycinone (**3**)	>80	>80	>80	>80
7-Deoxyauramycinone (**4**)	>80	>80	>80	>80
9,10-*seco*-7-Deoxy-nogalamycinone (**5**)	>80	>80	>80	>80
4-β-d-Glucosyl-nogalamycinone (**6**)	>80	>80	>80	>80
Aklavinone (**7**)	17.80 ± 6.24	7.72 ± 0.57	22.70 ± 1.79	26.10 ± 0.66
Nogalamycin A (**9**)	0.056 ± 0.011	0.021 ± 0.002*	0.062 ± 0.035*	0.095 ± 0.044*
Nogalamycin KO (**10**)	>80	>80	>80	>80
Nogalamycin R (**11**)	32.30 ± 4.09	28.30 ± 3.10	18.50 ± 2.64	35.60 ± 2.73
3′,4′-Demethoxy-nogalose-1-hydroxy-nogalamycinone (**12**)	2.88 ± 0.23	2.88 ± 0.79	1.07 ± 0.011	3.50 ± 0.36
Aclacinomycin T (**13**)	3.62 ± 0.087	2.00 ± 0.15	2.35 ± 0.78	10.70 ± 4.32

a Cytotoxicity IC_50_ values were obtained after 72 h incubation. Actinomycin D and H_2_O_2_ [A549 (non-small-cell lung), PC3 (prostate) human cancer cell lines, and Merkel cells MKL1 and MCC26] were used as a positive control at 20 μM and 1 mM concentration, respectively (0% viable cells, *n* = 3, but *(*n* = 6)).

## Conclusions

The “mixing and matching” of PKS components for the reconstitution of non-native aromatic polyketide synthases has been the subject of numerous in vivo studies, which provided the foundational basis for the biosynthetic logic of these enzymes.^66−70^ However, these experiments often utilized PKS enzymes from disparate pathways, which resulted in recombinant PKS systems that produced low yields of the expected novel polyketides, or that failed.^71^ This resulted in the observation that the failure of polyketide combinatorial biosynthesis derives from the inflexibility of downstream enzymes to recognize novel substrates or lack of enzyme solubility.^72^ In contrast, focusing on enzymes from evolutionarily related pathways might provide more fertile ground for combinatorial biosynthesis efforts and improved substrate turnover to produce novel polyketides. More recently, the use of cell-free biosynthesis (also known as “combinatorial biosynthetic enzymology”) has demonstrated the success of this “mix-and-match” approach via the use of PKS enzymes from closely related pathways for the synthesis of defucogilvocarcin M and steffimycinone *in vitro*.^73,74^

A “Design-Build-Test-Learn” (DBTL) approach has been suggested to uncover the logic of recombinant PKS systems.^75^ This is the approach that we have currently undertaken with the development of the BIOPOLYMER toolbox, via the identification of PKS gene orthologs that interact positively and result in the production of expected polyketides in *S. coelicolor* M1152Δ*matAB*. In this work, the “design phase” resulted in the selection of different *S. coelicolor* hosts, strong promoters, vector combinations, and gene orthologs. The “build phase” was facilitated by the use of the BioBricks-[RFC 10] synthetic biology standard and straightforward transformation of *Streptomyces* spp. using intergeneric conjugation. The “test phase” carried out several different comparisons of anthracyclinone genes to identify those with the best conversion percentage and production yield of the expected polyketide. Lastly, the “learn phase” resulted in the identification of an optimal production host, *S. coelicolor* M1152Δ*matAB*, promoter and gene combinations, and the structure elucidation of several anthracyclinones (**1**–**4**) and new compounds 9,10-*seco*-7-deoxy-nogalamycinone (**5**) and 4-*O*-β-d-glucosyl-nogalamycinone (**6**). Furthermore, the production platform was used to generate newly engineered metabolites for testing in mammalian cancer cell viability assays. These assays confirmed that the 7-*O*-glycoside is important for the anticancer activity of anthracyclines, including nogalamycin A (**9**), 3′,4′-demethoxy-nogalose-1-hydroxy-nogalamycinone (**12**), and aclacinomycin T (**13**). Nevertheless, the observation that nogalamycinone, auramycinone, and aklavinone exhibited moderate cytotoxicity is encouraging for further efforts to generate diverse anthracycline analogues incorporating these pharmacophores.

The pathway engineering studies described here resulted in the unexpected generation of 7-deoxygenated metabolites, **3** and **4**, and two new anthracycline metabolites, 9,10-*seco*-7-deoxy-nogalamycinone (**5**) and 4-*O*-β-d-glucosyl-nogalamycinone (**6**). Despite being a genome minimalized “superhost”, the genome *S. coelicolor* M1152Δ*matAB* still encodes hypothetical proteins that can interact with heterologous pathways in an unanticipated fashion. Compounds **3**, **4**, and **5** are thought to derive from the dehydration of the 7-hydroxyl group via an ancillary CytA-like enzyme.^50^ CytA has been shown to catalyze the 7-reduction of a wide variety of anthracycline saccharide chains within the cytorhodin pathway. CytA could be part of a larger family of promiscuous enzymes encoded within actinomycete genomes, perhaps as an evolutionary self-defense strategy against xenobiotics. Studies are ongoing to identify this reductase within *S. coelicolor* M1152.

Compound **6** is thought to derive from a hypothetical glucosyltransferase that catalyzes the transfer of NDP-d-glucose to the 4-position of nogalamycinone. Studies are ongoing to identify the glucosyltransferase responsible for the transfer of NDP-d-glucose to nogalamycinone. The macrolide glucosyltransferase OleD has been mutagenized via directed evolution to afford one of the most substrate-promiscuous glycosyltransferase catalysts for the glycorandomization of a variety of diverse natural products.^76−79^ Notably, **6** has a similar 4-*O*-glycosylation pattern to the previously discovered mutactimycin PR, andicoquinones A–D, komodoquinone A, and histomodulin.^80^ In the cancer cell line cytotoxicity assays, the 4-β-d-glucose substitution was deleterious to the topoisomerase II inhibition of **6** since this compound was only slightly cytotoxic (Figure 9). Instead, the closely related 4-*O*-β-d-glucopyranuronosyl-ε-rhodomycinone (histomodulin) has been shown to exhibit upregulation of major histocompatibility class-I molecules on the surface of T-cells.^81^ This class of 4-*O*-glucosides could be further investigated for unique immunomodulatory activities as potential new anticancer or anti-infective agents.

In summation, BIOPOLYMER is a flexible synthetic biology platform for the rational programming of *S. coelicolor* to produce aromatic polyketide natural products. We anticipate that BIOPOLYMER will be useful for providing access to known and new anthracycline natural products for antiproliferative activity studies. We also expect that BIOPOLYMER could be useful for studying cryptic type II PKS pathways and for providing a robust toolset for unraveling their biosynthetic logic.

## Methods

### Bacterial Strains and Growth Conditions

*Escherichia coli* JM109 and *E. coli* ET12567 were grown in LB broth or LB agar at 37 °C as previously described.^82^*E. coli* JM109 was used for plasmid propagation and subcloning, while *E. coli* ET12567/pUZ8002 was used as the conjugation donor host for mobilizing expression vectors into *S. coelicolor* and *S. lividans* expression hosts as previously described.^83^ When appropriate, ampicillin (100 μg mL^–1^), kanamycin (25 μg mL^–1^), apramycin (25 μg mL^–1^), viomycin (25 μg mL^–1^), and nalidixic acid (30 μg mL^–1^) were supplemented to media to select for recombinant microorganisms.

*S. lividans* and *S. coelicolor* derivative strains were routinely maintained on Soya-Mannitol Flour (SFM) agar supplemented with 10 mM MgCl_2_ and International Streptomyces Project medium #4 (ISP4) (BD Difco) at 30 °C as described previously.^83^*S. lividans* K4–114 was a gift from Prof. Dr. Lou Charkoudian (Haverford College, PA). *S. coelicolor* M1146 and *S. coelicolor* M1152 were gifts from Prof. Dr. Mervyn Bibb’s laboratory (John Innes Centre, Norwich, UK). *S. coelicolor* M1152Δ*matAB* was generated via replacement of the *sco2963-sco2962 matAB* locus via PCR-ReDirect mutagenesis as previously described (Supporting Information, Method 1).^19^ For liquid culturing, *S. coelicolor* derivative strains were grown in TSB media (3 mL) to ferment the seed culture and then grown in a modified 50 mL SG-TES liquid medium (soytone 10 g, glucose 20 g, yeast extract 5 g, TES free acid 5.73 g, CoCl_2_ 1 mg, per liter) or 50 mL E1 medium for production for 7 days.^84^ All media and reagents were purchased from Thermo-Fisher Scientific.

### General Manipulations

Routine genetic cloning and plasmid manipulation were carried out in *E. coli* JM109 (New England Biolabs). *E. coli* ET12567/pUZ8002 was used as the host for intergeneric conjugation with *S. coelicolor* as previously described.^83^*E. coli* chemically competent cells were prepared using the Mix and Go! *E. coli* Transformation Kit (Zymo Research). *E. coli* was transformed with plasmid DNA via chemically competent heat-shock transformation as described previously. Plasmid DNA was isolated via the Wizard Plus SV Minipreps DNA Purification System by following the manufacturer’s protocols (Promega). All molecular biology reagents and enzymes used for plasmid construction were purchased from New England Biolabs. The conjugation donor host *E. coli* ET12567/pUZ8002 was transformed with constructs for mobilization into *S. coelicolor* strains, as previously described. For each transformation, 9–12 independent exconjugants were plated to DNA plates supplemented with antibiotics and grown for 4–5 days until the formation of vegetative mycelium.

### General Experimental Procedures

Ultraviolet–visible (UV–vis) spectra were taken directly from analytical HPLC runs and show relative intensities. All NMR spectra were recorded at 600 MHz (14.1 T) using a Bruker Avance Neo console equipped with a TCI 5 mm cryoprobe (all spectra were processed in Bruker Topspin 4.1.3 version, and 2D spectra were apodized with QSINE or SINE window functions and zero-filled to 256 × 1024 points), and spectra were analyzed and plotted using Mnova [where δ-values were referenced to respective solvent signals (CD_3_OD, δ_H_ 3.31 ppm, δ_C_ 49.15 ppm; CDCl_3_, δ_H_ 7.24 ppm, δ_C_ 77.23 ppm)] (Bruker BioSpin Corporation, Billerica, MA). High-resolution electrospray ionization (HRESI) mass spectra were recorded on AB SCIEX Triple TOF 5600 system. HPLC-UV/MS analyses were accomplished with an Agilent InfinityLab LC/MSD mass spectrometer (MS Model G6125B; Agilent Technologies, Santa Clara, CA, USA) equipped with an Agilent 1260 Infinity II Series Quaternary LC system and a Phenomenex NX-C18 column (250 × 4.6 mm, 5 μm) [Method: solvent A: H_2_O/0.1% formic acid, solvent B: CH_3_CN; flow rate: 0.5 mL min^–1^; 0–30 min, 5–100% B (linear gradient); 30–35 min, 100% B; 35–36 min, 100%–5% B; 36–40 min, 5% B]. HPLC-UV analyses were carried out in a Agilent 1260 system equipped with a photodiode array detector (PDA) and a Phenomenex C_18_ column (Phenomenex, Torrance, CA; 250 × 4.6 mm, 5 μm; solvent A: H_2_O/0.1% TFA, solvent B: CH_3_CN; flow rate: 1.0 mL min^–1^; 0–30 min, 5–100% B; 30–35 min, 100% B; 35–36 min, 100%–5% B; 36–40 min, 5% B). Semipreparative HPLC were carried out in a Agilent 1260 Infinity II (Prep HPLC) system equipped with a Diode Array Detector (DAD) and a Gemini 5 μm C_18_ 110 Å, LC column 250 × 10 mm (Phenomenex, Torrance, CA) [**Method A:** H_2_O/0.025% TFA; solvent B: CH_3_CN; flow rate: 5.0 mL min^–1^; 0–2 min, 15% B; 2–31 min, 15–100% B; 31–33 min, 100% B; 33–34 min, 100–15% B; 34–36 min, 10% B; **Method B:** solvent A: H_2_O/0.025% TFA; solvent B: CH_3_CN; flow rate: 5.0 mL min^–1^; 0–2 min, 15% B; 2–28 min, 15–75% B; 28–30 min, 75–100% B; 30–32 min, 100% B; 32–34 min, 100–15% B; 34–36 min, 10% B; **Method C:** solvent A: H_2_O/0.025% TFA; solvent B: CH_3_CN; flow rate: 5.0 mL min^–1^; 0–2 min, 25% B; 2–28 min, 25–85% B; 28–30 min, 85–100% B; 30–32 min, 100% B; 32–34 min, 100–25% B; 34–36 min, 25% B; **Method D:** solvent A: H_2_O/0.025% TFA; solvent B: CH_3_CN; flow rate: 5.0 mL min^–1^; 0–2 min, 10% B; 2–22 min, 10–65% B; 22–25 min, 65–100% B; 25–27 min, 100% B; 27–28 min, 100–10% B; 28–30 min, 10% B]. All solvents used were of ACS grade and purchased from Pharmco-AAPER (Brookfield, CT). Size exclusion chromatography was performed on Sephadex LH-20 (25–100 μm; GE Healthcare, Piscataway, NJ). A549 and PC3 cells were obtained from ATCC (Manassas, VA). All other reagents used were reagent grade and purchased from Sigma-Aldrich (Saint Louis, MO).

### Statistical Analyses

The statistical significance of the impact of genetic manipulations and combinatorially assessed variables on production was assessed via post hoc analysis. One-way ANOVA, two-way ANOVA, and Student’s *t* test analyses were performed using GraphPad Prism version 9.4.1 for Mac OS X, GraphPad Software, San Diego, CA, USA, www.graphpad.com.

### Cancer Cell Line Viability Assay

A549 and PC3 cells were obtained from ATCC (Manassas, VA). Merkel cells MKL1 and MCC26 were a gift from Dr. Isaac Brownell’s laboratory (NIH/NIAMS, Bethesda, MD). All other reagents used were reagent grade and purchased from Sigma-Aldrich (Saint Louis, MO). Human cell line cytotoxicity [A549 (non-small-cell lung) and PC3 (prostate) human cancer cell lines, Merkel cells MKL1 and MCC26] assays were accomplished in triplicate following our previously reported protocols.^85−88^ Vehicle (DMSO) was used as the negative control, and actinomycin D and H_2_O_2_ (A549 and PC3; MKL1 and MCC26) were used as positive control at 20 μM and 1 mM concentration, respectively.

### Isolation of Compounds **1**–**6**

*S. coelicolor* M1152Δ*matAB*::pRW10000 was grown on a soya mannitol flour (SFM) agar plate until well-sporulated for 4–5 days. Spores from the SFM agar plate were used to inoculate a seed culture of 50 mL tryptic soy broth in a 250 mL Erlenmeyer shake flask and were grown for 48 h in an orbital shaker at 30 °C at 220 rpm. One milliliter of seed culture was used to inoculate each of the 40 × 100 mL SG liquid media shake flasks (1% v/v inoculum) which were fermented for 5 days. The 4 L culture was extracted 4 × 4 L of ethyl acetate +0.1% formic acid and was dried in vacuo on a rotary evaporator. The resulting 2.3 g crude extract was dissolved in 9:1 chloroform–methanol and was loaded onto a 25 g SiO_2_ solid load cartridge and chromatographed on a 24 g RediSep Gold Normal Phase Silica cartridge (Teledyne-ISCO). The extract was fractionated using the gradient setting and chloroform–methanol systems [**Method E:** chloroform; solvent B: methanol; flow rate: 40.0 mL min^–1^; 0–15 min, 0–10% B.] Compounds **1**–**5** eluted in combined fraction at a retention time of 3.0 min. The compounds were individually resolved and purified via preparatory HPLC.

The same strain was separately grown in 2 L of E1 media to produce substantive amounts of compound 6. The 2 L fermentation was centrifuged to separate the cell pellet from the fermentation broth. The fermentation broth was extracted with 40 g Amberlite XAD-7 resin for 16 h, washed with 2 L of water, eluted with 1 L of methanol, and dried in vacuo on a rotary evaporator. Compound **6** was isolated via preparatory HPLC.

### Physicochemical Properties of Compounds **1**–**6**

#### Nogalamycinone (**1**)

C_21_H_18_O_8_ (398); yellow solid; *HPLC*-*R*_t_ = 22.72 min (Supporting Information, Figures S18 – S28); UV–vis λ_max_ 198, 228, 258, 290 (sh), 430 nm; ^1^H NMR (CDCl_3_, 600 MHz) and ^13^C NMR (CDCl_3_, 150 MHz), see Tables S3 and S4; (−)**-**ESI-MS: *m*/*z* 397 [M – H]^−^; (+)-ESI-MS: *m*/*z* 381 [(M-H_2_O) + H]^+^, 363 [(M-2H_2_O) + H]^+^; (−)**-**HRESI-MS: *m*/*z* 397.0929 [M – H]^−^ (calcd for C_21_H_17_O_8_, 397.0929); (+)**-**HRESI-MS: *m*/*z* 363.0860 [(M-2H_2_O) + H]^+^ (calcd. for C_21_H_15_O_6_, 363.0863), 421.0865 [M + Na]^+^ (calcd for C_21_H_18_O_8_Na, 421.0893), 819.1865 [2 M + Na]^+^ (calcd for C_42_H_36_O_16_Na, 819.1895).

#### Auramycinone (9-*epi*-Nogalamycinone) (**2**)

C_21_H_18_O_8_ (398); yellow solid; *HPLC*-*R*_t_ = 22.68 min (Supporting Information, Figures S29 – S39); UV–vis λ_max_ 196, 228, 260, 290 (sh), 430 nm; ^1^H NMR (CDCl_3_, 600 MHz) and ^13^C NMR (CDCl_3_, 150 MHz), see Tables S3 and S4; (−)**-**ESI-MS: *m*/*z* 397 [M – H]^−^; (+)-ESI-MS: *m*/*z* 381 [(M-H_2_O) + H]^+^, 363 [(M-2H_2_O) + H]^+^; (−)**-**HRESI-MS: *m*/*z* 397.0930 [M – H]^−^ (calcd for C_21_H_17_O_8_, 397.0929); (+)**-**HRESI-MS: *m*/*z* 363.0863 [(M-2H_2_O) + H]^+^ (calcd for C_21_H_15_O_6_, 363.0863), 421.0890 [M + Na]^+^ (calcd for C_21_H_18_O_8_Na, 421.0893), 819.1913 [2 M + Na]^+^ (calcd for C_42_H_36_O_16_Na, 819.1895).

#### 7-Deoxy-nogalamycinone (**3**)

C_21_H_18_O_7_ (382); yellow solid; *HPLC*-*R*_t_ = 28.35 min (Supporting Information, Figures S40 – S50); UV–vis λ_max_ 200, 228, 260, 290 (sh), 432 nm; ^1^H NMR (CDCl_3_, 600 MHz) and ^13^C NMR (CDCl_3_, 150 MHz), see Tables S3 and S4; (−)**-**ESI-MS: *m*/*z* 381 [M – H]^−^; (+)-ESI-MS: *m*/*z* 383 [M + H]^+^, 365 [(M-H_2_O) + H]^+^; (−)**-**HRESI-MS: *m*/*z* 381.0971 [M – H]^−^ (calcd for C_21_H_17_O_7_, 381.0979); (+)**-**HRESI-MS: *m*/*z* 365.1241 [(M-H_2_O) + H]^+^ (calcd for C_21_H_17_O_6_, 365.1019), 383.1355 [M + H]^+^ (calcd for C_21_H_19_O_7_, 383.1126), 405.1187 [M + Na]^+^ (calcd for C_21_H_18_O_7_Na, 405.09447), 787.2493 [2 M + Na]^+^ (calcd for C_42_H_36_O_14_Na, 787.1997).

#### 7-Deoxyauramycinone (9-*epi*-7-deoxy-nogalamycinone) (**4**)

C_21_H_18_O_7_ (382); yellow solid; *HPLC*-*R*_t_ = 28.02 min (Supporting Information, Figures S51 – S61); UV–vis λ_max_ 200, 228, 260, 290 (sh), 432 nm; ^1^H NMR (CDCl_3_, 600 MHz) and ^13^C NMR (CDCl_3_, 150 MHz), see Tables S3 and S4; (−)**-**ESI-MS: *m*/*z* 381 [M – H]^−^; (+)-ESI-MS: *m*/*z* 405 [M + Na]^+^, 383 [M + H]^+^, 365 [(M-H_2_O) + H]^+^; (−)**-**HRESI-MS: *m*/*z* 381.0971 [M – H]^−^ (calcd for C_21_H_17_O_7_, 381.0979); (+)**-**HRESI-MS: *m*/*z* 383.1188 [M + H]^+^ (calcd for C_21_H_19_O_7_, 383.1126).

#### 9,10-*seco*-7-Deoxy-nogalamycinone (**5**)

C_21_H_20_O_7_ (384); yellow solid; *HPLC*-*R*_t_ = 27.95 min (Supporting Information, Figures S62 – S72); UV–vis λ_max_ 200, 228, 260, 290 (sh), 434 nm; ^1^H NMR (CDCl_3_, 600 MHz) and ^13^C NMR (CDCl_3_, 150 MHz), see Tables S3 and S4; (−)**-**ESI-MS: *m*/*z* 383 [M – H]^−^; (+)-ESI-MS: *m*/*z* 367 [(M-H_2_O) + H]^+^; (−)**-**HRESI-MS: *m*/*z* 383.1129 [M – H]^−^ (calcd for C_21_H_19_O_7_, 383.1136); (+)**-**HRESI-MS: *m*/*z* 367.1066 [(M-H_2_O) + H]^+^ (calcd for C_21_H_19_O_6_, 367.1176), 407.0976 [M + Na]^+^ (calcd for C_21_H_20_O_7_Na, 407.1101), 791.2076 [2 M + Na]^+^ (calcd for C_42_H_40_O_14_Na, 791.2310).

#### 4-β-d-Glucosyl-nogalamycinone (**6**)

C_27_H_28_O_13_ (560); yellow solid; *HPLC*-*R*_t_ = 15.54 min (Supporting Information, Figures S73 – S82); UV–vis λ_max_ 196, 226, 260, 286 (sh), 412 nm; ^1^H NMR (CD_3_OD, 600 MHz) and ^13^C NMR (CD_3_OD, 150 MHz), see Table S5; (−)**-**ESI-MS: *m*/*z* 559 [M – H]^−^; (+)-ESI-MS: *m*/*z* 363 [(M-glucose-2H_2_O) + H]^+^; (−)**-**HRESI-MS: *m*/*z* 559.1459 [M – H]^−^ (calcd for C_27_H_27_O_13_, 559.1457); (+)**-**HRESI-MS: *m*/*z* 363.0860 [(M-glucose-2H_2_O) + H]^+^ (calcd for C_21_H_15_O_6_, 363.0863).

## Supporting Information Available

Experimental procedure for generation of *S. coelicolor* M1152Δ*matAB* strain, plasmid maps for vectors, sequences of BioBricks parts and coding sequences, high-resolution mass spectrometry data, NMR tables, NMR spectra, HPLC-UV–vis chromatograms, and dose–response data for human cancer cell lines (PDF)
