# Supplementary material for: A BioBricks Metabolic Engineering Platform for the Biosynthesis of Anthracyclinones in Streptomyces coelicolor
Source: ACS Synth Biol. 2022 Nov 15;11(12):4193–209. doi: 10.1021/acssynbio.2c00498 (PMC9764417; doi:10.1021/acssynbio.2c00498)
Supplement: Supplementary file 1 — sb2c00498_si_001.pdf [file sb2c00498_si_001.pdf]

A BioBricks® metabolic engineering platform for the biosynthesis of anthracyclinones in *Streptomyces coelicolor*

Rongbin Wang [†,1], Jennifer Nguyen [†,2], Jacob Hecht [2], Nora Schwartz [2], Katelyn V. Brown [2], Larissa V. Ponomareva [3,4], Magdalena Niemczura [1], Dino van Dissel [5], Gilles van Wezel [5], Jon S. Thorson [3,4], Mikko Metsä-Ketelä [*,1], Khaled A. Shaaban [*,3,4], and S. Eric Nybo [*,2]

[1] Department of Life Technologies, University of Turku, FIN-20014 Turku, [2] Department of Pharmaceutical Sciences, College of Pharmacy, Ferris State University, Big Rapids, MI 49307, [3] Center for Pharmaceutical Research and Innovation, [4] Department of Pharmaceutical Sciences, College of Pharmacy, University of Kentucky, Lexington, Kentucky 40536, United States, [5] Institute of Biology, Leiden University, Sylviusweg 72, 2333 BE Leiden, The Netherlands

† These authors contributed equally to this work.

ORCID

Larissa V. Ponomareva: https://orcid.org/0000-0003-2182-8774
Jon S. Thorson: https://orcid.org/0000-0002-7148-0721
Khaled A. Shaaban: https://orcid.org/0000-0001-7638-4942
Mikko Metsä-Ketelä: https://orcid.org/0000-0003-3176-2908
S. Eric Nybo: https://orcid.org/0000-0001-7884-7787

† Email: EricNybo@Ferris.edu

Table of Contents

Method 1 Generation of the *Streptomyces coelicolor* M1152Δ*matAB* mutant..................7

Table S1. BioBricks® RFC[10] gene coding sequences (5’ → 3’) used in this study. 8

Table S2. Synthetic BioBrick RFC[10] promoter and terminator parts (5’ → 3’) used in this study...27

Figure S1. Overview of BIOPOLYMER metabolic engineering platform strategy 28

Figure S2. Plasmid maps for *snoa123* minPKS constructs. 29

Figure S3. Plasmid maps for *snoa123* minPKS (green) and KR/ARO/CYC/OXY constructs (red). 30

Figure S4. Plasmid maps for *oxyABCD* minPKS constructs. Green gene arrows correspond to wildtype sequences, and blue gene arrows correspond to codon-optimized sequences. 31

Figure S5. Plasmid maps for *dpsABGCD* minPKS constructs. Green gene arrows correspond to wildtype sequences, and blue gene arrows correspond to codon-optimized sequences. 32

Figure S7. Plasmid maps for *aknBCDE2F* minPKS constructs. 34

Figure S8. Plasmid maps for aknBCDE2F minPKS (green) and KR/ARO/CYC/OXY constructs (red). 35

Figure S9. Plasmid maps for MT/CYC/KR plasmids. 36

Figure S10. Shunt products detected from nogalonic acid engineering experiments. ...37

Figure S11. High-resolution ESI-MS/MS mass spectrum of SEK15. 38

Figure S12. High-resolution ESI-MS/MS mass spectrum of SEK15b. 39

Figure S13. ESI-MS positive mode mass spectrum of UWM7 $[M + H]^+$ = 399 *m/z*. 40

Figure S14. ESI-MS positive mode mass spectrum of WJ85 $[M + H]^+$ = 368 *m/z*. 41

Figure S15: Chemical structures of compounds 1-6. 42

Figure S16: ^{1}H,^{1}H-COSY (▬), selected HMBC (→) and NOESY (↶↷) correlations of compounds 1-5. 42

Figure S17: Selected 2D-NMR homonuclear and heteronuclear correlations for compound 6. 43

Table S3. ^{13}C (150 MHz) NMR Spectroscopic Data of Compounds 1-5 in $CDCl_3$ (δ in ppm)..44

Table S4. ^{1}H (600 MHz) NMR Spectroscopic Data of Compounds 1-5 in $CDCl_3$ (δ in ppm)..45

Table S5. ^{13}C (150 MHz) and ^{1}H (600 MHz) NMR Spectroscopic Data of 4-β-D-glucosyl-nogalamycinone (6) in CD_3OD (δ in ppm). 46

Figure S18. HPLC-UV/vis analysis of nogalamycinone (1). 47

Figure S19. HPLC-MS analysis of nogalamycinone (1). 48
Figure S20. (–)-HRESI-MS spectrum of nogalamycinone (1)....49
Figure S21. (+)-HRESI-MS spectrum of nogalamycinone (1). 50
Figure S22. ^{1}H NMR spectrum ($CDCl_3$, 600 MHz) of nogalamycinone (1). 51
Figure S23. ^{13}C NMR spectrum ($CDCl_3$, 150 MHz) of nogalamycinone (1)....52
Figure S24. ^{1}H ($CDCl_3$, 600 MHz) and ^{13}C ($CDCl_3$, 150 MHz) NMR spectra of nogalamycinone (1). 53
Figure S25. ^{1}H,^{1}H-COSY spectrum ($CDCl_3$, 600 MHz) of nogalamycinone (1). 54
Figure S26. HSQC spectrum ($CDCl_3$, 600 MHz) of nogalamycinone (1)....55
Figure S27. HMBC spectrum ($CDCl_3$, 600 MHz) of nogalamycinone (1). 56
Figure S28. NOESY spectrum ($CDCl_3$, 600 MHz) of nogalamycinone (1). 57
Figure S29: HPLC-UV/vis analysis of auramycinone (9-epi-nogalamycinone; 2). 58
Figure S30: HPLC-MS analysis of auramycinone (9-epi-nogalamycinone; 2). 59
Figure S31. (–)-HRESI-MS spectrum of auramycinone (9-epi-nogalamycinone; 2). 60
Figure S32. (+)-HRESI-MS spectrum of auramycinone (9-epi-nogalamycinone; 61
Figure S33. ^{1}H NMR spectrum ($CDCl_3$, 600 MHz) of auramycinone (9-epi-nogalamycinone; 2). 62
Figure S34. ^{13}C NMR spectrum ($CDCl_3$, 150 MHz) of auramycinone (9-epi-nogalamycinone; 2). 63
Figure S36. ^{1}H,^{1}H-COSY spectrum ($CDCl_3$, 600 MHz) of auramycinone (9-epi-nogalamycinone; 2). 65
Figure S37. HSQC spectrum ($CDCl_3$, 600 MHz) of auramycinone (9-epi-nogalamycinone; 2). 66
Figure S38. HMBC spectrum ($CDCl_3$, 600 MHz) of auramycinone (9-epi-nogalamycinone; 2). 67
Figure S39. NOESY spectrum ($CDCl_3$, 600 MHz) of auramycinone (9-epi-Nogalamycinone; 2). 68
Figure S40: HPLC-UV/vis analysis of 7-deoxy-nogalamycinone (3). 69
Figure S41: HPLC-MS analysis of 7-deoxy-nogalamycinone (3). 70
Figure S42. (–)-HRESI-MS spectrum of 7-deoxy-nogalamycinone (3). 71
Figure S43. (+)-HRESI-MS spectrum of 7-deoxy-nogalamycinone (3). 72
Figure S44. ^{1}H NMR spectrum ($CDCl_3$, 600 MHz) of 7-deoxy-nogalamycinone (3). 73
Figure S45. ^{13}C NMR spectrum ($CDCl_3$, 150 MHz) of 7-deoxy-nogalamycinone (3). 74
Figure S46. ^{1}H (CDCl3, 600 MHz) and ^{13}C ($CDCl_3$, 150 MHz) NMR spectra of 7-deoxy-nogalamycinone (3). 75

Figure S47. $^1H,^1H$-COSY spectrum ($CDCl_3$, 600 MHz) of 7-deoxy-nogalamycinone (3). 76

Figure S48. HSQC spectrum ($CDCl_3$, 600 MHz) of 7-deoxy-nogalamycinone (3)..........77

Figure S49. HMBC spectrum ($CDCl_3$, 600 MHz) of 7-deoxy-nogalamycinone (3). 78

Figure S50. NOESY spectrum ($CDCl_3$, 600 MHz) of 7-deoxy-nogalamycinone (3). 79

Figure S51: HPLC-UV/vis analysis of 7-deoxyauramycinone (9-epi-7-deoxy-nogalamycinone; 4). 80

Figure S52: HPLC-MS analysis of 7-deoxyauramycinone (9-epi-7-deoxy-nogalamycinone; 4). 81

Figure S53. (–)-HRESI-MS spectrum of 7-deoxyauramycinone (9-epi-7-deoxy-nogalamycinone; 4). 82

Figure S54. (+)-HRESI-MS spectrum of 7-deoxyauramycinone (9-epi-7-deoxy-nogalamycinone; 4). 83

Figure S55. ^1H NMR spectrum ($CDCl_3$, 600 MHz) of 7-deoxyauramycinone (9-epi-7-deoxy-nogalamycinone; 4)....................84

Figure S56. ^{13}C NMR spectrum ($CDCl_3$, 150 MHz) of 7-deoxyauramycinone (9-epi-7-deoxy-nogalamycinone; 4)....................85

Figure S57. ^1H ($CDCl_3$, 600 MHz) and ^{13}C ($CDCl_3$, 150 MHz) NMR spectra of 7-deoxyauramycinone (9-epi-7-deoxy-nogalamycinone; 4)....................86

Figure S58. $^1H,^1H$-COSY spectrum ($CDCl_3$, 600 MHz) of 7-deoxyauramycinone (9-epi-7-deoxy-nogalamycinone; 4). 87

Figure S59. HSQC spectrum ($CDCl_3$, 600 MHz) of 7-deoxyauramycinone (9-epi-7-deoxy-nogalamycinone; 4)....................88

Figure S60. HMBC spectrum ($CDCl_3$, 600 MHz) of 7-deoxyauramycinone (9-epi-7-deoxy-nogalamycinone; 4)....................89

Figure S61. NOESY spectrum ($CDCl_3$, 600 MHz) of 7-deoxyauramycinone (9-epi-7-deoxy-nogalamycinone; 4)....................90

Figure S62: HPLC-UV/vis analysis of 9,10-seco-7-deoxy-nogalamycinone (5). HPLC-conditions: solvent A: H2O/0.1% FA; solvent B: CH3CN; flow rate: 0.5 mL min-1; 0-30 min, 5-100% B; 30-35 min, 100% B; 35-36 min, 100-5% B; 36-40 min, 5% B; Phenomenex NX-C18 column (250 × 4.6 mm, 5 µm); 254 nm, 280 nm, 400 nm. UV-vis inset of full wavelength scan (190-600 nm). 91

Figure S63: HPLC-MS analysis of 9,10-seco-7-deoxy-nogalamycinone (5). 92

Figure S64. (–)-HRESI-MS spectrum of 9,10-seco-7-deoxy-nogalamycinone (5). 93

Figure S65. (+)-HRESI-MS spectrum of 9,10-seco-7-deoxy-nogalamycinone (5). 94

Figure S66. ^1H NMR spectrum ($CDCl_3$, 600 MHz) of 9,10-seco-7-deoxy-nogalamycinone (5). 95

Figure S67. ^{13}C NMR spectrum ($CDCl_3$, 150 MHz) of 9,10-seco-7-deoxy-nogalamycinone (5). 96

Figure S68. ^{1}H ($CDCl_3$, 600 MHz) and ^{13}C ($CDCl_3$, 150 MHz) NMR spectra of 9,10-seco-7-deoxy-nogalamycinone (5). 97

Figure S69. ^{1}H,^{1}H-COSY spectrum ($CDCl_3$, 600 MHz) of 9,10-seco-7-deoxy-nogalamycinone (5). 98

Figure S70. HSQC spectrum ($CDCl_3$, 600 MHz) of 9,10-seco-7-deoxy-nogalamycinone (5). 99

Figure S71. HMBC spectrum ($CDCl_3$, 600 MHz) of 9,10-seco-7-deoxy-nogalamycinone (5). 100

Figure S72. NOESY spectrum ($CDCl_3$, 600 MHz) of 9,10-seco-7-deoxy-nogalamycinone (5). 101

Figure S73: HPLC-MS analysis of 4-β-D-glucosyl-nogalamycinone (6). 102

Figure S74. (–)-HRESI-MS spectrum of 4-β-D-glucosyl-nogalamycinone (6). 103

Figure S75. (+)-HRESI-MS spectrum of 4-β-D-glucosyl-nogalamycinone (6). 104

Figure S76. ^{1}H NMR spectrum (CD_3OD, 600 MHz) of 4-β-D-glucosyl-nogalamycinone (6). 105

Figure S77. ^{13}C NMR spectrum (CD_3OD, 150 MHz) of 4-β-D-glucosyl-nogalamycinone (6). 106

Figure S78. ^{1}H,^{1}H-COSY spectrum (CD_3OD, 600 MHz) of 4-β-D-glucosyl-nogalamycinone (6). 107

Figure S79. HSQC spectrum (CD_3OD, 600 MHz) of 4-β-D-glucosyl-nogalamycinone (6). 108

Figure S80. HMBC spectrum (CD_3OD, 600 MHz) of 4-β-D-glucosyl-nogalamycinone (6). 109

Figure S81. NOESY spectrum (CD_3OD, 600 MHz) of 4-β-D-glucosyl-nogalamycinone (6). 110

Figure S82. TOCSY spectrum (CD_3OD, 600 MHz) of 4-β-D-glucosyl-nogalamycinone (6). 111

Figure S83: A) Dose-response of compounds 1, 2, 6, 7 and 9-13 against A549 (non-small cell lung) human cancer cell line (72 h). B) Dose-response of compounds 1, 2, 6, 7 and 9-13 against PC3 (prostate) human cancer cell line (72 h). 112

Figure S84: Dose-response of compounds 1, 2, 6, 7 and 9-13 against Merkel cells (MKL1 and MCC26), (72 h). 113

Figure S85: ESI-MS negative mode mass spectrum of aklanonic acid shunt products AA-1, AA-2, and AA-3 from *S. coelicolor* M1152Δ*matAB*::pSET-A2A5. 115

Figure S86: HPLC-UV/vis analysis of aklavinone standard. 116

Figure S87. ESI-MS negative mode mass spectrum of aklavinone standard $[M - H]^-$ = 411 *m/z*. 117

Figure S88: HPLC-UV/vis analysis of aklavinone produced from *S. coelicolor* M1152Δ*matAB*::pSET-A2A5::pTG-A6. 118

Figure S89. ESI-MS negative mode mass spectrum of aklavinone produced from *S. coelicolor* M1152Δ*matAB*::pSET-A2A5::pTG-A6. $[M - H]^-$ = 411 *m/z*. 119

Figure S90. HPLC-UV/vis analysis of 9-*epi*-aklavinone produced from *S. coelicolor* M1152Δ*matAB*::pSET-A2A5::pTG-S7. 120

Figure S91. ESI-MS negative mode mass spectrum of 9-*epi*-aklavinone produced from S. coelicolor M1152ΔmatAB::pSET-A2A5::pTG-S6. $[M - H]^-$ = 411 *m/z*. 121

Figure S92. ESI-MS negative mode mass spectrum of auramycinone produced from S. coelicolor M1152ΔmatAB::pSET-S2S5::pTG-A6. $[M - H]^-$ = 397 *m/z.* 122

Figure S93 ESI-MS positive mode mass spectrum of nogalamycinone produced from S. coelicolor M1152ΔmatAB::pRW10000. $[M + Na]^+$ = 421 *m/z*. 123

Method 1 Generation of the *Streptomyces coelicolor* M1152Δ*matAB* mutant

In-frame deletion mutants for SCO2963/SCO2962 in *S. coelicolor* M1152 were created as described earlier [15]. In brief, the upstream region of SCO2963 ranging from −1326 to +43 relative to the start codon and the downstream region of SCO2962 from +2190 to +3610 were amplified by PCR from the *S. coelicolor* genome using the primers listed below. The amplified flanks were cloned into the unstable shuttle vector pWHM3-oriT using the *EcoRI* and *HindIII* restriction sites [16]. The *Xba*I site, featured in both amplified regions, was used for insertion of the apramycin resistance cassette *aacC4* flanked by *loxP* sites between the flanking regions. The completed vector was introduced into *E. coli* ET12567 + PUZ8002, which allowed its transfer into *S. coelicolor* M1152 by conjugation. Mutants where the *matAB* locus was replaced by the aacC4 cassette and where the pWHM3 vector was lost were isolated by replicate plating for a Thio- / Apra+ phenotype. A marker free *S. coelicolor* M1152 Δ*matAB* strain was obtained by introduction of the pUWLcre plasmid, expressing the Cre recombinase, which incised the *loxP* sites surrounding the apramycin resistance gene.

matA_-1326	AGTCGAATTCCAGCCGGGCGGTGAGATTCC
matA_+43	ACTGTCTAGACGAGCACTCGTCGGCCGAAC
matB_+2190	AGTCTCTAGAAGGCCGGTCGGATGACCACC
matB_+3610	AGTCAAGCTTCCCTGTTCACTCCCGCAACCG

Table S1. BioBricks® RFC[10] gene coding sequences (5' → 3') used in this study.

pSET152BB (expression vector)

atctacgtctgtcgagaagtttctgatcgaaaagttcgacagcgtctccgacctgatgcagctctcgcagggcgaagaatctcgtgctttcagcttcgat
gtaggagggcgtggatatgtcctgcgggtaaatagctgcgccgatggtttctacaaagatcgttatgttgatcggcactttgcatcggccgcgctcccg
attccggaagtgcttgacattggggaatttatgcggtgtgaaataccgcacagatgcgtaaggagaaaataccgcatcaggcgccattcgccattca
ggctgcgcaactgttgggaagggcgatcggtgcgggcctcttcgctattacgccagctggcgaaagggggatgtgctgcaaggcgattaagttggg
taacgccagggttttcccagtcacgacgttgtaaaacgacggccagtgccaagcttgggctgcaggtcgactctagaggatccgcggccgcgcgc
gatatcgaattcgtaatcatgtcatagctgtttcctgtgtgaaattgttatccgctcacaattccacacaacatacgagccggaagcataaagtgtaaag
cctggggtgcctaatgagtgagctaactcacattaattgcgttgcgctcactgcccgctttccagtcgggaaacctgtcgtgccagctgcattaatgaat
cggccaacgcgcggggagaggcggtttgcgtattgggcgctcttccgcttcctcgctcactgactcgctgcgctcggtcgttcggctgcggcgagcg
gtatcagctcactcaaaggcggtaatacggttatccacagaatcaggggataacgcaggaaagaacatgtgagcaaaaggccagcaaaaggc
caggaaccgtaaaaaggccgcgttgctggcgtttttccataggctccgcccccctgacgagcatcacaaaaatcgacgctcaagtcagaggtggc
gaaacccgacaggactataaagataccaggcgtttccccctggaagctccctcgtgcgctctcctgttccgaccctgccgcttaccggatacctgtcc
gcctttctcccttcgggaagcgtggcgctttctcatagctcacgctgtaggtatctcagttcggtgtaggtcgttcgctccaagctgggctgtgtgcacga
accccccgttcagcccgaccgctgcgccttatccggtaactatcgtcttgagtccaacccggtaagacacgacttatcgccactggcagcagccact
ggtaacaggattagcagagcgaggtatgtaggcggtgctacagagttcttgaagtggtggcctaactacggctacactagaagaacagtatttggta
tctgcgctctgctgaagccagttaccttcggaaaaagagttggtagctcttgatccggcaaacaaaccaccgctggtagcggtggtttttttgtttgcaa
gcagcagattacgcgcagaaaaaaaggatctcaagaagatcctttgatcttttctacggggtctgacgctcagtggaacgaaaactcacgttaagg
gattttggtcatgagattatcaaaaaggatcttcacctagatccttttggttcatgtgcagctccatcagcaaaaggggatgataagtttatcaccaccga
ctatttgcaacagtgccgttgatcgtgctatgatcgactgatgtcatcagcggtggagtgcaatgtcgtgcaatacgaatggcgaaaagccgagctca
tcggtcagcttctcaaccttggggttacccccggcggtgtgctgctggtccacagctccttccgtagcgtccggcccctcgaagatgggccacttggac
tgatcgaggccctgcgtgctgcgctgggtccgggagggacgctcgtcatgccctcgtggtcaggtctggacgacgagccgttcgatcctgccacgtc
gcccgttacaccggaccttggagttgtctctgacacattctggcgcctgccaaatgtaaagcgcagcgcccatccatttgcctttgcggcagcggggc
cacaggcagagcagatcatctctgatccattgcccctgccacctcactcgcctgcaagcccggtcgcccgtgtccatgaactcgatgggcaggtact
tctcctcggcgtgggacacgatgccaacacgacgctgcatcttgccgagttgatggcaaaggttccctatggggtgccgagacactgcaccattcttc
aggatggcaagttggtacgcgtcgattatctcgagaatgaccactgctgtgagcgctttgccttggcggacaggtggctcaaggagaagagccttca
gaaggaaggtccagtcggtcatgcctttgctcggttgatccgctcccgcgacattgtggcgacagccctgggtcaactgggccgagatccgttgatct
tcctgcatccgccagaggcgggatgcgaagaatgcgatgccgctcgccagtcgattggctgagctcatgagcggagaacgagatgacgttggag
gggcaaggtcgcgctgattgctggggcaacacgtggagcggatcggggattgtctttcttcagctcgctgatgatatgctgacgctcaatgccgtttgg
cctccgactaacgaaaatcccgcatttggacggctgatccgattggcacggcggacggcgaatggcggagcagacgctcgtccgggggcaatg
agatatgaaaaagcctgaactcaccgcgacgtatcgggccctggccagctagctagagtcgacctccaggtccccggggatcggtcttgccttgct
cgtcggtgatgtacttcaccagctccgcgaagtcgctcttcttgatggagcgcatggggacgtgcttggcaatcacgcgcaccccccggccgttttag
cggctaaaaaagtcatggctctgccctcgggcggaccacgcccatcatgaccttgccaagctcgtcctgcttctcttcgatcttcgccagcagggcga
ggatcgtggcatcaccgaaccgcgccgtgcgcgggtcgtcggtgagccagagtttcagcaggccgcccaggcggcccaggtcgccattgatgcg
ggccagctcgcggacgtgctcatagtccacgacgcccgtgattttgtagccctggccgacggccagcaggtaggccgacaggctcatgccggcc
gccgccgccttttcctcaatcgctcttcgttcgtctggaaggcagtacaccttgataggtgggctgcccttcctggttggcttggtttcatcagccatccgct
tgccctcatctgttacgccggcggtagccggccagcctcgcagagcaggattcccgttgagcaccgccaggtgcgaataagggacagtgaagaa
ggaacacccgctcgcgggtgggcctacttcacctatcctgcccggctgacgccgttggatacaccaaggaaagtctacacgaaccctttggcaaa
atcctgtatatcgtgcgaaaaaggatggatataccgaaaaaatcgctataatgaccccgaagcagggttatgcagcggaaaagatccgtcgacct
ccaggcatgcaagctctagcgattccagacgtcccgaaggcgtggcgcggcttccccgtgccggagcaatcgccctgggtgggttacacgacgc
ccctctatggcccgtactgacggacacaccgaagccccggcggcaaccctcagcggatgccccggggcttcacgttttcccaggtcagaagcggt
tttcgggagtagtgccccaactggggtaacctttgagttctctcagttgggggcgtagggtcgccgacatgacacaaggggttgtgaccggggtgga
cacgtacgcgggtgcttacgaccgtcagtcgcgcgagcgcgaaaattcgagcgcagcaagcccagcgacacagcgtagcgccaacgaagac
aaggcggccgaccttcagcgcgaagtcgagcgcgacgggggccggttcaggttcgtcgggcatttcagcgaagcgccgggcacgtcggcgttc
gggacggcggagcgcccggagttcgaacgcatcctgaacgaatgccgcgccgggcggctcaacatgatcattgtctatgacgtgtcgcgcttctcg
cgcctgaaggtcatggacgcgattccgattgtctcggaattgctcgccctgggcgtgacgattgtttccactcaggaaggcgtcttccggcagggaaa
cgtcatggacctgattcacctgattatgcggctcgacgcgtcgcacaaagaatcttcgctgaagtcggcgaagattctcgacacgaagaaccttcag
cgcgaattgggcgggtacgtcggcgggaaggcgccttacggcttcgagcttgtttcggagacgaaggagatcacgcgcaacggccgaatggtca
atgtcgtcatcaacaagcttgcgcactcgaccactccccttaccggacccttcgagttcgagcccgacgtaatccggtggtggtggcgtgagatcaa
gacgcacaaacaccttcccttcaagccgggcagtcaagccgccattcacccgggcagcatcacggggctttgtaagcgcatggacgctgacgcc
gtgccgacccggggcgagacgattgggaagaagaccgcttcaagcgcctgggacccggcaaccgttatgcgaatccttcgggacccgcgtattg
cgggcttcgccgctgaggtgatctacaagaagaagccggacggcacgccgaccacgaagattgagggttaccgcattcagcgcgacccgatca
cgctccggccggtcgagcttgattgcggaccgatcatcgagcccgctgagtggtatgagcttcaggcgtggttggacggcagggggcgcggcaag
gggctttcccgggggcaagccattctgtccgccatggacaagctgtactgcgagtgtggcgccgtcatgacttcgaagcgcggggaagaatcgatc

aaggactcttaccgctgccgtcgccggaaggtggtcgacccgtccgcacctgggcagcacgaaggcacgtgcaacgtcagcatggcggcactc
gacaagttcgttgcggaacgcatcttcaacaagatcaggcacgccgaaggcgacgaagagacgttggcgcttctgtgggaagccgcccgacgct
tcggcaagctcactgaggcgcctgagaagagcggcgaacgggcgaaccttgttgcggagcgcgccgacgccctgaacgcccttgaagagctgt
acgaagaccgcgcggcaggcgcgtacgacggacccgttggcaggaagcacttccggaagcaacaggcagcgctgacgctccggcagcaag
gggcggaagagcggcttgccgaacttgaagccgccgaagccccgaagcttccccttgaccaatggttccccgaagacgccgacgctgacccga
ccggccctaagtcgtggtgggggcgcgcgtcagtagacgacaagcgcgtgttcgtcgggctcttcgtagacaagatcgttgtcacgaagtcgacta
cgggcagggggcagggaacgcccatcgagaagcgcgcttcgatcacgtgggcgaagccgccgaccgacgacgacgaagacgacgcccag
gacggcacggaagacgtagcggcgtagcgagacacccgggaagcctg

pSET154BB (expression vector)

caggcttcccgggtgtctcgctacgccgctacgtcttccgtgccgtcctgggcgtcgtcttcgtcgtcgtcggtcggcggcttcgcccacgtgatcgaag
cgcgcttctcgatgggcgttccctgcccccctgcccgtagtcgacttcgtgacaacgatcttgtctacgaagagcccgacgaacacgcgcttgtcgtcta
ctgacgcgcgcccccaccacgacttagggccggtcgggtcagcgtcggcgtcttcggggaaccattggtcaaggggaagcttcggggcttcggcg
gcttcaagttcggcaagccgctcttccgccccttgctgccggagcgtcagcgctgcctgttgcttccggaagtgcttcctgccaacgggtccgtcgtac
gcgcctgccgcgcggtcttcgtacagctcttcaagggcgttcagggcgtcggcgcgctccgcaacaaggttcgcccgttcgccgctcttctcaggcg
cctcagtgagcttgccgaagcgtcgggcggcttcccacagaagcgccaacgtctcttcgtcgccttcggcgtgcctgatcttgttgaagatgcgttccg
caacgaacttgtcgagtgccgccatgctgacgttgcacgtgccttcgtgctgcccaggtgcggacgggtcgaccaccttccggcgacggcagcggt
aagagtccttgatcgattcttccccgcgcttcgaagtcatgacggcgccacactcgcagtacagcttgtccatggcggacagaatggcttgcccccg
ggaaagccccttgccgcgccccctgccgtccaaccacgcctgaagctcataccactcagcgggctcgatgatcggtccgcaatcaagctcgaccg
gccggagcgtgatcgggtcgcgctgaatgcggtaaccctcaatcttcgtggtcggcgtgccgtccggcttcttcttgtagatcacctcagcggcgaag
cccgcaatacgcgggtcccgaaggattcgcataacggttgccgggtcccaggcgcttgaagcggtcttcttcccaatcgtctcgcccccgggtcggca
cggcgtcagcgtccatgcgcttacaaagccccgtgatgctgcccgggtgaatggcggcttgactgcccggcttgaagggaaggtgtttgtgcgtcttg
atctcacgccaccaccaccggattacgtcgggctcgaactcgaagggtccggtaaggggagtggtcgagtgcgcaagcttgttgatgacgacattg
accattcggccgttgcgcgtgatctccttcgtctccgaaacaagctcgaagccgtaaggcgccttcccgccgacgtacccgcccaattcgcgctgaa
ggttcttcgtgtcgagaatcttcgccgacttcagcgaagattctttgtgcgacgcgtcgagccgcataatcaggtgaatcaggtccatgacgtttccctg
ccggaagacgccttcctgagtggaaacaatcgtcacgcccagggcgagcaattccgagacaatcggaatcgcgtccatgaccttcaggcgcgag
aagcgcgacacgtcatagacaatgatcatgttgagccgcccggcgcggcattcgttcaggatgcgttcgaactccgggcgctccgccgtcccgaa
cgccgacgtgcccggcgcttcgctgaaatgcccgacgaacctgaaccggcccccgtcgcgctcgacttcgcgctgaaggtcggccgccttgtcttc
gttggcgctacgctgtgtcgctgggcttgctgcgctcgaattttcgcgctcgcgcgactgacggtcgtaagcacccgcgtacgtgtccaccccggtcac
aaccccttgtgtcatgtcggcgaccctacgcccccaactgagagaactcaaaggttaccccagttggggcactactcccgaaaaccgcttctgacct
gggaaaacgtgaagccccggggcatccgctgagggttgccgccggggcttcggtgtgtccgtcagtacgggccatagaggggcgtcgtgtaacc
cacccagggcgattgctccggcacggggaagccgcgccacgccttcgggacgtctggaatcgctagagcttgcatgcctggaggtcgacggatct
tttccgctgcataaccctgcttcggggtcattatagcgatttttcggtatatccatcctttttcgcacgatatacaggattttgccaaagggttcgtgtagactt
tccttggtgtatccaacggcgtcagccgggcaggataggtgaagtaggcccacccgcgagcgggtgttccttcttcactgtcccttattcgcacctggc
ggtgctcaacgggaatcctgctctgcgaggctggccggctaccgccggcgtaacagatgagggcaagcggatggctgatgaaaccaagccaac
caggaagggcagcccacctatcaaggtgtactgccttccagacgaacgaagagcgattgaggaaaaggcggcggcggccggcatgagcctgt
cggcctacctgctggccgtcggccagggctacaaaatcacgggcgtcgtggactatgagcacgtccgcgagctggcccgcatcaatggcgacct
gggccgcctgggcggcctgctgaaactctggctcaccgacgacccgcgcacggcgcggttcggtgatgccacgatcctcgccctgctggcgaag
atcgaagagaagcaggacgagcttggcaaggtcatgatgggcgtggtccgcccgagggcagagccatgactttttagccgctaaaacggccgg
ggggtgcgcgtgattgccaagcacgtccccatgcgctccatcaagaagagcgacttcgcggagctggtgaagtacatcaccgacgagcaaggc
aagaccgatccccggggacctggaggtcgactctagctagctggccagggcccgatacgtcgcggtgagttcaggctttttcatatctcattgccccc
ggacgagcgtctgctccgccattcgccgtccgccgtgccaatcggatcagccgtccaaatgcgggattttcgttagtcggaggccaaacggcattga
gcgtcagcatatcatcagcgagctgaagaaagacaatccccgatccgctccacgtgttgccccagcaatcagcgcgaccttgcccctccaacgtc
atctcgttctccgctcatgagctcagccaatcgactggcgagcggcatcgcattcttcgcatcccgcctctggcggatgcaggaagatcaacggatct
cggcccagttgacccagggctgtcgccacaatgtcgcgggagcggatcaaccgagcaaaggcatgaccgactggaccttccttctgaaggctctt
ctccttgagccacctgtccgccaaggcaaagcgctcacagcagtggtcattctcgagataatcgacgcgtaccaacttgccatcctgaagaatggtg
cagtgtctcggcaccccatagggaacctttgccatcaactcggcaagatgcagcgtcgtgttggcatcgtgtcccacgccgaggagaagtacctgc
ccatcgagttcatggacacgggcgaccgggcttgcaggcgagtgaggtggcaggggcaatggatcagagatgatctgctctgcctgtggccccgc
tgccgcaaaggcaaatggatgggcgctgcgctttacatttggcaggcgccagaatgtgtcagagacaactccaaggtccggtgtaacgggcgac
gtggcaggatcgaacggctcgtcgtccagacctgaccacgagggcatgacgagcgtccctcccggacccagcgcagcacgcagggcctcgatc
agtccaagtggcccatcttcgaggggccggacgctacggaaggagctgtggaccagcagcacaccgccgggggtaaccccaaggttgagaag
ctgaccgatgagctcggcttttcgccattcgtattgcacgacattgcactccaccgctgatgacatcagtcgatcatagcacgatcaacggcactgttg
caaatagtcggtggtgataaacttatcatccccttttgctgatggagctgcacatgaaccaaaaggatctaggtgaagatcctttttgataatctcatgac
caaaatcccttaacgtgagttttcgttccactgagcgtcagaccccgtagaaaagatcaaaggatcttcttgagatcctttttttctgcgcgtaatctgctg
cttgcaaacaaaaaaaccaccgctaccagcggtggtttgtttgccggatcaagagctaccaactctttttccgaaggtaactggcttcagcagagcg

cagataccaaatactgttcttctagtgtagccgtagttaggccaccacttcaagaactctgtagcaccgcctacatacctcgctctgctaatcctgttacc
agtggctgctgccagtggcgataagtcgtgtcttaccgggttggactcaagacgatagttaccggataaggcgcagcggtcgggctgaacggggg
gttcgtgcacacagcccagcttggagcgaacgacctacaccgaactgagatacctacagcgtgagctatgagaaagcgccacgcttcccgaagg
gagaaaggcggacaggtatccggtaagcggcagggtcggaacaggagagcgcacgagggagcttccagggggaaacgcctggtatctttata
gtcctgtcgggtttcgccacctctgacttgagcgtcgatttttgtgatgctcgtcaggggggcggagcctatggaaaaacgccagcaacgcggcctttttt
acggttcctggccttttgctggccttttgctcacatgttctttcctgcgttatcccctgattctgtggataaccgtattaccgcctttgagtgagctgataccgct
cgccgcagccgaacgaccgagcgcagcgagtcagtgagcgaggaagcggaagagcgcccaatacgcaaaccgcctctccccgcgcgttgg
ccgattcattaatgcagctggcacgacaggtttcccgactggaaagcgggcagtgagcgcaacgcaattaatgtgagttagctcactcattaggcac
cccaggctttacactttatgcttccggctcgtatgttgtgtggaattgtgagcggataacaatttcacacaggaaacagctatgacaaaaaaaaaaag
ccccgcgattgcggggcttttttttttgattacgaattcgatatcgcgcgcggccgcggatcctctagagtcgacctgcagcccaagtctaaagttttgtc
gtctttccagacgttagtaaatgaattttctgtatgaggttttgctaaacaactttcaacagtttcagcggagtgagaatagaaaggaacaactaaagga
attgcgaataataatttttcacgttgaaaatctccaaaaaaaaagggctccaaaaggagcctttaattgtatcggtttatcagcttgctttcgaggtgaattt
cttaaacagcttgataccgatagttgcgccgacaatgacaacaaccatcgcccacgcataaccgatatattcggtcgctgaggcttgcagggagtc
aaaggccgcttttgcgggacttggcactggccgtcgttttacaacgtcgtgactgggaaaaccctggcgttacccaacttaatcgccttgcagcacatc
ccccttcgccagctggcgtaatagcgaagaggcccgcaccgatcgcccttcccaacagttgcgcagcctgaatggcgaatggcgcctgatgcgg
tattttctccttacgcatctgtgcggtatttcacaccgcataaattccccaatgtcaagcacttccggaatcgggagcgcggccgatgcaaagtgccgat
aaacataacgatctttgtagaaaccatcggcgcagctatttacccgcaggacatatccacgccctcctacatcgaagctgaaagcacgagattcttc
gccctccgagagctgcatcaggtcggagacgctgtcgaacttttcgatcagaaacttctcgacagacgtagat

pENSV1 (expression vector)

ccaatgatgaattcgcggccgcttctagagatacatgaacatgcaataccaatacgcaaaccgcctctccccgcgcgttggccgattcattaatgca
gctggcacgacaggtttcccgactggaaagcgggcagtgagcgcaacgcaattaatgtgagttagctcactcattaggcaccccaggctttacactt
tatgcttccggctcgtatgttgtgtggaattgtgagcggataacaatttcacacatactagagaaagaggagaaatactagatggcttcctccgaaga
cgttatcaaagagttcatgcgtttcaaagttcgtatggaaggttccgttaacggtcacgagttcgaaatcgaaggtgaaggtgaaggtcgtccgtacg
aaggtacccagaccgctaaactgaaagttaccaaaggtggtccgctgccgttcgcttgggacatcctgtccccgcagttccagtacggttccaaagc
ttacgttaaacacccggctgacatcccggactacctgaaactgtccttcccggaaggtttcaaatgggaacgtgttatgaacttcgaagacggtggtgt
tgttaccgttacccaggactcctccctgcaagacggtgagttcatctacaaagttaaactgcgtggtaccaacttcccgtccgacggtccggttatgca
gaaaaaaaccatgggttgggaagcttccaccgaacgtatgtacccggaagacggtgctctgaaaggtgaaatcaaaatgcgtctgaaactgaaa
gacggtggtcactacgacgctgaagttaaaaccacctacatggctaaaaaaccggttcagctgccgggtgcttacaaaaccgacatcaaactgga
catcacctcccacaacgaagactacaccatcgttgaacagtacgaacgtgctgaaggtcgtcactccaccggtgcttaataacgctgatagtgctag
tgtagatcgctactagagccaggcatcaaataaaacgaaaggctcagtcgaaagactgggcctttcgttttatctgttgtttgtcggtgaacgctctcta
ctagagtcacactggctcaccttcgggtgggcctttctgcgtttataaacgctctctactagtagcggccgctgcaggtgtaggctggagctgcttggaa
taggaacttcatgagctcagccaatcgactggcgagcggcatcttatttgccgactaccttggtgatctcgcctttcacgtagtggacaaattcttccaa
ctgatctgcgcgcgaggccaagcgatcttcttcttgtccaagataagcctgtctagcttcaagtatgacgggctgatactgggccggcaggcgctcca
ttgcccagtcggcagcgacatccttcggcgcgattttgccggttactgcgctgtaccaaatgcgggacaacgtaagcactacatttcgctcatcgcca
gcccagtcgggcggcgagttccatagcgttaaggtttcatttagcgcctcaaatagatcctgttcaggaaccggatcaaagagttcctccgccgctgg
acctaccaaggcaacgctatgttctcttgcttttgtcagcaagatagccagatcaatgtcgatcgtggctggctcgaagatacctgcaagaatgtcattg
cgctgccattctccaaattgcagttcgcgcttagctggataacgccacggaatgatgtcgtcgtgcacaacaatggtgacttctacagcgcggagaat
ctcgctctctccaggggaagccgaagtttccaaaaggtcgttgatcaaagctcgccgcgttgtttcatcaagccttacggtcaccgtaaccagcaaat
caatatcactgtgtggcttcaggccgccatccactgcggagccgtacaaatgtacggccagcaacgtcggttcgagatggcgctcgatgacgccaa
ctacctctgatagttgagtcgatacttcggcgatcaccgcttccctcatgacattgcactccaccgctgatgacatcagtcgatcatagcacgatcaac
ggcactgttgcaaatagtcggtggtgataaacttatcatccccttttgctgatggagctgcacatgaacccattcaaaggccggcattttcagcgtgaca
tcattctgtgggccgtacgctggtactgcaaatacggcatcagttaccgtgagctgcattttccgctgcataaccctgcttcggggtcattatagcgattttt
tcggtatatccatcctttttcgcacgatatacaggattttgccaaagggttcgtgtagactttccttggtgtatccaacggcgtcagccgggcaggatagg
tgaagtaggcccacccgcgagcgggtgttccttcttcactgtcccttattcgcacctggcggtgctcaacgggaatcctgctctgcgaggctggcggg
aacttcgaactccaggtcgacggatccccggatcgcgctccgatgtggtcctttagatccactgacgtgggtcagtgtctctaaaggactcgcgagca
tcgttcccctctccctgcactgaggtgcggttttcagagggtggcagcaggcggagaaacaatcaagcgcgcctgcatggtttgcgctccagcggt
caagaccaggggtcgggctcccacacgggatgaaactcgaagctgcgcacgacactccactgtgcgccctgatcgctcttacggttgtggatgac
cagacggcgcagcaggcggcgcaggatggcgttcttctcggtggtgtgcaggatgtcccactcctgcaagagcccgacgatcagggggcggaa
ctcctcccgcgtgggcgcgacttcaacctcgctgagcgacttcaagtgcttgatgatgtcgcccttcttaccgagtagctggtcgcgtacgcgcccgaa
cgtgtccgccgggtacttgtccgggtccagcgcgtaatccgtgacgagacggtcgagtgcaccctcgatcttggccaactcggcttccgtgcgggtg
cgctcctcgaccaggcgggcccgcgggtccggcgcggtgcccggcgcggtgcgctgagcgggaagggccggcgcgttgtcgatgtcgtcggcg
accgtgtcggcgagccacttcaacacctcggcctcgacttcgtcgcggcgcacgtagaggccgggctcacaggccgacttccccttgttcctgcggt
tgaagcacacgaacacgtggcccggaacgaatccgcccttcccgtcgcggccggatcgcgcaacggccgtcccgcggcagtgtccgtgccgca
tgatgccgctggtcgggtatgaggcccggcgggcgcgcgggggcgtcttgcgcgtctgctctctgtgcgccccgtactccttccactgctcgggaac

gatgagcgccggctgtgccccggggagccagagccaccggttttctttgcacgcagagaagtggtcctgtcccagcttgcaccggcactccgggtc
gtggacgcgtagcaggccggcggcgaatccggagtcgaggtagcgctgaacggtgttggtgccccagcggttgccccgtgtggtggggatgagt
agttcgtcgttcagccagtaggcgagctgggagaacccctgtccggcgagctttcgctcgtagagttcggccgccacgggggcgaactctgggtgc
cgttcgtagcgctcctcttgcaggcggaacccgcccggcgcggtgaggtcgggtacgcgcctcgggtgccacacgtagccgaaccgctgacgcc
cggtggcggggagtttcagggcccgacggtgggcgtgcgtctccttccactgttcgccggcccgatccgactcgaatacggcgaggtcgaacaga
atcgcgcggttgaagcgtccgacggccgtgcgggcgtcgacttcttccgtggcggacgcgaggtctccgccggcctgttcgaggcgggcgaggttg
atagcgatgcccaggtcgttccggccgaagcggctgaacttccatacggcgattccgacggcctcgcggccctcgacgcgctggatgccgcccat
gatcttccgcttgaagttgcggcccgtagcgtcgaggtcaacgatccagtcgacgatccgacgtcccgttcgggcggcccatgactcgatcgcggat
tgctgtagctccgggctgatcttctcctcgcgccatgtgctgaccctgatgtagccgagccacggctcgcccggcgtgcgggagccgcggaacgtgc
ttggtaggtctcgtttcatggttgaaagatagcgcgcgttctgccatgtgggcaggggcaacgtagaagatccagttttgtcccatagtcgccccccttg
ccccatgcgatagcccgtacgagccccttcgttcgtacgcatgtccgattctcatacgtgcgactaccgggcgtcaacgtgcgtttccacaaagcgttt
gaagacctaacttccaaacttgaaaattagctgctaactttcaacctcggtagttagttcagccggaaggaacgccccccatcggaaagaacatgtg
agcaaaaggccagcaaaaggccaggaaccgtaaaaaggccgcgttgctggcgtttttccataggctccgcccccctgacgagcatcacaaaaa
tcgacgctcaagtcagaggtggcgaaacccgacaggactataaagataccaggcgtttccccctggaagctccctcgtgcgctctcctgttccgac
cctgccgcttaccggatacctgtccgcctttctcccttcgggaagcgtggcgctttctcatagctcacgctgtaggtatctcagttcggtgtaggtcgttcg
ctccaagctgggctgtgtgcacgaaccccccgttcagcccgaccgctgcgccttatccggtaactatcgtcttgagtccaacccggtaagacacgac
ttatcgccactggcagcagccactggtaacaggattagcagagcgaggtatgtaggcggtgctacagagttcttgaagtggtggcctaactacggct
acactagaagaacagtatttggtatctgcgctctgctgaagccagttaccttcggaaaaagagttggtagctcttgatccggcaaacaaaccaccgct
ggtagcggtggtttttttgtttgcaagcagcagattacgcgcagaaaaaaaggatctcaagaagatcctttgatcttttctacggggtctgacgctcagt
ggaacgaaaactcacgttaagggattttggtcatgagattatcaaaaaggatcttcacctagatccttttaaattaaaaatgaagttttaaatcaatctaa
agtatatatgagtaaacttggtctgacagttaccaatgcttaatcagtgaggcacctatctcagcgatctgtctatttcgttcatccatagttgcctgactcc
ccgtcgtgtagataactacgatacgggagggcttaccatctggccccagtgctgcaatgataccgcgagacccacgctcaccggctccagatttatc
agcaataaaccagccagccggaagggccgagcgcagaagtggtcctgcaactttatccgcctccatccagtctattaattgttgccgggaagctag
agtaagtagttcgccagttaatagtttgcgcaacgttgttgccattgctacaggcatcgtggtgtcacgctcgtcgtttggtatggcttcattcagctccggt
tcccaacgatcaaggcgagttacatgatcccccatgttgtgcaaaaaagcggttagctccttcggtcctccgatcgttgtcagaagtaagttggccgc
agtgttatcactcatggttatggcagcactgcataattctcttactgtcatgccatccgtaagatgcttttctgtgactggtgagtactcaaccaagtcattct
gagaatagtgtatgcggcgaccgagttgctcttgcccggcgtcaatacgggataataccgcgccacatagcagaactttaaaagtgctcatcattgg
aaaacgttcttcggggcgaaaactctcaaggatcttaccgctgttgagatccagttcgatgtaacccactcgtgcacccaactgatcttcagcatctttt
actttcaccagcgtttctgggtgagcaaaaacaggaaggcaaaatgccgcaaaaaagggaataagggcgacacggaaatgttgaatactcata
ctcttcctttttcaatattattgaagcatttatcagggttattgtctcatgagcggatacatatttgaatgtatttagaaaaataaacaaataggggttccgcg
cacatttccccgaaaagtgccacctgacgtc

pENSV3 (expression vector)

cgtcagtttcacctgttttacgtaaaaacccgcttcggcgggtttttacttttggcaatgatgaattcgcggccgcttctagagatacatgaacatgcaata
cgcaaaccgcctctccccgcgcgttggccgattcattaatgcagctggcacgacaggtttcccgactggaaagcgggcagtgagcgcaacgcaat
taatgtgagttagctcactcattaggcaccccaggctttacactttatgcttccggctcgtatgttgtgtggaattgtgagcggataacaatttcacacata
ctagagaaagaggagaaatactagatggcttcctccgaagacgttatcaaagagttcatgcgtttcaaagttcgtatggaaggttccgttaacggtca
cgagttcgaaatcgaaggtgaaggtgaaggtcgtccgtacgaaggtacccagaccgctaaactgaaagttaccaaaggtggtccgctgccgttcg
cttgggacatcctgtccccgcagttccagtacggttccaaagcttacgttaaacacccggctgacatcccggactacctgaaactgtccttcccggaa
ggtttcaaatgggaacgtgttatgaacttcgaagacggtggtgttgttaccgttacccaggactcctccctgcaagacggtgagttcatctacaaagtta
aactgcgtggtaccaacttcccgtccgacggtccggttatgcagaaaaaaaccatgggttgggaagcttccaccgaacgtatgtacccggaagac
ggtgctctgaaaggtgaaatcaaaatgcgtctgaaactgaaagacggtggtcactacgacgctgaagttaaaaccacctacatggctaaaaaacc
ggttcagctgccgggtgcttacaaaaccgacatcaaactggacatcacctcccacaacgaagactacaccatcgttgaacagtacgaacgtgctg
aaggtcgtcactccaccggtgcttaataacgctgatagtgctagtgtagatcgctactagagccaggcatcaaataaaacgaaaggctcagtcgaa
agactgggcctttcgttttatctgttgtttgtcggtgaacgctctctactagagtcacactggctcaccttcgggtgggcctttctgcgtttataaacgctctct
actagtagcggccgctgcaggtgtagttcagccaaaaaacttaagaccgccggtcttgtccactaccttgcagtaatgcggtggacaggatcggcg
gttttcttttctcttctcaagctggagctgcttggaataggaacttcatgagctcagccaatcgactggcgagcggcatcttatttgccgactaccttggtga
tctcgcctttcacgtagtggacaaattcttccaactgatctgcgcgcgaggccaagcgatcttcttcttgtccaagataagcctgtctagcttcaagtatg
acgggctgatactgggccggcaggcgctccattgcccagtcggcagcgacatccttcggcgcgattttgccggttactgcgctgtaccaaatgcggg
acaacgtaagcactacatttcgctcatcgccagcccagtcgggcggcgagttccatagcgttaaggtttcatttagcgcctcaaatagatcctgttcag
gaaccggatcaaagagttcctccgccgctggacctaccaaggcaacgctatgttctcttgcttttgtcagcaagatagccagatcaatgtcgatcgtg
gctggctcgaagatacctgcaagaatgtcattgcgctgccattctccaaattgcagttcgcgcttagctggataacgccacggaatgatgtcgtcgtgc
acaacaatggtgacttctacagcgcggagaatctcgctctctccaggggaagccgaagtttccaaaaggtcgttgatcaaagctcgccgcgttgtttc
atcaagccttacggtcaccgtaaccagcaaatcaatatcactgtgtggcttcaggccgccatccactgcggagccgtacaaatgtacggccagcaa
cgtcggttcgagatggcgctcgatgacgccaactacctctgatagttgagtcgatacttcggcgatcaccgcttccctcatgacattgcactccaccgc

tgatgacatcagtcgatcatagcacgatcaacggcactgttgcaaatagtcggtggtgataaacttatcatccccttttgctgatggagctgcacatga
acccattcaaaggccggcattttcagcgtgacatcattctgtgggccgtacgctggtactgcaaatacggcatcagttaccgtgagctgcattttccgct
gcataaccctgcttcggggtcattatagcgatttttcggtatatccatcctttttcgcacgatatacaggattttgccaaagggttcgtgtagactttccttgg
tgtatccaacggcgtcagccgggcaggataggtgaagtaggcccacccgcgagcgggtgttccttcttcactgtcccttattcgcacctggcggtgct
caacgggaatcctgctctgcgaggctggcgggaacttcgaactccaggtcgacggatccccggatcgcgctccgatgtggtcctttagatccactga
cgtgggtcagtgtctctaaaggactcgcgagcatcgttcccctctccctgcactgaggtgcggttttcagagggtggcagcaggcggagaaacaat
caagcgcgcctgcatggtttgcgctccagcggtcaagaccaggggtcgggctcccacacgggatgaaactcgaagctgcgcacgacactccact
gtgcgccctgatcgctcttacggttgtggatgaccagacggcgcagcaggcggcgcaggatggcgttcttctcggtggtgtgcaggatgtcccactc
ctgcaagagcccgacgatcaggggggcggaactcctcccgcgtgggcgcgacttcaacctcgctgagcgacttcaagtgcttgatgatgtcgccctt
cttaccgagtagctggtcgcgtacgcgcccgaacgtgtccgccgggtacttgtccgggtccagcgcgtaatccgtgacgagacggtcgagtgcacc
ctcgatcttggccaactcggcttccgtgcgggtgcgctcctcgaccaggcgggcccgcgggtccggcgcggtgcccggcgcggtgcgctgagcgg
gaagggccggcgcgttgtcgatgtcgtcggcgaccgtgtcggcgagccacttcaacacctcggcctcgacttcgtcgcggcgcacgtagaggccg
ggctcacaggccgacttccccttgttcctgcggttgaagcacacgaacacgtggcccggaacgaatccgcccttcccgtcgcggccggatcgcgc
aacggccgtcccgcggcagtgtccgtgccgcatgatgccgctggtcgggtatgaggcccggcgggcgcgcgggggcgtcttgcgcgtctgctctct
gtgcgccccgtactccttccactgctcgggaacgatgagcgccggctgtgccccggggagccagagccaccggttttctttgcacgcagagaagtg
gtcctgtcccagcttgcaccggcactccgggtcgtggacgcgtagcaggccggcggcgaatccggagtcgaggtagcgctgaacggtgttggtgc
cccagcggttgccccgtgtggtggggatgagtagttcgtcgttcagccagtaggcgagctgggagaacccctgtccggcgagctttcgctcgtagag
ttcggccgccacgggggcgaactctgggtgccgttcgtagcgctcctcttgcaggcggaacccgcccggcgcggtgaggtcgggtacgcgcctcg
ggtgccacacgtagccgaaccgctgacgcccggtggcggggagtttcagggcccgacggtgggcgtgcgtctccttccactgttcgccggcccga
tccgactcgaatacggcgaggtcgaacagaatcgcgcggttgaagcgtccgacggccgtgcgggcgtcgacttcttccgtggcggacgcgaggt
ctccgccggcctgttcgaggcgggcgaggttgatagcgatgcccaggtcgttccggccgaagcggctgaacttccatacggcgattccgacggcct
cgcggccctcgacgcgctggatgccgcccatgatcttccgcttgaagttgcggcccgtagcgtcgaggtcaacgatccagtcgacgatccgacgtc
ccgttcgggcggcccatgactcgatcgcggattgctgtagctccgggctgatcttctcctcgcgccatgtgctgaccctgatgtagccgagccacggct
cgcccggcgtgcgggagccgcggaacgtgcttggtaggtctcgtttcatggttgaaagatagcgcgcgttctgccatgtgggcaggggcaacgtag
aagatccagttttgtcccatagtcgccccccttgccccatgcgatagcccgtacgagccccttcgttcgtacgcatgtccgattctcatacgtgcgacta
ccgggcgtcaacgtgcgtttccacaaagcgtttgaagacctaacttccaaacttgaaaattagctgctaactttcaacctcggtagttagttcagccgg
aaggaacgccccccatcggaaagaacatgtgagcaaaaggccagcaaaaggccaggaaccgtaaaaaggccgcgttgctggcgtttttccata
ggctccgcccccctgacgagcatcacaaaaatcgacgctcaagtcagaggtggcgaaacccgacaggactataaagataccaggcgtttcccc
ctggaagctccctcgtgcgctctcctgttccgaccctgccgcttaccggatacctgtccgcctttctcccttcgggaagcgtggcgctttctcatagctcac
gctgtaggtatctcagttcggtgtaggtcgttcgctccaagctgggctgtgtgcacgaaccccccgttcagcccgaccgctgcgccttatccggtaact
atcgtcttgagtccaacccggtaagacacgacttatcgccactggcagcagccactggtaacaggattagcagagcgaggtatgtaggcggtgcta
cagagttcttgaagtggtggcctaactacggctacactagaagaacagtatttggtatctgcgctctgctgaagccagttaccttcggaaaaagagttg
gtagctcttgatccggcaaacaaaccaccgctggtagcggtggtttttttgtttgcaagcagcagattacgcgcagaaaaaaaggatctcaagaaga
tcctttgatcttttctacggggtctgacgctcagtggaacgaaaactcacgttaagggattttggtcatgagattatcaaaaaggatcttcacctagatcct
tttaaattaaaaatgaagttttaaatcaatctaaagtatatatgagtaaacttggtctgacagttaccaatgcttaatcagtgaggcacctatctcagcgat
ctgtctatttcgttcatccatagttgcctgactccccgtcgtgtagataactacgatacgggagggcttaccatctggccccagtgctgcaatgataccgc
gagacccacgctcaccggctccagatttatcagcaataaaccagccagccggaagggccgagcgcagaagtggtcctgcaactttatccgcctc
catccagtctattaattgttgccgggaagctagagtaagtagttcgccagttaatagtttgcgcaacgttgttgccattgctacaggcatcgtggtgtcac
gctcgtcgtttggtatggcttcattcagctccggttcccaacgatcaaggcgagttacatgatcccccatgttgtgcaaaaaagcggttagctccttcggt
cctccgatcgttgtcagaagtaagttggccgcagtgttatcactcatggttatggcagcactgcataattctcttactgtcatgccatccgtaagatgctttt
ctgtgactggtgagtactcaaccaagtcattctgagaatagtgtatgcggcgaccgagttgctcttgcccggcgtcaatacgggataataccgcgcca
catagcagaactttaaaagtgctcatcattggaaaacgttcttcggggcgaaaactctcaaggatcttaccgctgttgagatccagttcgatgtaaccc
actcgtgcacccaactgatcttcagcatcttttactttcaccagcgtttctgggtgagcaaaaacaggaaggcaaaatgccgcaaaaaagggaata
agggcgacacggaaatgttgaatactcatactcttcctttttcaatattattgaagcatttatcagggttattgtctcatgagcggatacatatttgaatgtatt
tagaaaaataaacaaataggggttccgcgcacatttccccgaaaagtgccacctgacgtc

pENTG1 (expression vector)

ccaatgatgaattcgcggccgcttctagagatacatgaacatgcaataccaatacgcaaaccgcctctccccgcgcgttggccgattcattaatgca
gctggcacgacaggtttcccgactggaaagcgggcagtgagcgcaacgcaattaatgtgagttagctcactcattaggcaccccaggctttacactt
tatgcttccggctcgtatgttgtgtggaattgtgagcggataacaatttcacacatactagagaaagaggagaaatactagatggcttcctccgaaga
cgttatcaaagagttcatgcgtttcaaagttcgtatggaaggttccgttaacggtcacgagttcgaaatcgaaggtgaaggtgaaggtcgtccgtacg
aaggtacccagaccgctaaactgaaagttaccaaaggtggtccgctgccgttcgcttgggacatcctgtccccgcagttccagtacggttccaaagc
ttacgttaaacacccggctgacatcccggactacctgaaactgtccttcccggaaggtttcaaatgggaacgtgttatgaacttcgaagacggtggtgt
tgttaccgttacccaggactcctccctgcaagacggtgagttcatctacaaagttaaactgcgtggtaccaacttcccgtccgacggtccggttatgca
gaaaaaaaccatgggttgggaagcttccaccgaacgtatgtacccggaagacggtgctctgaaaggtgaaatcaaaatgcgtctgaaactgaaa

gacggtggtcactacgacgctgaagttaaaaccacctacatggctaaaaaccggttcagctgccgggtgcttacaaaaccgacatcaaactgga
catcacctcccacaacgaagactacaccatcgttgaacagtacgaacgtgctgaaggtcgtcactccaccggtgcttaataacgctgatagtgctag
tgtagatcgctactagagccaggcatcaaataaaacgaaaggctcagtcgaaagactgggcctttcgttttatctgttgtttgtcggtgaacgctctcta
ctagagtcacactggctcaccttcgggtgggcctttctgcgtttataaacgctctctactagtagcggccgctgcaggtgtaggctggagctgcttcgga
ataggaacttcatgagctcagccaatcgactggcgagcggcatcctaccggtagccgctgaggccgtcggcgagttcctcctcgtcgccgtcgcgc
tgcgcgtagagggcctgctggagtgcgaaggtgcctcggatcgccgagatccgctcggccgttccgttgtcggcccagccgccgagcgcgagca
ctcggcccagcagttcctcgccgtagctcgccccgatggcggccaggtcctcagccgggtcgccgatgccgacctcgtcccagtcgacgacgccg
ctcatgcgcggcactccgtccaccgtctcccacaggacgttctcgccgccgaggtcaccgtggaccaccgcggaggtgagatggggcagggcgt
cgagcgcggcgagctcgcgctcggcacgctcccggccgccgtcggacatcagcgggaacagttcggtacgcacccccgtggcgaactcctgcc
actcgttcgcgggagcctccggcagcgcggcgcgcaccttctcctcgtcgcccgccgccgcgagcccggacagcagggtcgcgtactgtcgggc
gacggcctccgccacctccgggctggtgagcacatcgtcctccaacggtgctccgggaatgcggctcagcaccaggtacggcggctcgtccgtgc
cctgggcgccgccctcggacagcggctgcggcgtgcgaaacccgaggtcgatcccggcaagagcgcgcaggacgtccgccctgccgggcag
acggtcggcggccgcccgggtgcgggcgaagcagaccacccggtgcgatccgatcaccacatggtggaactgcccctcgtggacggcgagtc
cgcccacggtgtccccgggcaggagccggctcagcagatcgcggtgcgtctcaatgattctcatgacattgcactccaccgctgatgacatcagtc
gatcatagcacgatcaacggcactgttgcaaatagtcggtggtgataaacttatcatccccttttgctgatggagctgcacatgaacccattcaaaggc
cggcattttcagcgtgacatcattctgtgggccgtacgctggtactgcaaatacggcatcagttaccgtgagctgcattttccgctgcataaccctgcttc
ggggtcattatagcgatttttcggtatatccatcctttttcgcacgatatacaggattttgccaaagggttcgtgtagactttccttggtgtatccaacggcgt
cagccgggcaggataggtgaagtaggcccacccgcgagcgggtgttccttcttcactgtcccttattcgcacctggcggtgctcaacgggaatcctg
ctctgcgaggctggcgggaacttcgaactccaggtcgacggatccccggaatgtaagcgtcacggcacgcgccgactgagagacgtttccgcag
gtcaaccccgttccagcccaacagtgttagtctttgctcttacccagttgggcgggatagcctgcccggcatgagcgtgaaggttgaaggcatggtca
ttctggcaggcggctacgaccgacagtcggcggaacgggagaacagttcgaccgcttcaccggccacccagcgcgccgcgaaccgggggaa
ggctgaggcgctggcgaaggagtacgcgcgcgacggcgtcgaggtgaagtggctgggtcacttcagcgaagcgcccggcacgtcggcattcac
gggcgtcgaccggccggagttcaaccggattttggacatgtgccggaaccgggaaatgaacatgatcattgttcattacatttcgcgcctcagccgc
gaagagccgctggacattattccggtcgtcacggaattgctccggctgggcgtgaccattgtcagcgtgaacgaaggcacattccgccccggcga
aatgatggaccttattcacctgatcatgcgccttcaggcttcgcatgatgagtcgaagaacaagagcgtcgccgtgtcgaacgctaaggaattggcg
aagcggctgggcggacacacggggtcgacgccgtacggattcgacacggtcgaggaaatggttccgaacccggaagacggcggaaagctgg
ttgccattcgccgactggtgcccagcgcgcacacctgggaaggcgcacacggcagcgaaggggcggtaatccgctgggcgtggcaggagatc
aagacgcaccgcgatacgccattcaagggtggcggagccgggtcgtttcaccctgggtcgctgaacgggctttgtgagcggctgtaccgcgacaa
ggtgcctacgcgcggcacgctggtcggtaagaagcgcgccggttccgattgggaccccggcgttttgaagcgcgtactcagcgacccgcgcattg
ccgggtatcaagctgacatcgcatacaaggtgcgcgccgacggttcgcggggcggcttcagccattacaagatcaggcgcgacccggtcaccat
ggagccgctgaccctgcccggcttcgagccgtacattcccccggcggaatggtgggaacttcaggagtggcttcagggtcgaggacgcgggaag
ggtcagtaccgggggcaatcgctcctgtcggcaatggacgtcctttactgctacggctccggccagctcgacccggagacgggttacagcaacgg
gtcgaccatggcgggcaacgtccgcgaaggtgatcaagctcacaagtcgtcgtacgcgtgcaagtgcccccgccgggttcatgacgggtcgtcat
gctcgatcacgatgcacaaccttgacccgtacatcgtcggcgcgatcttcgcgcgcatcacggccttcgaccctgccgaccctgacgacctcgaag
gcgacacggcagcgctcatgtacgaagccgcacggcgctggggagcgacgcacgaacgcccggagttgaagggtcagcgctccgaactgat
ggcacagcgcgcggacgccgtgaaggcgctcgaagagctttacgaagacaagcggaacggcggctaccggtccgccatgggacggcgcgc
gtttctcgaagaggaagccgcgctgacgctccgcatggaaggggccgaagaacggcttcgtcagctcgacgccgccgactcccccgtgctgccg
atcggcgaatggctgggcgaccggggcagcgacccgacgggaccgggttcgtggtgggcgctagcgccccttgaagaccgtcgggcgttcgtcc
ggctcttcgtggaccggatcgaggtgatcaagcttccgaagggcgttcagcggcccggacgggttcccccgatcgccgaccgtgtgcgtatccact
gggcgaagccgaaggtcgaggaagagacggagccggagacgctgaacgggttcacagcggcggcgtgacggcggcaccagcgcaacgg
gaaggggcttcggccccttttctcgtgcccggcgtcggttcgttgccctaagcaactgttcctagcgatcggaaagaacatgtgagcaaaaggccag
caaaaggccaggaaccgtaaaaaggccgcgttgctggcgtttttccataggctccgcccccctgacgagcatcacaaaaatcgacgctcaagtca
gaggtggcgaaacccgacaggactataaagataccaggcgtttccccctggaagctccctcgtgcgctctcctgttccgaccctgccgcttaccgga
tacctgtccgcctttctcccttcgggaagcgtggcgctttctcatagctcacgctgtaggtatctcagttcggtgtaggtcgttcgctccaagctgggctgtg
tgcacgaaccccccgttcagcccgaccgctgcgccttatccggtaactatcgtcttgagtccaacccggtaagacacgacttatcgccactggcagc
agccactggtaacaggattagcagagcgaggtatgtaggcggtgctacagagttcttgaagtggtggcctaactacggctacactagaagaacag
tatttggtatctgcgctctgctgaagccagttaccttcggaaaaagagttggtagctcttgatccggcaaacaaaccaccgctggtagcggtggtttttt
gtttgcaagcagcagattacgcgcagaaaaaaaggatctcaagaagatcctttgatcttttctacggggtctgacgctcagtggaacgaaaactcac
gttaagggattttggtcatgagattatcaaaaaggatcttcacctagatccttttaaattaaaaatgaagttttaaatcaatctaaagtatatatgagtaaa
cttggtctgacagttaccaatgcttaatcagtgaggcacctatctcagcgatctgtctatttcgttcatccatagttgcctgactccccgtcgtgtagataac
tacgatacgggagggcttaccatctggccccagtgctgcaatgataccgcgagacccacgctcaccggctccagatttatcagcaataaaccagc
cagccggaagggccgagcgcagaagtggtcctgcaactttatccgcctccatccagtctattaattgttgccgggaagctagagtaagtagttcgcc
agttaatagtttgcgcaacgttgttgccattgctacaggcatcgtggtgtcacgctcgtcgtttggtatggcttcattcagctccggttcccaacgatcaag
gcgagttacatgatcccccatgttgtgcaaaaaagcggttagctccttcggtcctccgatcgttgtcagaagtaagttggccgcagtgttatcactcatg
gttatggcagcactgcataattctcttactgtcatgccatccgtaagatgcttttctgtgactggtgagtactcaaccaagtcattctgagaatagtgtatgc
ggcgaccgagttgctcttgcccggcgtcaatacgggataataccgcgccacatagcagaactttaaaagtgctcatcattggaaaacgttcttcggg

gcgaaaactctcaaggatcttaccgctgttgagatccagttcgatgtaacccactcgtgcacccaactgatcttcagcatcttttactttcaccagcgtttc
tgggtgagcaaaaacaggaaggcaaaatgccgcaaaaaagggaataagggcgacacggaaatgttgaatactcatactcttcctttttcaatatta
ttgaagcatttatcagggttattgtctcatgagcggatacatatttgaatgtatttagaaaaataaacaaataggggttccgcgcacatttccccgaaaa
gtgccacctgacgtc

pENTG3 (expression vector)

ccactcggtaccaaattccagaaaagaggcctcccgaaagggggccttttttcgttttggtccatgatgaattcgcggccgcttctagagatacatga
acatgcaataccaatacgcaaaccgcctctccccgcgcgttggccgattcattaatgcagctggcacgacaggtttcccgactggaaagcgggca
gtgagcgcaacgcaattaatgtgagttagctcactcattaggcaccccaggctttacactttatgcttccggctcgtatgttgtgtggaattgtgagcgga
taacaatttcacacatactagagaaagaggagaaatactagatggcttcctccgaagacgttatcaaagagttcatgcgtttcaaagttcgtatggaa
ggttccgttaacggtcacgagttcgaaatcgaaggtgaaggtgaaggtcgtccgtacgaaggtacccagaccgctaaactgaaagttaccaaagg
tggtccgctgccgttcgcttgggacatcctgtccccgcagttccagtacggttccaaagcttacgttaaacacccggctgacatcccggactacctgaa
actgtccttcccggaaggtttcaaatgggaacgtgttatgaacttcgaagacggtggtgttgttaccgttacccaggactcctccctgcaagacggtga
gttcatctacaaagttaaactgcgtggtaccaacttcccgtccgacggtccggttatgcagaaaaaaaccatgggttgggaagcttccaccgaacgt
atgtacccggaagacggtgctctgaaaggtgaaatcaaaatgcgtctgaaactgaaagacggtggtcactacgacgctgaagttaaaaccaccta
catggctaaaaaaccggttcagctgccgggtgcttacaaaaccgacatcaaactggacatcacctcccacaacgaagactacaccatcgttgaac
agtacgaacgtgctgaaggtcgtcactccaccggtgcttaataacgctgatagtgctagtgtagatcgctactagagccaggcatcaaataaaacg
aaaggctcagtcgaaagactgggcctttcgttttatctgttgtttgtcggtgaacgctctctactagagtcacactggctcaccttcgggtgggcctttctgc
gtttataaacgctctctactagtagcggccgctgcaggtgtaaaaaaaaaaaaaacaccctaacgggtgttttttttttttggtctcccggctggagctgct
tcggaataggaacttcatgagctcagccaatcgactggcgagcggcatcctaccggtagccgctgaggccgtcggcgagttcctcctcgtcgccgt
cgcgctgcgcgtagagggcctgctggagtgcgaaggtgcctcggatcgccgagatccgctcggccgttccgttgtcggcccagccgccgagcgc
gagcactcggcccagcagttcctcgccgtagctcgccccgatggcggccaggtcctcagccgggtcgccgatgccgacctcgtcccagtcgacga
cgccgctcatgcgcggcactccgtccaccgtctcccacaggacgttctcgccgccgaggtcaccgtggaccaccgcggaggtgagatggggcag
ggcgtcgagcgcggcgagctcgcgctcggcacgctcccggccgccgtcggacatcagcgggaacagttcggtacgcacccccgtggcgaactc
ctgccactcgttcgcgggagcctccggcagcgcggcgcgcaccttctcctcgtcgcccgccgccgcgagcccggacagcagggtcgcgtactgtc
gggcgacggcctccgccacctccgggctggtgagcacatcgtcctccaacggtgctccgggaatgcggctcagcaccaggtacggcggctcgtc
cgtgccctgggcgccgccctcggacagcggctgcggcgtgcgaaacccgaggtcgatcccggcaagagcgcgcaggacgtccgccctgccgg
gcagacggtcggcggccgcccgggtgcgggcgaagcagaccacccggtgcgatccgatcaccacatggtggaactgcccctcgtggacggcg
agtccgcccacggtgtccccgggcaggagccggctcagcagatcgcggtgcgtctcaatgattctcatgacattgcactccaccgctgatgacatc
agtcgatcatagcacgatcaacggcactgttgcaaatagtcggtggtgataaacttatcatcccctttgctgatggagctgcacatgaacccattcaa
aggccggcattttcagcgtgacatcattctgtgggccgtacgctggtactgcaaatacggcatcagttaccgtgagctgcattttccgctgcataaccct
gcttcggggtcattatagcgatttttcggtatatccatcctttttcgcacgatatacaggattttgccaaagggttcgtgtagactttccttggtgtatccaac
ggcgtcagccgggcaggataggtgaagtaggcccacccgcgagcgggtgttccttcttcactgtcccttattcgcacctggcggtgctcaacgggaa
tcctgctctgcgaggctggcgggaacttcgaactccaggtcgacggatccccggaatgtaagcgtcacggcacgcgccgactgagagacgtttcc
gcaggtcaaccccgttccagcccaacagtgttagtctttgctcttacccagttgggcgggatagcctgcccggcatgagcgtgaaggttgaaggcat
ggtcattctggcaggcggctacgaccgacagtcggcggaacgggagaacagttcgaccgcttcaccggccacccagcgcgccgcgaaccggg
ggaaggctgaggcgctggcgaaggagtacgcgcgcgacggcgtcgaggtgaagtggctgggtcacttcagcgaagcgcccggcacgtcggca
ttcacgggcgtcgaccggccggagttcaaccggattttggacatgtgccggaaccgggaaatgaacatgatcattgttcattacatttcgcgcctcag
ccgcgaagagccgctggacattattccggtcgtcacggaattgctccggctgggcgtgaccattgtcagcgtgaacgaaggcacattccgccccgg
cgaaatgatggaccttattcacctgatcatgcgccttcaggcttcgcatgatgagtcgaagaacaagagcgtcgccgtgtcgaacgctaaggaattg
gcgaagcggctgggcggacacacggggtcgacgccgtacggattcgacacggtcgaggaaatggttccgaacccggaagacggcggaaagc
tggttgccattcgccgactggtgcccagcgcgcacacctgggaaggcgcacacggcagcgaaggggcggtaatccgctgggcgtggcaggag
atcaagacgcaccgcgatacgccattcaagggtggcggagccgggtcgtttcaccctgggtcgctgaacgggctttgtgagcggctgtaccgcga
caaggtgcctacgcgcggcacgctggtcggtaagaagcgcgccggttccgattgggaccccggcgttttgaagcgcgtactcagcgacccgcgc
attgccgggtatcaagctgacatcgcatacaaggtgcgcgccgacggttcgcggggcggcttcagccattacaagatcaggcgcgacccggtcac
catggagccgctgaccctgcccggcttcgagccgtacattcccccggcggaatggtgggaacttcaggagtggcttcagggtcgaggacgcggg
aagggtcagtaccgggggcaatcgctcctgtcggcaatggacgtcctttactgctacggctccggccagctcgacccggagacgggttacagcaa
cgggtcgaccatggcgggcaacgtccgcgaaggtgatcaagctcacaagtcgtcgtacgcgtgcaagtgcccccgccgggttcatgacgggtcgt
catgctcgatcacgatgcacaaccttgacccgtacatcgtcggcgcgatcttcgcgcgcatcacggccttcgaccctgccgaccctgacgacctcga
aggcgacacggcagcgctcatgtacgaagccgcacggcgctggggagcgacgcacgaacgcccggagttgaagggtcagcgctccgaactg
atggcacagcgcgcggacgccgtgaaggcgctcgaagagctttacgaagacaagcggaacggcggctaccggtccgccatgggacggcgcg
cgtttctcgaagaggaagccgcgctgacgctccgcatggaaggggccgaagaacggcttcgtcagctcgacgccgccgactcccccgtgctgcc
gatcggcgaatggctgggcgaccggggcagcgacccgacgggaccgggttcgtggtgggcgctagcgccccttgaagaccgtcgggcgttcgt
ccggctcttcgtggaccggatcgaggtgatcaagcttccgaagggcgttcagcggcccggacgggttcccccgatcgccgaccgtgtgcgtatcca
ctgggcgaagccgaaggtcgaggaagagacggagccggagacgctgaacgggttcacagcggcggcgtgacggcggcaccagcgcaacg

ggaaggggcttcggccccttttctcgtgcccggcgtcggttcgttgccctaagcaactgttcctagcgatcggaaagaacatgtgagcaaaaggcca
gcaaaaggccaggaaccgtaaaaaggccgcgttgctggcgtttttccataggctccgcccccctgacgagcatcacaaaaatcgacgctcaagtc
agaggtggcgaaacccgacaggactataaagataccaggcgtttccccctggaagctccctcgtgcgctctcctgttccgaccctgccgcttaccgg
atacctgtccgcctttctcccttcgggaagcgtggcgctttctcatagctcacgctgtaggtatctcagttcggtgtaggtcgttcgctccaagctgggctgt
gtgcacgaaccccccgttcagcccgaccgctgcgccttatccggtaactatcgtcttgagtccaacccggtaagacacgacttatcgccactggcag
cagccactggtaacaggattagcagagcgaggtatgtaggcggtgctacagagttcttgaagtggtggcctaactacggctacactagaagaaca
gtatttggtatctgcgctctgctgaagccagttaccttcggaaaaagagttggtagctcttgatccggcaaacaaaccaccgctggtagcggtggtttttt
tgtttgcaagcagcagattacgcgcagaaaaaaaggatctcaagaagatcctttgatcttttctacggggtctgacgctcagtggaacgaaaactca
cgttaagggattttggtcatgagattatcaaaaaggatcttcacctagatccttttaaattaaaaatgaagttttaaatcaatctaaagtatatatgagtaa
acttggtctgacagttaccaatgcttaatcagtgaggcacctatctcagcgatctgtctatttcgttcatccatagttgcctgactccccgtcgtgtagataa
ctacgatacgggagggcttaccatctggccccagtgctgcaatgataccgcgagacccacgctcaccggctccagatttatcagcaataaaccag
ccagccggaagggccgagcgcagaagtggtcctgcaactttatccgcctccatccagtctattaattgttgccgggaagctagagtaagtagttcgc
cagttaatagtttgcgcaacgttgttgccattgctacaggcatcgtggtgtcacgctcgtcgtttggtatggcttcattcagctccggttcccaacgatcaa
ggcgagttacatgatcccccatgttgtgcaaaaaagcggttagctccttcggtcctccgatcgttgtcagaagtaagttggccgcagtgttatcactcat
ggttatggcagcactgcataattctcttactgtcatgccatccgtaagatgcttttctgtgactggtgagtactcaaccaagtcattctgagaatagtgtatg
cggcgaccgagttgctcttgcccggcgtcaatacgggataataccgcgccacatagcagaactttaaaagtgctcatcattggaaaacgttcttcgg
ggcgaaaactctcaaggatcttaccgctgttgagatccagttcgatgtaacccactcgtgcacccaactgatcttcagcatcttttactttcaccagcgttt
ctgggtgagcaaaaacaggaaggcaaaatgccgcaaaaaagggaataagggcgacacggaaatgttgaatactcatactcttcctttttcaatatt
attgaagcatttatcagggttattgtctcatgagcggatacatatttgaatgtatttagaaaaataaacaaataggggttccgcgcacatttccccgaaa
agtgccacctgacgtc

***snoa123* (codon-optimized)**

gaattcgcggccgcttctagagaaagaggagaaatactagatgaaggagtccatcaaccgtcgcgtcgtcatcaccggcatcggcatcgtggccc
ccgacgccaccggcgtcaagcccttctgggacctgctgaccgccggccggaccgccacccggaccatcaccgccttcgaccccctccccttccg
gtcccgcatcgccgccgagtgcgacttcgacccgctggccgagggcctgaccccgcagcagatccgtcgcatggaccgcgccacccagttcgcc
gtggtctccgcccgggagtccctggaggactccggcctggacctgggcgccctggacgcctcccggaccggcgtcgtcgtcggctccgccgtcgg
ctgcaccacctcgctggaggaagagtacgccgtggtctccgactccggccgcaactggctggtcgacgacggctacgccgtcccccacctgttcg
actacttcgtcccctcctccatcgccgccgaggtcgcccacgaccggatcggcgccgagggcccggtctccctggtctccaccggctgcacctcgg
gcctggacgccgtgggccgggccgccgacctgatcgccgagggcgccgccgacgtcatgctggccggcgccaccgaggcccccatctcccccc
atcaccgtggcctgcttcgacgccatcaaggccaccaccccgcggaacgacaccccggccgaggcctcccgccccttcgaccggacccgcaac
ggcttcgtcctgggcgagggcgccgccgtcttcgtcctggaggagttcgagcacgcccgccggcgcggcgccctggtctacgccgagatcgccgg
cttcgccacccgctgcaacgccttccacatgaccggcctgcggccggacggccgcgagatggccgaggccatcggcgtggccctggcccaggc
cggcaaggcccccgccgacgtggactacgtcaacgcccacggctccggcacccggcagaacgaccggcacgagaccgccgccttcaagcgg
tccctgggcgaccacgcctaccgggtccccgtctcctccatcaagtccatgatcggccactccctgggcgccatcggctccctggagatcgccgcct
ccgtcctggccatcacccacgacgtggtcccgcccaccgccaacctgcacgagcccgacccggagtgcgacctggactacgtcccccctgcgggc
ccgggcctgccccgtcgacaccgtcctgaccgtgggctccggcttcggcggcttccagtccgccatggtcctgtgcggcccgggctcccggggccg
ctccgccgcctgatactagagaaagaggagaaatactagatgaccgccgccgtcgtcgtcaccggcctgggcgtcgtggcccccaccggcctgg
gcgtccgcgagcactggtcctccaccgtccgcggtgcctccgccatcggccccgtcacccgcttcgacgccggccggtacccctccaagctggcc
ggcgaggtccccggcttcgtcccggaggaccacctgccgtcccgcctgatgccgcagaccgaccacatgacccgcctggccctggtcgccgccg
actgggccttccaggacgccgccgtcgacccgtccaagctgcccgagtacggcgtcggcgtcgtcaccgcctcctccgccggcggcttcgagttcg
gccaccgcgagctccagaacctgtggtccctgggcccccagtacgtctccgcctaccagtccttcgcctggttctacgccgtcaacaccggccaggt
gtccatccggcacggcctgcgtggcccgggtggagtcctggtcaccgagcaggccggtggcctggacgcactgggccaggcccgtcgccagctg
cgccggggtctgccgatggtcgtcgccggtgcggtggacggttcccctgcccgtggggctgggtcgcccagctgtcctccggcggcctgtccacct
cggacgacccgcgtcgggcctacctgcccttcgatgccgcggccggtggtcacgtcccgggagagggcggtgccctgctggtcctggagtccgac
gagtccgcgcgggcacggggtgtcacccggtggtacggccggatcgacggctacgccgccaccttcgacccccctccgggctccggccgtccgc
cgaacctgctgcgggcagcccaggcagccctggacgacgccgaggtcggcccggaggccgtggacgtggtcttcgccgacgcctccggcacc
ccggacgaggacgccgccgaggccgatgccgtgcgccggctgttcggcccgtacggcgtcccggtcaccgcccccaagaccatgaccggccg
cctgtccgcgggcggcgcggccctggatgtcgccaccgccctgctcgccctgcgggagggtgtcgtcccgccgaccgtcaacgtctcccgccccc
ggccggagtacgagctggacctggtcctggccccgcgtcgcaccccgctggcccgcgccctggtcctggcccgcggccggggcggcttcaacgc
cgccatggtcgtcgccggcccgcgcgccgagacccggtgatactagagaaagaggagaaatactagatgaagcagcagctgaccaccgagc
gcctgatggagatcatgcgggagtgcgccggctacggcgaggacgtggacgccctgggcgacaccgacggcgccgacttcgccgccctgggct
acgactccctggccctgctggagaccgccggccgcctggagcgggagttcggcatccagctgggcgacgaggtggtggccgacgcccgcaccc
cggccgagctgaccgccctggtcaaccggaccgtcgccgaggccgcctgag

***snoa123* (wildtype)**

gaattcgcggccgcttctagaggaagggcatcttcgacgaatgaaggaatccatcaaccgtcgcgtggtcatcaccggaatagggatcgtcgcgc
ccgatgccaccggggtgaaaccgttctgggatctgctgacggccggtcgcaccgcgacccggaccatcaccgccttcgatccctctccgttccgttc
ccgcatcgccgcggaatgcgatttcgacccgcttgccgaagggctgaccccccagcagatccggcgtatggaccgggccacgcagttcgcggtc
gtcagcgcccgggaaagcctggaggacagcggactcgacctcggcgccctggacgcctcccgcaccggcgtggtcgtcggcagcgcggtcgg
ctgcaccacgagcctggaagaggagtacgcggtcgtcagcgacagcggccggaactggctggtcgacgacggctacgccgtaccgcacctatt
cgactacttcgtgcccagctccatcgccgccgaggtggcacacgaccgcatcggcgcggagggccccgtcagcctcgtgtcgaccgggtgcacc
tcgggcctggacgccgtgggccgcgcggccgacctgatcgccgagggagcggcggatgtgatgctggccggtgcgaccgaggcgcccatctcc
cccatcaccgtggcgtgcttcgatgccatcaaggcgaccaccccccgcaacgacacgcccgccgaggcgtcccgtccgttcgaccgcaccagg
aacgggttcgtactcggcgagggcgctgccgtgttcgtcctggaggagttcgaacacgcgcgccgccggggcgcgctcgtgtacgcggagatcgc
cgggttcgccactcgctgcaacgccttccacatgaccggtctgcgcccggacgggcgggagatggcggaggccatcggggtggcgctcgccca
ggcgggcaaggcgccggctgacgtcgactacgtcaacgcccacggttccggcacccggcagaatgaccgtcacgagacggcggccttcaagc
gcagtctcggcgaccacgcctaccgggtcccggtcagcagcatcaaatccatgatcgggcactcgctgggcgcgatcggctccctggagatcgcc
gcctccgtgctggccatcacacacgacgtggtgccgcccaccgccaatctgcacgagccggatcccgagtgcgatctggactacgtgccgctgcg
ggcgcgtgcgtgcccggtggacacggtgctcacggtgggcagcgggttcggcggtttccagagcgccatggtgctgtgcggtccgggctcgcggg
gaaggtcggccgcgtgacggccgccgtggtggtgaccggtctcggcgtcgtcgcccccaccggtctcggggtgcgggagcactggtcgagtacg
gtccgggggcgtcggcgatcggaccggtcacccggttcgacgccggccggtaccccagcaaactggccggagaggtgcccggtttcgtcccg
gaggaccatctgcccagccggctgatgccgcagacggaccatatgacgcgcctggcgctcgtcgcggcggactgggccttccaggacgccgcc
gtggacccgtcgaagctgccggagtacggcgtcggcgtggtcaccgcgagttcggcgggggggttcgagttcggccaccgcgagctccagaacc
tgtggagcctgggcccgcagtacgtcagcgcgtatcagtcgttcgcatggttctatgccgtgaacaccggtcaggtgtccatccggcacgggctgcg
cggcccgggcggggtgctggtgacggaacaggcgggcggcctggacgcccttgggcaggcccggcggcagttgcggcgcggactgccgatg
gtggtcgcgggagccgttgacggctcgccctgcccctggggctgggtggcgcagctcagctcgggcggcctcagcacgtcggacgacccgcgcc
gggcctatctgccgttcgacgccgcagccggcggacacgtgccgggagagggcggcgccctgctcgtcctggagagcgacgagtcggcccgg
gcgcgcggggtgacgcggtggtacgggcgcatcgatgggtacgccgccacattcgacccccgcccggttcggggcgcccgccgaacctgctg
cgggccgcgcaggcggcactggacgacgcggaggtcggacccgaggcggtcgacgtggtgttcgcggacgcgtccggcaccccggacgagg
acgcggcggaggccgacgcggtgcggcgcctgttcggaccgtacggcgttccggtgacggcgccgaagaccatgaccggccgcctcagcgcg
ggcggcgcggccctcgacgtggcgacggcgctgctggcgctgcgcgagggcgtcgtcccgccgacggtcaacgtctcccggccgcggccgga
gtacgagctggacctggtgctcgcccccccggcgcacgcccctggccagggccctggtgctcgcgcggggccggggcgggttcaatgcggcgatg
gtcgtggcggggccgcgcgcggagacacggtgaagcggcccggcgcagccggagccgcggtaagaggccacggaagagagagggatgcg
acggtgaagcagcagctgacgacggaacggctcatggagatcatgcgggagtgcgcgggctacggtgaggacgtcgacgctctgggcgacac
ggacggcgccgacttcgccgcactcggctacgactcgctggcgctcctggaaacggccggccggctcgagcgcgagttcggcatccagctcggt
gacgaggtggtcgccgacgccaggacgcctgccgagctgaccgccctggtcaaccggacggtggccgaggcggcctgatactagtagcggcc
gctgcag

dpsABCDG (codon-optimized)

gaattcgcggccgcttctagagaaagaggagaaatactagatgaaccgtcgtatcgtgattaccggtattggtgttgttgcgccgggtgcggttggta
ccaagccgttctgggaactgctgctgagcggtaccaccgcgacccgtgcgatcagcaccttcgacgcgaccccgtttcgtagccgtattgcggcgg
agtgcgactttgatccggttgcggcgggtctgagcgcggaacaagcgcgtcgtctggaccgtgcgggccagtttgcgctggttgcgggtcaagagg
cgctggcggatagcggtctgcgtattgacgaagatagcgcgcaccgtgtgggtgtttgcgtgggcaccgcggtgggttgcacccaaaaactggag
agcgaatacgttgcgctgagcgcgggtggcgcgcactgggtggttgacccgggtcgtggcagcccggaactgtacgattatttcgtgccgagcagc
ctggcggcggaagtggcgtggctggcgggtgcggaaggcccggttaacatcgtgagcgcgggctgcaccagcggtatcgacagcattggctatg
cgtgcgagctgattcgtgaaggtaccgttgatgcgatggttgcgggtggcgttgatgcgccgattgcgccgattaccgtggcgtgctttgatgcgatcc
gtgcgaccagcgaccacaacgataccccggaaaccgcgagccgtccgttcagccgtagccgtaacggctttgtgctgggcgagggtggcgcgat
tgtggttctggaggaagcggaagcggcggttcgtcgtggcgcgcgtatctacgcggaaattggtggctatgcgagccgtggtaacgcgtaccacat
gaccggtctgcgtgcggatggtgcggagatggctgcggcgattaccgcggcgctggacgaagcgcgtcgtgatccgagcgacgttgattacgtga
acgcgcacggtaccgcgaccaagcagaacgatcgtcacgagaccagcgcgtttaaacgtagcctgggcgaacacgcgtatcgtgtgccgatca
gcagcattaagagcatgattggtcacagcctgggtgcggtgggtagcctggaagtggcggcgaccgcgctggcggtggaatacggtgttattccgc
cgaccgcgaacctgcacgacccggatccggagctggacctggattatgttccgctgaccgcgcgtgaaaaacgtgtgcgtcacgcgctgaccgtt
ggtagcggcttcggtggctttcaaagcgcgatgctgctgagccgtctggagcgttgatactagagaaagaggagaaatactagatgaccggtaccg
cggcgcgtaccgcgagcagccagctgcacgcgagcccggcgggtcgtcgtggtctgcgtggtcgtgcggtggttaccggtctgggtattgtggcgc
cgaacggtctgggcgttggtgcgtactgggatgcggtgctgaacggccgtaacggcattggtccgctgcgtcgtttcaccggtgatggtcgtctgggtc
gtctggcgggcgaggtgtctgattttgttccggaagaccacctgccgaagcgtctgctggcgcagaccgatccgatgacccaatatgcgctggcgg
cggcggagtgggcgctgcgtgaaagcggttgcagcccgagcagcccgctggaggcgggcgttattaccgcgagcgcgagcggtggcttcgcgtt
tggtcagcgtgaactgcaaaacctgtggagcaaaggcccggcgcacgttagcgcgtacatgagcttcgcgtggttttatgcggtgaacaccggtca
gatcgcgattcgtcacgatctgcgtggtccggttggcgtggttgtggcggagcaagcgggtggcctggacgcgctggcgcatgcgcgtcgtaaagt

gcgtggtggcgcggaactgattgtgagcggtgcggttgatagcagcctgtgcccgtatggtatggcggcgcaagtgaaaagcggtcgtctgagcgg
cagcgataacccgaccgcgggttatctgccgtttgaccgtcgtgcggcgggtcatgttccgggtgagggtggcgcgattctgaccgttgaagatgcg
gaacgtgcggcggaacgtggtgcgaaggtttacggcagcattgcgggctatggtgcgagctttgacccgccgccgggtagcggtcgtccgagcgc
gctggcgcgtgcggtggagaccgcgctggcggacgcgggtctggatggcagcgacatcgcggttgtgttcgcggatggtgcggcggttccggaac
tggatgcggcggaggcggaagcgctggcgagcgtgtttggtccgcgtcgtgttccggtgaccgttccgaaaaccctgaccggccgtctgtacagcg
gtgcgggtccgctggatgttgcgaccgcgctgctggcgctgcgtgatgaggttgttccggcgaccgcgcatgtggacccggatccggacctgccgct
ggatgttgtgaccggtcgtccgcgtagcctggcggatgcgcgtgcggcgctgctggttgcgcgtggttatggtggcttcaacagcgcgctggttgtgcg
tggcgcggcgtgatactagagagaaagaggagaaatactagatgagcgtgccgcagggtgcgccgggtgacctgtacgttgcgggttgcggtgtt
tggctgccgccgccggttaccaccgagcaagcgctggcggcgggtcattgcgaccgtcgtctggcgagcagcacccgtatgctgagcgtggcggt
tgcggataaggagaccccggcggaaatggcggcgctggcggcgcagaccgcgctggaccgtagcggtgtggcgccggcgcatgtggatctggt
tctgcacgcgagcctgtactttcaaggtcaccacctgtgggcgccgagcagctatgttcaacgtgtggcggttggtaaccgttgcccggcgatggaa
gtgcgtcaagttagcaacggtggcatggcggcgctggaactggcgcgtgcgtacctgctggcggcgccggaccgtgtggcggcgctgattaccac
cggtgatcgtatgcacccgccgggttttgaccgttggagcagcgatccgggtaccgtgtatgcggatggtggcaccgcgctggttctgagccgtcag
ggtggcttcgcgcgtctgcgtagcctggtgaccgttagcgagccggttctggaaggcatgcatcgtggtggccacccgtttggtccgccgagcccgg
aggaacagcgtgcggtggacctggatgcgcacaaacgtgcgtatgttgcggaggcgggtagcagctttagcgtggcgcgtgttagcgcgggcca
agaggaagcgctgaccggtgcgctggaagcggcgggtgcgggtctggacgatattagccgtgtggttctgccgcacatgggttggcgtcgtctgag
cgcggcgtatttcaacaaatggcacatccaaccggagcgtaccacctgggaatttggtcgtcgtaccggtcatctgggtggcggtgacccgattgcg
ggtttcgatcatctggtgggtagcggtcgtctggcgccgggtgaactgtgcctgctggtgagcgttggcgcgggttttagctggagctgcgcggtggttg
agctgctggaacgtccgagctgggctgcggcgccggctgcgcgttgatactagagaaagaggagaaatactagatggatgcgccggcgatcctg
ctgatgtttccgggtcagggtagccaacatgcgcgtatggcggttggtctgtatggtagcgagccggcgttcaccgcggcgatggacgaagtgtttga
tgcgtatgacgatgcgggtgatagcgagggtggccgtctgcgtgcggactggctgagcgatcatccggcggttccgctggaccatgtgacccgtag
ccagccgctgctgttcgcggttgattacgcgctgggtcgtatggtgatggcgaccaccggtcgtccgtgggcgctgctgggtcacagcgtgggcgag
atggctgcggcggcgctggcgggtgtgtttaccctgcgtgatgcggcgggtctgctgctggatcgtgttcgtcgtctggcgcaggcgccgccgggtgg
catgctggcggttgcggcggcggaggacgtgctgggtccgtatctgggtaacggcgaagtggttgtggcggcggttaacagcccgcgtcaaacca
tcctggcgggtccggttgcgagcctggcgggcgtggcgcgtgcgctgcgtgaggcgggtctgacctgccgtgaagtgccgagcctgaccgcgttcc
acagcccgatgctggcgccgattgcggcgggtgcggcggagcgttttgcgagcacccgtgttgcgccgagcattccgctgcgtagcggttaccgtc
cgggtgcggtgaccggtcagctggcggcggacccgagctattgggcgggtcacccgctggagaccgttcgtttctggccggcgctggatgcgatg
ctgggtagccgtaccggcgttgtgtgcctggaaaccggtccgggccaaggtctgagcaccattgcgcgtcgtcacccgaccgtgcgttttggcaaga
acagcgttgttccgctgctgccgccgcaggcgggtaccgcggaagtggatcgtaccagcgtggctgcggcgctggcggcggttcgtgaacaaggt
accgtgctgagcggccgtccgggtcgttgagaaagaggagaaatactagatggcggaactgagcctggcggaactgcgtgagatcatgcgtcag
agcctgggtgaagacgaggtgccggatctggcggacgcggataccgttaccttcgaggacctgggtctggatagcctggcggtgctggaaaccgt
taaccacatcgagcgtacctacggcgtgaagctgccggaggaagagctggcggaagttcgtaccccgcacagcatgctgatttttgtgaacgagc
gtctgcgtgctgcggcgtgatactagtagcggccgctgcag

dpsABCDG **(wildtype)**

gaattcgcggccgcttctagagggagggcggacgacgtgaaccggcggatcgtcatcaccgggatcggcgtggtggctcccggcgccgtcggg
accaagccgttctgggagctgctgctgtccggaaccaccgccacccgcgccatcagcactttcgacgccacgcccttccgttcccgtatcgccgcc
gagtgcgacttcgatccggtggcggcggggctgtccgcggagcaggcccggcgcctcgaccgggccgggcagttcgcgctggtcgccggtcag
gaggcgctcgccgacagcgggctgcgaatcgatgaggactccgcgcaccgggtcggggtgtgcgtgggcaccgccgtcggctgcacgcagaa
actggagtccgagtacgtcgctctcagcgcgggcggagcgcactgggtcgtggaccccggccgggggtcgcccgagctgtacgactacttcgtgc
cgagttccctcgccgccgaagtggcctggctcgccggggcggaggggcccgtgaacatcgtctcggccgggtgcacctcggggatcgactccatc
gggtacgcctgcgagctcatccgcgaggggacggtggacgccatggtcgcgggaggcgtggacgcacccatcgcgccgatcacggtggcctgc
ttcgacgccatcagggcgacctccgaccacaacgacacccccgagaccgcctcgcgtcccttcagccgcagccgcaacggctttgtgctgggcg
agggcggtgcgatcgtggtcctggaagaggcggaggcggccgtgcgcaggggcgcgcggatctacgccgagatcggcgggtacgcctcacgc
ggcaacgcgtaccacatgaccgggctgcgcgcagacggcgccgagatggcggcggcgatcaccgcggccctcgacgaggcccgccgcgac
ccctcggacgtggactacgtcaacgcccacggaacggcgacgaagcagaacgaccggcacgagacctccgcgttcaagcgctccctgggggg
agcacgcgtaccgggtgcccatcagctccatcaagtcgatgatcggtcactccctgggcgctgtcggctcgctcgaagtggcggcgaccgcactg
gccgtggagtacggggtgatcccgccgaccgcgaatctgcacgacccggatccggaactcgacctggactacgtgccgctgacggcacgagaa
aagcgggtgcggcacgcgctgacggtgggcagcggcttcggcggcttccagagcgccatgctgctgagccgtctcgagaggtgagctccatgac
aggtaccgcggcacggaccgcgtcttcgcagctccacgcatcgcccgcgggccgacggggcctcaggggccgcgcggtggtgacgggtctcgg
gatcgtcgcccccaacgggctgggggtgggcgcgtattgggacgccgtcctgaacggccgcaacggcatcggaccgctgcggcggttcaccgg
cgacggccgcctgggccggctggccggtgaggtgagcgacttcgtccccgaggaccatctgccgaagcggctgctggcgcagaccgatccgat
gacccagtacgcgctggccgccgcggagtgggccctgcgggagtccggctgctccccgtcctcgccgctggaggcgggggtgatcacggccag
cgcgtccggcggcttcgccttcggccagcgggagttgcagaatctttggagcaagggaccggcccacgtcagcgcctacatgtcgttcgcctggttc
tacgccgtcaacaccgggcagatcgccatccggcacgatctgcgcggcccggtcggcgtggtggtggccgagcaggcgggcggcctcgacgcc

ctcgcccacgctcgccgcaaggtccggggcggagccgagctgatcgtctcgggcgccgtggactcgtcgctgtgcccgtacggcatggcggccc
aggtgaagtccggccggctgagcggctcggacaacccgacagccgggtatctgccgttcgaccggcgggcggccggacatgtgcccggcgag
ggaggcgcgatcctcaccgtcgaggacgcggagcgggccgccgagcggggcgcgaaagtgtacgggtcgatcgccgggtacggcgcgagct
tcgacccgccgcccggctccggccgcccctccgccctcgcccgtgccgtcgagacggcgctggccgacgccgggctggacggctccgacatag
ccgtggtgttcgccgacggcgcggccgtccccgaactggacgcagccgaggcggaggctctggcgtcggtcttcgggccgcgccgggtcccggt
gaccgtccccaagactctcaccggccggctgtattcgggcgccgggccgctggacgtggcgaccgcgctgctggcgctgcgggacgaggtggtc
ccggcgaccgcccacgtcgaccccgacccggacctgccgctcgacgtggtgaccgggcggccccggtccctggccgacgccagggcggccct
cctcgtcgcccgcggctacgggggcttcaacagcgctctggtggtccgggggcggcgtgagcgtgccgcagggggcgcccggggacctttacg
tggccggttgcggcgtctggctgcccccgccggtgaccacggaacaggccctggccgccggccactgcgaccgcaggctcgcctcctcgacgcg
gatgctgtcggtggcggtcgccgacaaggagacaccggccgaaatggcggccttggcggcccagaccgctctggaccgctccggcgtcgcacc
ggcccacgtcgacctcgtcctgcacgccagtctgtacttccaggggcaccatctgtgggctccctcctcctacgtccagcgggtggccgtcgggaac
cgctgccccgccatggaggtgcggcaggtgtccaacggcggcatggccgccctggaactggcccgcgcgtacctgcttgccgccccggaccgg
gtggccgctctcatcacgacaggcgaccggatgcatccgccgggcttcgaccgctggagcagcgaccccggcacggtgtacgccgacggtggt
accgccctcgtgctgtcccggcagggcggtttcgcccggctgcgcagcctggtgacggtctccgaaccggtgctggagggcatgcaccggggcg
ggcacccgttcggcccgccgtctccggaggagcagcgggccgtggacctggacgcgcacaagcgggcgtacgtggccgaggcgggaagctc
gttcagcgtcgcccgggtctcggcgggccaggaggaggctctgaccggcgcgctggaggcggccggggccggactcgacgacatcagccggg
tcgtgctgccgcacatggggtggcgccggctgagcgccgcctacttcaacaagtggcacatccaacccgagcggaccacttgggagttcgggcg
gcggaccggccacctgggggcggtgacccgatcgccgggttcgaccaccttgtgggctccggccgcctcgccccgggggagctgtgtctgctgg
tcagcgtcggcgccggcttcagctggtcgtgcgccgtcgtggagttgctggaacgcccgtcgtgggcggccgccccggccgcccggtgaggagcc
cgtcatggatgcgcccgcgattctcctgatgttccccgggcaggggtcccagcatgcgcggatggccgtcggcctgtacggatccgaaccggccttc
acggccgccatggacgaggtcttcgacgcgtacgacgacgccggcgacagtgagggcgggcggctgcgtgccgactggctgagcgaccatcc
ggccgtgccattggaccacgtcacccggtcccagccgttgttgttcgcggtggactacgccctcggccggatggtgatggccacgacgggccggcc
gtgggccctgctcgggcacagcgtcggcgaaatggcggcggccgcgctcgccggggtgttcacgctgcgcgacgcggccggcctcctgctggac
cgggtgcgacggctggcccaggcaccgcccgggggaatgctggcggtcgccgcggccgaggacgtgctgggcccgtatctcgggaatgggga
ggtggtggtcgcggccgtcaactcgccccgccagaccatcctcgccggacccgtcgcatcgctcgccggggtcgcccgggcgctgcgggaggc
agggcttacgtgccgtgaggtgccctcgctgaccgccttccacagtccgatgctggccccgtacgcggcgggggcggccgagcggttcgcatcca
cgcgcgtggcggcgccgtccatccccctccgctccgggtaccggccgggtgcggtgaccgggcaactggccgccgacccgtcgtactgggccgg
gcatcccctggagacggtccgcttctggccggcactggacgcgatgctcggctcccgcacgggcgtcgtctgcctggagacggggccggggcag
gggctgagcaccatcgcccgccgccatccgacggtgcggttcgggaagaactcggtggtgccgctgctgccgccgcaggccggcaccgccgag
gtggaccggacgtccgtggcggcggcgctggccgcggtgcgcgagcagggcaccgtgctcagtgggcgacccgggcggtgagaggagcagg
gatggctgagctcagcctggcggaactgcgggagatcatgcggcagagcctgggggaggacgaggtccccgaccttgcggacgcggacaccg
tgaccttcgaggacctcgggctcgactccctggccgtcctggaaacggtcaaccacatcgagcggacctatggcgtgaagctgcccgaggagga
actggcggaggtcaggacgccgcatagcatgctgatcttcgtcaacgagaggctgcgagcggcggcatgatactagtagcggccgctgcag

***aknBCDE2F* (codon-optimized)**

gaattcgcggccgcttctagagaaagaggagaaatactagatgaccgcccgtcgcgtggtcatcaccggcctgggcgtcatcgccccgggtggc
atcggcaccaaggccttctgggagcggatcgtctccggcgtctccgccacccgcaccatcaccgccttcgacgcctccgagttccgctcccggatg
gccgccgagtgcgacttcgacggcgtccgctccggcctgaccgtccgggacaccgcccgcctggaccggccacccagttcgccgtggtggccg
cccgcgaggccctggccgactccggcatcgagatcgacgagcgcaacgcccaccggaccggcgtctccctgggctccgccgtcggctgcaccc
agaagctggaggaagagtacgtggcccgctccgacggtggccagcggtggctcgtggaccacgccgccggcaccccgtacctgtacgactactt
cgtcccgtcctcgatggccgccgaggtcgcctgggaggccggcgccgagggcccggccgccctggtctccgccggctgcacctcgggcctggac
tccctgggccacgccctggacctgatccgcgagggcgccgtggacatcatgatcgccggcggctccgacgcccccatcgcccccatcaccgtgg
cctgcttcgacgccatcaaggccacctcgccgcgcaacgacaccccggagcacgcctcccggccgttcgaccgcacccggtccggcttcgtcctg
ggcgagggcgccgccgtcctggtcctggaggagcgggagtccgccctgcgccgcggtgcccaaatctacgccgagatcgccggctacgccggc
cgcgccaacgcccaccacatgaccggcctgcggcccgacggcctggagatgtccgccgccatcaccggcgccctggacgacgcccgcatcga
ccgggaggccgtgggctacgtcaacgcccacggcaccgcgacccgccagaacgacatccacgagaccgccgccatcaagcactccctgggc
gagcacgcccgccgggtcccggtctcctccatcaaggccgtcatcggccactccctgggcgccgtgggctccatcgaggccgtcgcctccgccct
ggtcatccgccacggcgtcgtcccgcccaccgccggcctgcacgagccggacccgcagctggacctggactacgtcccccctgatcgcccggga
ccaggccaccgacaccgtcctgaccgtgggctccggcttcggcggcttccagtccgcgatggtcctgacctcggccgagggcggccggtcctgat
actagagaaagaggagaaatactagatgtccgccgccaccgtggtcaccggcatcggcgtcctggccccgaacggcatcggcgccgaggagtt
ctgggccgccaccctgcgggccgagtccggcatcggccggatcacccacttcgagcccgcctcctacccctcccggctggccggcgaggtcacc
ggcttctccgcccgcgagcacctgccctcccggctggtcccgcagaccgaccggaccacccagttcgccctgaccggctccgagtgggccctgcg
ggactccggcctgtccgccgacaccctgccggccggtgagcgcggcgtcgtcaccgcctccgcctccggcggcttcgagttcggccagcgggag
ctgggccacctgtggggcaaggacccgcgccacgtctccgcctacatgtccttcgcctggttctacgccgtcaactccggccagatcagcatccgcc
acgacctgcggggcccgaccggcgtcctggtcaccgaccaggccggcggcctggacgccgtggcccaggcccgccggcgcatccgcaaggg

caccccggtcatgctgtccggcggcatggacgcctccctgtgcccgtacggcctggtcgcccagatcagcgccggcatgctgtccgagtccgacg
accccacccgcgcctaccggccgttcgaccccgccgccgacggccacgtcccgggcgagggcggcgccatcctgaccctggaggacggcga
ccgcgcccgcgcccgcggtgcccggtcccacggcgagatcagcggctacgccgccaccttcgacccgcgcccgggctccggccggcccgcca
acctggaccgcgccatccgcggtgccctggccgacgccggcctgtccccgcgcgacatcgccttcgtcctggccgacggcgccggcgagcccg
agccggaccgcgccgaggcccgtgccctgaccgacgtcttcggcccgcgcggcgtccccgtcaccgtcccgaagtccatgaccggccggctgta
cgccggcgccgccccgctggacctggtcaccgccctgttcgccctgcgggacggcgtcgtcccgcccaccgtccacgtggacgagccggacccc
gcctacgacatcgacctggtcaccggctccgcccgccccgtccggggcgacgccgccctggtcctggcccgcggccggggcggcttcaactccg
cgatggtcgtccgtcgcccgccggccgcctgatactagagaaagaggagaaatactagatgtccgccttcaccgtcgaggagctgttccagatcat
gcgcgagtgcgccggcgaggaagaggccgtggacctggccgacgccgccgagcaggagttcgccctgctgggctacgactccctggccctgat
ggaggccatctcccgcgtcgagcggggcctgggcatcgccctgccggaggagaccgtgggcgaggtcctgaccccggccgccttcgtggacgt
ggtcaacgccgagctggcccggtccgccccggtcgtcgaggccgccggttgatactagagaaagaggagaaatactagatgaccgaggagca
cctggacccggccggcggcgccccgctggcccaggccccggcccaggacatccgcatcgccggctgcgccgtctggctgccgccccgggccc
ccgtcgcccaggccgtcgccgccggtctgtgcgacgaggccctggccaccgccaccgcgatggtctccgtcgccgtcgcccaggacgagccgg
cccccgagatggccgcccgtgccgcccgcaccgccctggcccgcggcggctccgacgacgtctccctgatcctgcacgcctccttcttctaccagg
gccacgacctgtgggcccccgcctcctacgtccagcgcgtggccgtgggcaaccactgcccggccatcgaggtgggccaggtctccaacggtgg
catggccgccctgggcctggccgtggaccacctgtccgccggccgcccggccggcgccgccggtcgccgcgtcctggtcaccaccggcgacgc
cttccgtccgccgggcttcgaccgctggcggtccgaccccggcaccttctacggcgacggcggcaccgccctggtcctgtcctcccaggaaggctt
cgcccgcatccggggcctggccaccgtctccgcccccgagctggagggcatgcaccgcggcgacgaccccttcggctccgccccgttctcccac
cggccggtggtggacctggaggcctgcaagaaggacttcctggcctcccgccgggtcacccaggtcatcgccgcctccgccgccgcccaggac
gccgccctgggccaggccctggccgccgccggtgccgagctggccgacatcgaccgcttcgtcctgccgcacatgggccgcaagcggctgcgc
gccggcttcctgaaccgcctgggcatcggcgaggaccgcaccacctgggagtggtcccggggcgtcggccacctgggcgccggcgaccagatc
gccggcttcgaccacctggtgggctccggctccctgggccccggcgacctggtcctgtggatgtccgtgggcgccggcttcacctactcctgcgccgt
cgtcgagatgctggagcgccccggctgggccgccaccgccggcaccgccggcgccgcctgatactagagaaagaggagaaatactagatgac
cggcaccgccggcgccctgcccgtggccctgctgctgcccggccagggctcccagcaccgtcgcatggccgccggtctgtacggccacgagccc
gtcttcaccgaggcgatggacgagttcttcgacgccgccggtcccgagggcgacccgctgcgcgacgactggctggccgagcggcccgtcaccg
acatcgaccacgtcacccgctcccagcccctgctgttcgccgtggaccacgccctgggccggctggtcctgggccgcggcgtccggccggccgc
actgctgggccactccatcggcgagctggccgccgcaaccctggccggcgtcttcgccccgcgcgacgccgccggcctggtcctggaccggatc
cgccggctgtccgccgccccgcccggcggcatgctggccgtcgccgcctccaccgccgaggtcgcccccctacctgcgcggcgacgtcgtcgtcg
gcgccgtcaacgccccgcgtcagaccgtcctggccggcccggacggcccccctggacgaggtggaccgcgccctgcgggaggccggcttcgtct
gccgccgggtcccctccctgtccgccttccactcccccgtcctggagccggcctgccgcggcgccgccccgctgttcgccgccgcatgcaagcacc
cgcccgccgtcccggtccactccgcctacaccgccgccccgctgaccgagtccgacatcgacgacccggccttctgggcccgccagccggtcgc
cccgtcctgttctggccggccctggagggcctgctggccaccggcgaccacctgctggtcgaggtcggccccggccagggcctgtcccagctggt
ccgccggcacccggccgtccgccggggcggctccgccgtcgtctccctgctgcccgcccgccccggtccgccggaggccgaccggccgccgt
cgccgccgcaaccgagcagatcaccgccgccggccgccaggccgccccggcctccgccgaccacggccgcccctcccggcaggccgccgc
cggttgatactagtagcggccgctgcag

aknBCDE2F (wildtype)

gaattcgcggccgcttctagaggaaggggaggagatatgacggcccgccgggtggtgatcaccggcctgggagtgatcgccccgggtggcatcg
gcaccaaggcgttctgggaacgcgtcgtctccggcgtctccgcgaccaggaccatcacggccttcgacgcgtccgggttccgctcccggatggcc
gcggagtgcgacttcgacggcgtccgctcgggcctgaccgtccgtgacaccgcacggctggaccgtgccacccagttcgcggtggtcgccgcgc
gcgaggcgctcgccgacagcggcatcgagatcgacgagaggaacgcccaccgcaccggggtcagcctcggctccgccgtggggtgcaccca
gaagctcgaggaggagtacgtcgcgcgcagtgacggcgggcagcggtggctggtcgatcacgccgccggcacaccgtacctgtacgactacttc
gtgccgagttcgatggcggccgaagtggcgtgggaggccggcgccgagggccccgccgcgctcgtctcggcgggctgcacctccggtctcgact
ccctcgggcacgcgctcgacctcatcagggagggcaccgtcgacatcatgatcgccggtggcagcgacgcgcccatcgcacccatcaccgtgg
cctgtttcgacgccatcaaggcgacctcgccgcgcaacgacaccccggagcacgcctcgcggcccttcgacaggacccgcagcggcttcgtgct
cggcgagggtgcggccgtactcgtcctggaggagcgggagtccgccctgcggcgcggagcacagatctacgccgagatcgcgggctacgccg
ggcgtgccaacgcccaccacatgaccggactgcgccccgacggactggagatgtccgccgccatcaccggcgccctcgacgacgcccgcatc
gaccgcgaagccgtcggttacgtcaacgcgcacggtacggccacccggcagaacgacatccacgagacggccgcgatcaagcacagcctgg
gcgagcacgcccaccgggtcccggtcagctccatcaaggcggtcatcggtcactcgctcggcgccgtcggctccatcgaggccgtcgcctccgcc
ctggtgatccggcacggagtggtgccgcccacggcgggactgcacgagcccgatccccagctcgacctcgactacgttccgctgaccgcgcgcg
atcaggccaccgacaccgtgctgacggtgggcagcggattcggcggattccagagcgccatggtgctcaccagcgccgaagggagccgctcat
gagtgccgcgaccgtggtcaccggaatcggtgtgctcgcgcccaacgggatcggggcggaggagttctgggcggccaccctgcgcgccgagag
cggcatcggccgcatcacccgcttcgagcccgcctcgtatccctcccggctggccggcgaggtcacggggttctcggcacgggaccacataccga
gccggctcgtcccgcagaccgaccgcaccacgcagttcgccctgacgggctccgagtgggcactgcgggactccggactgtccgccgacagcct
gccggcggacgagcgcggggtggtcaccgccagcgcctccggcgggttcgagttcggccagcgcgaactgggccatctgtggggcaaggacc

cccggcacgtcagcgcgtacatgtccttcgcctggttctacgccgtcaactccggtcagatctccatccggcacgatctgcgaggccccaccggagt
gctcgtcaccgaccaggcgggcggtctcgacgcggtggcacaggccaggcgccggatccgcaagggcaccccggtcatgctgtccggcggcat
ggacgcctcgctgtgcccctacgggctcgcggcccagatctccgccggaatgctcagcgagagcgacgacccggcccgcgcctaccggccgttc
gacccggcggccgacggccatgtgccgggcgagggcggggcgatcctgaccctggaggacggggaacgggcgcgggcgcgcggggcacg
cagccacggggagatcagcgggtacgccgccaccttcgaccccgtcccggctcgggccggcccgccaacctggaccgggcgatccggggcg
cgctggccgacgccgggctgaccccgcgggacatcgccttcgtcctcgcggacggcgccggtgaaccggaacccgaccgggccgaggcgcg
ggcgctcaccgacgtcttcggcccccggggcgtacccgtgacggtccccaagagcatgacggggcgcctctacgcgggtgccgccccgctcgat
ctcgtcaccgccctgttcgcactgcgcgacggcgtcgtcccgccgacggtccacgtcgacggaccggaccccgcctacgacatcgacctcgtgac
cggctccgcccggcccgtccgcggagacgcggcgctggtcctggcccggggacgcggcggcttcaacagcgccatggtcgtacggcgcccgtc
ggcggcctgagccgtcaccccggcggccgaggccgccgccatccgctcacccggccggccagtcggcggtcggcccccacaccgcggcccgt
gcggccgcctttggagacgtcatgtccgctttcacggtagaagagcttttccagatcatgcgcgagtgcgcgggcgaggaggaggccgtcgatctg
gccgacgccgcggagcaggagttctccctgctcggctacgactccctcgcgctcatggaggcgatcagcagggtggagcggggtctcggcatcg
acctgcccgaggagacggtgggcgaggtgctgacgcccgccgcgttcgtcgacgtcgtcaacgccgaactggcccgcagcgcgcccgtggtgg
aggccgccggatgaccgaggagcacctcgaccccgcgggcggcgctccccccgcgcgggccccggagcaggacatccggatcgcggggtg
cgcagcctggctgccgccccgcgtgccggtcgcgaaggcggtggctgcgggactgtgcgacgaggccctggccacggccaccgccatggtctc
ggtggccgtggcgcaggacgagccggcccccgagatggcggcgagggcggcccggaccgccctcgcgcgcggcggctccgacgacgtgtcg
ctgatcctgcacgccagcttcttctaccaggggcacgacctgtgggcgcccgcctcctacgtccagcgcgtcgccgtcggcaaccggtgccccgcg
atcgaggtcggccaggtgtccaacggcggcatggccgccctcggcctcgccgtcgaccacctgagcgccgggcggccggccgaggcgggccg
gcgcgtcctggtgacgaccggtgacgccttccgcccgcccggcttcgaccgctggcgcagcgatccgggcaccttctacggggacgggggcacc
gcgctggtgctgtcctcgggggaggggttcgcccggatccgcggactggccaccgtctccgctccggagctggaggggatgcaccgcggtgacg
acccccttcggggacacaccgttcagccaccgtccggtcgtcgacctggaagcctgcaagaaggacttcctcgcctctcgccgggtgacccaggtg
atcgccgcgagcgcggccgcgcaggacgcagccctcggccaggccctcgcggcggccggggccggactcgcggacatcgaccgcttcgtcctt
ccccacatggccgcaagcggttgcgggccggcttcctcaaccgcctcgggatcggggaggaccgcaccacctgggagtggagccgcggtgtc
gggcacctgggggccggtgaccagatcgccggcttcgaccacctggtcggctccgggagcctcggccccggcgacctggtcctgtggatgagcg
tgggcgccgggttcacctactcctgcgcggtcgtcgagatgctggaacggcccggctgggccggtacggcgcgtacggcaggtgccgcgtgacc
ggcaccgcgggggccctgccggtcgccctgctgctgccgggccagggctcccagcaccggcggatggccgcggggctctacgggcacgagcc
ggtgttcaccgaggccatggacgagttcttcgacgcggccggccccgagggcgatccgctgcgcgacgactggctggccgaacggcccgtcac
ggacatcgaccacgtcacccgctcacagccgctgctcttcgccgtggaccacgcgctggggcggctggtgctcggccggggtgtgcgcccggcg
gccctcctggggcacagtatcggggagctggcggcggccacgctggccggggtgttcgctccgcgggacgccgccggcctggtgctcgaccgg
atacggcggctgtcggcggctccgccgggcggcatgctcgcggtggccgcgtccacggcggaggtggccccgtacctgaggggagacgtcgtg
gtcggcgcggtcaacgccccgcggcagaccgtgctcgccggacccgacggcccgctggacgaggtggaccgtgccctgcgggaggccgggtt
cgtctgccgccgggtgccgtcgctgagcgccttccacagcccggtgctcgaacccgcctgccgggggcggcccctctcttcgccgcggcccgca
agcatccgccggccgtgccggtccactccgcctacaccgccgccccgctgacggagtcagacatcgacgaccccgccttctgggcgcggcagc
cggtggcgccggtgctgttctggcccgctctggaagggctgctggccaccggcgaccatctgctggtggaggtggggccgggacaagggctgtcc
cagctggtccgccgtcacccggctgtgcgccggggcggcagtgccgtggtgtcgctgcttccggcgcggccggggccgccggaggcggaccgg
gccgcggtggccgccgccacggagcagatcacggcggcggggcgccaggccgcccccgcgagcgcggaccacggccggccgtcacggca
ggccgcggccggctgatactagtagcggccgctgcag

***oxyABCD* (codon-optimized)**

gaattcgcggccgcttctagagaaagaggagaaatactagatgtccaagatccacgacgcccgtcgcgtggtcatcaccggcatcggcgtggtgg
cccccggcgacgtcggcaccaagccgttctgggagatgctgaccgccggccggaccgccacccggcccatctcctccttcgacgcctccccttc
cggtcccaggtggccgccgagtgcgacttcgacccggccgccgagggcctgtcccagcggcaggtgcgggcctgggaccggaccatgcagttc
gcctacgtggccgcccgggaggccctggccgactccggcgtcaccggcgaggccgacccgctgcgcaccggcgtcatggccggcaccgcctg
cggcatgaccatgtccctggaccgggagtacgccgtggtctccgacgagggccgcctgtggcaggtcgacgacgcccacggcgtcccgtacctgt
acgactacttcgtgccgtcctccatggccgccgagatcgcctggctggccgaggccgagggcccggccggcgtcgtctccgccggctgcacctcg
ggcatcgacgtcctgacccacgccgccgacctggtccgggacggcgccgccgaggtcatggtcgccggcgcctccgacgccgccatctcccc
atcaccgtggcctgcttcgacgccatcaaggccaccaccccgcggaacgacgagccggagaccgcctcccgccccttcgaccggacccggaa
cggcttcgtcctgggcgagggcgccgccttcttcgtcctggaggagtacgcccacgcccgccgccgcggtgcccgggcctacgccgagatcgccg
gctacgccggccgctgcaacgcctactccatgaccggcctgcggtccgacggccgggagctggccgaggccgtctcccgggccctggacatcgc
ccgcgtcgacccgtccgaggtcgactacgtcaacgcccacggctccgccaccaagcagaacgacctgcacgagaccgccgccttcaagcgctc
cctgggcccccacgcctactccgtccccatctcctccatcaagtccatgatcggccactccctgggcgccatctgcgccctcgaggtcgccgcctccg
ccctgcggatcgagcacggcgtcatcccgcccaccgccaacctgcgcgagcccgaccccgactgcgacctggactacgtcccgctggtcgcccg
ggaggccgaggtctccaccgtggtctccgtcgcctccggcttcggcggcttccagtccgccatcgtcctgaccgagcccggccggcagcgctgata
ctagagaaagaggagaaatactagatgaccggccagctggccccggcccccgagaccggcaccggccgccccggcggctccgtccggcccg
tcgtcaccggcctgggcgtcgtcgccccgaacggcctgggcaccgagcgctactgggccgccaccctgcggggcgactccggcatcggccgcat

cacccggttcgacccgtccggctacacctcgtccctggccggcgagatcgccgacttcgaccccgcccgcctgcccaaccggctgctgccccaga
ccgacctgatgacccgcctggccctggtcgccgccgaggaagccctggacgacgccggcgccgacccgcggaccatgcccgacttcgccgcc
ggcgtcgtcaccgccgcctccgccggcggcttcgacttcggccagcgggagctggaggccctgtggtccaagggcggcgcccacgtctccgcct
accagtccttcgcctggttctaccccgtcaactccggccagatctccatccgccacgacatgcgcggcccgggcggcgccctggtcgccgagcag
gccggcggcctggacgccgtcgccaaggcccgtcgccacgtccgcgacggcaccccgctgatgctgaccggcggcgtcgacggctccctgtgc
ccgtggggctggctgtgcatgacccgctccggcgccctgaccacccggaccgacccgcgccgcgcctacctgccgttctcccccgacgcctccgg
ctacgtcccgggcgagggcggcgccctgctggtcctggaggacccgcgcgccgccgccgagcggggcgccccgcaggtctacggccgcatcg
ccggctacgccgccaccttcgacccgcgccccggctccggccgggagccgggcctgcgtcgcgccctgcggctggccctggacgacgcgggc
atcggccccgccgacgtggacgtggtcttcgccgacgccatgggcgtccccgccctggacgccgtcgagacccaggtcctggccgccgagttcg
gcccccgcggcgtcccggtcaccgcccccaagaccatgaccggccggctgctggccggtggcgcctccctggacctggccgccgcactgctgtc
cctgcgggaccaggtggtcccgcccaccgtgcacgtggacggcggcgagatccccgactccctggacctggtcaccggcgccccgcgtccggc
ccgcctgcggcacgccctggtcctggcccggggccacggcggcttcaactccgccatggtcgtctccggccgggactgatactagagaaagagg
agaaatactagatgaccctgctgaccctgtccgacctgctgaccctgctgcgggagtgcgccggcgaggaagagtccatcgacctgggcggcga
cgtggaggacgtggccttcgacgccctgggctacgactccctggccctgctgaacaccgtgggccgcatcgagcgggactacggcgtgcagctg
ggcgacgacgccgtggagaaggccaccaccccgcgcgccctgatcgagatgaccaacgcctccctgaccggcgcctcccccctccgccggcgg
cgccgcccgggacaagtgatactagagaaagaggagaaatactagatgtgcggcatcgccggctggatcgacttcgagcggaacctggcccag
gagcgggccaccgcctgggccatgaccgacaccatggcctgccgcggcccggacgacgccggcctgtggaccggcggccacgccgccctgg
gccaccgccggctggccgtcatcgacccggcccacggccgccagccgatgcactccaccctgcccgacggcacctcgcacgtcatcaccttctc
cggcgagatctacaacttccgcgagctgcgcgtggagctggagtcccagggccaccgcttccggacccactgcgacaccgaggtcgtcctgcac
ggctacacccggtggggccgggagctggtcgaccggctgaacggcatgtacgccttcgccgtctgggacgaggcccgccaggagctgctgctgg
tccgggaccggatgggcgtcaagcccctgtactaccaccccaccgccaccggcgtcctgttcggctccgagcccaaggccgtcctggcccaccc
gtccctgcgtcgccgcgtcaccgccgagggcctgtgcgaggtcctggacatggtcaagaccccgggccggaccgtcttctccggcatgcgcgagg
tcctgcccggcgagatggtcaccgtggccggtccggcgtcgcccggcgccggtactggaccctccaggcccgggagcacaccgacgacctgg
agaccaccatcgccaccgtccggggcctgctgaccgaccgggtccgtcgccagctggtctccgacgtcccccctgtgcaccctgctgtccggcggc
ctggactcctccgccgtcaccgccctggccgcccgggccggcgacggcccggtccgcaccttctccgtcgacttctccggcgcgggcacccggttc
cagccggacgccgtccgcggcaacaccgacgccccgtacgtccaggagatggtccggcacgtggccgccgaccacaccgaggtcgtcctgga
ctccgccgacctggccgcccccgaggtccgcgccgccgtcctgggcgccaccgacctgccgcccgccttctggggcgacatgtggccgtccctgt
acctgttcttccggcaggtccggcagcactgcaccgtggccctgtccggcgaggccgccgacgagctgttcggcggctaccgctggttccaccgga
ccgccgccatcgacgccggcaccttcccctggctgaccgccggctccgcccgctacttcggtggccggggcctgttcgaccgcaagctgctggac
aagctggacctgccgggctaccagcgggaccgctacgccgaggcccggaaggaagtccccgtcctgccgggcgaggacgcccgcgaggccg
agctgcgtcgcgtcacctacctgaacctgacccgcttcgtccagaccctgctggaccgcaaggaccggatgtccatggccaccggcctcgaggtc
cgggtccccttctgcgaccaccgcctggtcgactacgtcttcaacgtcccgtgggccatgaagtccttcgacggccgggagaagtccctgctgcggg
ccgccgtccgcgacctgctgccggagtccgtcgtcacccgggtcaagaccccgtacccggccacccaggaccccgtctacgagcgcctgctgcg
ggacgagctggccgccctgctggccgactcccaggccccggtccgcgagctgctggacctgggccgggcccgggacctgctgcgtcgccccgtc
ggcgccgtctcccagccgtacgaccgcggctccctggagctggtcctgtggatgaacacctggctggccgagtacggcgtctccctggagctgtga
tactagtagcggccgctgcag

***oxyABCD* (wildtype)**

gaattcgcggccgcttctagagagaggagactcaatgtccaagatccatgacgcgcgacgcgtcgtaatcacagggatcggcgtggtggctccgg
gagacgtgggcaccaaaccgttctgggagatgctcaccgcgggccgtaccgccacccgcccgatctcgtccttcgacgcctcgcccttccgctccc
aggtggcggcggagtgcgacttcgacccggcggcggaggggctgagccagcggcaggtccgcgcctgggaccgcaccatgcagttcgcttacg
tggccgcgcgggaggcgctggcggacagcggtgtgacgggcgaggcggacccgctgcgcaccggcgtcatggccggcaccgcctgcggcat
gacgatgagcctggaccgcgagtacgcggtggtcagcgacgagggccggctgtggcaggtggacgacgcccatggggtgccgtacctctacga
ctacttcgtgccctcgtcgatggcggccgagatcgcctggctggcggaggcggagggcccggccggggtggtctcggccggctgcacctccggc
atcgacgtgctcacccacgcggcggacctggtacgcgacggcgcggccgaggtgatggtggccggggcatcggacgccgccatctcccccatc
acggtggcgtgcttcgacgcgatcaaggccaccacgccgcgcaacgacgagccggagacggcgtcccgtccgttcgaccgcacccgcaacgg
cttcgtgctgggcgagggggcggcgttcttcgtcctggaggagtacgcgcacgccaggcggcgggggcccgcgcgtacgcggagatcgccgg
gtacgccgggcggtgcaacgcgtacagcatgaccgggctgcgctcggacgggcgggagctggccgaggcggtgagccgcgcgctcgacatcg
cgcgcgtcgatccgtcggaggtggactacgtcaacgcccacggttcggcgaccaagcagaacgatctccatgagacggcggcgttcaagcgga
gcctgggtccgcacgcctactcggtcccgatcagctccatcaaatcgatgatcggccactcgctcggtgccatctgcgcgctggaggtcgcggcga
gcgcgctgaggatcgagcacggggtgatcccacccaccgcgaacctgcgggagccggaccccgactgcgacctggactatgtgccgctggtcg
ccagggaggccgaggtgtccaccgtggtgtccgtggccagcggattcggcggcttccagagcgcgatcgtgctcaccgagccggggaggcagc
gatgaccggccagctcgcccccgctcccgaaaccggcaccggccgccccggcggcagcgtccggccggtggtcaccggcctcggtgtggtcgc
gcccaacggcctgggcaccgagcggtactgggccgcgacgctgcgcggcgacagcggcatcggccggatcacccggttcgacccgtccggct
acacctcctcgctggccggggagatcgcggacttcgaccccgcgcggctgccgaaccggctgctgccgcagaccgacctgatgacccggctcgc

gctggtcgcggccgaggaggcgctggacgacgcgggcgccgacccgcgcacgatgcccgacttcgccgccggggtggtcaccgccgcgtcgg
cgggcggcttcgacttcggccagcgggagctggaggccctgtggagcaagggtggcgcgcacgtcagcgcgtatcagtccttcgcctggttctatc
cggtcaactccggtcagatctcgatccggcacgatatgcggggccccggcggcgcgctggtggccgagcaggccggcgggctggacgcggtgg
ccaaggcgcgccgccacgtacgggacgggacgccgctgatgctcaccggcggtgtggacggttcgctgtgcccgtggggctggctgtgcatgac
ccgttccggcgcgctgaccacccggaccgatccgcgccgcgcgtacctgcccttctcccccgacgcctccggatacgtgccgggtgagggcggg
gcgctgctggtcctggaggaccctcgggccgccgcggagcggggtgccccgcaggtctacgggcggatcgccgggtacgcggccaccttcgac
ccccgtccgggctccggccgggaaccgggtctgcgcagagcgctccggctggcgctggacgacgccgggatcggaccggcggacgtggacgt
ggtcttcgccgacgcgatgggcgtaccggcgctcgacgcggtggaaacacaggtgctggccgccgagttcggcccgcgcggggtgccggtgac
ggctcccaagacgatgacgggacggctgctggccggcggcgcctctctggacctggcggcggcgctgctgtcgctgcgcgaccaggtcgtaccg
ccgacggtgcacgtggacggcggcgagatcccggactcgctggacctggtgaccggcgctccccggccggcgcgcctgcggcacgcgctggtg
ctggcccgcggtcacggcgggttcaactccgcgatggtggtcagcggccgggactgagccgccgtcagctgctcgcttccggcccggccggtctc
atgaatcctctgcgactccccgaaaggtaggactgtcatgaccctgctcaccctctccgacctgctcaccctgctgcgcgagtgcgcgggcgagga
ggaatccatcgacctgggaggggacgtcgaggacgtcgccttcgacgccctcggctacgactcgctggcgctgctgaacaccgtgggacgcatc
gagcgcgactacggggtccagctcggggacgacgcggtggagaaggcgaccacgccgcgcgccctgatcgagatgaccaacgcgtcgctca
ccggcgcgtccccgtccgcgggtggcgccgcgcgggacaagtgaggaccggccgatgtgcggaatcgccggctggatcgatttcgaacgcaat
ctggcgcaggagcgggcgacggcctgggccatgacggacaccatggcctgccggggcccggacgacgcgggcctgtggaccggtggccacg
cggcgctcggccaccgcaggctggccgtcatcgacccggcgcacgggcggcagccgatgcacagcacgctgccggacggcacgagccacgt
catcacgttcagcggggagatctacaacttccgtgaactgcgggtcgagctggagtcccaggggcaccgcttccgtacccactgcgacacggagg
tggtgctgcacggctacacccggtggggccgggagctggtggaccggctcaacggcatgtacgcgttcgccgtgtgggacgaggcccggcagg
aactgctgctggtccgcgaccggatgggggtcaagccgctctactaccaccccaccgccaccggtgtgctgttcggctccgagccgaaggccgtg
ctcgcgcacccctcgctgcggcggcgggtgacggccgagggcctgtgcgaggtgctggacatggtcaagacccccggccggaccgtcttctccg
gtatgcgcgaggtgctgccgggcgagatggtcaccgtcgggcgctccggtgtggcccgcaggcggtactggacgctccaggcgcgggagcaca
ccgacgatctggagaccaccatcgccacggtgcgcggcctgctgacggacagggtgcgccggcagctggtctccgatgtgccgctgtgcaccctg
ctctccggcgggctggactcgtcggcggtgacggcgctggcggcgcgggcgggcgacgggccggtccgtacgttctcggtggacttctccggcgc
gggcacccggttccagccggacgccgtacggggcaacacggacgcgccgtacgtccaggagatggtgcggcacgtggcggcggaccatacg
gaagtcgtgctggacagcgccgatctcgcggcacccgaggtgcgtgccgccgtcctgggggccaccgacctgcccccggcgttctggggcgaca
tgtggccctcgctgtacctgttcttccggcaggtgcggcagcactgcacggtggccctgtcgggcgaggcggcggacgaactcttcggcggttaccg
gtggttccaccggacggcggccatcgacgccggtacgttcccctggctgaccgccggctcggcccggtacttcggcggccggggcctgttcgacc
gcaagctgctggacaagctcgacctgccgggctaccagcgcgaccggtacgccgaggcccgtaaggaagtccccgtcctgcccggcgaggac
gcgcgggaggcggagctgcgccgggtgacctacctcaacctcacccgcttcgtgcagaccctgctggaccgcaaggaccggatgagcatggcg
accggcctggaggtgcgggtgccgttctgcgaccaccggctggtcgactacgtcttcaacgtgccgtgggccatgaagtccttcgacggcagggag
aagagcctgctgcgggccgcggtgcgcgacctgctgccggagtcggtggtcacccgggtcaagacgccctaccccgccacacaggacccggtc
tacgagcggctgttgcgcgacgaactggccgcgctgctggccgacagccaggcgccggtccgcgaactgctcgacctgggtcgggcgcgcgac
ctgctgcggcggccggtcggcgccgtcagccaaccgtacgaccgcggcagtctggagctggtcctgtggatgaacacctggctggcggagtacg
gcgtcagcctggagctatgatactagtagcggccgctgcag

snoaDEMB

gaattcgcggccgcttctagagaaagaggagaaatactagatgccggataccgacggccgggtggcggtcgtgaccggggtaccagcgggat
cggcctggcagtagtcaaggcgctcgccgccacgggctacgcctgccggtcgctgtgcgcccgcagccaggacgccgtcgacagcacggtgga
ggaactgcgggacgagaagctggaggtggacgggcggtcctgcgacgtgcgttcgaccgacgacgtccgcgagttcatcgggttcgccacccg
gcgcttcggcggggtcgacgtcctggtcaacaacgccggccgcaacggcggtggacccacggcgcgaatcacggacgagctgtggctggacgt
gatcgagaccaacctcacctcggtcttccgcgtcacacgggaggtgctgacgaccggtggcatgctggccgcgggcaggggacgcatcatcaac
atcgcctccaccggcggcaagcagggcgtggtcctcggcgccccgtactcggcgtccaagcacggcgtcgtcggcttcaccaaggccctgggcc
tcgaactcgcgaagtccggcatcaccgtgaacgcggtgtgcccggggtacgtcgagacgccgatgggcgagcgcgtggccgcggctacgccg
aggtctgggacacgtccgaggaagccgtgctggagcggttccaggccaagatcccgctgggtcgctacgcgcagcccgaggaggtcgccgggc
tcgtcacctacctgaccacccccaccgcggactccatcaccgcccaggcgctcaacgtctgcggcggcctcggcaactactgatactagagaaag
aggagaaatactagatgaccgccggtgacacgaccctgaccacccaccggaccgagcaccgggtgaccgtgagcgcccccgcgcggcgggt
cttcgacctggtcgccgacatcaccggctggccgcacaccttccccccccaccgtgcacgccgagtacgcggaacgcggcgcctccgaggaacg
aatccgcatctgggcgacggccaacggggaggtcaaggcctggaccagccaccgctcgctcgaccgcgagggcctccgggtgcggttccggc
aggagaagtcccagcaccccgtcgcgggcatgggcggcgagtggatcatccggccggtcgacggcgagcggtcggaggtcatcctgacgcac
gacttccaggcggtcgaggacgacccggcccacgtcgactggatccaccgcgccgtagatagaaacagcggtgcggagctggccgccctgaa
gccgcgccgcaacgcccgcacggcccgtaacggcacgctcttcacgttcgcggacgaggtgaccgtcaccggagcacccggcgacgtgtacg
actacctgaacgaggcccgtcgctggcaggaacggctgccgcacgtggcggcggtgtcgctggacgagcccgcgccgggcgtgcagcgcctc
gggatggacaccaagggccccgacggcaccgtccacacgacggagtcggtccgcatctgcttcccgcaggaccggatcgtctacaagcagttc
accatgccgccgctgatggccgtgcataccgggacctggcggatcgcccccgctgcggacgggcacgggaccacggtcacctccctgcacacc

gtcgtcgtggacgactccgccgtcactacggtgctcggcccggacgccaccctcgccgacgcccggcgcttcctgcgcgacgcgctgggccgca
actcccgcaccacactcggcttcgccaaggagtacgccgaggagcgcgcgcgccgggcgtgatactagagaaagaggagaaatactagatga
ccgccgcctggggcgccccgctgtacccgccctggatcccggcgcgtccggggcgccggcgatgcggcgcggggcggcgggtccgctgcccg
ccggtggagccggcctcccggccccggcaggagggccgggtcagcgtggtgcccgccctcaggcagccctcgccgtcgaccaacccggaggt
gcgcgtccgactgatcgacctgtcgtcccccgtggactcctcccagtacgagcccgacccggtggtgcacgacgtccttacgccgcgccagggcg
cggaacacatgtgcgccgagatgcgcgagcacttcggtgtcgagttcagccccgacgaactgccggacggcgagttcctctccctggaccggatc
accctgaccacccacacggggacgcacgtcgacgccccctcccactacggcagccgcgcgctctacggcgacggggtgccgcggcacatcga
ccagatgccgctcgagtggttcttcggccgcggcgtggtgctggacctcaccgacgcgcccaccggcaccgtttcggccgcgcgtctggagaagg
agctggcccgcaccggctgtgcgctgcgccccggcgacatcgtcctgctgcacaccggtgcccagcgccacgccggaacgccccgctacttcac
cgacttcgccgggctcgacggcccagccgtacggatgctgctcgaccacggcgtccgggtgatcggcacggatgccttctcactcgacgcaccgtt
cggccacatcatcgaccggtaccgggccacgggagaccgcagcgtgctgtggcccgcccatgtcgtcggccgcgagcgggagtactgccagat
cgagcgcctcgcgaacctggaccgcctccccgtctccttcggcttccgcgtctgctgcttcccggtcaaggtcgcgggtgcgggggcaggctggac
ccgggcggtcgccctggtcgacgaggactgatactagagaaagaggagaaatactagatgccgacccgggtcaacgacggcgtcgacgccga
cgaggtcaccttcgtcaaccgcttcacggtgcacgggggacccgcggagttcgagtcggtcttcgcccggacggccgccttcttcgcgcggcagcc
cggcttcgtgcgccacaccctcctgcgcgaacgcgacaaggacaactcctacgtgaacatcgccgtgtggaccgaccacgacgcgttccggcgt
gcgctcgcccagccggggttcctgccgcacgccacggcgctgcgggcactcagcacgtccgagcacggcctgttcaccgcccgccagaccctg
cccgagggcggcgacaccaccggctcgggtcaccgctgatactagtagcggccgctgcag

aknAE1WX

gaattcgcggccgcttctagagaaagaggagaaatactagatgccgcccgcctccgagcgagtcgcgatcgtcaccggtgccacgtccggcatc
ggcctggccgtggcgcgctccctcgccgagggcggtgctcgggtgttcatctgcgcccgcgacggcgaccgcgtcgcccacacggtgaaggagc
tgcgcgaggccgggcacgacgtcgacggggcgtcctgcgacgtgcgggacaccgcgcgggtacgcgccttcgtccaggaggcgagggaccg
gttcggcccggtggacgcactggtcaacaacgccggacgcagcgggggcggccacaccgcccagatcgccgacgagctgtggctggacgtca
tcgagaccaacctgaactccgtcttccggatgacccgggaggtcctgaccaccgggggcatgctggaacgcggcgcgggccggatcgtgaacat
cgcctcgacgggtggcaagcagggcgtcgccctcggcgccccgtactcggcgagcaagcacggcgtggtgggcttcaccaaggccttggggct
cgaactcgcgaagaccggcatcaccgtcaacgcggtgtgccccggctacgtcgagacgccgatggccgagcgtgtccggcaggggtacgcgg
gggcctgggacatcaccgaggacgaggtgctcgagcgcttcgaggccaagatcccgctgggccgctactccacccccgatgaggtggccggac
tcgtcggctacctgctgtcgagcacggccgcgtcgatcacggcgcaggccatgaacgtctgcggcggcctcggcaactactgatactagagaaag
aggagaaatactagatggcgcacgaggtgcacaccacggagcacaccatcacggtcgccgccgccccggaggcggtcttcggcctggtcgag
cgcgccgaggattggccgcacgtgttccccccctcgctgcacgtggagtacttggagcgcagcggggaggaagagcgtctcaggatctgggcga
cggccaacggcgaggtgaagtcgtggaccagccggcgaacgctggaccgggccggactgcggatccggttccggcaggaggtctcccaggac
cccgttgcggcgatgggcggggagtggatcgtggaggcgctccccggcggcgggtcgcgggtccggctgctgcacgacttccgcgccgtcgacg
acctggagcggaacgtcagctggatcctcgaggcgatcgagcgcaactccggcgccgagctggccgccctggagagcgcggcgctgcggacg
ggaaccgacgaggcgccgtacaccttcgaggactccgtgcgtatcgacgcccccgcgcaggacgtgcgtgacttcctccacgacgcgggccagt
ggcaggaacgcctcccgcacgtcgcgcgcgtcctcctcaaggaggacaccccggacgtccagcacctggagatggacacccggaccccggat
ggctccacccatacgaccgcctccgtgcggatctgcctgcccgacgggcgcatcacctacaagcagctccagacccccgccctgatgagcgtcc
acaccggtgagtggtccgtccgcgagatcccgagcggctgcgtggcagtttcgcggcacacagtagtcgtcaacgccgccgccgtcaccaaggt
cctcggcgcgtccgcgaccaccgccgacgcccgcaccttcatccgcgacgccctcggcggcaacagtacgatcaccatgcggcacgccaagg
agtacgcccagagacgcgcagccaccgagccggtgccgccggccgagtacgcgtctgtgcagcgcttctacgcccgccagatgcgcctgctcg
acgagggcgaaggcgacgcgtgggccgacacgttcaccgaagacggcacgttcgaccagtcctccttcgccgaacccccttcgaggtcgggcgg
ccatcgcggctgccgtgcgcggccgcccagcggctcccgccgggaccgtccgccgccactggctgggcctcccggcggcatggcggttcccgg
acggctcggtgcggacggcctacgacgcactggtcgtgtcgacggaggccggtgccgccccggtgatccggctgtccacctcctgccaggacgtc
ctggtggcggacggcgacggctggctggtctcgcaccgctacgtcggccacgacgggcaatgatactagagaaagaggagaaatactagatgc
ggatcatcgacctctcctcgccggtggacgccgccggcttcgagcccgaccccgtcgtgcacgacgtcctcggccccaaggaggcggccaccca
catgagcgaggagatgcgcgagcacttcggcatcgacttcgaccccgccgagctgcccgagggcgagttcctgtcgctggaccggctccagctg
accacccacaccggcacgcatgtcgacgcgcccagccactacggcacccgcgccgcgtaccgcgacgggccgccgcggcacatcgacgag
atgccgctggactggttcttccgccccgccgtggtcctggacctgtccgaccagggcaccggtgccgtcggcgccgacgtgctgcgccgagaaatg
gaccgcatcggccacacgccctccccgatggacatcgtcctcctccgcaccggggcggacgcctgggcgggtacgccgaagtacttcaccgactt
caccggcctcgacggcagcgcggtccacctgctcctggacctgggcgtacgcgtcatcgggacggacgccttctccctcgatgccccgttcgggg
acatcatcacccgctaccgggccacgggcgacccgtcggtgctgtggccggcgcacgtgatcggccgggaccgtgagtactgccaggtcgagc
ggctggccgggctcgacaggctgccggctgcccacggcttccgggtcgcctgcttcccggtccgcatcgccggagcgggagcaggctggacccg
ggcggtggcgctggtggacgaatgatactagagaaagaggagaaatactagatgaccgaccacgagccgggcaccgagggcgccgacgcc
gtcacgttcgtgaacaccttcaccgtgcacgcggaacccgaggtcttcgagaaggagttcgccaggacgtccgagttcatggcccgccagcccgg
cttcgtccgccacaccctgtgccggcacgccgagcggccggggcagtacgtcaacgtcgccgagtggcgggacctggcctccttccgggcggcg

gtgtcgcacgacgacttccgcccgcacgccggggcgctgcgcgcgctcagcgagagccgtccggagctgtacctggtgcgcctccgccgggag
ggcgcgcccggtctcgacggcccggcctcggaaggcgaggagatctgatactagtagcggccgctgcag

dpsEFYdnrG

gaattcgcggccgcttctagagaaagaggagaaatactagatgtccgaggccgccgaccgggtggccctggtcaccggcggcacctcgggcat
cggcctggccgtcgtccggaagctggcccaggacggcacccgcgtcttcctgtgcgcccgggacgagtccgccatcaccggcaccgtcaagga
gctccaggcctccggcctggaagtggacggcgccccctgcgacgtccgctccaccgccgacgtggaccggctggtccagaccgcccgcaaccg
gttcggccccatcgacatcgtcgtcaacaacgccggccgcggcggcggcggcgtcaccgccgagatcaccgacgacctgtggctggacgtcgtg
gacaccaacctgtccggcgccttccgggtcacccgggccgtcctgaccggcggcgccatgcaggagcacggctggggccggatcatctccatcg
cctccaccggcggcaagcagggcgtcgccctgggcgccccgtactccgcctccaagtccggcctgatcggcttcaccaaggccgtggccctgga
gctggccaagaccggcatcaccgtcaacgccgtctgccccggctacgtggagaccccgatggcccagggcgtccgccagcggtacgccgccttc
tggggcatcaccgaggacgacgtcctggagaagttccaggccaagatcccccctgggccgctactccatgccggaggaagtcgccggcatggtcc
actacctggcctccgactccgccgactccatcaccgcccaggccatcaacgtctgcggcggcctgggctcctactgatactagaaagaggagaaa
tactagatgtccgagctgcccctccagcagaccgagcacgagatccacacctcggccgccccggacgccgtcttcgccgtcctggccgacgccc
gtgcctggccggccgtcttcccgccctccgtccacgtggagcaggtggagcacaccggctcctccgagcgcatccggatctgggccaccgccaac
ggctccctgcgcacctggacctcgcgccgcgagctggacgagcgggcccgccggatccgcttccggcaggaagtctccgcccacccggtggcc
gcgatgggcggcgagtggatcgtggaggaagccggcgacggcggcacccgcgtccggctgacccacgacttccgggccgtggacgacgacc
ccgagaccatcggctggatccaccgggccgtggaccggaactccgaggccgagctggcctccctgcgcaccgccctggagcggcccgacggc
accgcccccaccaccttcgaggacaccgtggtggtccgcggtcgcgccgaggacgtctacgacttcctgcaccggtccgacctgtggaagaagc
gcctgtcccacgtggcccggatcgccgtcaaggaagaggagcccggcctccagcacatggagatggacaccctgaccgccgacggctccgtcc
acaccaccgcctccgtccgggtctgcttccccgagcgtcgcgtcatcgtctacaagcagctgcggaccccgcccctgctggccctgcacctgggcc
gctggtccgtccggcccgccgacgacggcgacggcatcgccgtcacctcggcccacaccgtctccgtcgcccgctccgccatcccgggcgtcctg
ggcgccggcgcctccgagaccgacgccgtggacttcgtccgtcgcgccctgggccgcaactccctgctgaccctggaggccgcccggcagtacg
ccgagtcctccgcctgatactagaaagaggagaaatactagatgcgcatcatcgacatctcctccgccgtggacgcctccggctgggagcccgac
gaggtgcggcacgaggtcctctccccgcgggagggcgccgtccacatgtccgaggagatgcgccggcacttcggcgtggccttcgaccccgacg
agctgccggagggcgagttcctgtccctggaccggctgaccctgacctcgcacaccggcacccacatcgacgcccccctcccactacggctcccg
ggcccactacggcgacggtcgcccgcgcaacatcgacgagctgcccctggactggttctacggccccggcctgctgctggacctgaccggctgc
gagggccccaccgccggcgccggcgacctggagaaggagctggcccgcatcggccgggtcccggagcccggcaccatcgtcctgctgcgca
ccggcgcctccgagcgggccggcaccgagcagtacttcaccgacttcaccggcctggacggcccggccgtcaacctgctgctggaccacggcgt
ccgggtcatcggcaccgacgccttctccctggacgccccctttcggcgccgtcatccgccgctaccgcgagaccggcgaccggtccgtcctgtggcc
cgcccacgtcaccggccgccaccgggagtactgccagatcgagcggctgggcaacctggccgccctgcccggctgcgacggcttccaggtggc
ctgcttccccgtcaagatcaccggcggcggcgccggctggacccgggccgtcgccttcgtggacgagtgatactagaaagaggagaaatactag
atgcccctgccggagcccaacgacgccggctccggctccgtcaccttcgtcaaccgcttcaccctgtccggctccgccgaggacttcgaggccgcc
ttcgccgagaccgccgagttcctgtgccgccggcccggcttccgctggcacgccctgctggtccccgccgacaccggccccggctccgccgacgc
ccgcccgcagtacgtcaacatcgccgtctgggacgacgaggcctccttccgggccgccgtcgcccaccccgagttccccgcccacgccgccgca
ctgcgggccctgtccacctcggagccgaccctgtaccgccaccggcagatccgcgtcgcccccgacgtcccggccgtctccggcccgggtggcc
gcaccacctgatactagtagcggccgctgcag

snoaLCF

gaattcgcggccgcttctagagaaagaggagaaatactagatgtcccgccagaccgagatcgtgcgccgcatggtgtccgccttcaacaccggcc
gcaccgacgacgtcgacgagtacatccacccggactacctgaacccggccaccctggagcacggcatccacaccggccccaaggccttcgcc
cagctggtcggctgggtccgggccaccttctccgaggaagcccgcctggaggaagtccggatcgaggagcggggcccctgggtcaaggcctac
ctggtcctgtacggccgccacgtgggccggctggtcggcatgcctccgaccgaccgccggttctccggcgagcaggtccacctgatgcggatcgtc
gacggcaagatccgcgaccaccgggactggcccgacttccagggcaccctgcgccagctgggcgacccgtggcccgacgacgagggctggc
ggccctgatactagagaaagaggagaaatactagatggacaccgccgagtacaagcagaccgtcaccgccgccttcgaccggccgccgcac
actacgaccggctgggcgtcgagttcttcaccccgatgggcctgcgcctggtcgaccacgccgacccgcgccccggccagcgggtcctggacgtc
ggctgcggccgcggtgcctgcctgttcccggccgcacgtcgcgtcgcccccgacggctacgccctgggcatcgacatcgccccggccatggtcga
ggaagccgccgccgaggccgagcgcctgggcatgccgcacgtcgaggtccgggtcatggacggcgagcggccggccctgccggagcgctcct
tcgagcgggtcctgggctcctactccgtcatcttcttcccggaggccctgccggccctggtccgctacgccggcctgctggccgacggtggccggatc
gccttcacctcgccggtcttcaccgaggacaccttccccttcctgccgcccgtcttcaccgagctgatcccccgctccctgctggacaccctgccgccc
gactggcggcccgaggccctgcgccggcgcttcaactcctggctggagcgcccggacgacctggaggccaccatgcgtcgcgccggcttcggc
gaggtcggcatcaccgacgagacggtccccatggtggccgtctccggcgaggcctgggtcgactggtcccacaccaacggcatgcgcctgctgt
ggcagcacctgccgcgggacgaggcccagcggctgcgggagcggctgatcaccgagctggacgccatgcgtcgcgccgacgccccgctgtcc

atcccggtccccgtccgcttcgtcaccgccgtcgtcgaccggtgatactagagaaagaggagaaatactagatgcgcgccatgaccgactccacc
ggcccgcgtccggtcccggccatgtcccccgccccgtccccgaccccgtccccgggccccgcccccggctccgagccggccccgctggccgtca
tcgtcaccggcggcggctccggcatcggccgcgccaccgcccgggccttcgccgcccagggcgccaaggtcctggtcgtcggccggaccgagg
acgccctggcccagaccgccgagggctgcgccgacatgcgcgtcctggtcgccgacgtggcctcccccgacggcccccaggccgtcgtcaacg
ccgccctgcgcgagttcggccggatcgacgtcctggtcaacaacgccgccgtcgccggcatggagacgctccagaccgtggaccgggacgccg
tggcccggcagttcggcaccaacctgaccgccccgctgttcctggtccagtccgccctgggcgccctggagaagtcccggggcatcgtggtcaac
gtgggcaccgccgccaccctgggcctgcgggccgccccgaccggcgccctgtacggcgcctccaaggtggccctggactacctgacccgcacc
tgggccgtggagctggccccgcgtggcatccgggtcgtcggcgtcgcccccggcgtcatcgacaccggcatcggcgtgcggatgggcatgaccc
cggagggctaccgggagttcctgaccggcatgggcggccgggtccccgtgggccgggtcggtcgcccggaggacgtggcctggtggatcgtcca
gctggcccgtccggaggccggctacgccaccggcatggtcgtccccgtcgacggcggcctgtccctggtctgatactagtagcggccgctgcag

snoaC+kyc34+snoaF

gaattcgcggccgcttctagagaaagaggagaaatactagatggacaccgccgagtacaagcagaccgtcaccgccgccttcgaccgggccgc
cgcacactacgaccggctgggcgtcgagttcttcaccccgatgggcctgcgcctggtcgaccacgccgacccgcgccccggccagcgggtcctg
gacgtcggctgcggccgcggtgcctgcctgttcccggccgcacgtcgcgtcgcccccgacggctacgccctgggcatcgacatcgccccggccat
ggtcgaggaagccgccgccgaggccgagcgcctgggcatgccgcacgtcgaggtccgggtcatggacggcgagcggccggccctgccggag
cgctccttcgagcgggtcctgggctcctactccgtcatcttcttcccggaggccctgccggccctggtccgctacgccggcctgctggccgacggtgg
ccggatcgccttcacctcgccggtcttcaccgaggacaccttccccttcctgccgcccgtcttcaccgagctgatcccccgctccctgctggacaccct
gccgcccgactggcggcccgaggccctgcgccggcgcttcaactcctggctggagcgcccggacgacctggaggccaccatgcgtcgcgccgg
cttcggcgaggtcggcatcaccgacgagacggtccccatggtggccgtctccggcgaggcctgggtcgactggtcccacaccaacggcatgcgc
ctgctgtggcagcacctgccgcgggacgaggcccagcggctgcgggagcggctgatcaccgagctggacgccatgcgtcgcgccgacgcccc
gctgtccatcccggtccccgtccgcttcgtcaccgccgtcgtcgaccggtgatactagagaaagaggagaaatactagatgtcctcccagaccgac
gccgtccgtcgcgtcatcaacgccttcaacaccggcaagaccgacgacgtcgacgagtacatccacgacgagtacctgaaccccgccaccctg
gagcacggcatctcccagggccccaaggccttcgccatggtggtggcctgggtccgtcgcaccttctccgaggaagcccacctggaggagatca
acgtcaccgagaacggcgagtgggtccgggcccacctggtcctgtacggccggcacgtgggcaacctggtcggcatcccgcccaccggccgtc
gcttcgccggcgagcagatccacctgttccgcatcgtcgacggcaagatccgcgaccaccgggactggcccgacttccagggcaccctgcgcca
gctgggcgacccgtggcccgagaacggtggctggcggccctgatactagagaaagaggagaaatactagatgcgcgccatgaccgactccac
cggcccgcgtccggtcccggccatgtcccccgccccgtccccgaccccgtccccgggccccgcccccggctccgagccggccccgctggccgtc
atcgtcaccggcggcggctccggcatcggccgcgccaccgcccgggccttcgccgcccagggcgccaaggtcctggtcgtcggccggaccgag
gacgccctggcccagaccgccgagggctgcgccgacatgcgcgtcctggtcgccgacgtggcctcccccgacggcccccaggccgtcgtcaac
gccgccctgcgcgagttcggccggatcgacgtcctggtcaacaacgccgccgtcgccggcatggagacgctccagaccgtggaccgggacgcc
gtggcccggcagttcggcaccaacctgaccgccccgctgttcctggtccagtccgccctgggcgccctggagaagtcccggggcatcgtggtcaa
cgtgggcaccgccgccaccctgggcctgcgggccgccccgaccggcgccctgtacggcgcctccaaggtggccctggactacctgacccgcac
ctgggccgtggagctggccccgcgtggcatccgggtcgtcggcgtcgcccccggcgtcatcgacaccggcatcggcgtgcggatgggcatgacc
ccggagggctaccgggagttcctgaccggcatgggcggccgggtccccgtgggccgggtcggtcgcccggaggacgtggcctggtggatcgtcc
agctggcccgtccggaggccggctacgccaccggcatggtcgtccccgtcgacggcggcctgtccctggtctgatactagtagcggccgctgcag

dnrCDE

gaattcgcggccgcttctagagaaagaggagaaatactagaaagaggagaaatactagatgcaggactcctcctacaaggagcaggtcaccca
ggccttcgaccagtcctcctccacctacgaccgcctgggcgtcgagttcttcaccccgatgggccgcccgctggtcgagatcagcgagcccgtcac
cggcgagcgggtcctggacatcggctgcggccggggcgcctgcctgttcccggccgccgagaaggtcggcccccagggccgcgtccacggcat
cgacatcgcccccggcatgatcgaggaagcccgcaaggaagccgccgagcgcggcctgcggaacatcgccctggacgtcatggacgccgag
accccggagctgccggcccgctccttcgacctggtcatgggctcctactccgtcatcttcctgcccgacgccgtgggcgccctggcccggtacgccg
gcatcctggaccacggcggccggatcgccttcacctcgcccgtcttccgcgccggcaccttccccttcctgccgcccgagttcaccccgctgatcccg
caggccctgctggagcacctgccggagcagtggcgcccggaggccctggtccgccggttcaactcctggctggagcgggccgaggacctgctgc
ggaccctggagcgctgcggctacacctcggtcgccgtcaccgacgagcccgtgcggatgaccgccctgtcctccgaggcctgggtggactggtcc
cacacccagggcatgcggctgctgtggcagaacctgccccaggcccagcggaccgagctgcgcgcccggctggtcgagggcctggacaagct
gtccgacgccaccggcgccctggccatcgacgtcccggtccgcttcgtcaccgcccgggtcgcccactgatactagagaaagaggagaaatact
agatgtccacccagatcgacctggtccgtcgcatggtcgaggcctacaacaccggcaagaccgacgacgtcgccgagttcatccacctggagta
cctgaaccccggcgccctggagcacaaccccgagctgcggggccccgaggccttcgccgccgccgtcacctggctgaagtacgccttctccgag
gaagcccacctggaggagatcgagtacgaggagaacggcccctgggtccgggccaagctggccctgtacggccggcacgtgggcaacctggt
gggcatgcccgccaccggccgccggttctccggcgagcagatccacctgatccggatcgtggacggcaagatccgggaccaccgggactggcc
ggactacctgggcacctaccgccagctgggcgagccgtggcccaccccggagggctggcggccgtgatactagagggaggagtccatggacg
gacgtccacggccagcacggcaccgctggcgtacgcggcttcgggatcggcgagccgcaccacccaccacgcgacgtcctcgggccggccca

cacggccgagcgggacgcgctgccccatggcttccaggaacccgtcgtacgcctcctgggacatcccggcccggacccccgcgcccgtcccga
tcactcccggggcgacaccgaccacgcgaatgccgcggggaccgagttccacggcccaggaccgcgtcagcaggtccaggccggccttggcc
gccccgtacaccgcgttccccggccaggcccgccggccgagtgcgcccgccgaaccgatgttcaccaccaggccggaagcggtctccagggg
gccgagcagggcctgggtgagcagcacgggggccacgaggttcgtggccacctgtgcctccaccgcggtgcggtgcagctctcccaggtgccc
gaaggcggccgtggcggcattgttcacgagcacgtcgaccacgccgccgagccgctcgcgcacctcccggacgaccgcctccggcgccgcgg
ggtcggtgatgtccacggccaacgtgtgcgcgtccttgtgcccgtccaccgtctcggccagcggaccggcggtcctgcccacgacgaggacgcgg
tcgccccgggcggcgaaggcgcgggcgacggctcgtccgatgccggagccgccgccggtcacgatcacggatcgctgtgtgttctccatgcggc
gaggctaggcaccgccggtggaaggcgacttga tactagtagcggccgctgcag

aknGHU

gaattcgcggccgcttctagagaaagaggagaaatactagatggacacctccacctacaagtccgaggtggccgccgcattcaactccgccgcc
gccacctacgaccgcctgggcgtcgagttcttcaccccgatgggccgccggctggtcgagcgggccgccccggccgccggtgcccgcgtcctgg
acgtcggctgcggccgcggtgcctgcgtcttcccggccgccgagcgggtcggccccgagggccgcgtcgtcggcatcgacgtcgccgaggccat
gatcgaggaagccaccaaggaagccgccctgcgggacgccgccatggtggagctccgggtcatggacggcgagcaccccgacttcgccccgc
actccttcgacgtggtcctgggctcctactccgtcatcttcctgcccgacgcccccaccgccctggcccgctacgccccgctgctgtccccgggtggcc
gcctggccttcacctcccccgtcttctccgacgacaccttccccttcctgccgcccgtcttcaccgagctgatcccccgggagctgctgctggacctgcc
gccccagtggcagcccgagcagctccagcagcgcttcaactcctggctggccgacccccgcgacctgacccggaccctggagcgctgcggcttc
caggaagtcaccgtggtggacgagcccgtcaccctggtcgccgagtccggcctggcctgggtggactggtcccacacccagggcatgcgcctgct
gtggaaccacctggcccaggacaagcgccagcagctgcgggagcggctgatcacctccctggacgccatgcgggacggcgacggcccgctg
accatcgacaccccggtccgctacgtcaccgccaccgtccgcaactgatactagagaaagaggagaaatactagatgtccgagcagatcgccg
ccgtccgtcgcatggtggaggcctacaacaccggcaagaccgacgacgtcgccgactacatccacccggagtacatgaaccccggcaccctgg
agttcacctccctgcggggccccgagctgttcgccatcaacgtcgcctgggtcaagaagaccttctccgaggaagcccgcctggaggaagtcggc
atcgaggagcgggccgactgggtccgggcccggctggtcctgtacggccgccacgtgggcgagatggtcggcatggcccccaccggccggctg
ttctccggcgagcagatccacctgctgcacttcgtcgacggcaagatccaccaccaccgggactggcccgactaccagggcacctaccggcagc
tgggcgagccgtggcccgagacggagcaccgtcgcccgtgatactagagaaagaggagaaatactagatgaaccgcgccgcccgtccgacc
gtccgcggcatgtccgccatcgccgagcccaccgccccgcgcggcgtcatcgtcaccggcggcggcaccggcatcggccgcgccaccgccca
cgccttcgccgaccgcggcgaccgggtcctggtcgtcggccggaccgccgccaccctggccggcaccgccgagggccaccccggcatctccgt
cctgaccgccgacctgaccgaccccgacggcccccgcgccatcaccgacgccgccctggacgccctgggccggatcgacgtcctggtcaacaa
cgccgccaccggcggcttcgccggcctggccgagacggagccggaggccgcccgcgagcagttcgactccaacctgctggccccgctgctgct
gacccgccagaccctggacgccctgtccgccgacggtggcggcaccgtcctgaacatcggctccgccggcgccctgggccgtcgcgcctggcc
gcagaacggcgtctacggcgccgccaaggccggcctggacttcctgacccggacctgggccgtggagctggccccgcgtggcatccgggtcctg
ggcctggcccccggcgtgatcgacaccggcatcggcgagcgctccggcatgtcccgggaggcctacgccggcttcctgggccagatcgccgccc
gggtcccggccggtcgggtcggtcgcccggaggacatcgcctggtgggccgtccagctggccgacccccgggccgcctacgccaccggcgccg
tcctggccgtcgacggcggcctgtccctgacctgatactagtagcggccgctgcag

Table S2. Synthetic BioBrick RFC[10] promoter and terminator parts (5' → 3') used in this study.

***ermE*p* promoter** [3]

gaattcgcggccgcttctagagggtaccagcccgacccgagcacgcgccggcacgcctggtcgatgtcggaccggagttcgaggtacgcggctt
gcaggtccaggaaggggacgtccatgcgagtgtccgttcgagtggcggcttgcgcccgatgctagtcgcggttgatcggcgatcgcaggtgcacg
cggtcgatcttgacggctggcgagaggtgcggggaggatctgaccgacgcggtccacacgtggcaccgcgatgctgttgtgggcacaatcgtgcc
ggttggtaggatcctactagtagcggccgctgcag

***kasOp** promoter** [4]

gaattcgcggccgcttctagtgttcacattcgaacggtctctgctttgacaacatgctgtgcggtgttgtaaagtcgtggccaggagaatacgacagcg
tgcaggactgggggagtgcgcattactagtagcggccgctgcag

***p15* promoter (promoter for *xnr1500* gene in *S. albus* J1074)**[5]
gaattcgcggccgcttctagagtccgcgccgccggcccgacggtgcccggccccgtacccccccgggtggtgcggggccgggcaccggcctttt
ggcgctgcggagttgacggaagttggccgaaccggatgcgctcggcgcccgggggctgaaagatgctcacagcccctttccacggcggtccggg
aggggaggccgggcaaccggttttcgggggcggagtgtccggtatgcggacggccgcgcccgatagatgtgtaacgagtccgtttcgcaaccatc
tatctcggatcggtttgtccggattttggaagatgtgagtgtcaggtgtgatcgaaccgagaccaaaagggtgtggtcgggccgaacaccatggcta
atagttgagcgcgtagagctcgggtcaatgggtcacgcgctgtggggagcgccgactcacgagcacactggggcactcgatcttcgccgtcaggg
gtgtcggcggatcgtcctgtgccctctcttgcagtgaacaagtggactcattactagtagcggccgctgcag

***sp44* promoter** [6]

gaattcgcggccgcttctagagtgttcacattcgaaccgtctctgctttgacaacatgctgtgcggtgttgtaaagtctggtgtatactagtagcggccgct
gcag

***tt-sbi-A* terminator** [7]
gaattcgcggccgcttctagagaaaaaaaaaagccccgcgattgcggggcttttttttttactagtagcggccgctgcag

***fd* phage terminator** [8]
gaattcgcggccgcttctagagtctaaagttttgtcgtctttccagacgttagtaaatgaattttctgtatgaggttttgctaaacaactttcaacagtttcagc
ggagtgagaatagaaaggaacaactaaaggaattgcgaataataatttttcacgttgaaaatctccaaaaaaaagggctccaaaaggagccttt
aattgtatcggtttatcagcttgctttcgaggtgaatttcttaaacagcttgataccgatagttgcgccgacaatgacaacaaccatcgcccacgcataa
ccgatatattcggtcgctgaggcttgcagggagtcaaaggccgcttttgcgggatactagtagcggccgctgcag

ECK120010818-term [9]
gaattcgcggccgcttctagaggtcagtttcacctgttttacgtaaaaacccgcttcggcgggtttttacttttggtactagtagcggccgctgcag

ECK120029600-spy-term [9]
gaattcgcggccgcttctagagttcagccaaaaaacttaagaccgccggtcttgtccactaccttgcagtaatgcggtggacaggatcggcggttttct
tttctcttctcaatactagtagcggccgctgcag

L3S3P41-term [9]
gaattcgcggccgcttctagagaaaaaaaaaaaaacaccctaacgggtgttttttttttttggtctccctactagtagcggccgctgcag

L3S2P21-term [9]
gaattcgcggccgcttctagagctcggtaccaaattccagaaaagaggcctcccgaaagggggcctttttcgttttggtcctactagtagcggccg
ctgcag

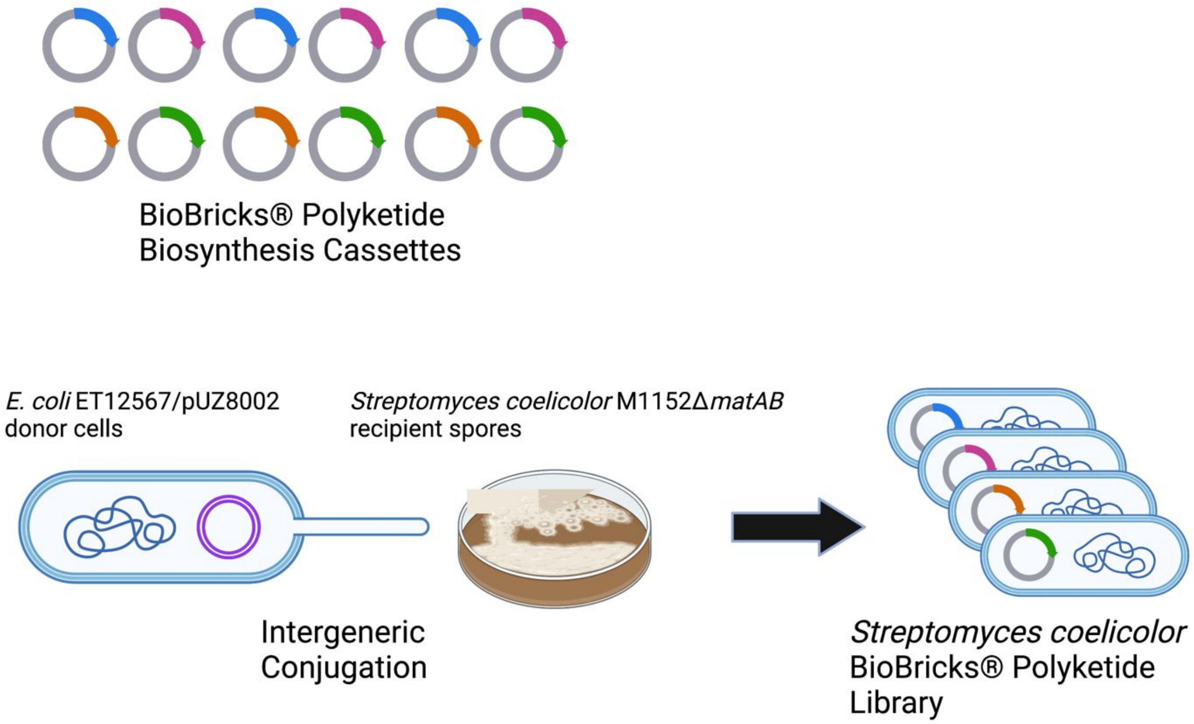

Figure S1. Overview of BIOPOLYMER metabolic engineering platform strategy**.**

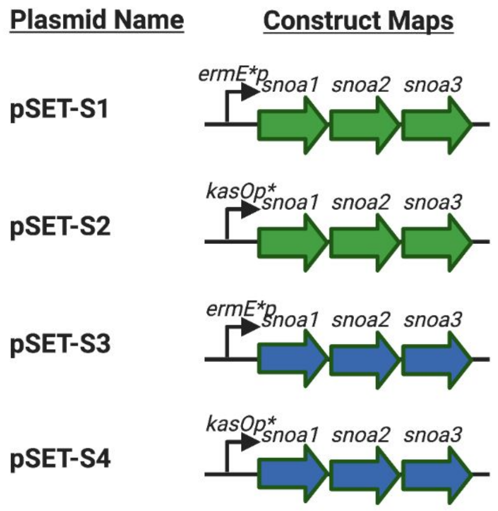

Figure S2. Plasmid maps for *snoa123* minPKS constructs.

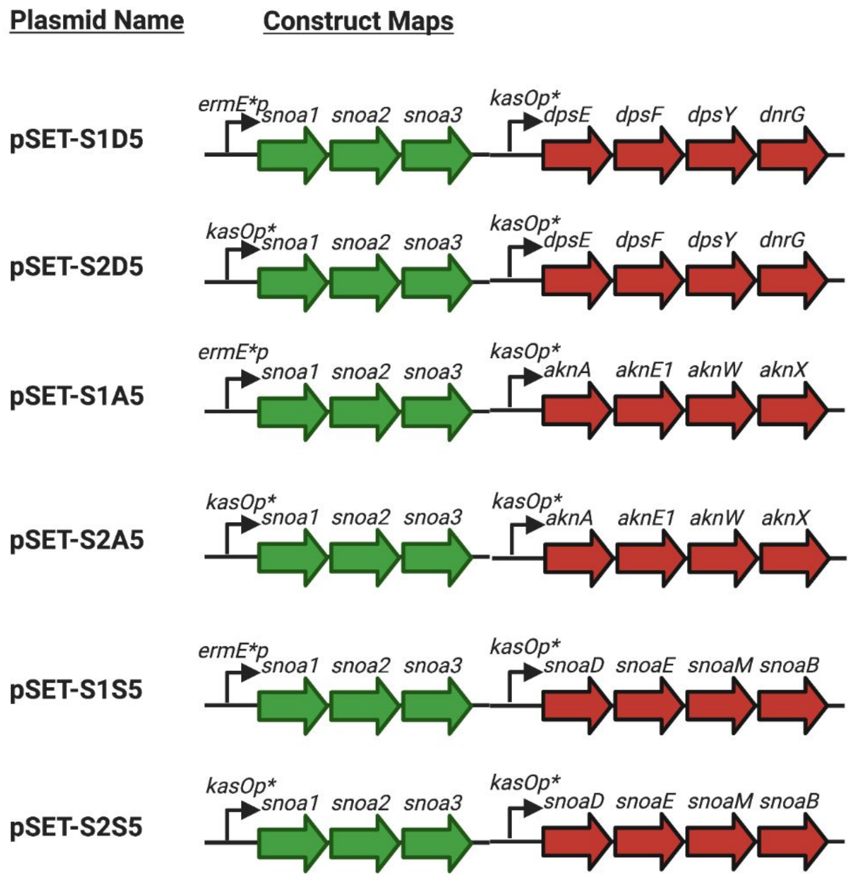

Figure S3. Plasmid maps for *snoa123* minPKS (green) and KR/ARO/CYC/OXY constructs (red).

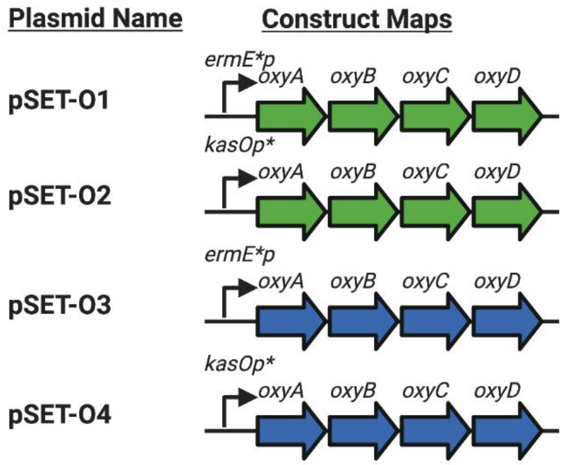

Figure S4. Plasmid maps for *oxyABCD* minPKS constructs. Green gene arrows correspond to wildtype sequences, and blue gene arrows correspond to codon-optimized sequences.

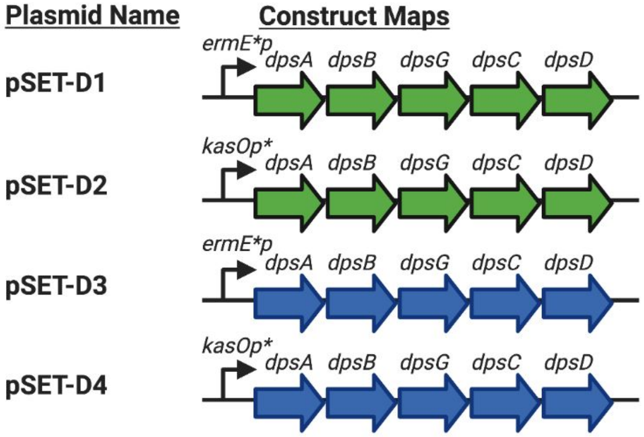

Figure S5. Plasmid maps for *dpsABGCD* minPKS constructs. Green gene arrows correspond to wildtype sequences, and blue gene arrows correspond to codon-optimized sequences.

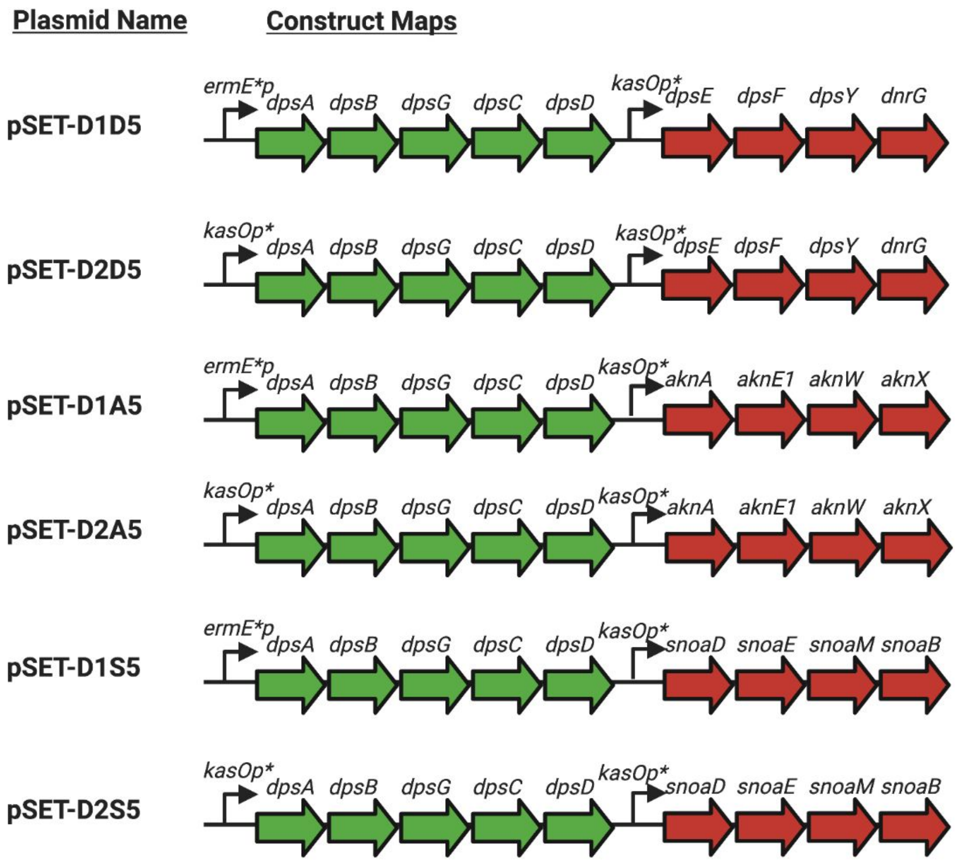

Figure S6. Plasmid maps for *dpsABCDG* minPKS (green) and KR/ARO/CYC/OXY constructs (red).

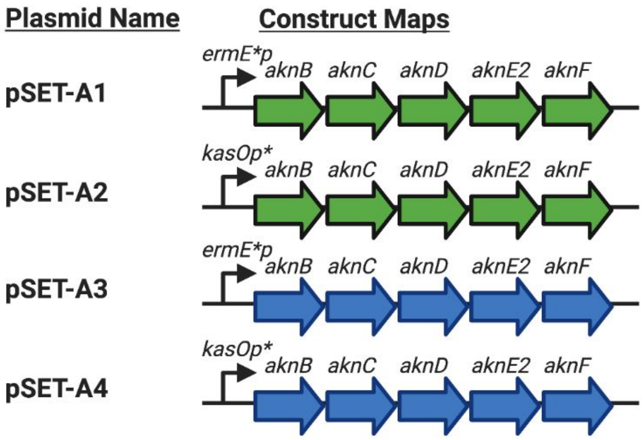

Figure S7. Plasmid maps for *aknBCDE2F* minPKS constructs.

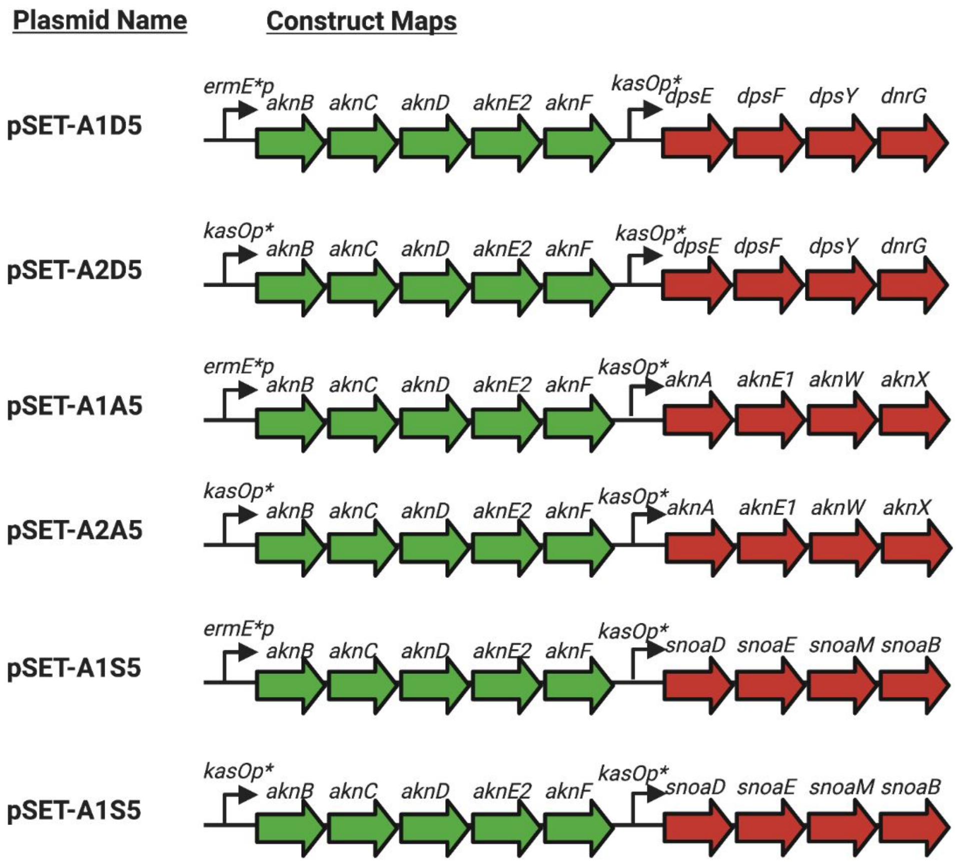

Figure S8. Plasmid maps for aknBCDE2F minPKS (green) and KR/ARO/CYC/OXY constructs (red).

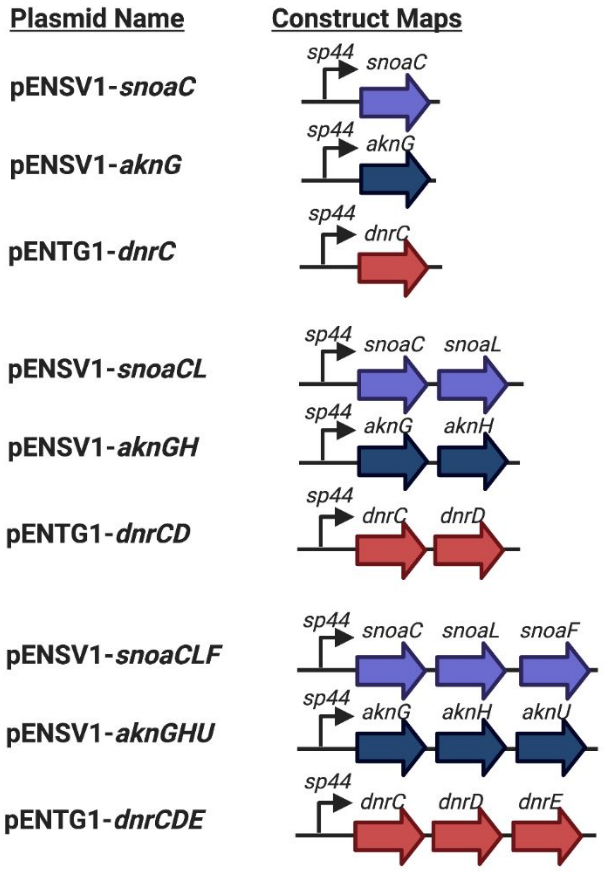

Figure S9. Plasmid maps for MT/CYC/KR plasmids.

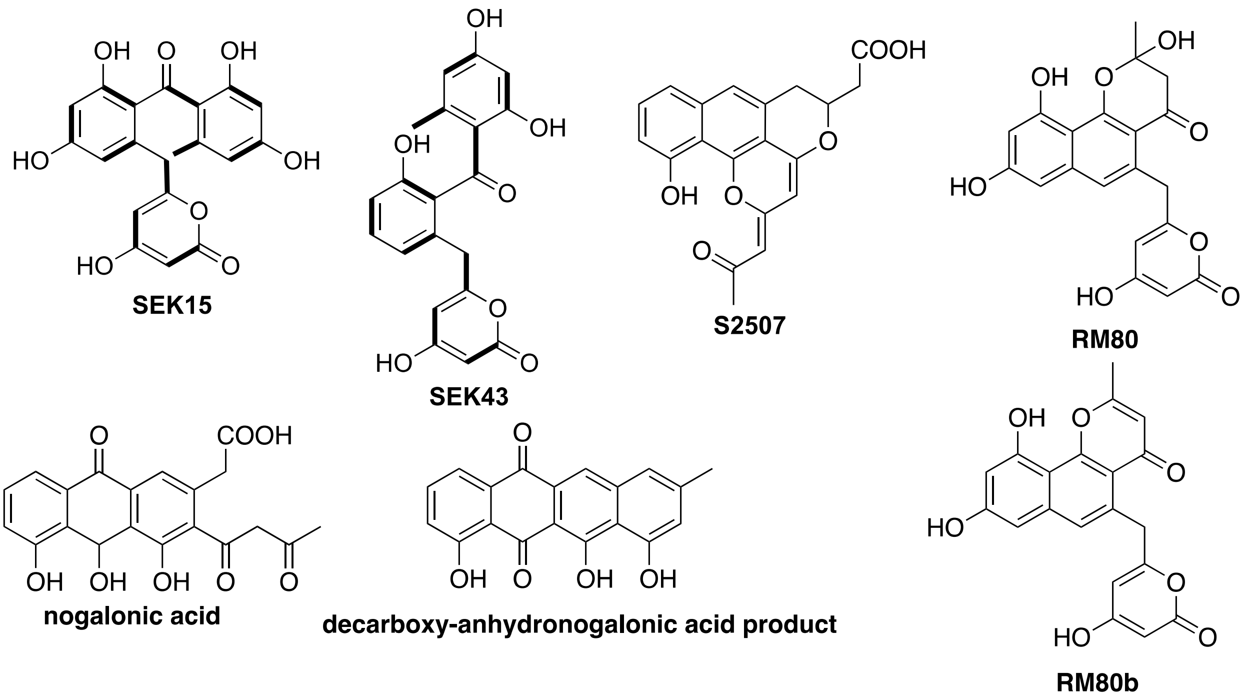

Figure S10. Shunt products detected from nogalonic acid engineering experiments.

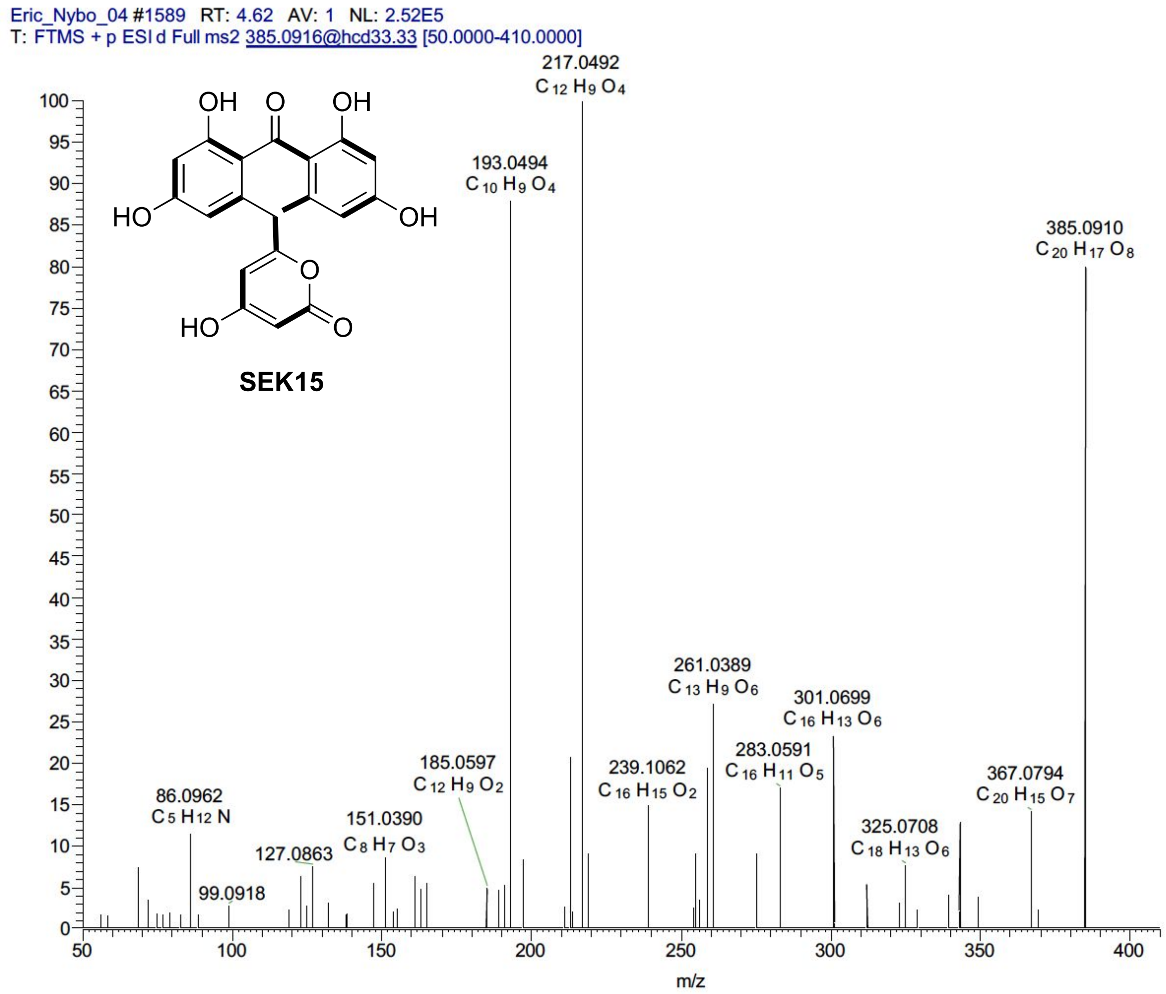

Figure S11. High-resolution ESI-MS/MS mass spectrum of SEK15. (+)-HRESIMS *m/z* 385.0910 $[M + H]^+$ (calc. for $C_{20}H_{17}O_8$, 385.0923).

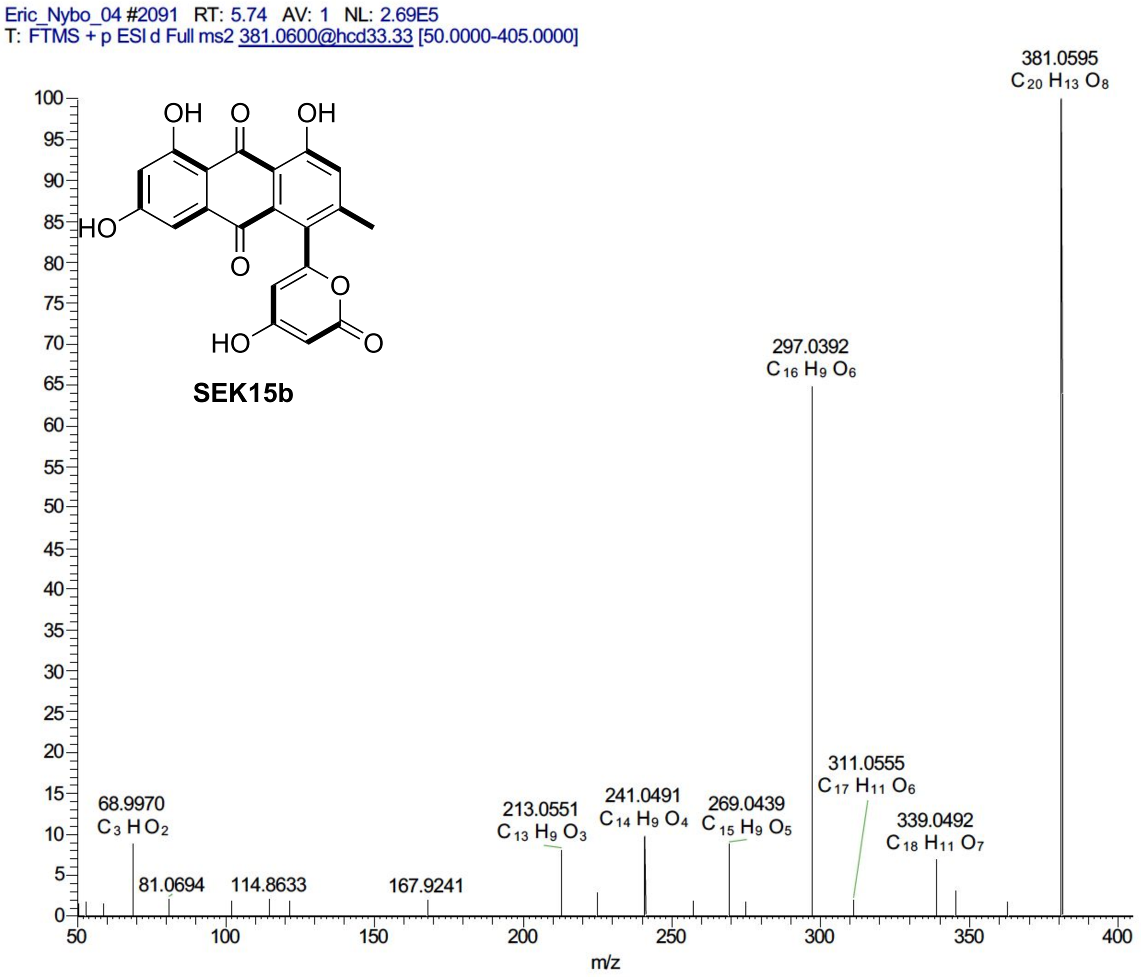

Figure S12. High-resolution ESI-MS/MS mass spectrum of SEK15b. (+)-HRESIMS *m/z* 381.0595 $[M + H]^+$ (calc. for $C_{20}H_{13}O_8$, 381.0610).

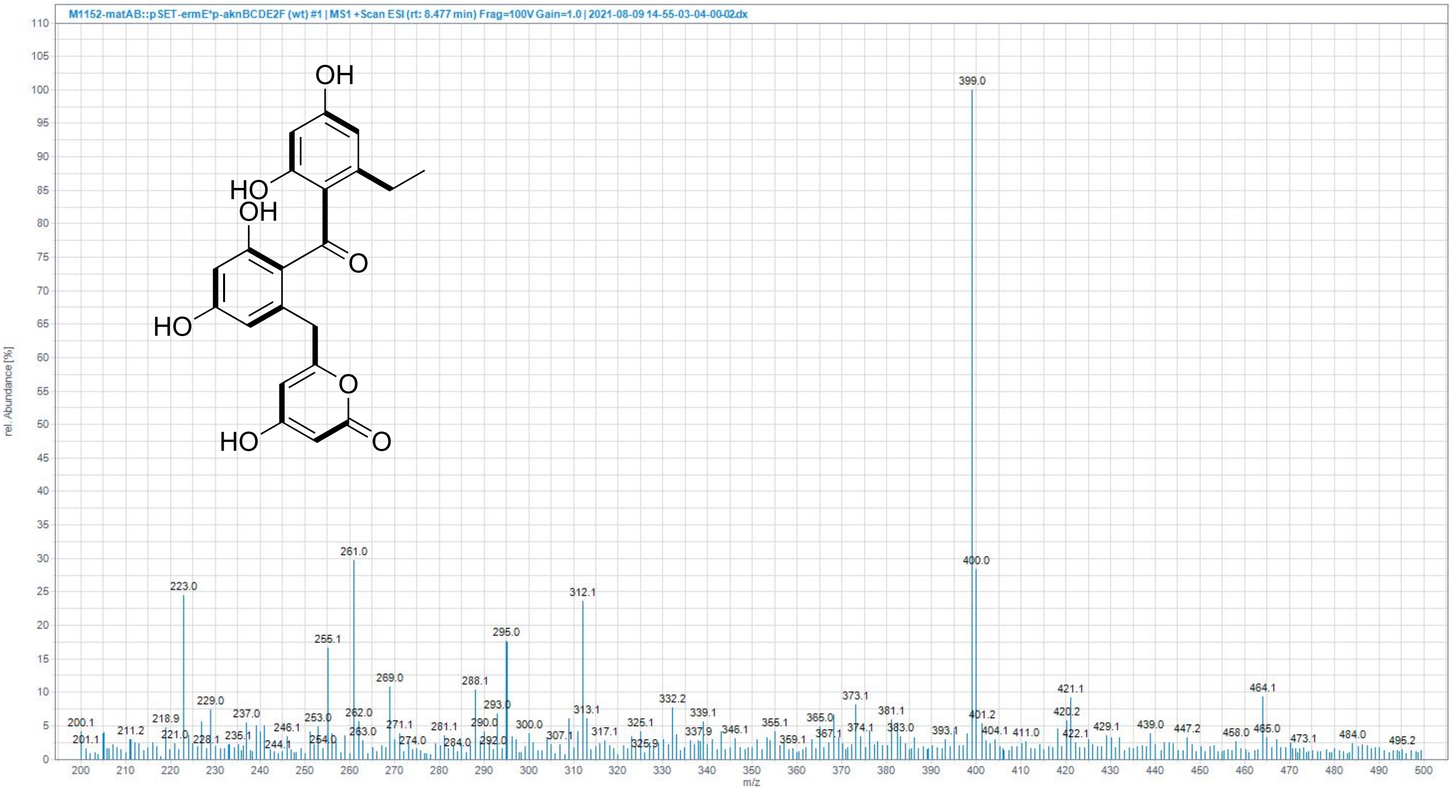

Figure S13. ESI-MS positive mode mass spectrum of UWM7 $[M + H]^{+}$ = 399 *m/z*.

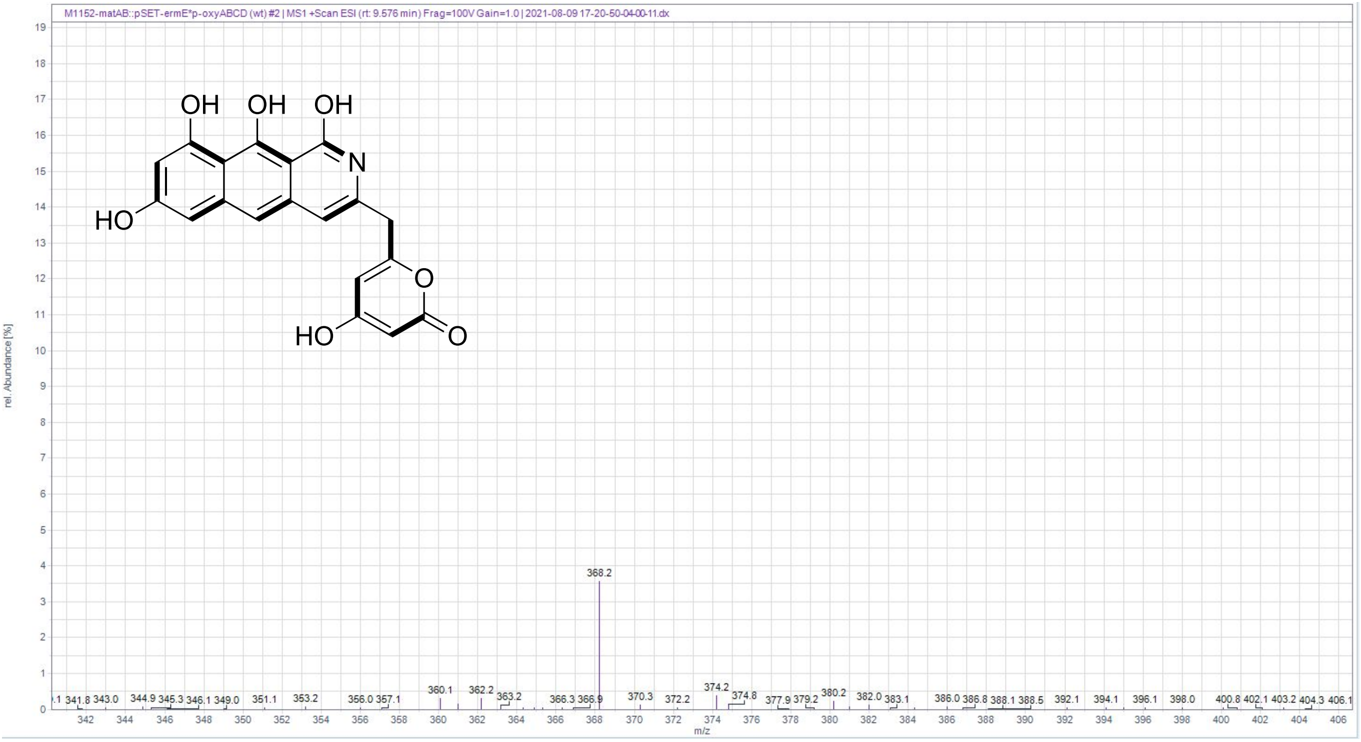

Figure S14. ESI-MS positive mode mass spectrum of WJ85 $[M + H]^+$ = 368 *m/z*.

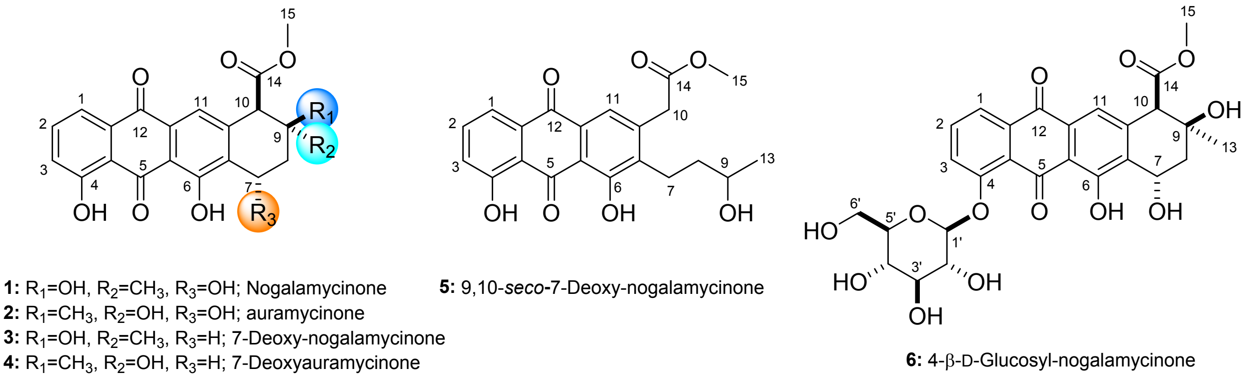

Figure S15: Chemical structures of compounds 1-6.

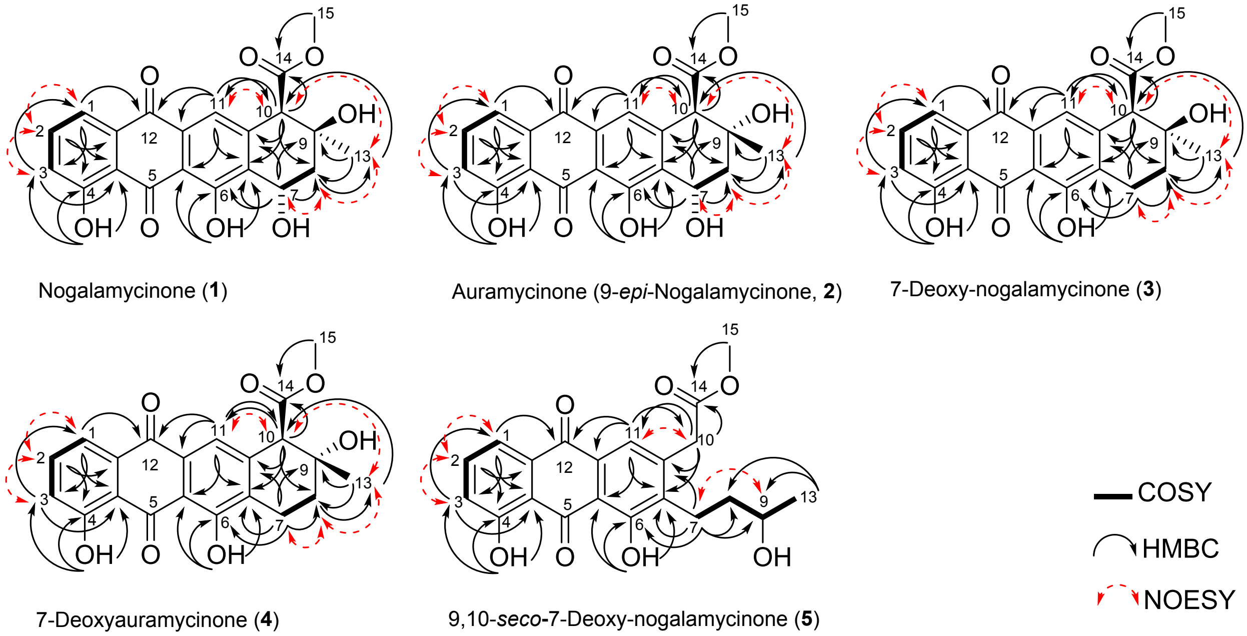

Figure S16: ^{1}H,^{1}H-COSY (▬), selected HMBC (→) and NOESY (⌒) correlations of compounds **1-5**.

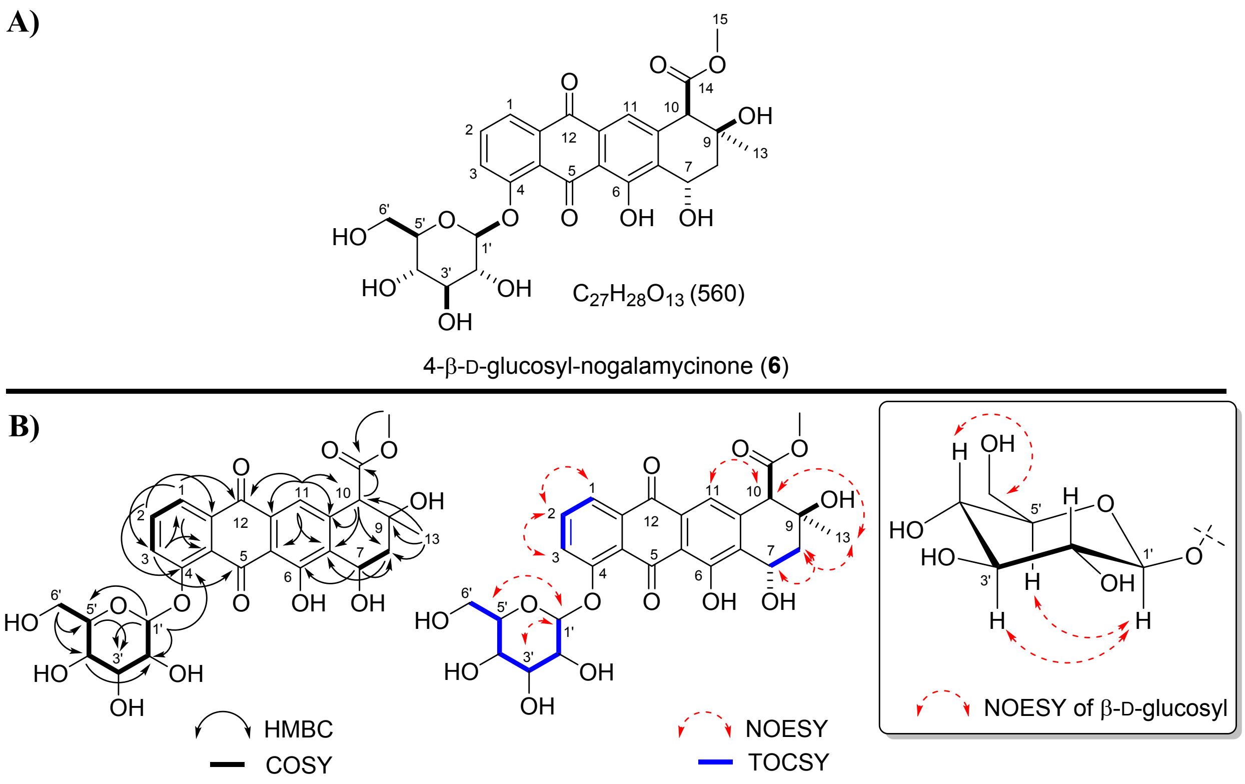

Figure S17: Selected 2D-NMR homonuclear and heteronuclear correlations for compound **6.** a) Chemical structure of 4-β-D-glucosyl-nogalamycinone (**6**). b) $^{1}H,^{1}H$-COSY (▬), selected HMBC (→), TOCSY (▬) and selected NOESY (⤺⤻) correlations of compound **6**.

Table S3. ^{13}C (150 MHz) NMR Spectroscopic Data of Compounds **1-5** in $CDCl_3$ (δ in ppm).

	1	2	3	4	5*
Position	δC, type	δC, type	δC, type	δC, type	δC, type
1	120.5, CH	120.5, CH	120.2, CH	120.2, CH	120.2, CH
2	137.8, CH	137.7, CH	137.4, CH	137.3, CH	137.5, CH
3	125.1, CH	125.1, CH	124.8, CH	124.8, CH	124.8, CH
4	162.9, C	162.9, C	162.7, C	162.7, C	162.8, C
4a	115.9, C	116.0, C	116.1, C	116.2, C	116.1, C
5	193.1, C	193.2, C	193.1, C	193.1, C	193.2, C
5a	114.9, C	114.8, C	113.9, C	113.8, C	114.5, C
6	161.2, C	161.7, C	161.1, C	161.2, C	161.3, C
6a	134.4, C	134.4, C	133.7, C	133.8, C	138.0, C
7	64.4, CH	64.1, CH	21.7, CH2	20.4, CH2	22.9, CH2
8	41.1, CH2	39.4, CH2	32.5, CH2	31.1, CH2	38.1, CH2
9	70.8, C	71.8, C	69.5, C	69.9, C	67.4, CH
10	56.2, CH	58.2, CH	56.2, CH	57.7, CH	39.3, CH2
10a	142.3, C	142.7, C	142.3, C	142.2, C	142.6, C
11	120.1, CH	121.5, CH	120.7, CH	121.4, CH	122.6, CH
11a	132.5, C	132.4, C	131.1, C	131.0, C	131.2, C
12	181.4, C	181.5, C	181.8, C	181.9, C	181.8, C
12a	133.7, C	133.7, C	133.9, C	134.0, C	133.9, C
13	28.9, CH3	28.5, CH3	27.9, CH3	27.7, CH3	23.7, CH3
14	173.4, C	171.1, C	173.4, C	171.9, C	170.9, C
15	53.0, CH3	53.0, CH3	52.9, CH3	52.8, CH3	52.7, CH3

See Supplementary Information for NMR spectra. Assignments supported by 2D HSQC and HMBC experiments.
*Atom numbering based on compound 1 for better comparison.

Table S4. ^{1}H (600 MHz) NMR Spectroscopic Data of Compounds **1-5** in $CDCl_3$ (δ in ppm).

Position	1	2	3	4	5*
	δH (mult, *J* in [Hz])	δH (mult, *J* in [Hz])	δH (mult, *J* in [Hz])	δH (mult, *J* in [Hz])	δH (mult, *J* in [Hz])
1	7.80 (d, 7.4)	7.82 (dd, 7.5, 1.3)	7.80 (dd, 7.5, 1.2)	7.80 (dd, 7.5, 1.1)	7.82 (dd, 7.5, 1.2)
2	7.68 (t, 7.9)	7.69 (t, 8.2)	7.66 (dd, 8.4, 7.5)	7.66 (dd, 8.4, 7.4)	7.67 (dd, 8.4, 7.5)
3	7.30 (d, 8.4)	7.30 (dd, 8.5, 1.2)	7.27 (dd, 8.4, 1.2)	7.27 (dd, 8.4, 1.1)	7.28 (dd, 8.4, 1.1)
4-OH	11.91 (s)	11.94 (s)	12.06 (s)	12.08 (s)	12.04 (s)
6-OH	12.82 (s)	12.92 (s)	12.46 (s)	12.50 (s)	12.63 (s)
7	5.39 (t, 7.0)	5.39 (dd, 8.5, 7.4)	3.09 (ddd, 19.1, 6.7, 1.1)<br>2.81 (dt, 19.1, 6.7)	3.03 (ddd, 19.1, 6.7, 3.0)<br>2.86 (ddd, 18.7, 10.3, 6.9)	2.90 (m, 2H)
8	2.65 (ddd, 13.9, 7.1, 1.6)<br>1.87 (ddd, 13.8, 7.0, 1.7)	2.43 (dd, 13.9, 8.5)<br>2.35 (m)	2.31 (dt, 13.5, 6.8)<br>1.79 (ddd, 13.4, 6.7, 1.1)	2.31 (m)<br>1.90 (ddd, 13.4, 6.7, 1.1)	1.76-1.62 (m, 2H)
9					3.80 (m)
10	3.92 (s)	3.87 (s)	3.85 (brs)	3.89 (s)	3.83 (brd, 7.3, 2H)
11	7.54 (s)	7.67 (s)	7.53 (s)	7.62 (s)	7.72 (s)
13	1.42 (s)	1.46 (s)	1.32 (s)	1.40 (s)	1.22 (d, 6.2)
15	3.88 (s)	3.74 (s)	3.84 (s)	3.73 (s)	3.71 (s)

See Supplementary Information for NMR spectra. Assignments supported by 2D HSQC and HMBC experiments.
*Atom numbering based on compound 1 for better comparison.

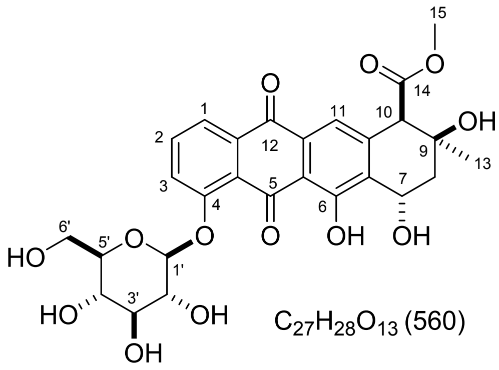

4-β-D-glucosyl-nogalamycinone (**6**)

Table S5. ^{13}C (150 MHz) and ^{1}H (600 MHz) NMR Spectroscopic Data of 4-β-D-glucosyl-nogalamycinone (**6**) in CD_3OD (δ in ppm).

Position	δC, type	δH (mult, J in [Hz])
1	123.0, CH	8.01 (dd, 7.4, 1.4)
2	137.4, CH	7.83 (dd, 8.5, 7.4)
3	125.1, CH	7.79 (dd, 8.5, 1.4)
4	160.2, C	
4a	123.0, C	
5	190.2, C	
5a	117.2, C	
6	163.1, C	
6a	134.6, C	
7	64.0, CH	5.34 (dd, 6.0, 2.7)
8	41.3, CH2	2.68 (dd, 14.0, 6.1)
		1.99 (ddd, 13.9, 2.6, 1.2)
9	70.6, C	
10	59.3, CH	4.03 (s)
10a	143.4, C	
11	120.1, CH	7.49 (s)
11a	133.2, C	
12	183.3, C	
12a	136.7, C	
13	29.9, CH3	1.45 (s)
14	173.5, C	
15	52.8, CH3	3.71 (s)
1’	103.5, CH	5.12 (d, 7.7)
2’	75.0, CH	3.69 (t, 7.9)
3’	77.8, CH	3.54 (t, 9.1)
4’	71.4, CH	3.45 (dd, 9.8, 8.9)
5’	78.8, CH	3.54 (m)
6’	62.7, CH2	3.94 (dd, 12.2, 6.0)
		3.73 (dd, 12.1, 2.3)

See Supplementary Information for NMR spectra. Assignments supported by 2D HSQC and HMBC experiments

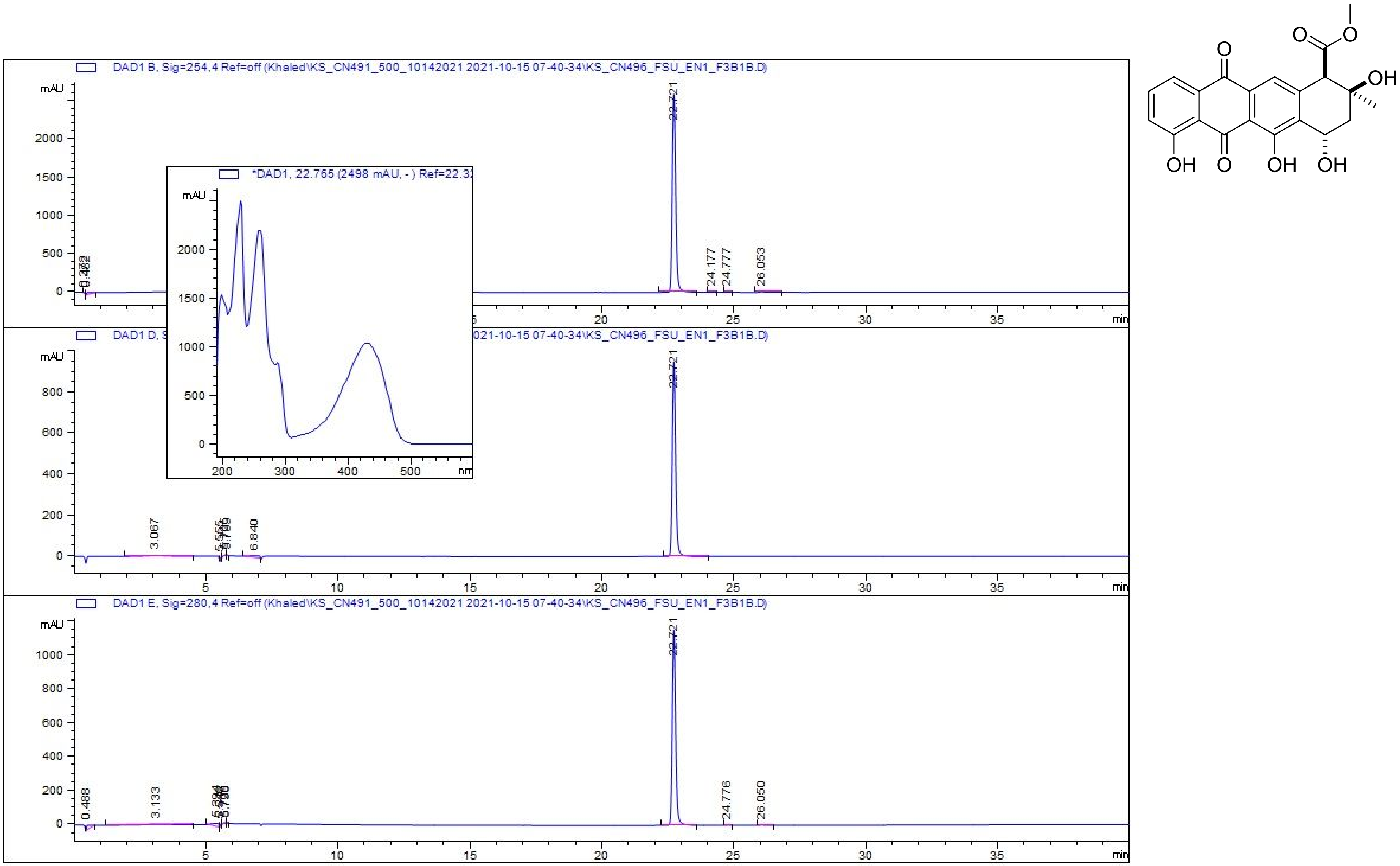

Figure S18. HPLC-UV/vis analysis of nogalamycinone **(1).** HPLC-conditions: solvent A: H2O/0.1% FA; solvent B: CH3CN; flow rate: 0.5 mL min-1; 0-30 min, 5-100% B; 30-35 min, 100% B; 35-36 min, 100-5% B; 36-40 min, 5% B; Phenomenex NX-C18 column (250 × 4.6 mm, 5 μm); 254 nm, 280 nm, 400 nm. UV-vis inset of full wavelength scan (190-600 nm).

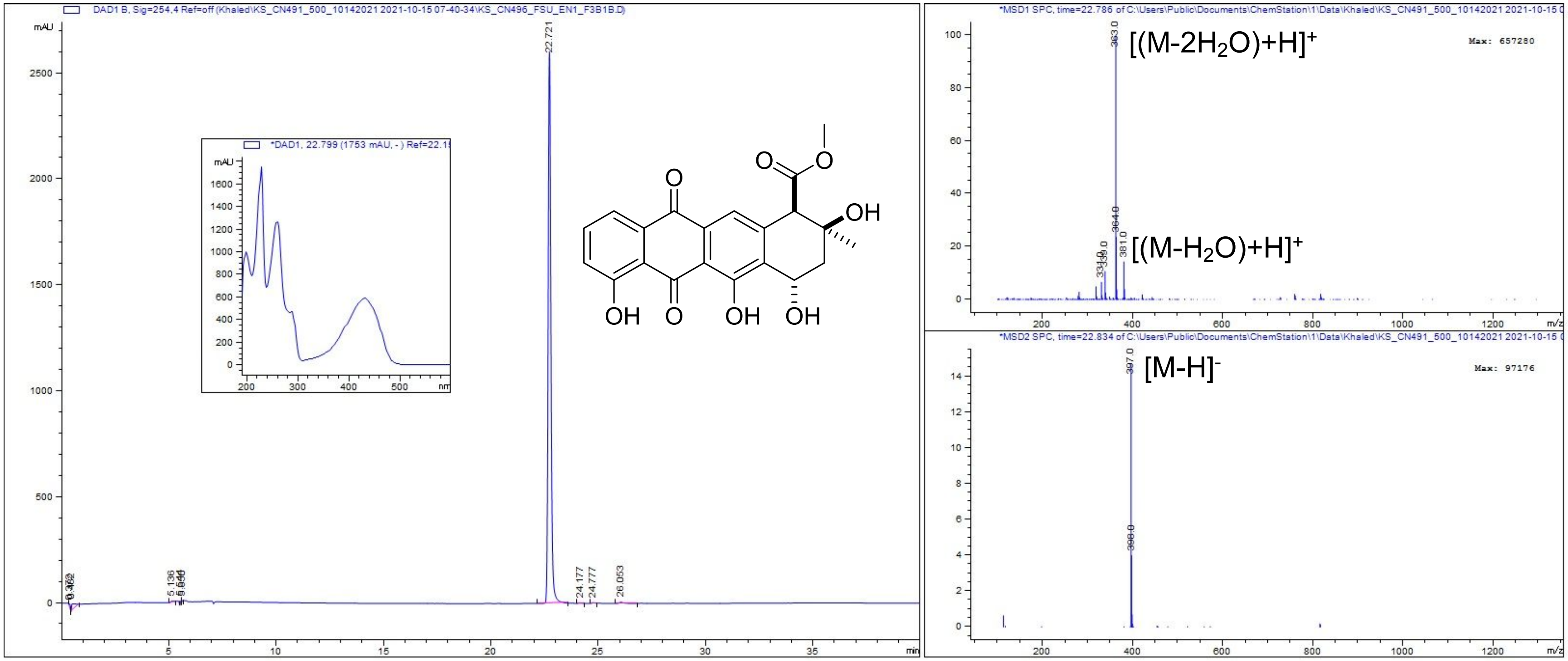

Figure S19. HPLC-MS analysis of nogalamycinone **(1).** HPLC-conditions: solvent A: H2O/0.1% FA; solvent B: CH3CN; flow rate: 0.5 mL min-1; 0-30 min, 5-100% B; 30-35 min, 100% B; 35-36 min, 100-5% B; 36-40 min, 5% B; Phenomenex NX-C18 column (250 × 4.6 mm, 5 µm); 254 nm. UV-vis inset of full wavelength scan (190-600 nm).

**Sample Name**	FSU_EN1_F3B1B	**Position**	P2-A2	**Instrument Name**	Instrument 1
**User Name**		**Inj Vol**	4	**InjPosition**	
**Sample Type**	Sample	**IRM Calibration Status**	Success	**Data Filename**	FSU_EN1_F3B1B.d
**ACQ Method**	Zheng_AQC ACC short_Neg.m	**Comment**		**Acquired Time**	9/21/2021 11:23:01 PM (UTC-04:00)

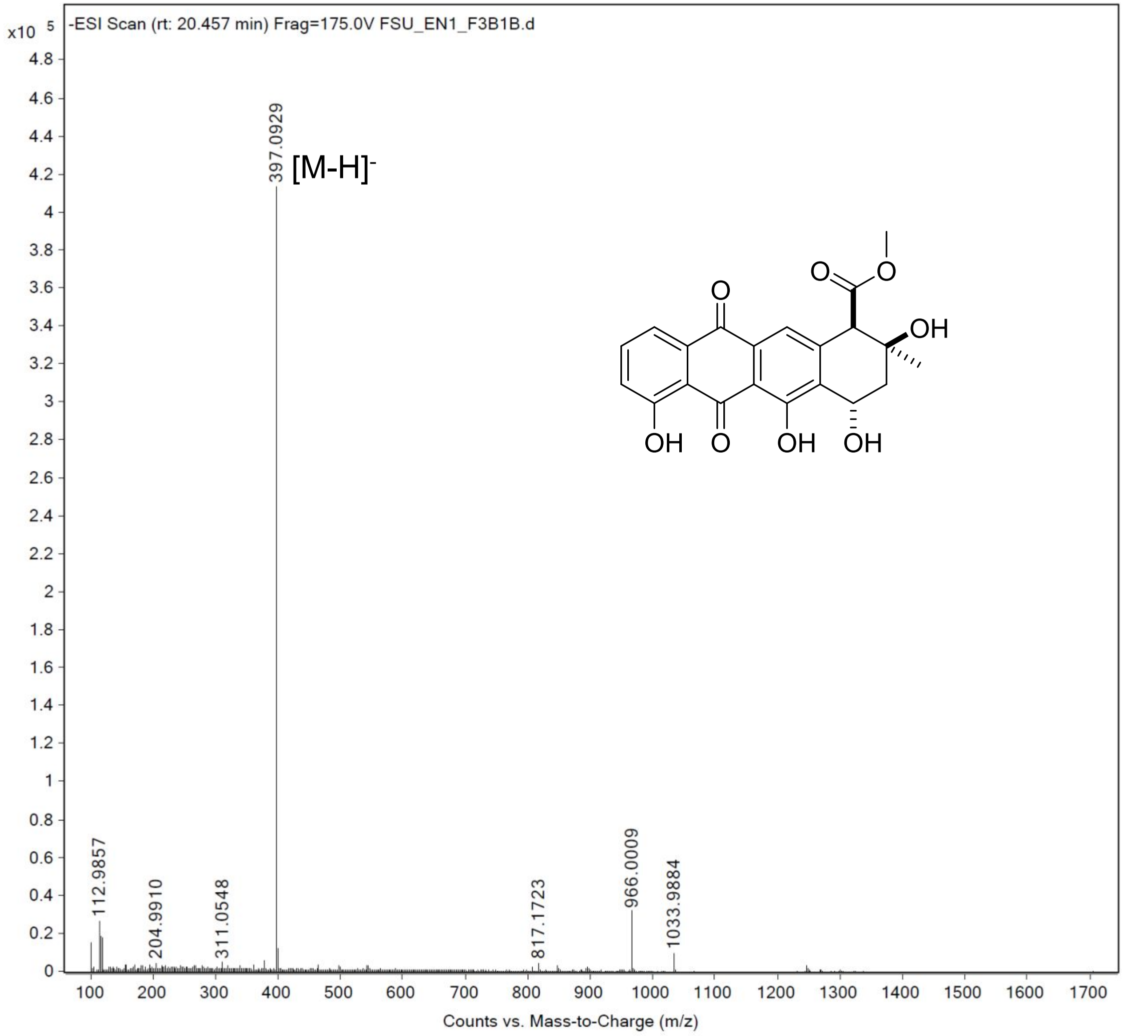

Figure S20. (–)-HRESI-MS spectrum of nogalamycinone **(1).**

**Sample Name**	FSU_EN1_F3B1B	**Position**	P2-A2	**Instrument Name**	Instrument 1
**User Name**		**Inj Vol**	4	**InjPosition**	
**Sample Type**	Sample	**IRM Calibration Status**	Success	**Data Filename**	FSU_EN1_F3B1B.d
**ACQ Method**	Zheng_AQC ACC short_Pos.m	**Comment**		**Acquired Time**	9/22/2021 8:46:45 PM (UTC-04:00)

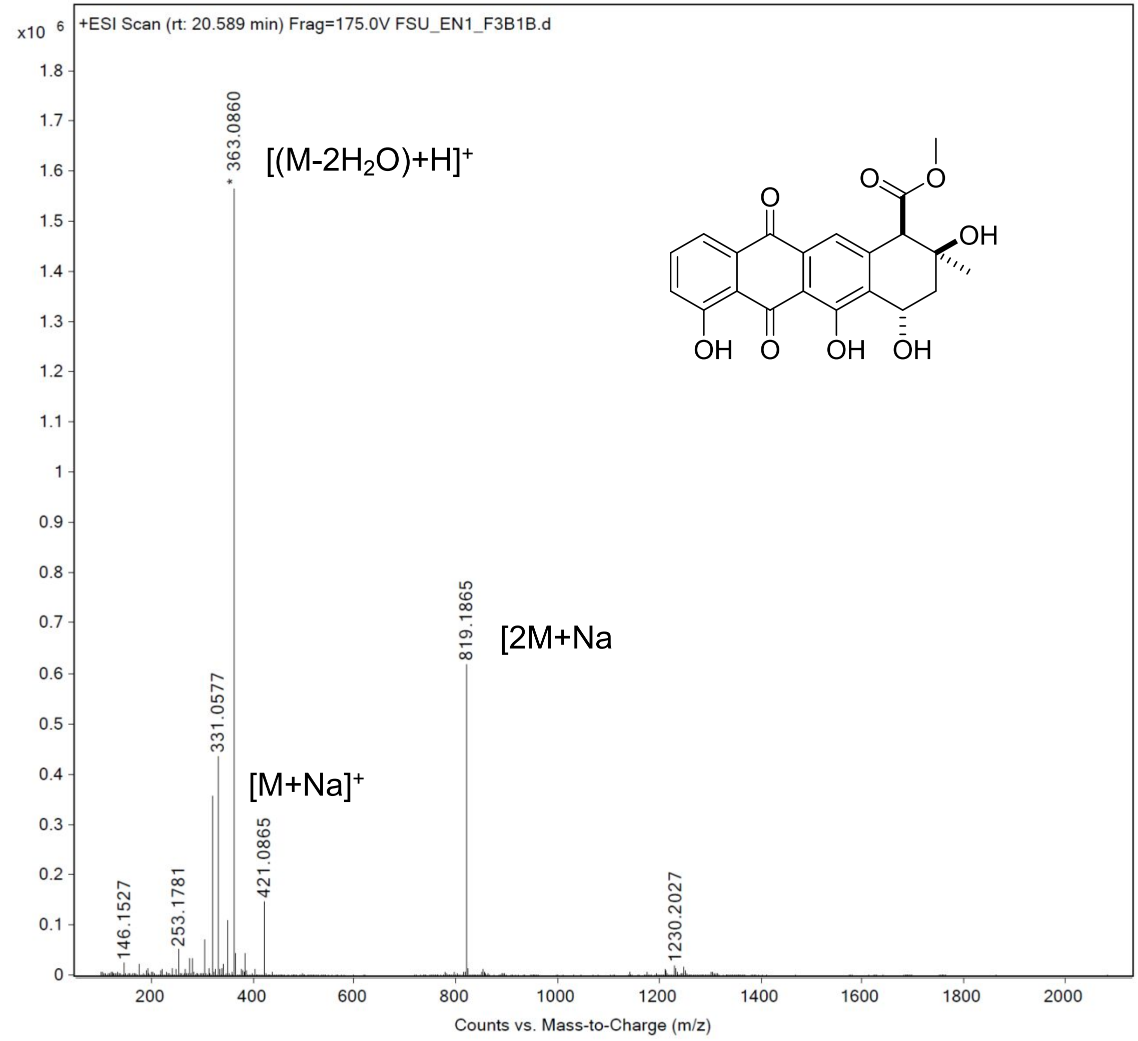

Figure S21. (+)-HRESI-MS spectrum of nogalamycinone **(1).**

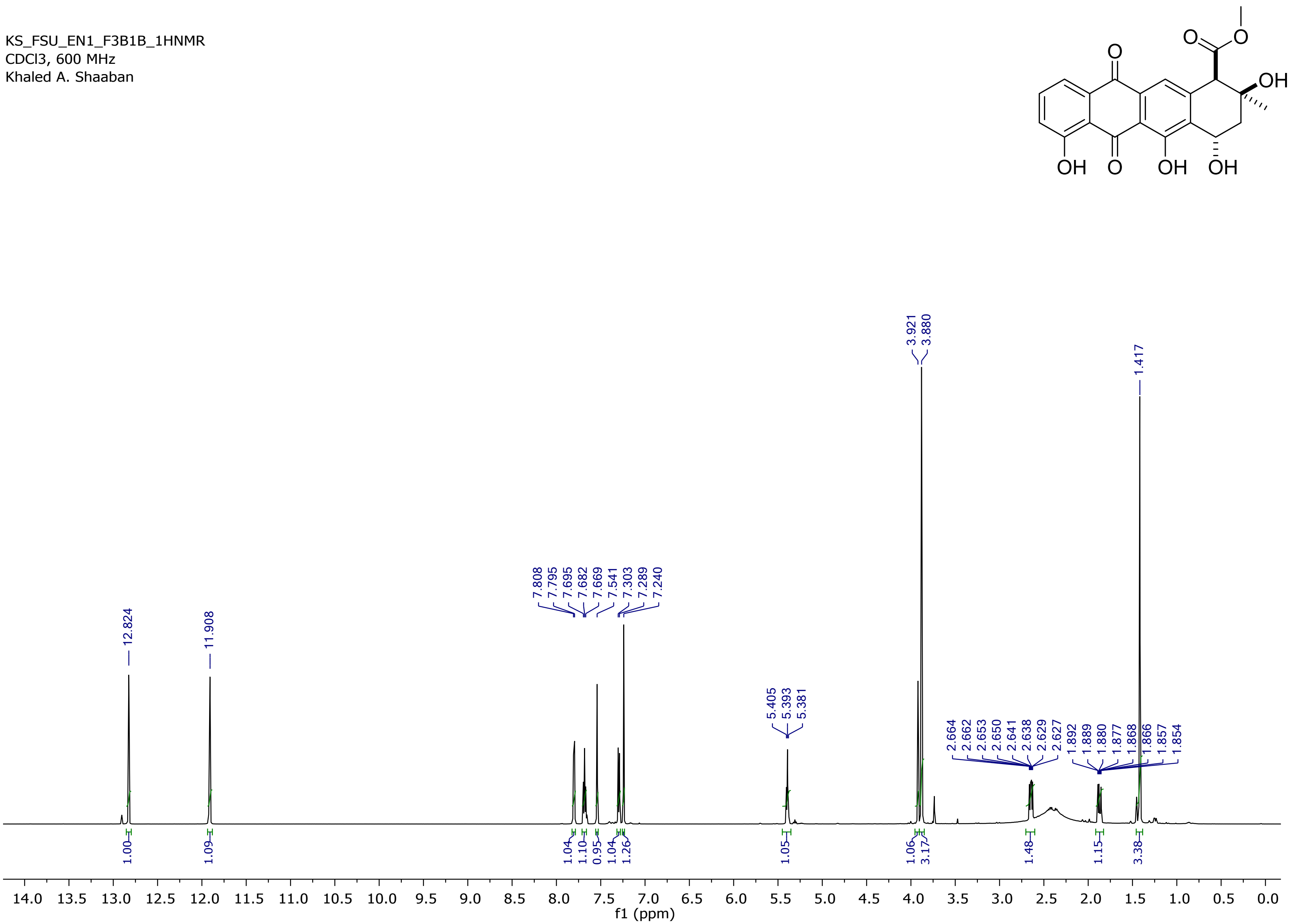

Figure S22. ^{1}H NMR spectrum ($CDCl_3$, 600 MHz) of nogalamycinone **(1).**

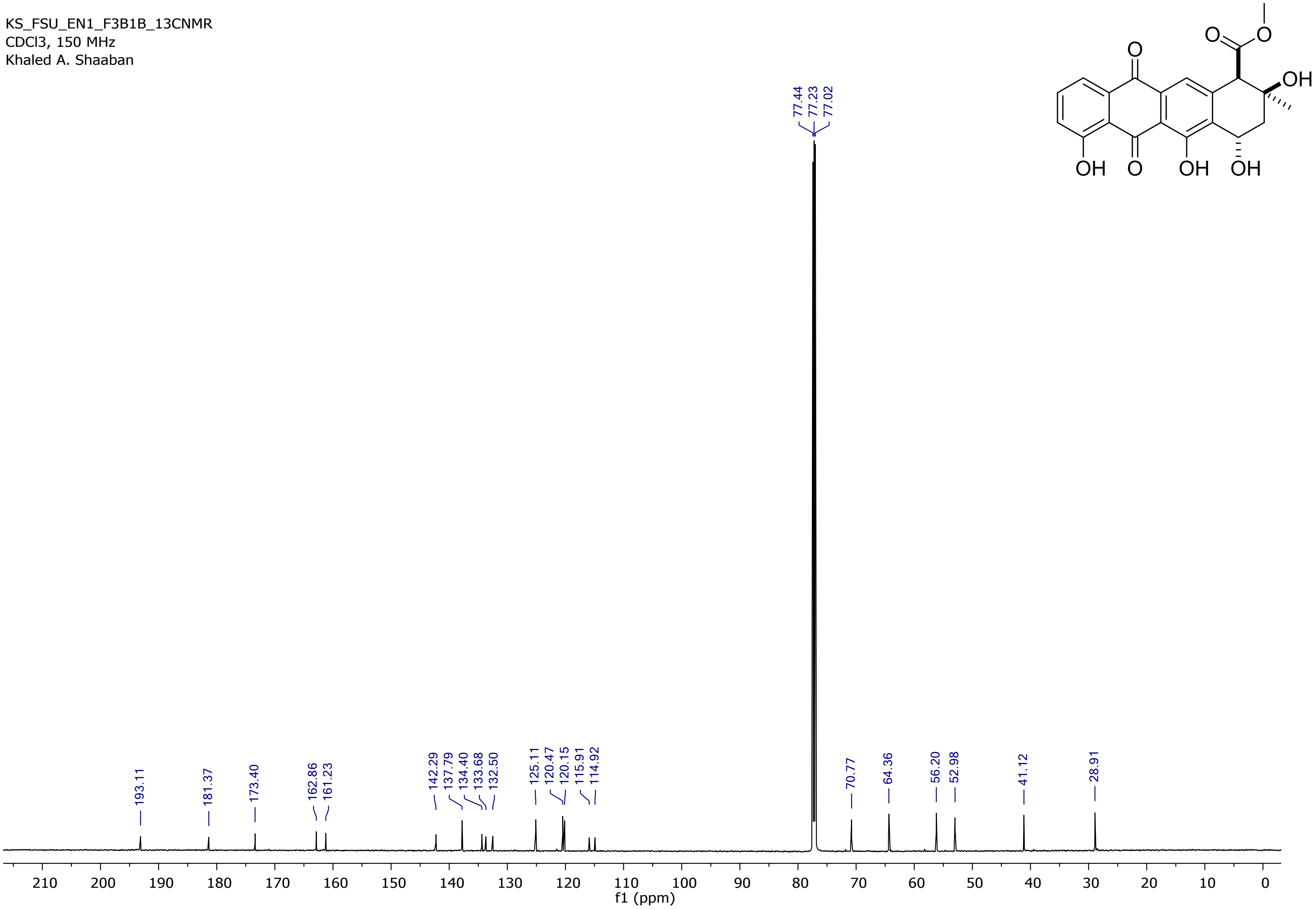

Figure S23. ^{13}C NMR spectrum ($CDCl_3$, 150 MHz) of nogalamycinone (**1**).

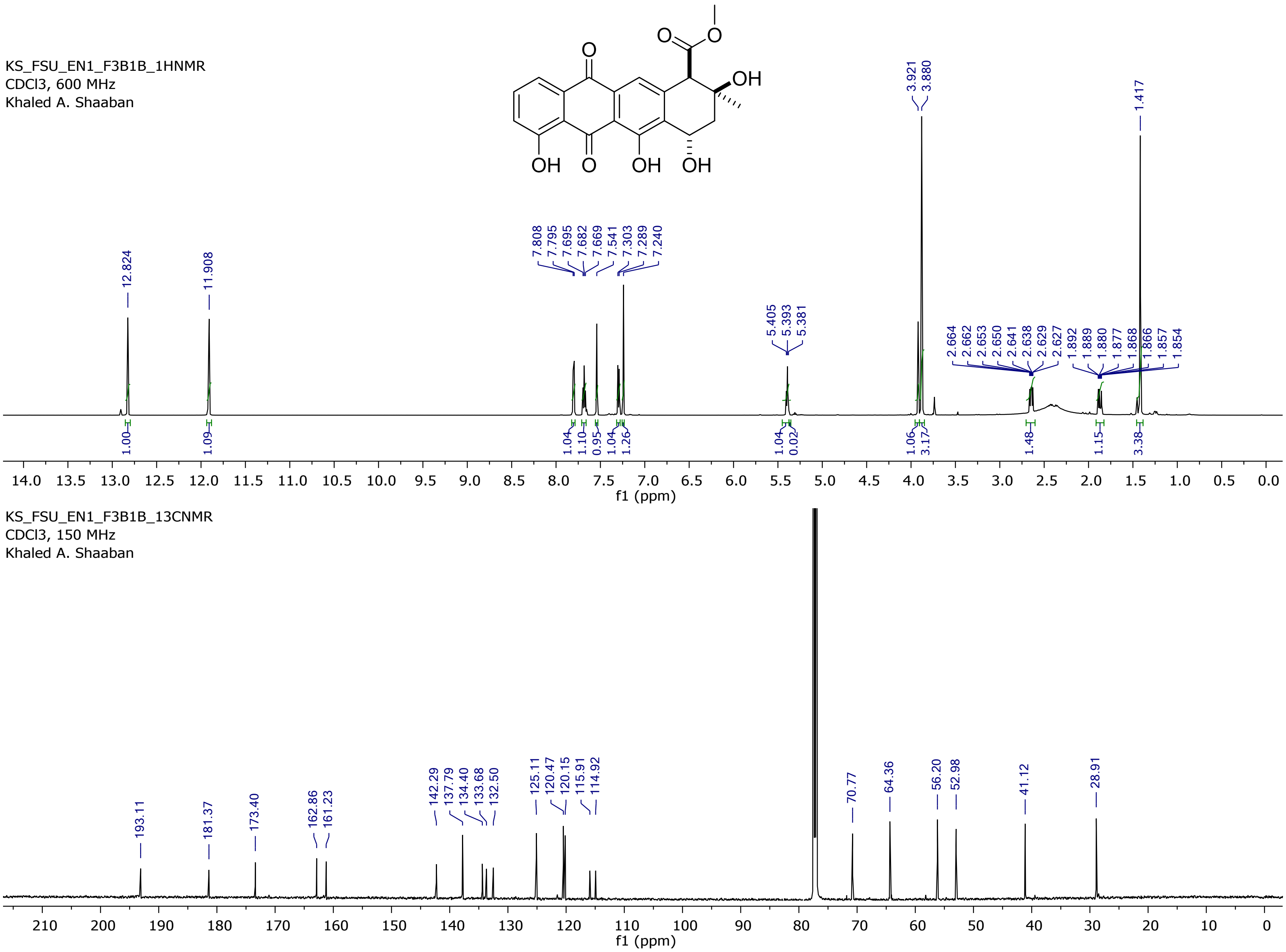

Figure S24. ^{1}H ($CDCl_3$, 600 MHz) and ^{13}C ($CDCl_3$, 150 MHz) NMR spectra of nogalamycinone **(1).**

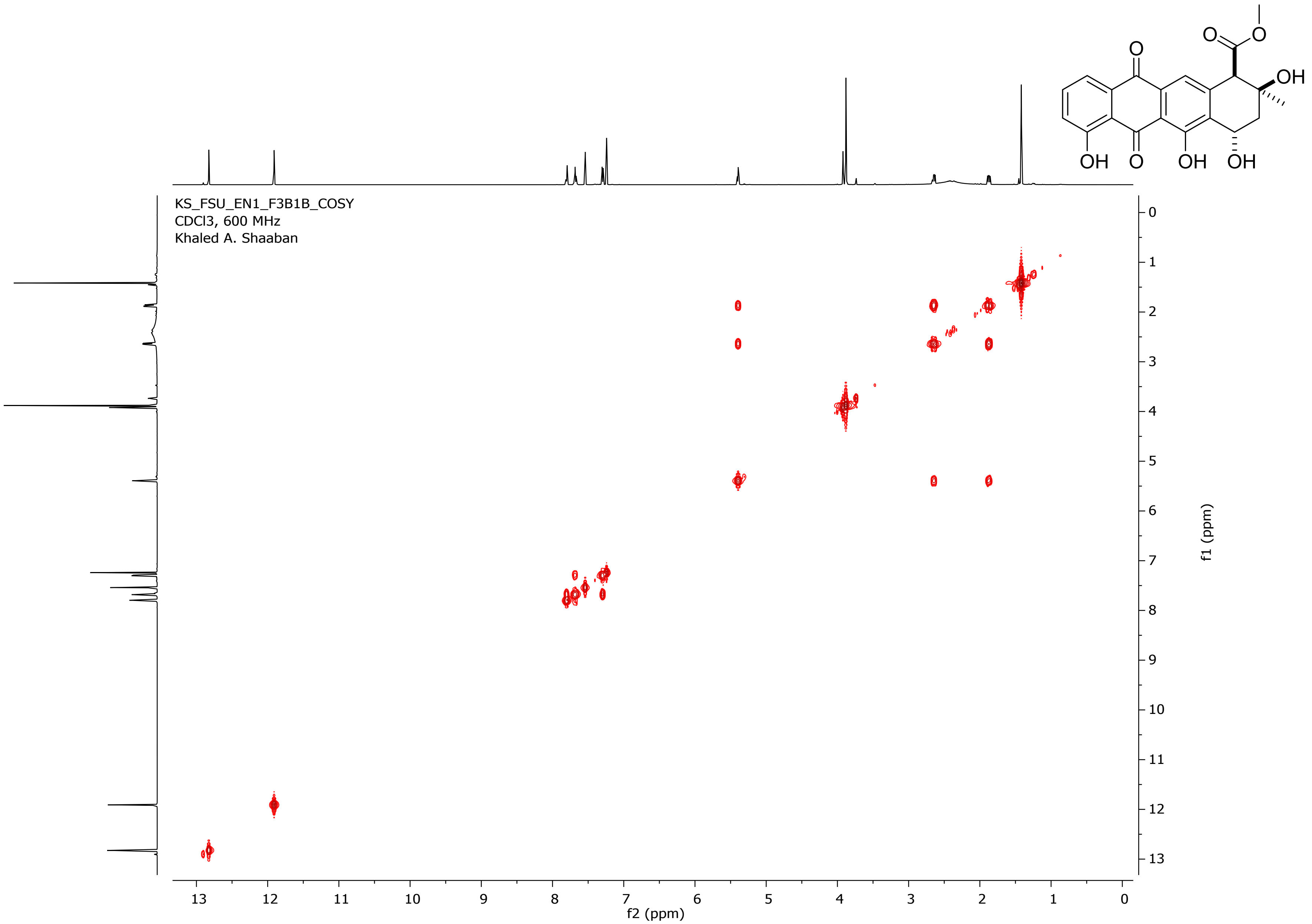

Figure S25. $^{1}H,^{1}H$-COSY spectrum ($CDCl_3$, 600 MHz) of nogalamycinone **(1).**

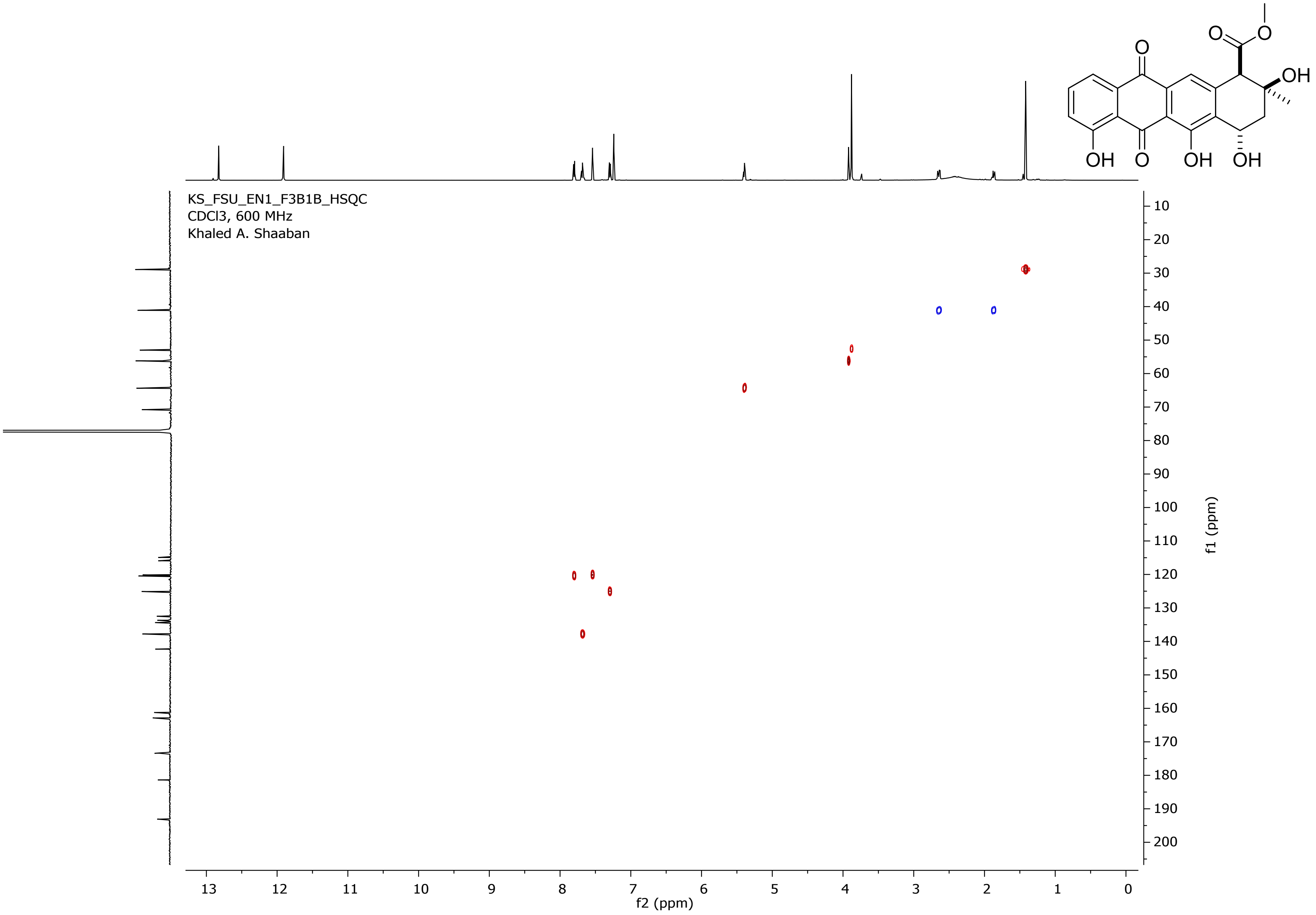

Figure S26. HSQC spectrum ($CDCl_3$, 600 MHz) of nogalamycinone **(1).**

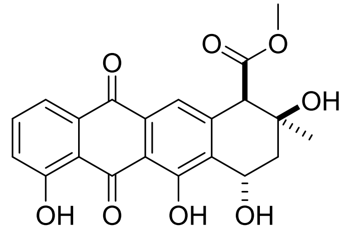

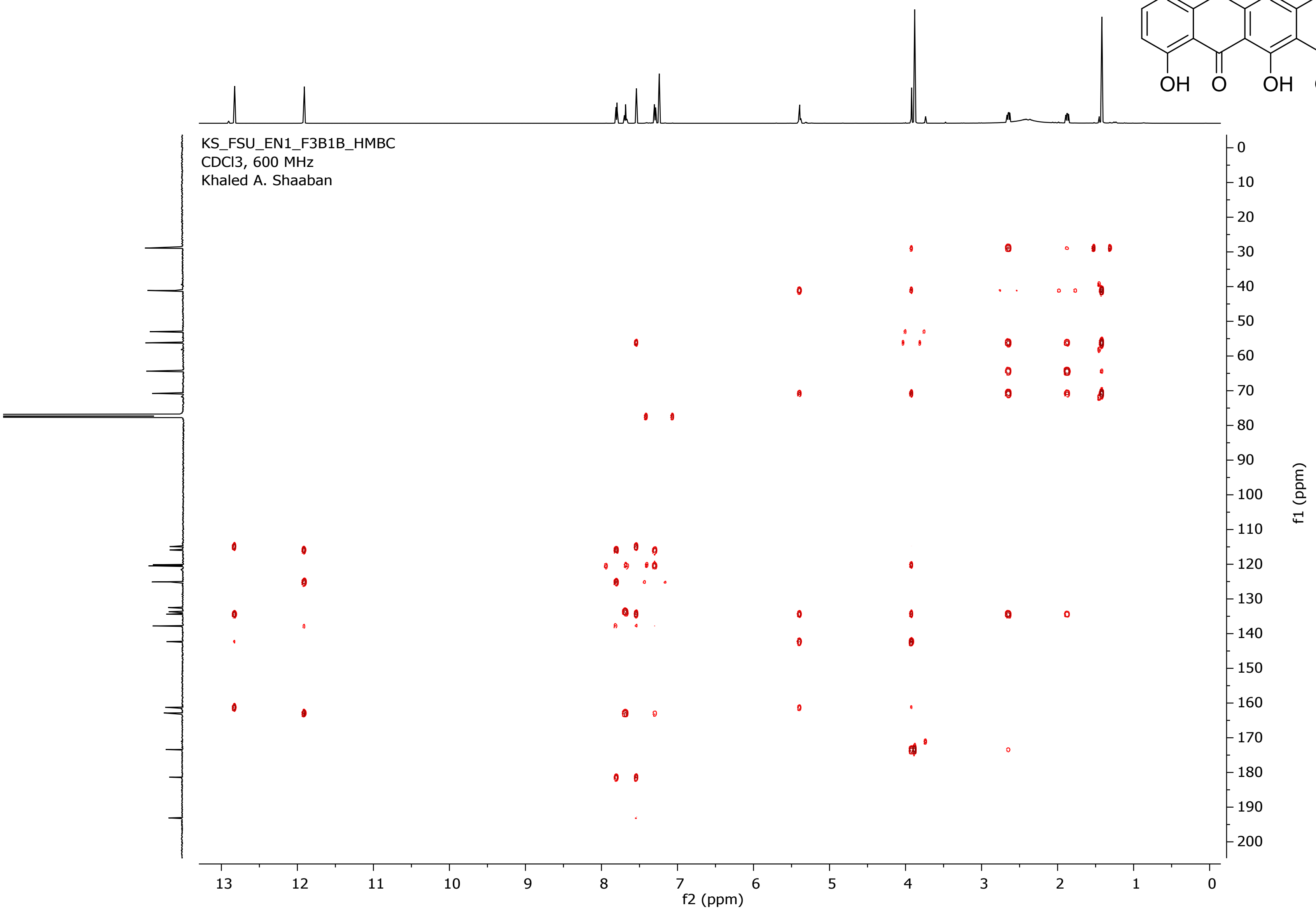

Figure S27. HMBC spectrum ($CDCl_3$, 600 MHz) of nogalamycinone **(1).**

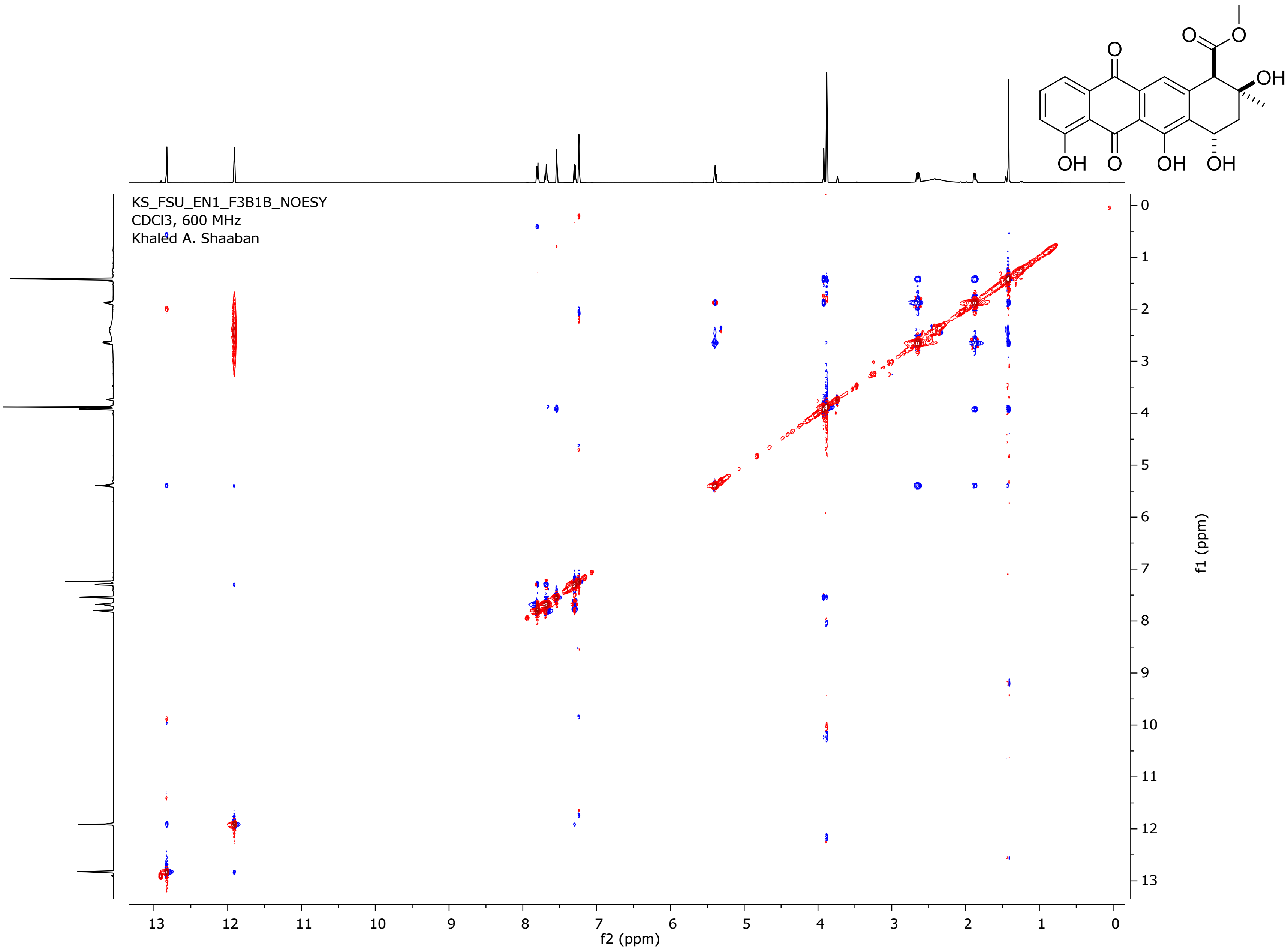

Figure S28. NOESY spectrum ($CDCl_3$, 600 MHz) of nogalamycinone **(1).**

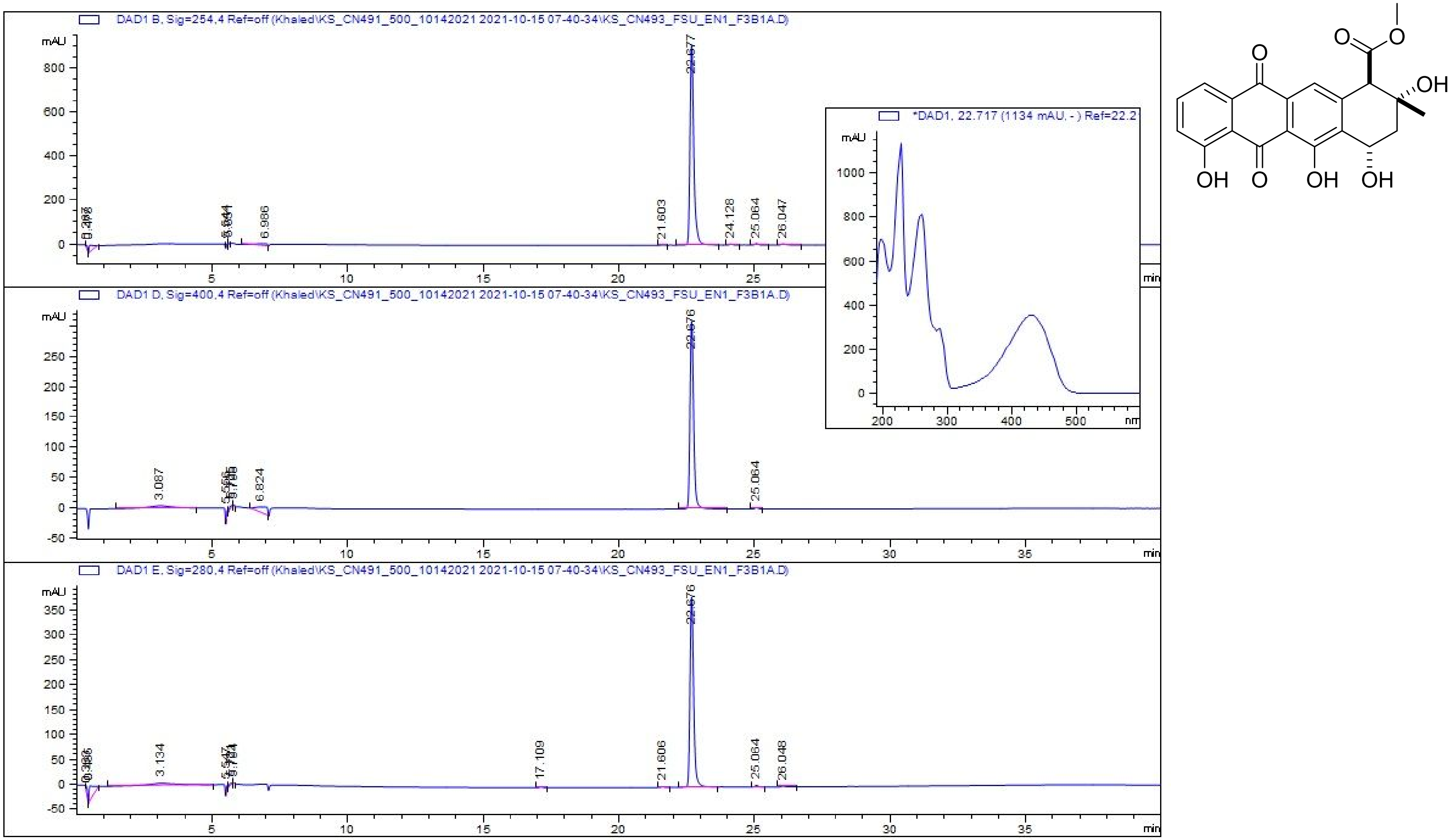

Figure S29: HPLC-UV/vis analysis of auramycinone (9-epi-nogalamycinone; **2).** HPLC-conditions: solvent A: H2O/0.1% FA; solvent B: CH3CN; flow rate: 0.5 mL min-1; 0-30 min, 5-100% B; 30-35 min, 100% B; 35-36 min, 100-5% B; 36-40 min, 5% B; Phenomenex NX-C18 column (250 × 4.6 mm, 5 µm); 254 nm, 280 nm, 400 nm. UV-vis inset of full wavelength scan (190-600 nm).

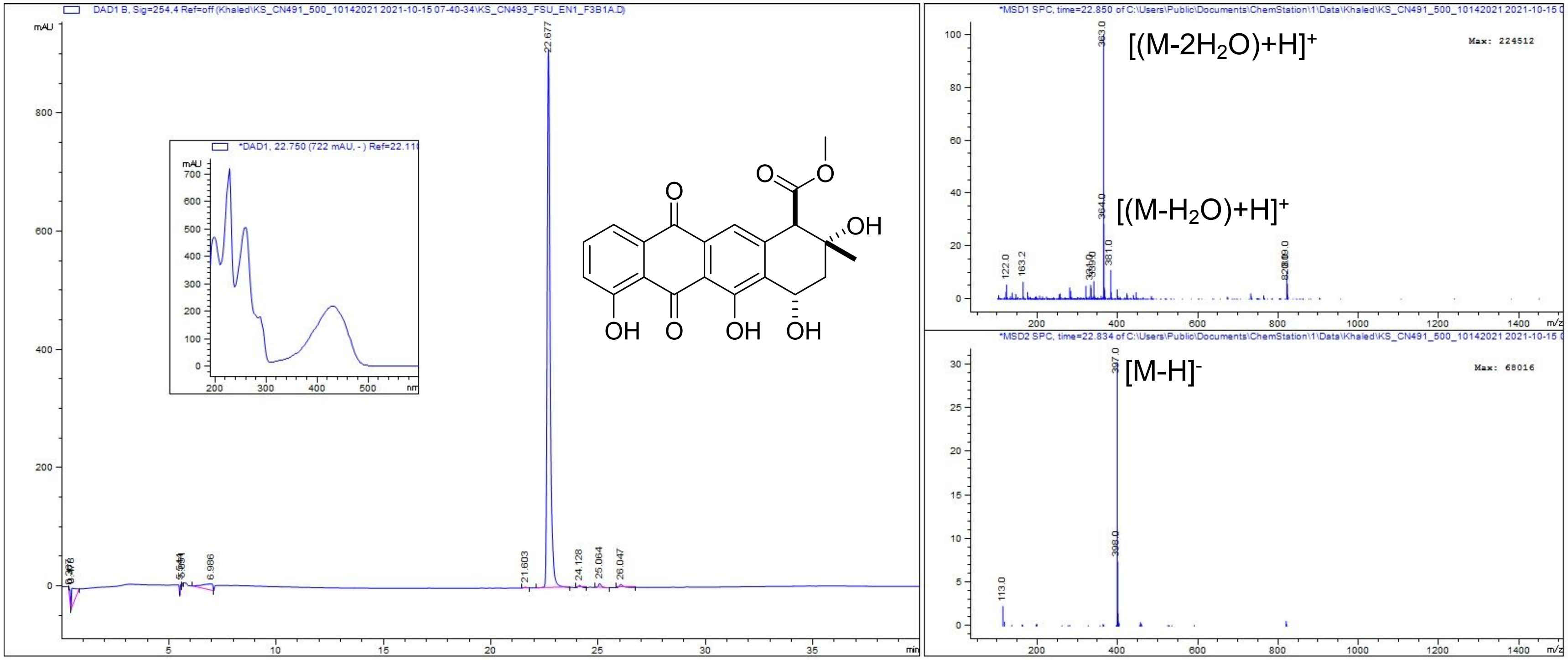

Figure S30: HPLC-MS analysis of auramycinone (9-epi-nogalamycinone; **2).** HPLC-conditions: solvent A: H2O/0.1% FA; solvent B: CH3CN; flow rate: 0.5 mL min-1; 0-30 min, 5-100% B; 30-35 min, 100% B; 35-36 min, 100-5% B; 36-40 min, 5% B; Phenomenex NX-C18 column (250 × 4.6 mm, 5 µm); 254 nm. UV-vis inset of full wavelength scan (190-600 nm).

**Sample Name**	FSU_EN1_F3B1A	**Position**	P2-A1	**Instrument Name**	Instrument 1
**User Name**		**Inj Vol**	4	**InjPosition**	
**Sample Type**	Sample	**IRM Calibration Status**	Success	**Data Filename**	FSU_EN1_F3B1A.d
**ACQ Method**	Zheng_AQC ACC short_Neg.m	**Comment**		**Acquired Time**	9/21/2021 10:54:03 PM (UTC-04:00)

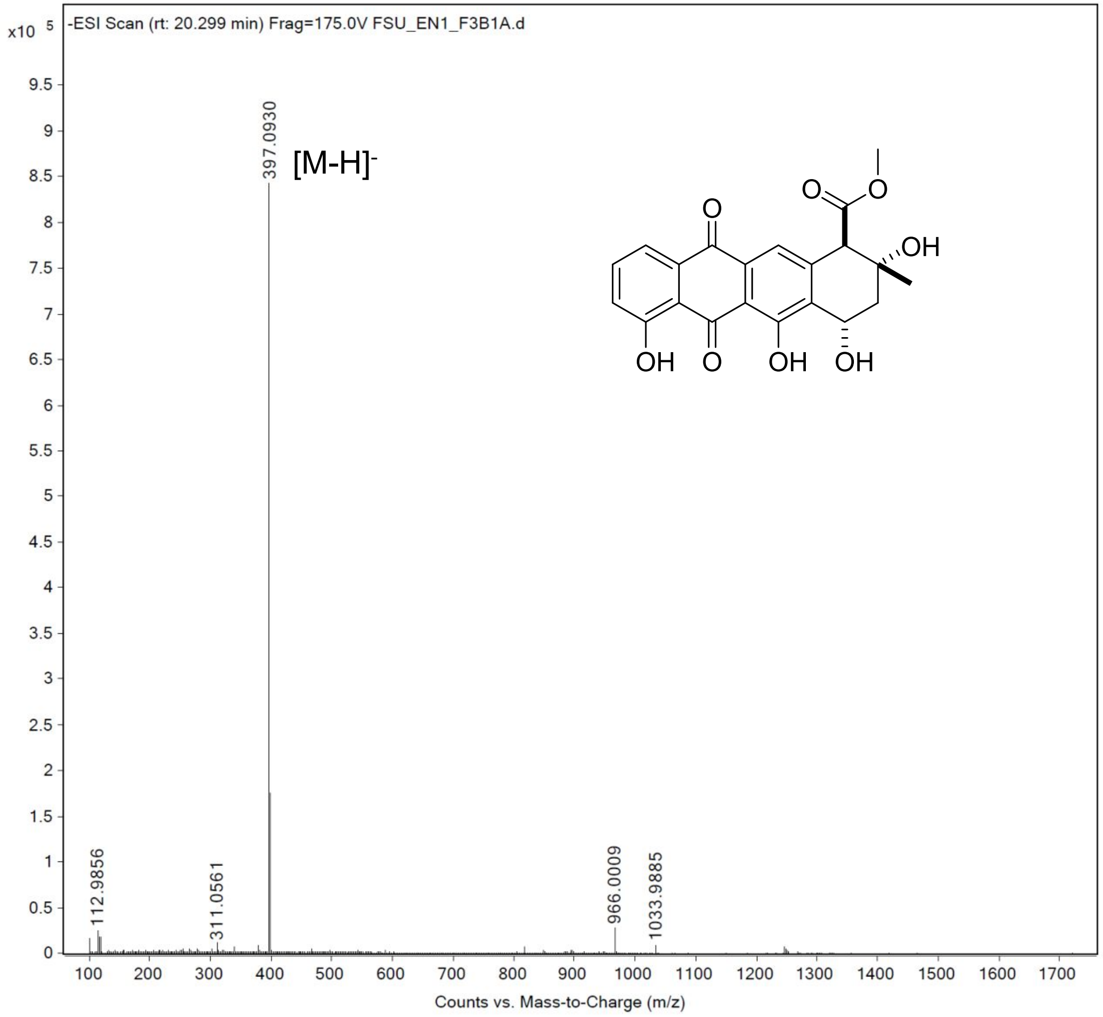

Figure S31. (–)-HRESI-MS spectrum of auramycinone (9-epi-nogalamycinone; **2**).

**Sample Name**	FSU_EN1_F3B1A	**Position**	P2-A1	**Instrument Name**	Instrument 1
**User Name**		**Inj Vol**	4	**InjPosition**	
**Sample Type**	Sample	**IRM Calibration Status**	Success	**Data Filename**	FSU_EN1_F3B1A.d
**ACQ Method**	Zheng_AQC ACC short_Pos.m	**Comment**		**Acquired Time**	9/22/2021 8:17:47 PM (UTC-04:00)

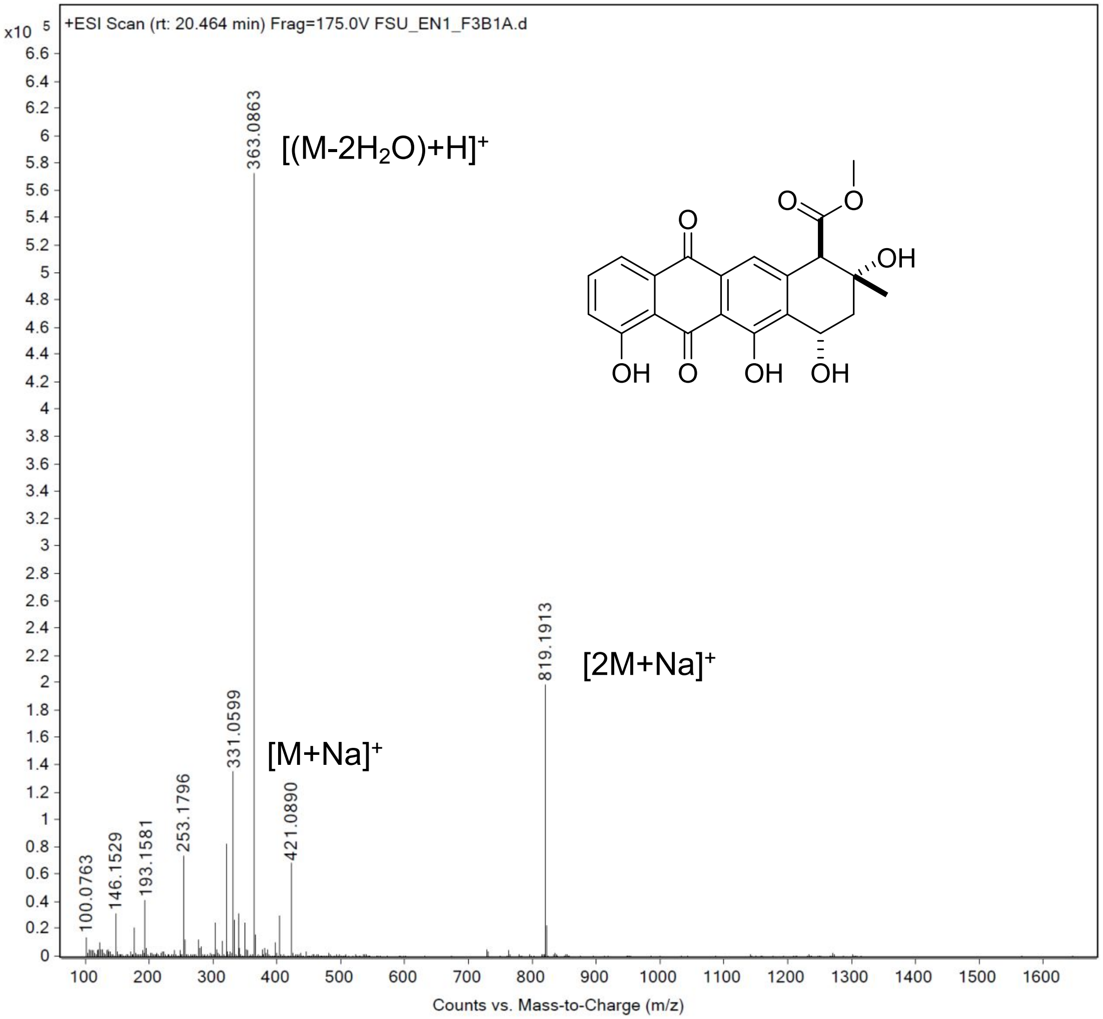

Figure S32. (+)-HRESI-MS spectrum of auramycinone (9-epi-nogalamycinone; 2).

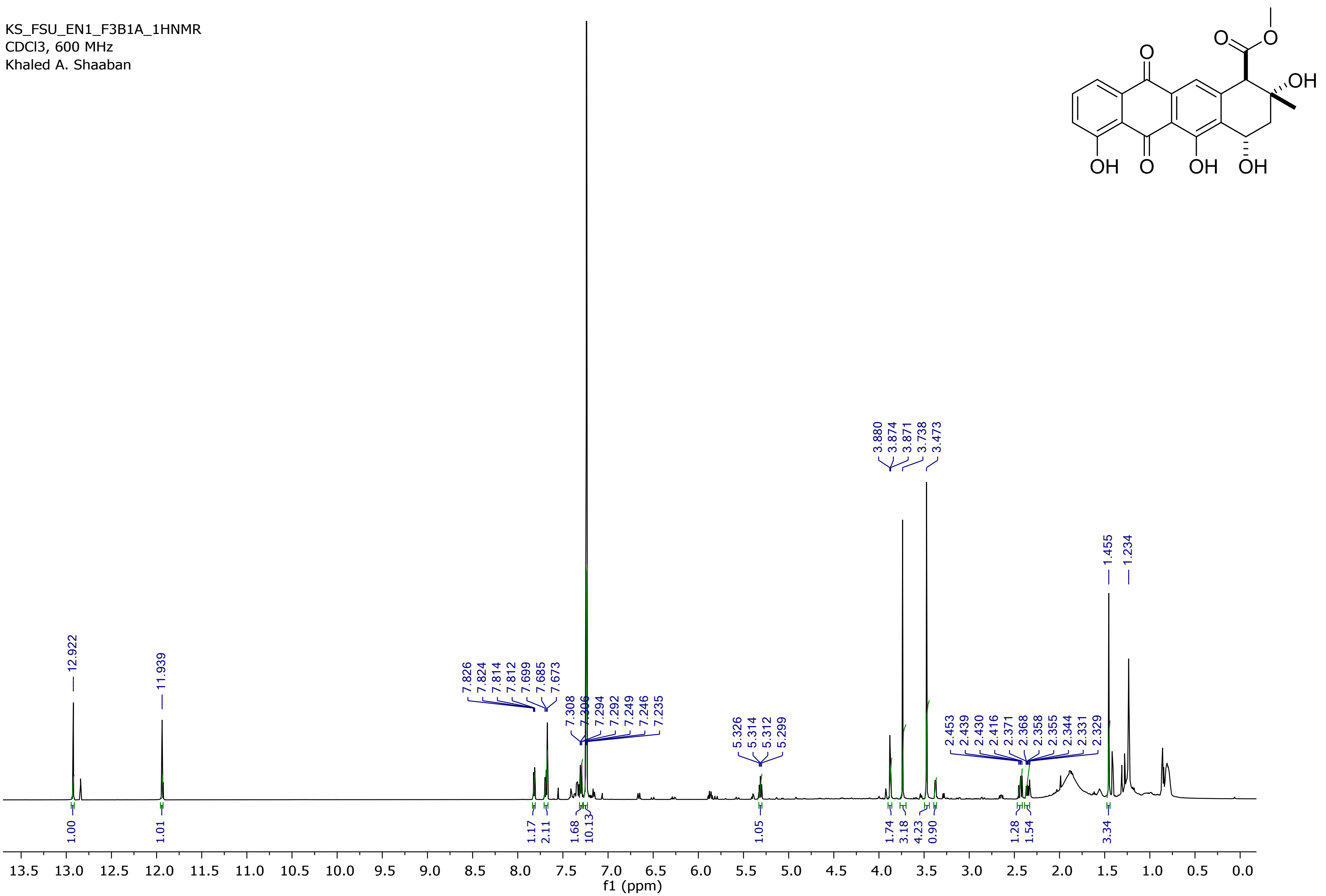

Figure S33. ^{1}H NMR spectrum ($CDCl_3$, 600 MHz) of auramycinone (9-epi-nogalamycinone; **2**).

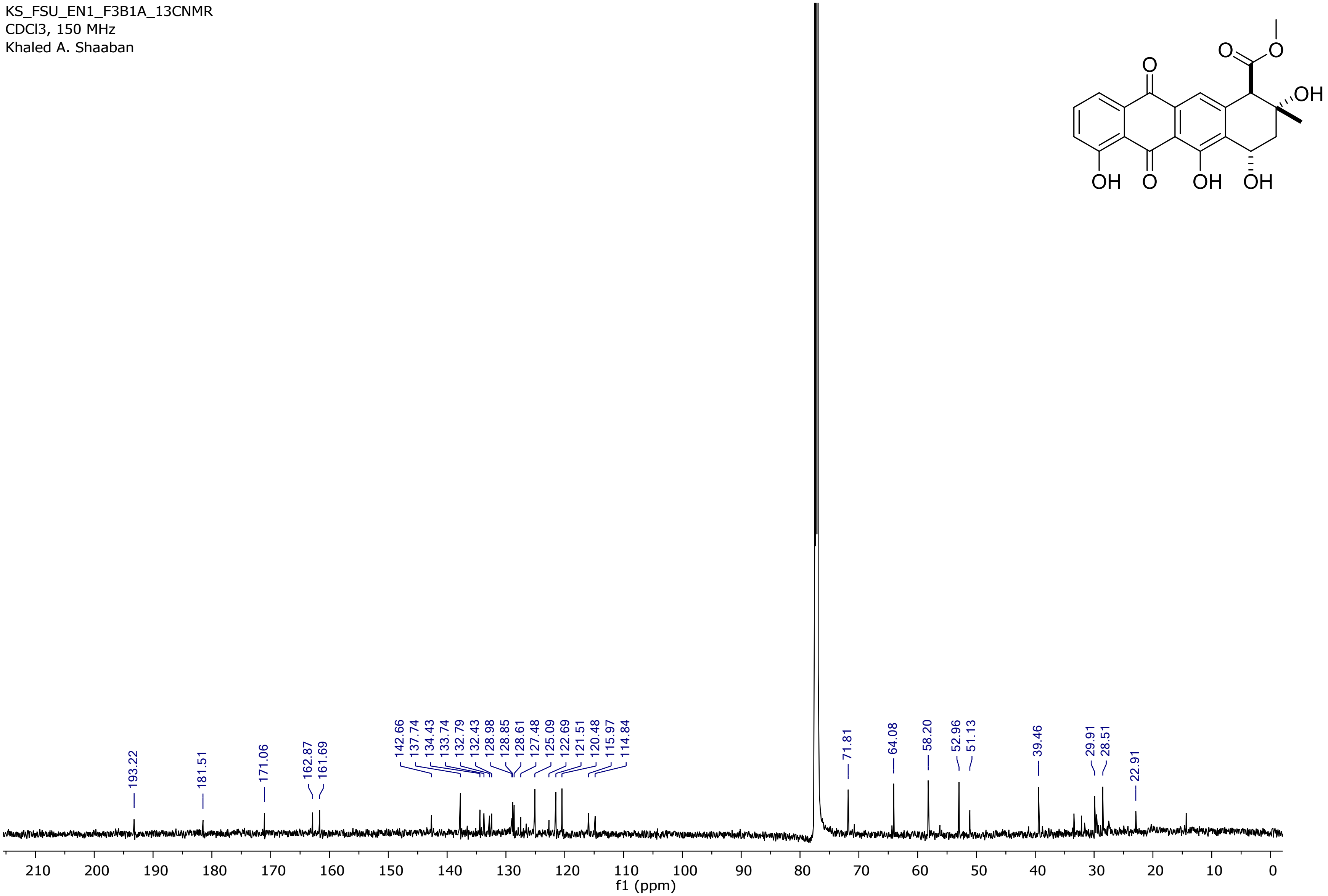

Figure S34. ^{13}C NMR spectrum ($CDCl_3$, 150 MHz) of auramycinone (9-epi-nogalamycinone; **2**).

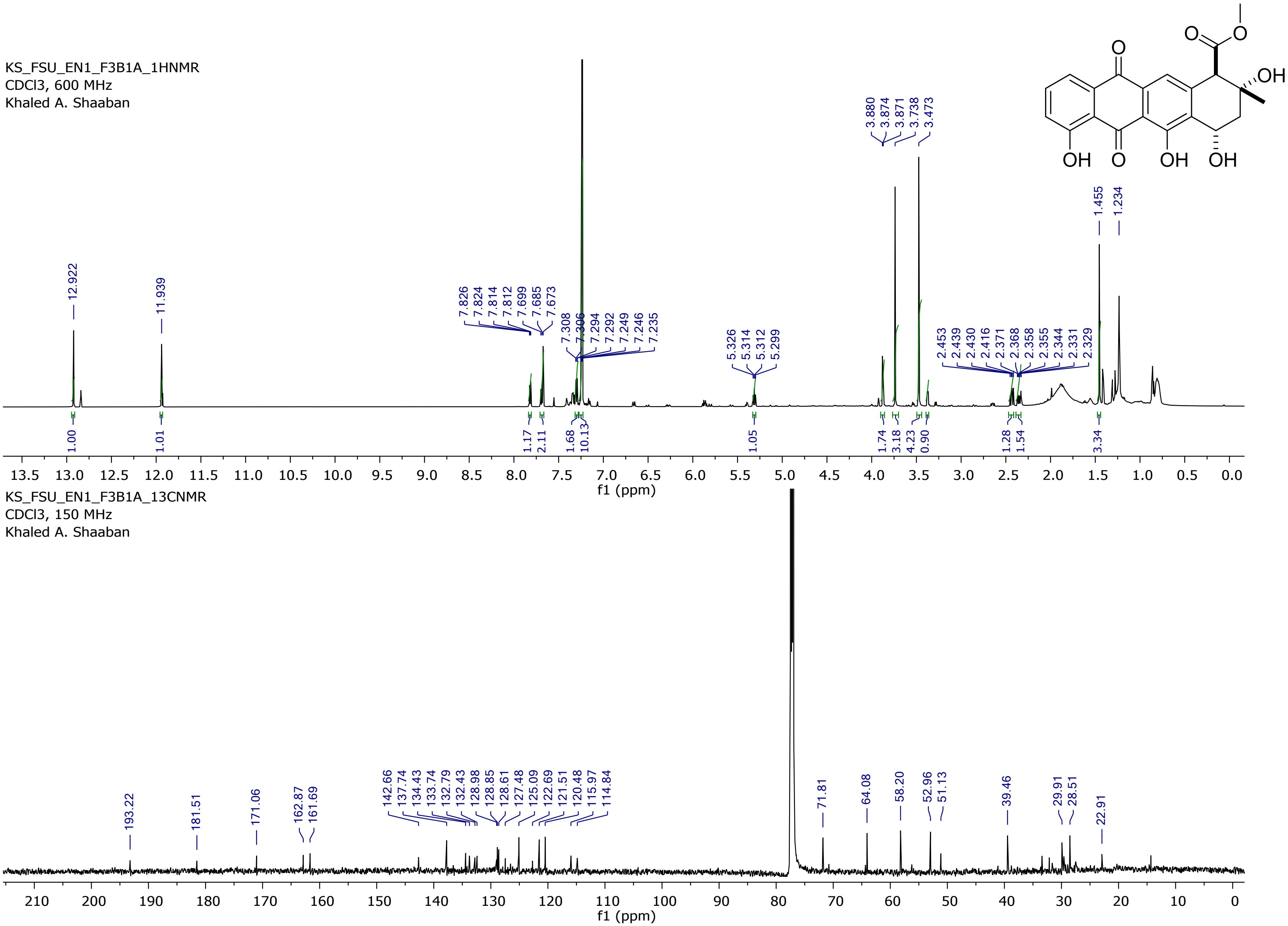

Figure S35. ^{1}H ($CDCl_3$, 600 MHz) and ^{13}C ($CDCl_3$, 150 MHz) NMR spectra of auramycinone (9-epi-nogalamycinone; **2**).

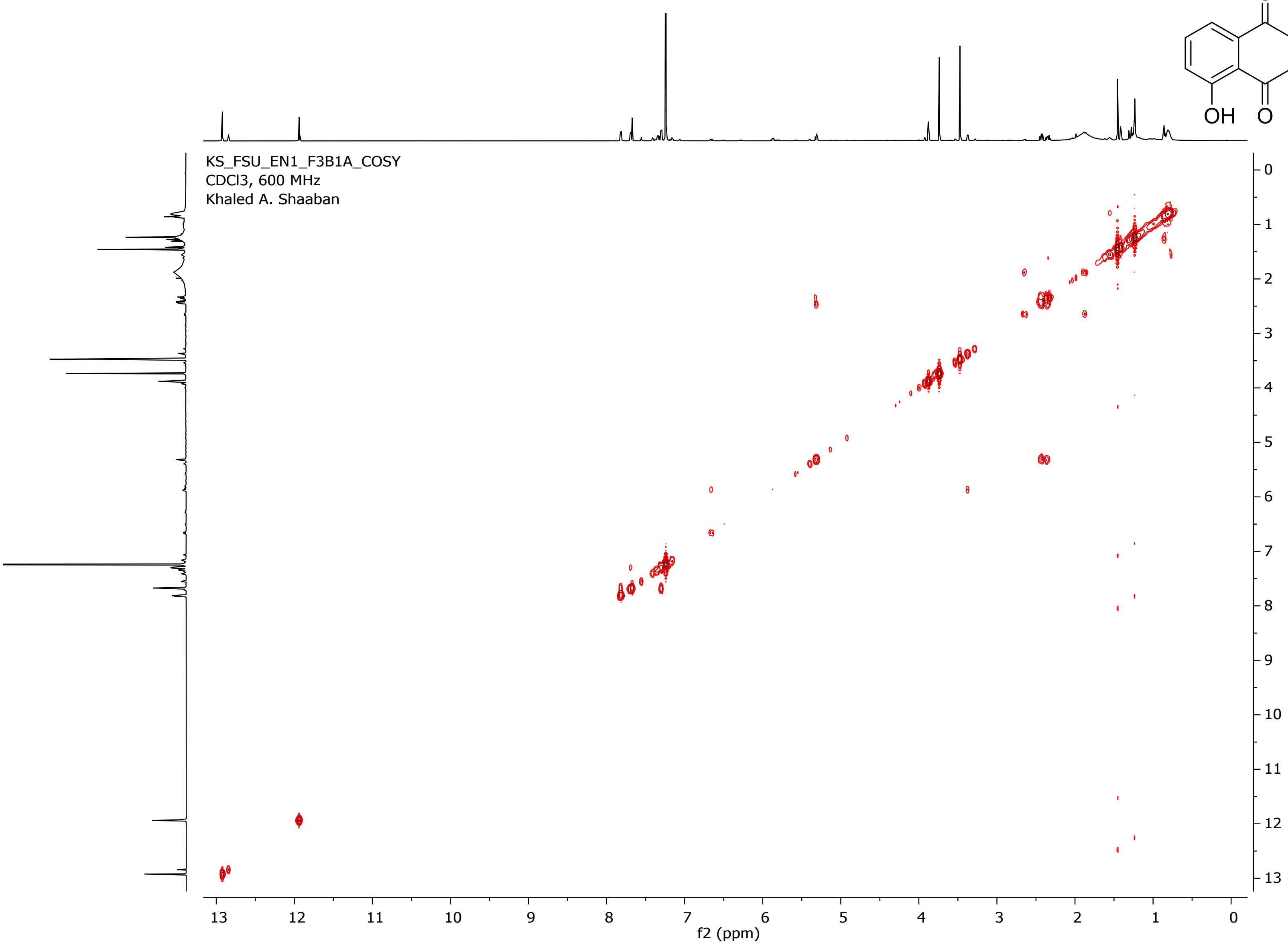

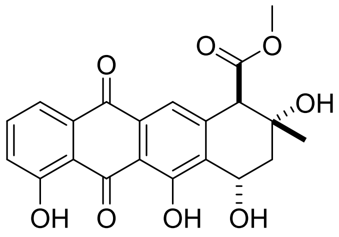

Figure S36. $^1H,^1H$-COSY spectrum ($CDCl_3$, 600 MHz) of auramycinone (9-epi-nogalamycinone; **2**).

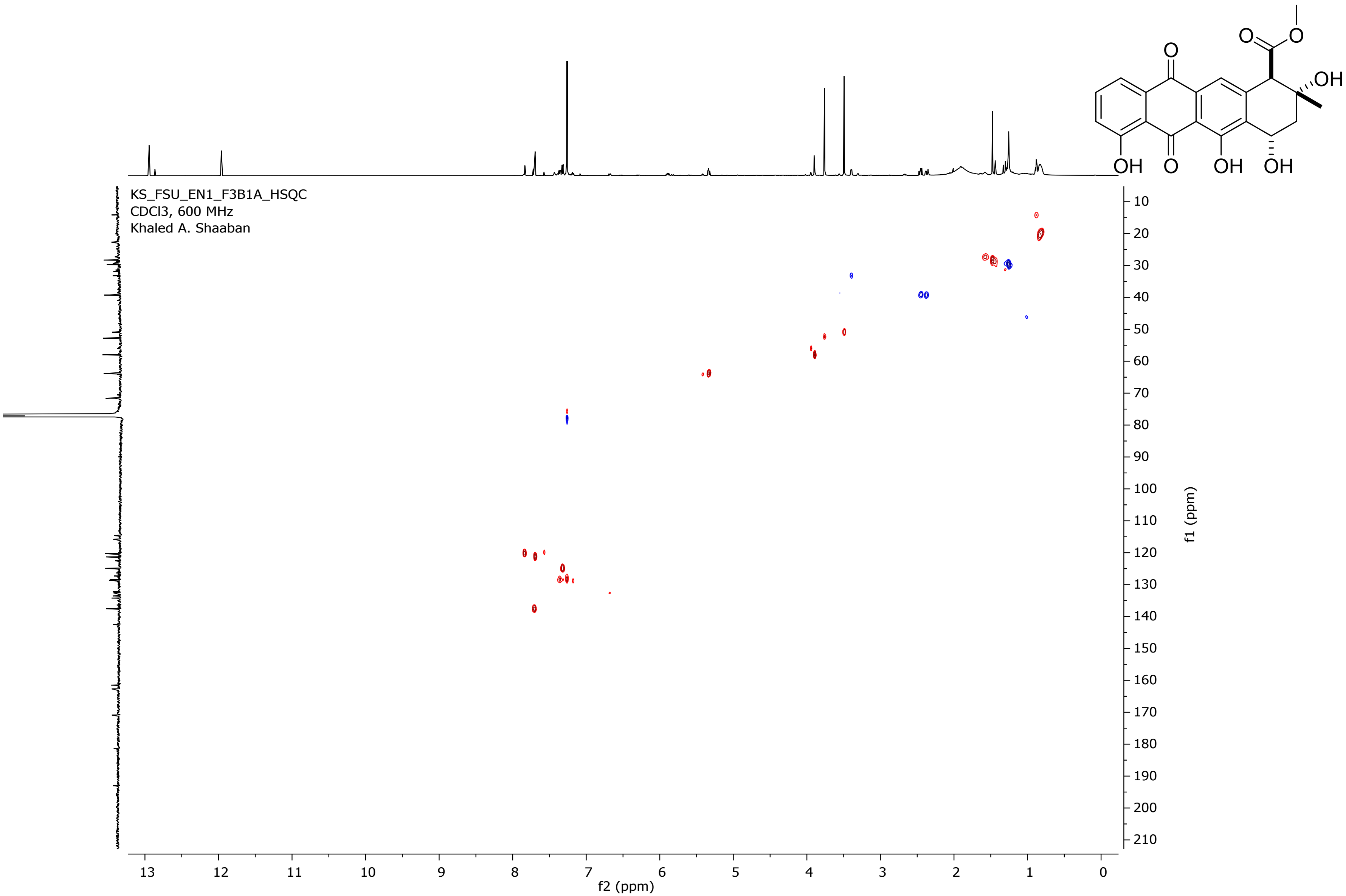

Figure S37. HSQC spectrum ($CDCl_3$, 600 MHz) of auramycinone (9-epi-nogalamycinone; **2**).

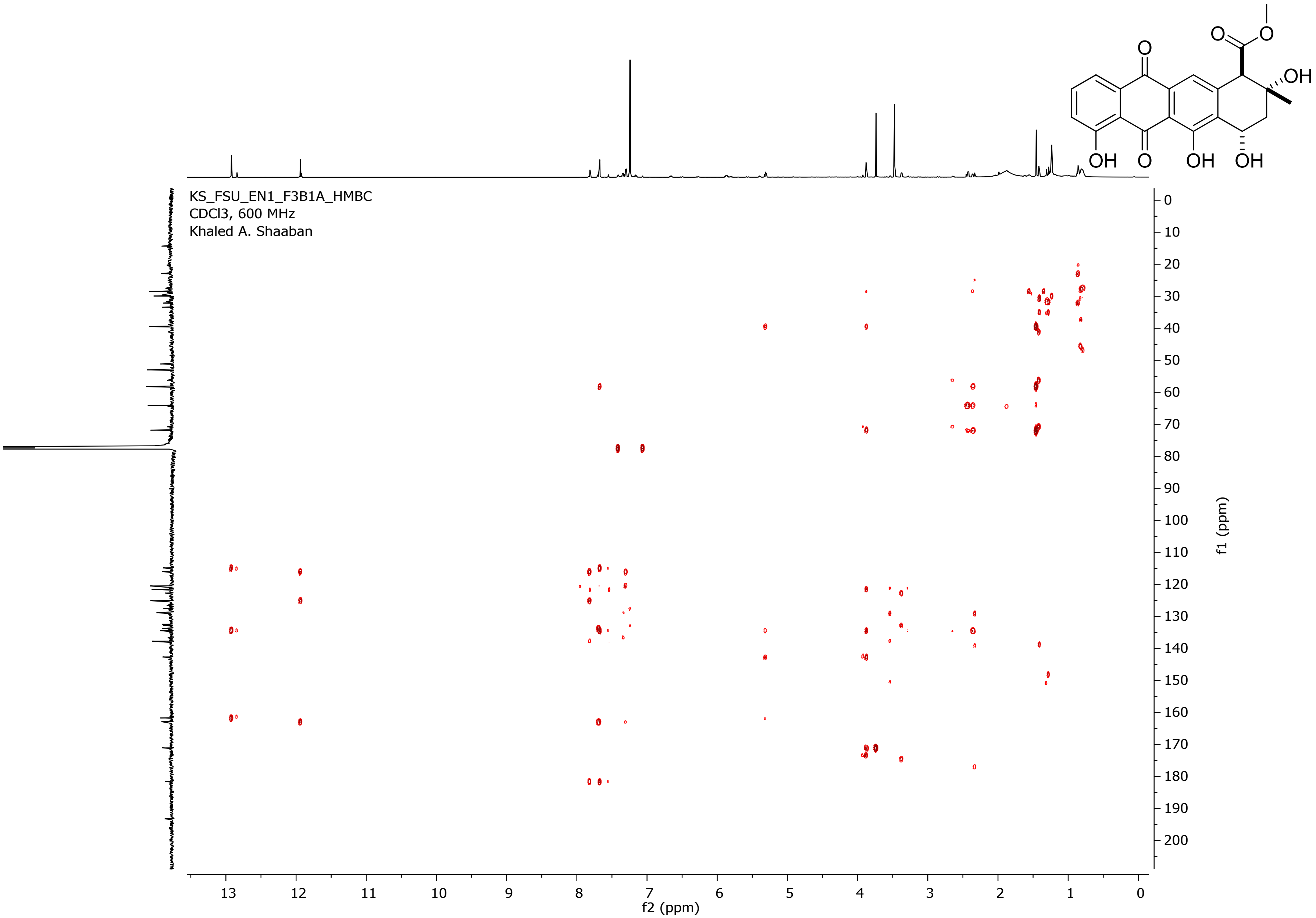

Figure S38. HMBC spectrum ($CDCl_3$, 600 MHz) of auramycinone (9-epi-nogalamycinone; **2**).

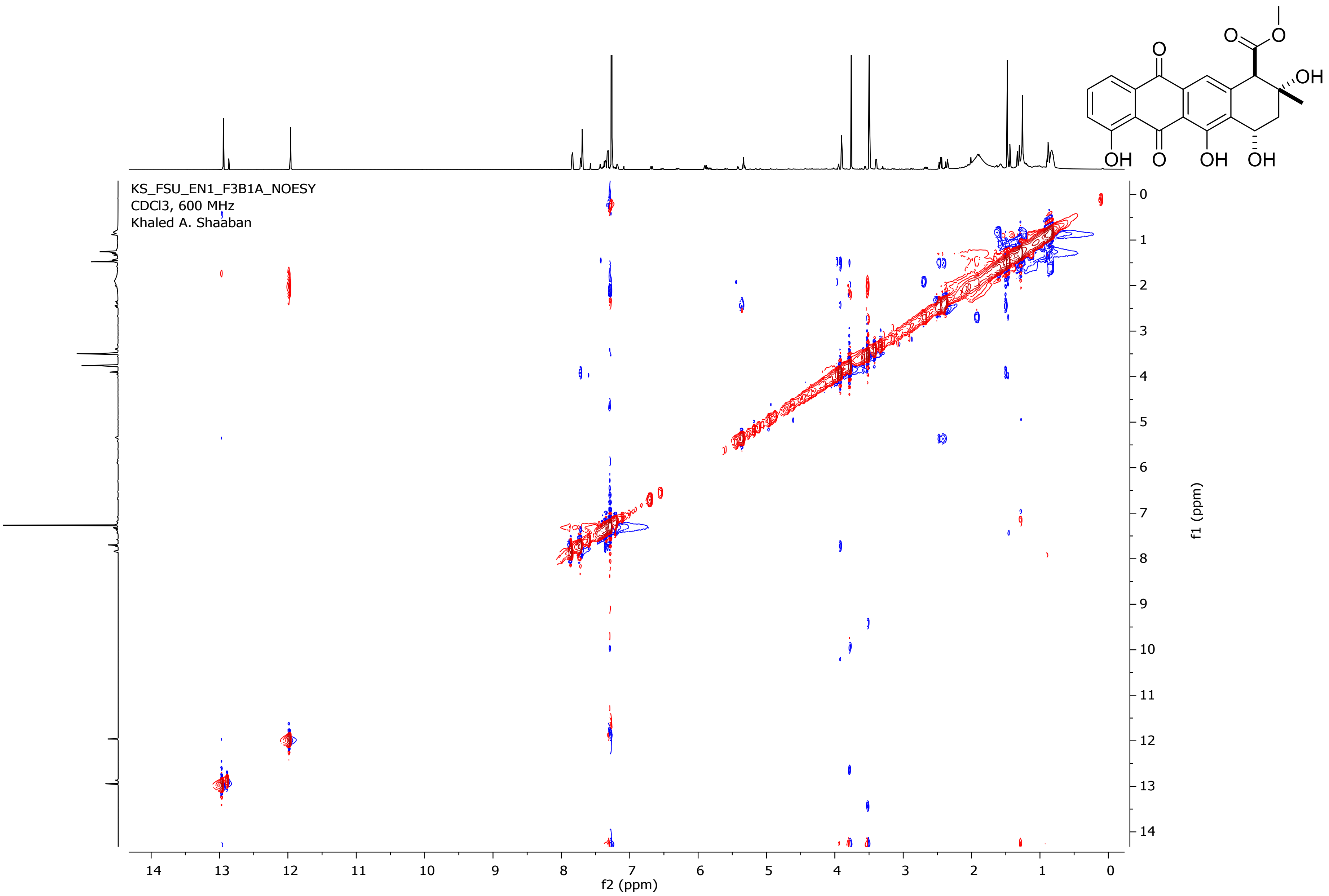

Figure S39. NOESY spectrum ($CDCl_3$, 600 MHz) of auramycinone (9-epi-Nogalamycinone; **2**).

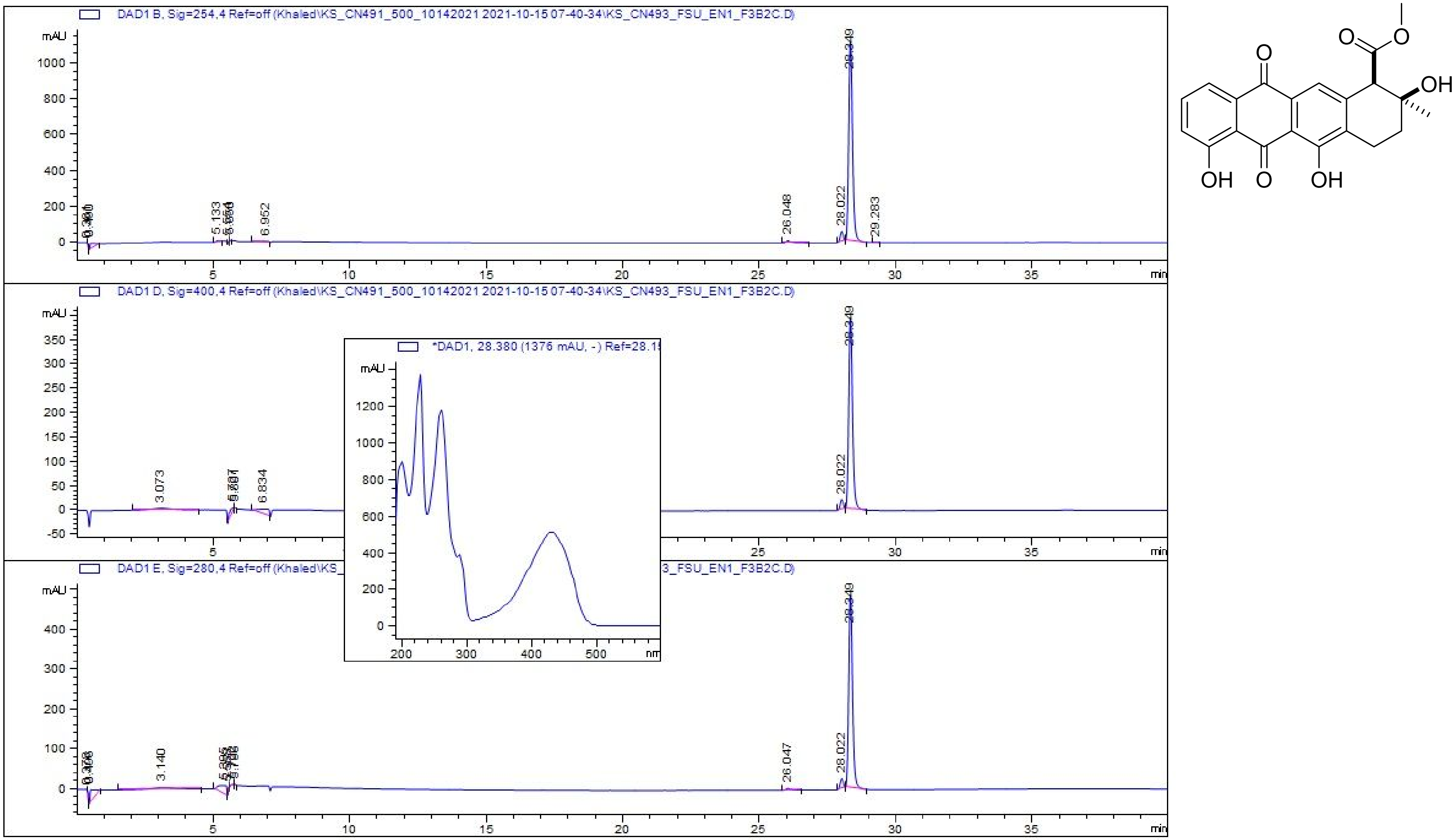

Figure S40: HPLC-UV/vis analysis of 7-deoxy-nogalamycinone (**3**). HPLC-conditions: solvent A: H2O/0.1% FA; solvent B: CH3CN; flow rate: 0.5 mL min-1; 0-30 min, 5-100% B; 30-35 min, 100% B; 35-36 min, 100-5% B; 36-40 min, 5% B; Phenomenex NX-C18 column (250 × 4.6 mm, 5 µm); 254 nm, 280 nm, 400 nm. UV-vis inset of full wavelength scan (190-600 nm).

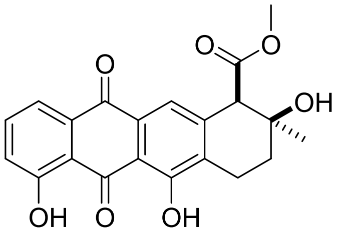

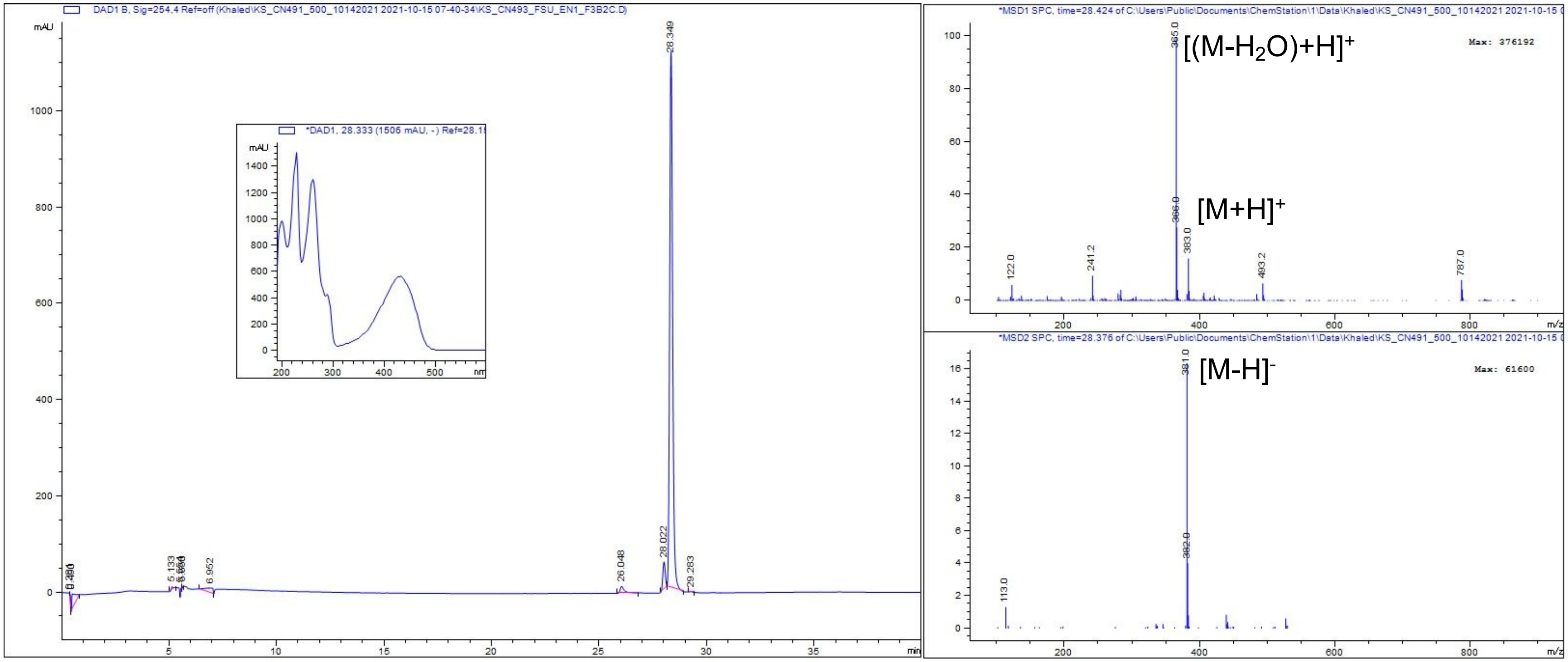

Figure S41: HPLC-MS analysis of 7-deoxy-nogalamycinone (**3**). HPLC-conditions: solvent A: H2O/0.1% FA; solvent B: CH3CN; flow rate: 0.5 mL min-1; 0-30 min, 5-100% B; 30-35 min, 100% B; 35-36 min, 100-5% B; 36-40 min, 5% B; Phenomenex NX-C18 column (250 × 4.6 mm, 5 μm); 254 nm. UV-vis inset of full wavelength scan (190-600 nm).

**Sample Name**	FSU_EN1_F3B2C	**Position**	P2-A5	**Instrument Name**	Instrument 1
**User Name**		**Inj Vol**	4	**InjPosition**	
**Sample Type**	Sample	**IRM Calibration Status**	Success	**Data Filename**	FSU_EN1_F3B2C.d
**ACQ Method**	Zheng_AQC ACC short_Neg.m	**Comment**		**Acquired Time**	9/22/2021 12:49:54 AM (UTC-04:00)

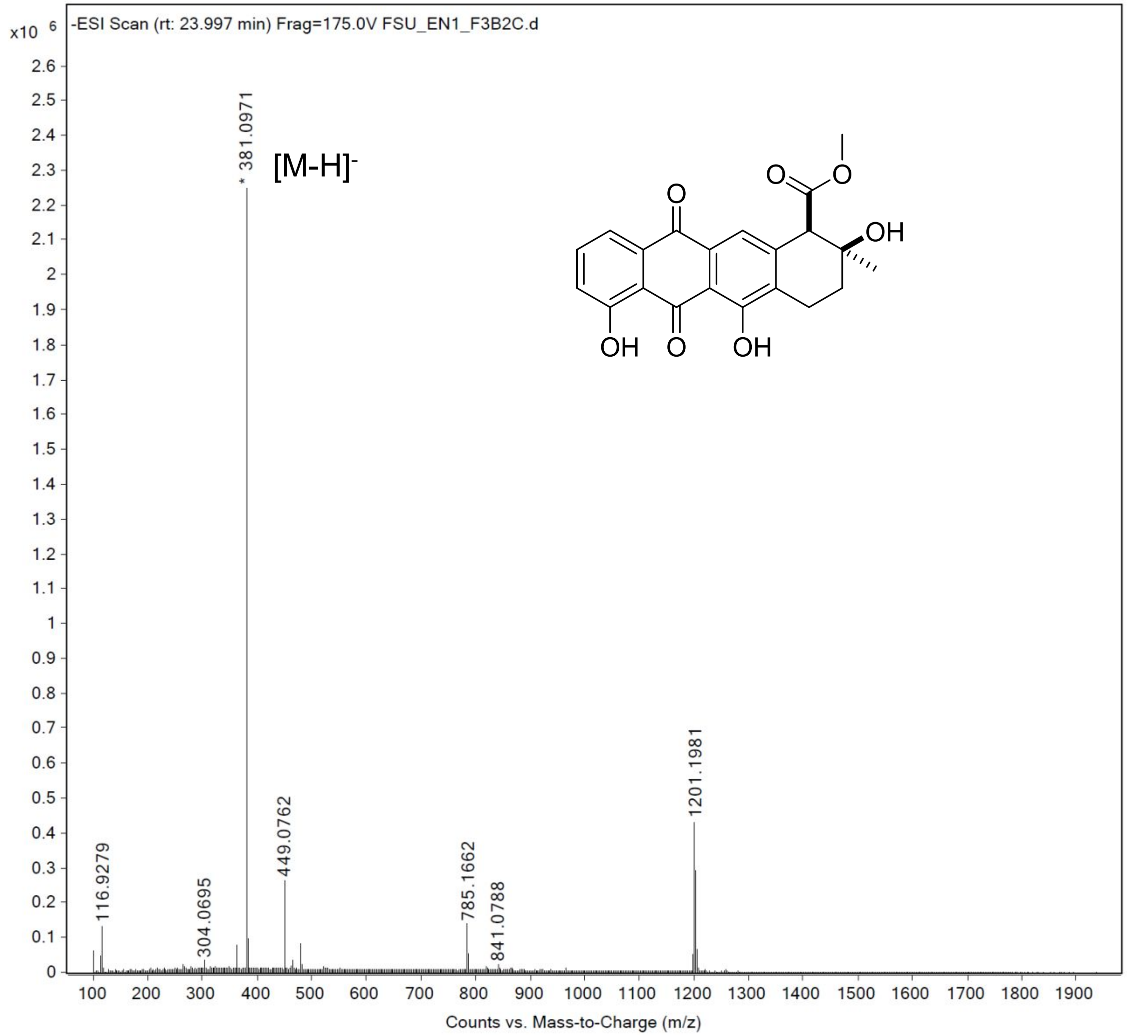

Figure S42. (–)-HRESI-MS spectrum of 7-deoxy-nogalamycinone (**3**).

**Sample Name**	FSU_EN1_F3B2C	**Position**	P2-A5	**Instrument Name**	Instrument 1
**User Name**		**Inj Vol**	4	**InjPosition**	
**Sample Type**	Sample	**IRM Calibration Status**	Success	**Data Filename**	FSU_EN1_F3B2C.d
**ACQ Method**	Zheng_AQC ACC short_Pos.m	**Comment**		**Acquired Time**	9/22/2021 10:13:37 PM (UTC-04:00)

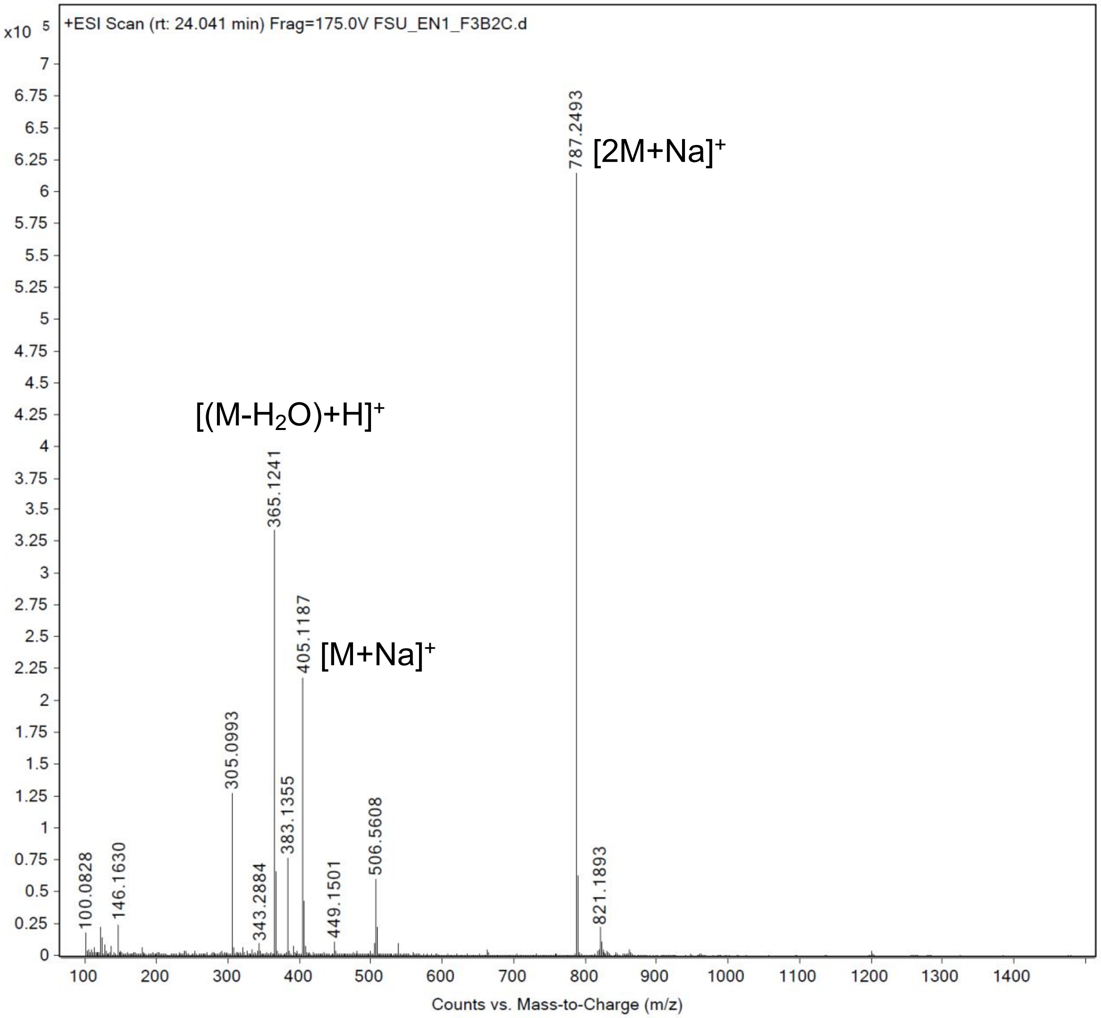

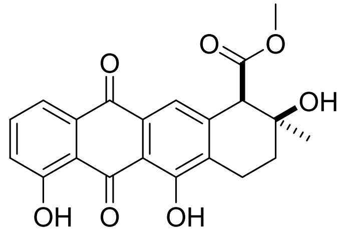

Figure S43. (+)-HRESI-MS spectrum of 7-deoxy-nogalamycinone (**3**).

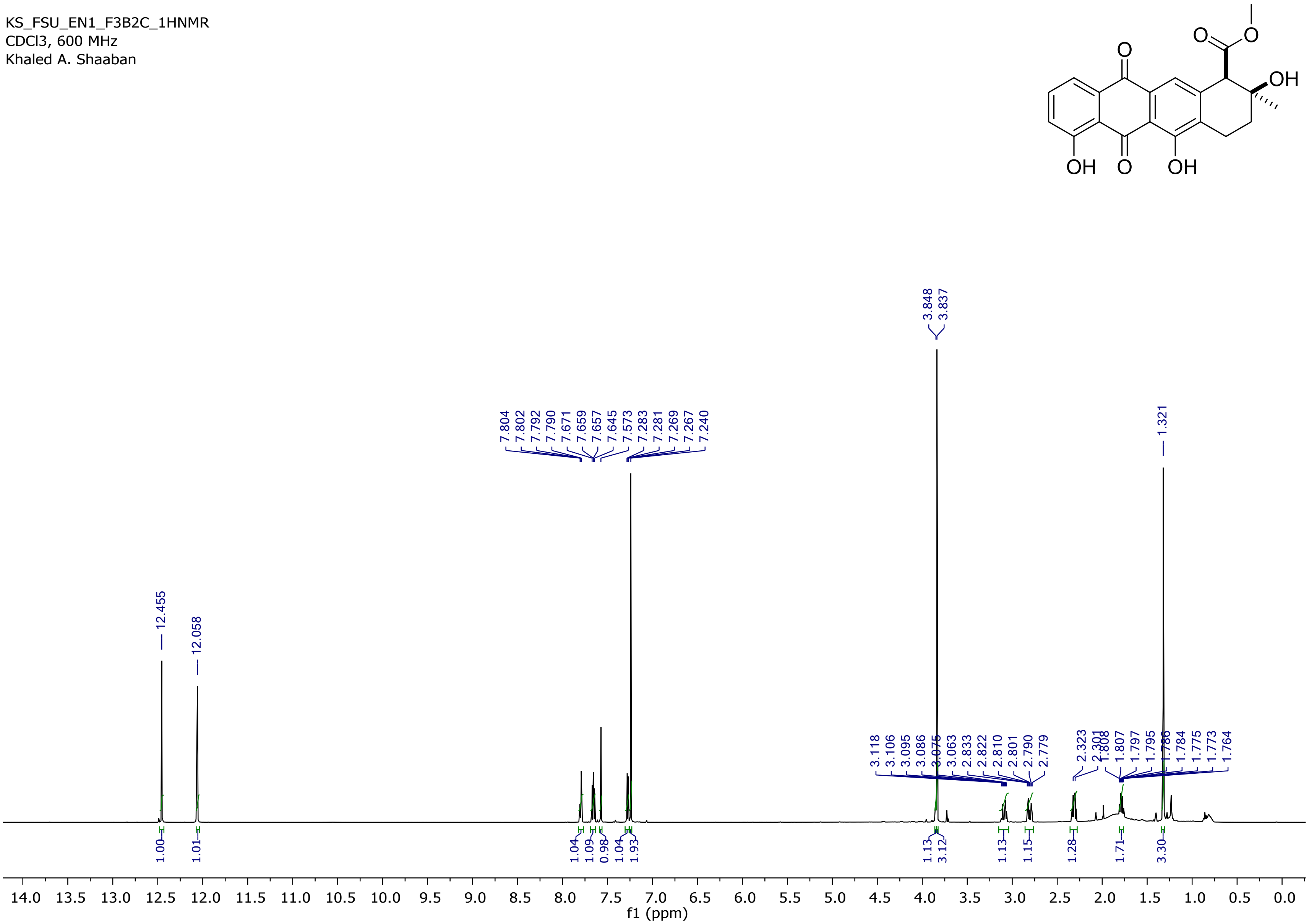

Figure S44. ^1H NMR spectrum ($CDCl_3$, 600 MHz) of 7-deoxy-nogalamycinone (**3**).

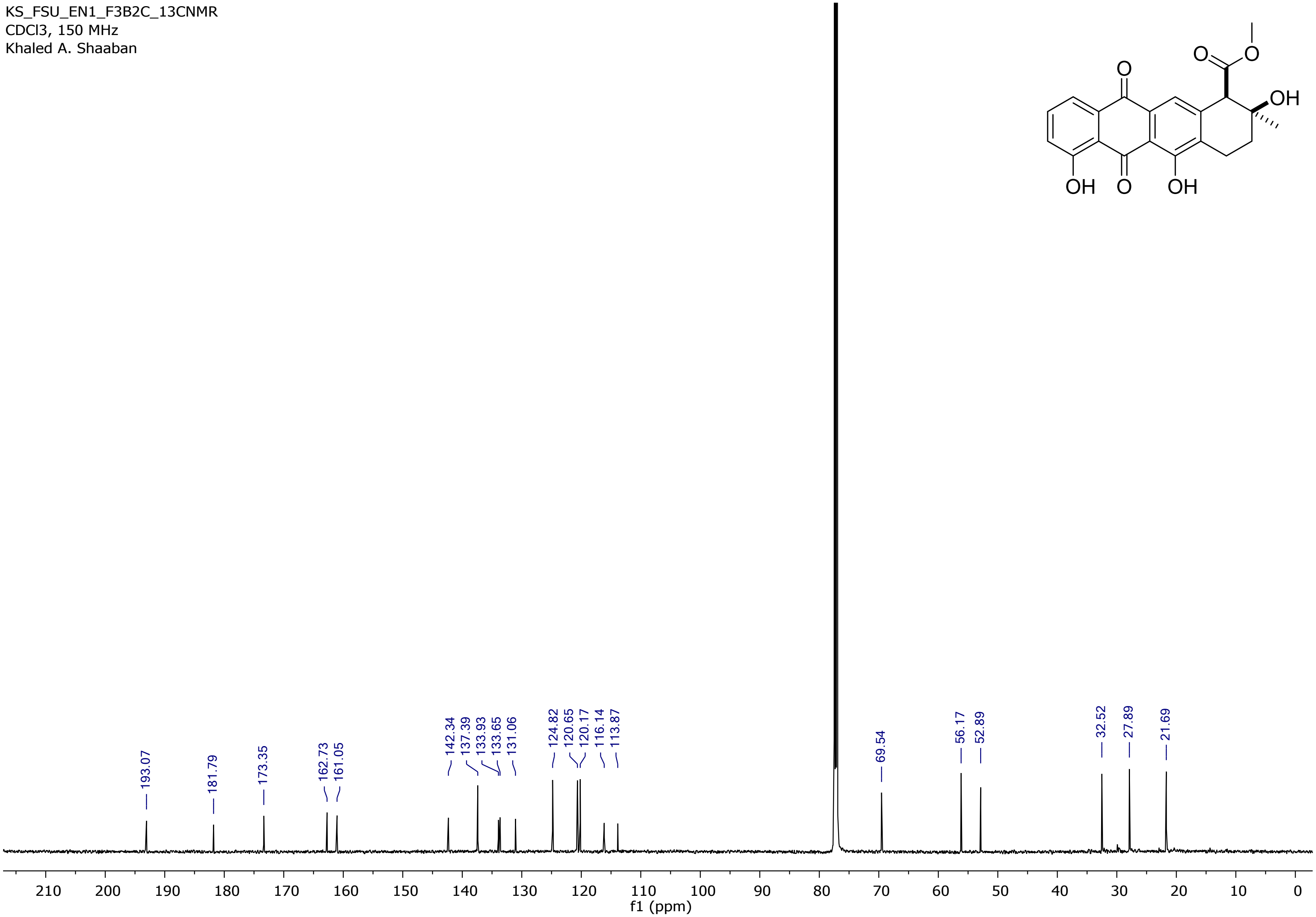

Figure S45. ^{13}C NMR spectrum ($CDCl_3$, 150 MHz) of 7-deoxy-nogalamycinone (**3**).

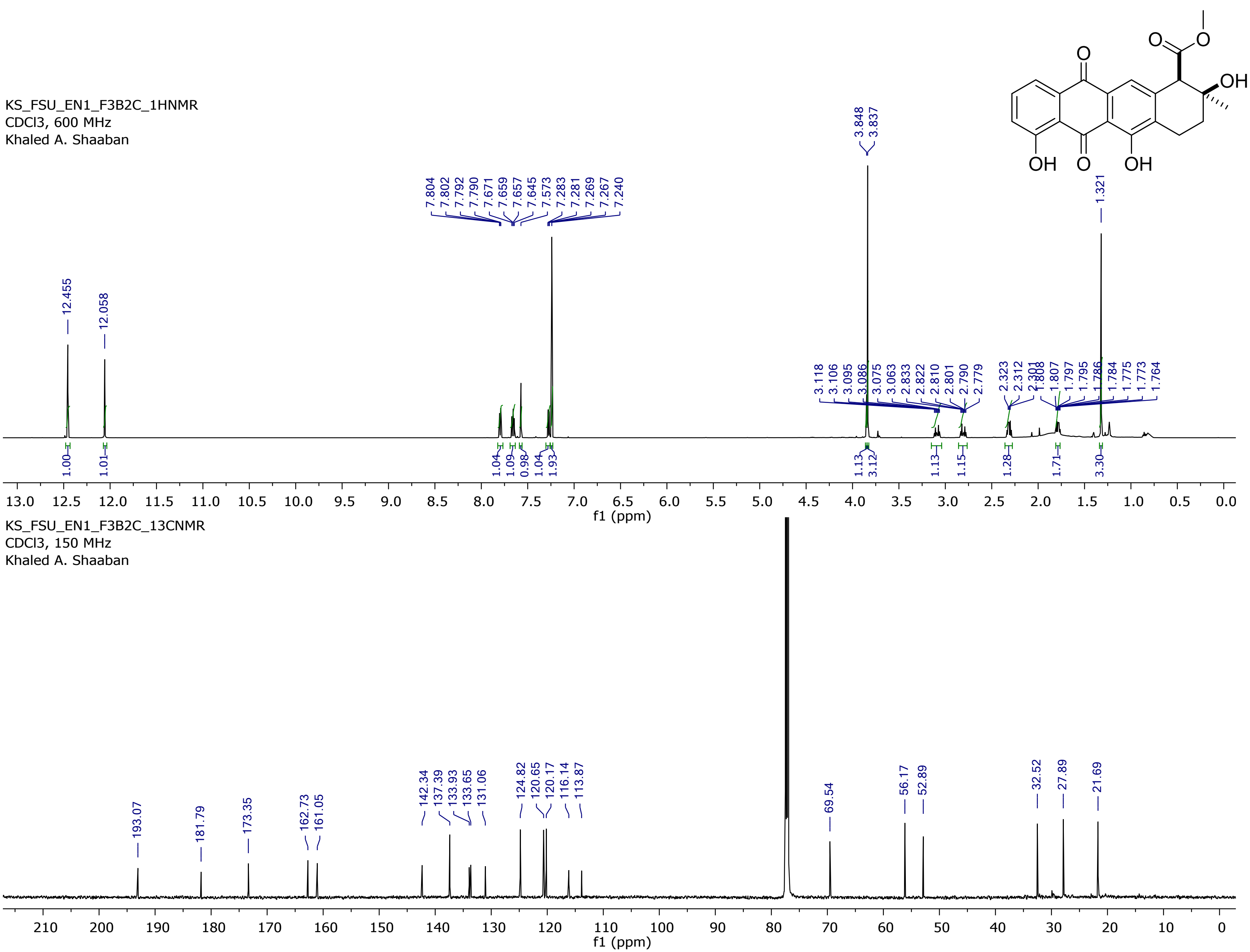

Figure S46. ^{1}H (CDCl3, 600 MHz) and ^{13}C ($CDCl_3$, 150 MHz) NMR spectra of 7-deoxy-nogalamycinone (**3**).

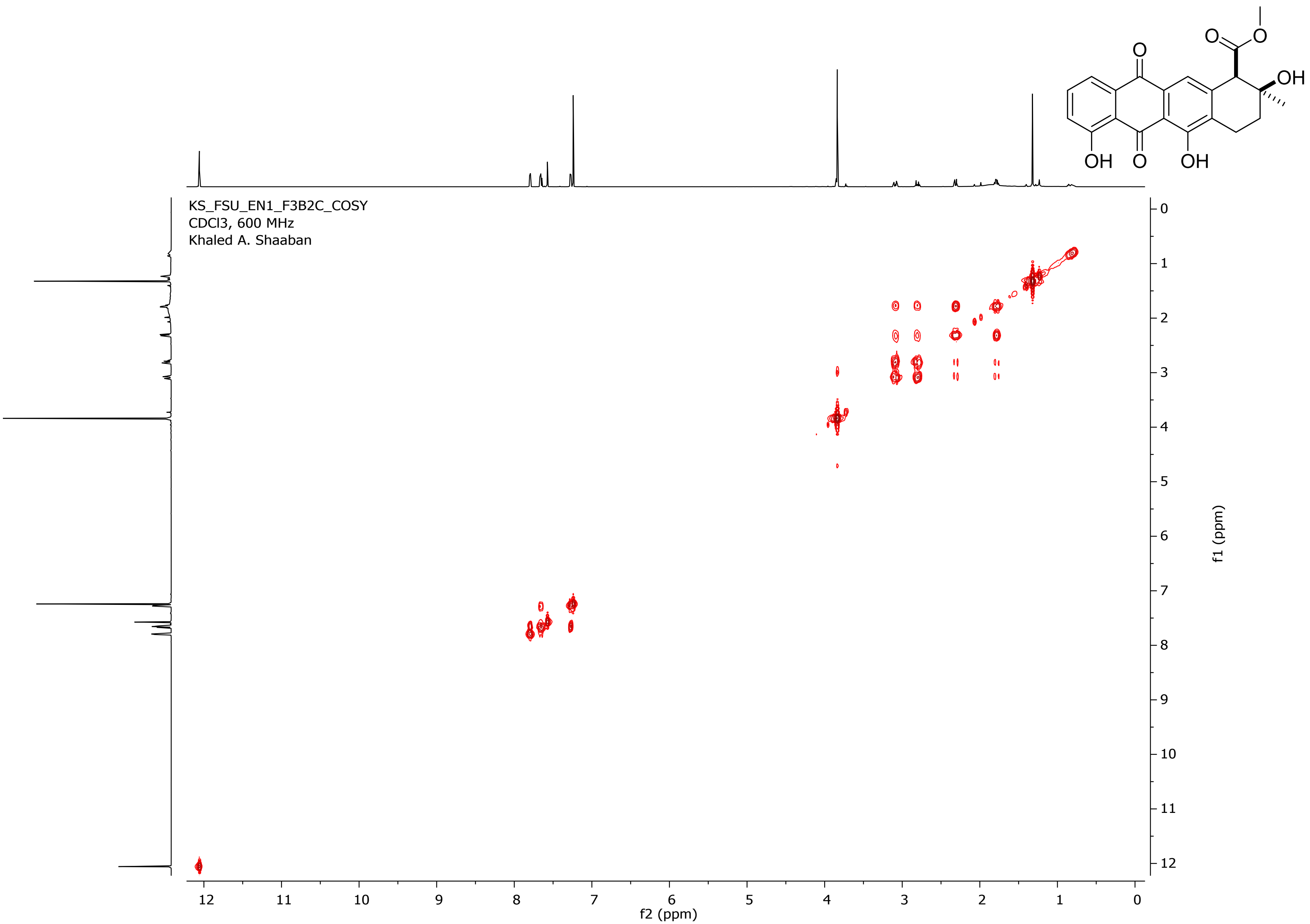

Figure S47. $^1H,^1H$-COSY spectrum ($CDCl_3$, 600 MHz) of 7-deoxy-nogalamycinone (**3**).

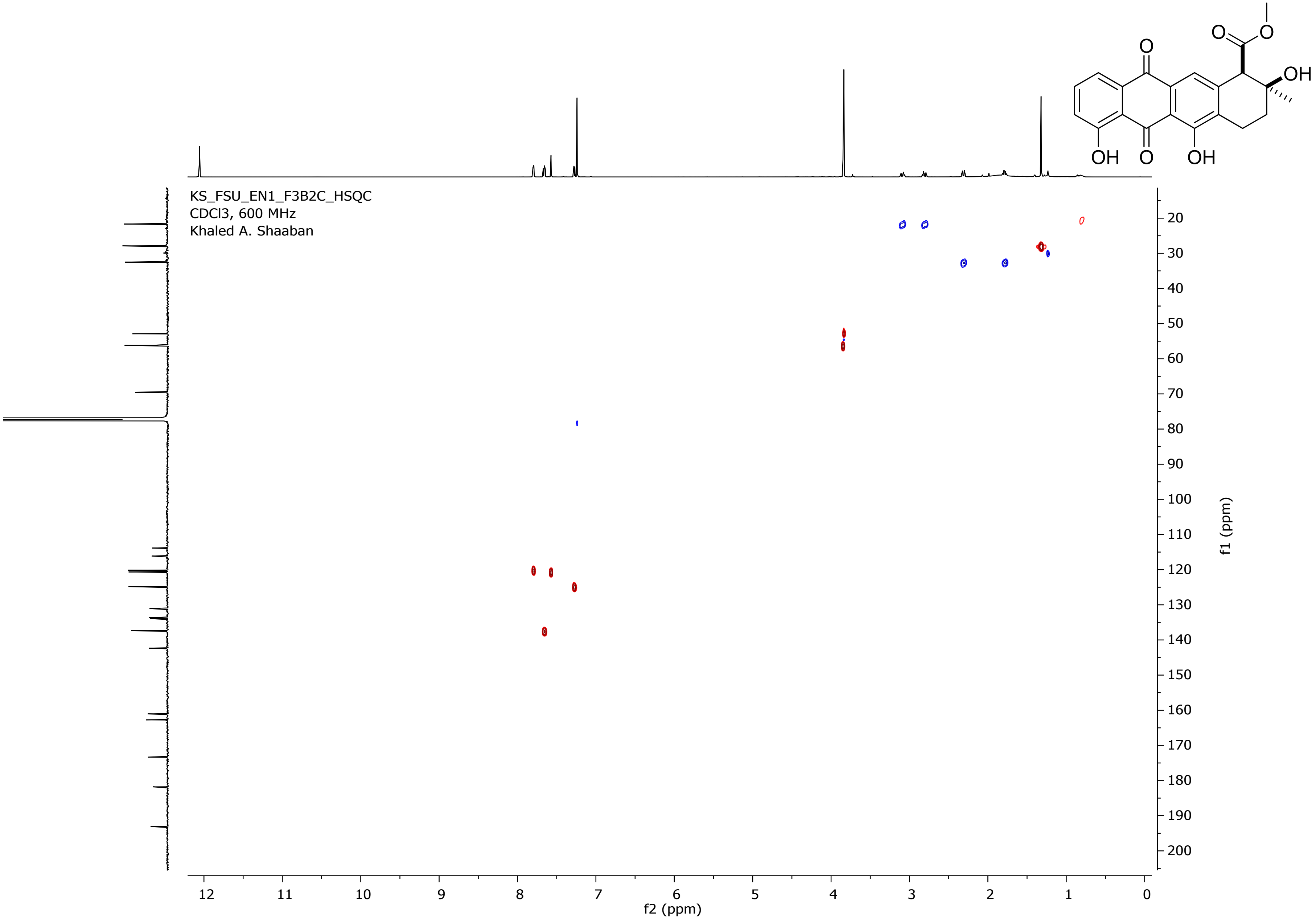

Figure S48. HSQC spectrum ($CDCl_3$, 600 MHz) of 7-deoxy-nogalamycinone (**3**).

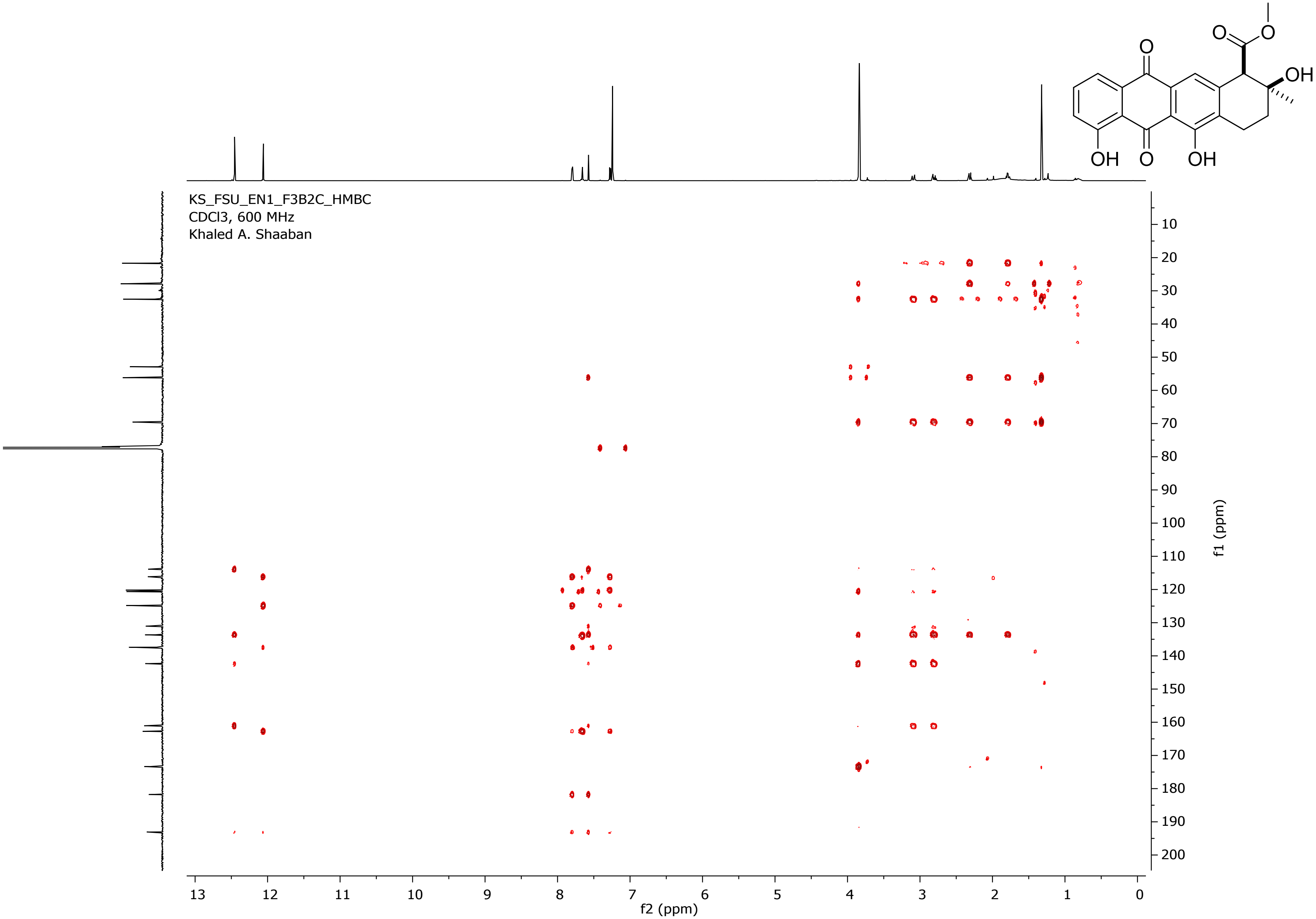

Figure S49. HMBC spectrum ($CDCl_3$, 600 MHz) of 7-deoxy-nogalamycinone (**3**).

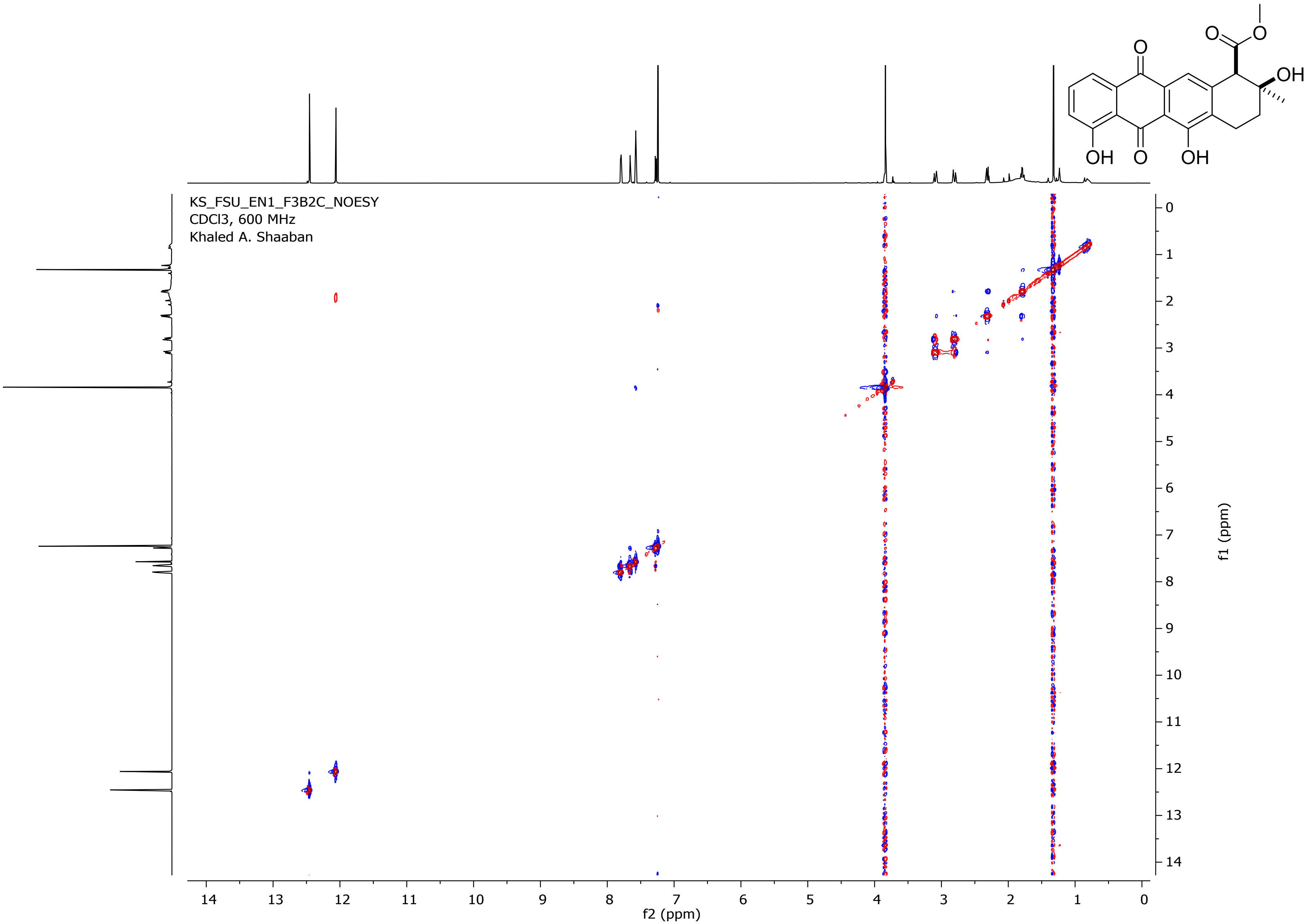

Figure S50. NOESY spectrum ($CDCl_3$, 600 MHz) of 7-deoxy-nogalamycinone (**3**).

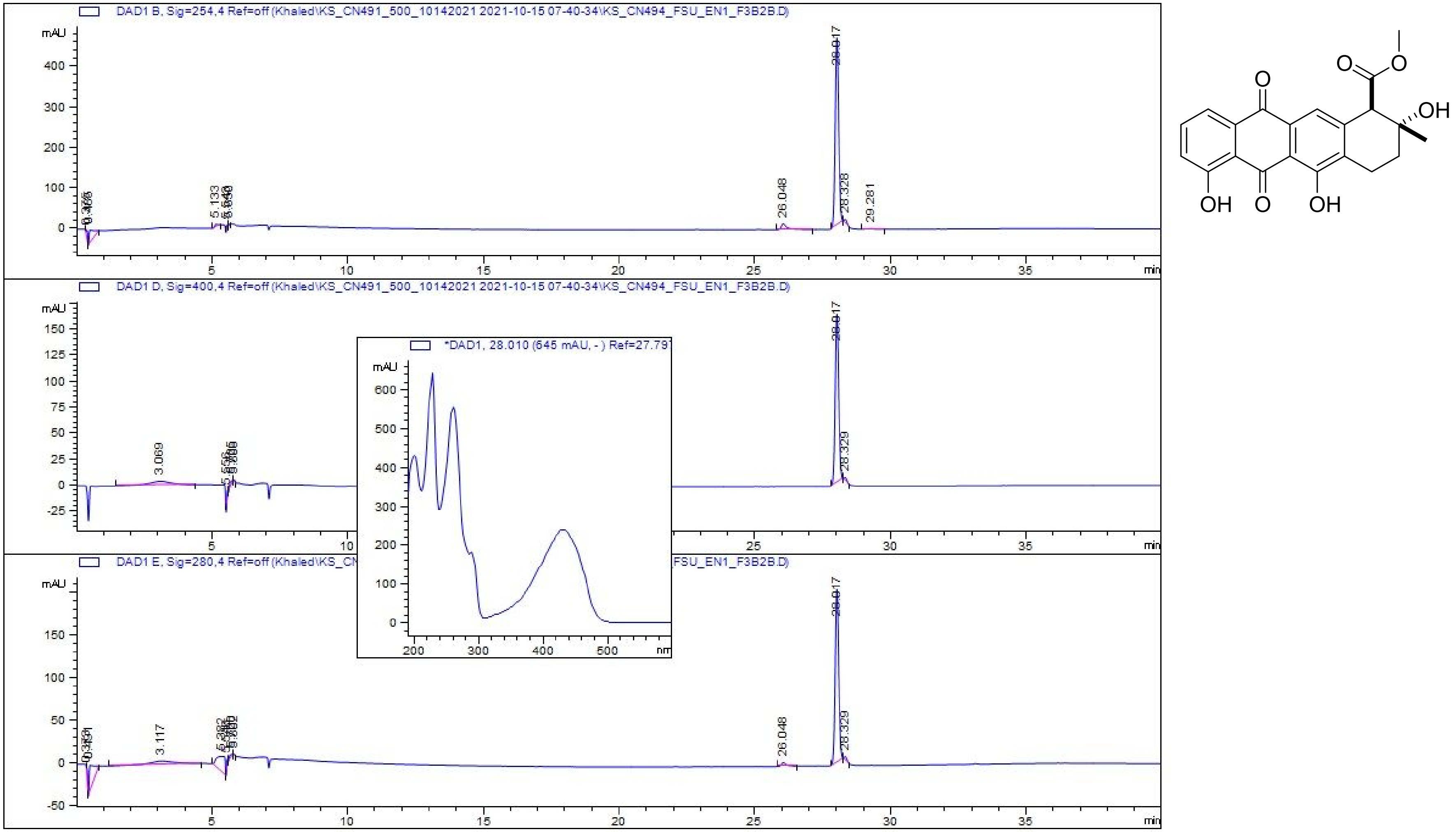

Figure S51: HPLC-UV/vis analysis of 7-deoxyauramycinone (9-epi-7-deoxy-nogalamycinone; **4**). HPLC-conditions: solvent A: H2O/0.1% FA; solvent B: CH3CN; flow rate: 0.5 mL min-1; 0-30 min, 5-100% B; 30-35 min, 100% B; 35-36 min, 100-5% B; 36-40 min, 5% B; Phenomenex NX-C18 column (250 × 4.6 mm, 5 µm); 254 nm, 280 nm, 400 nm. UV-vis inset of full wavelength scan (190-600 nm).

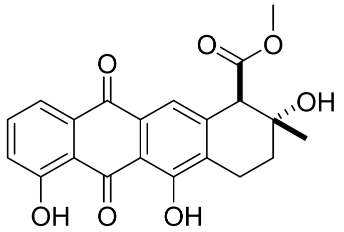

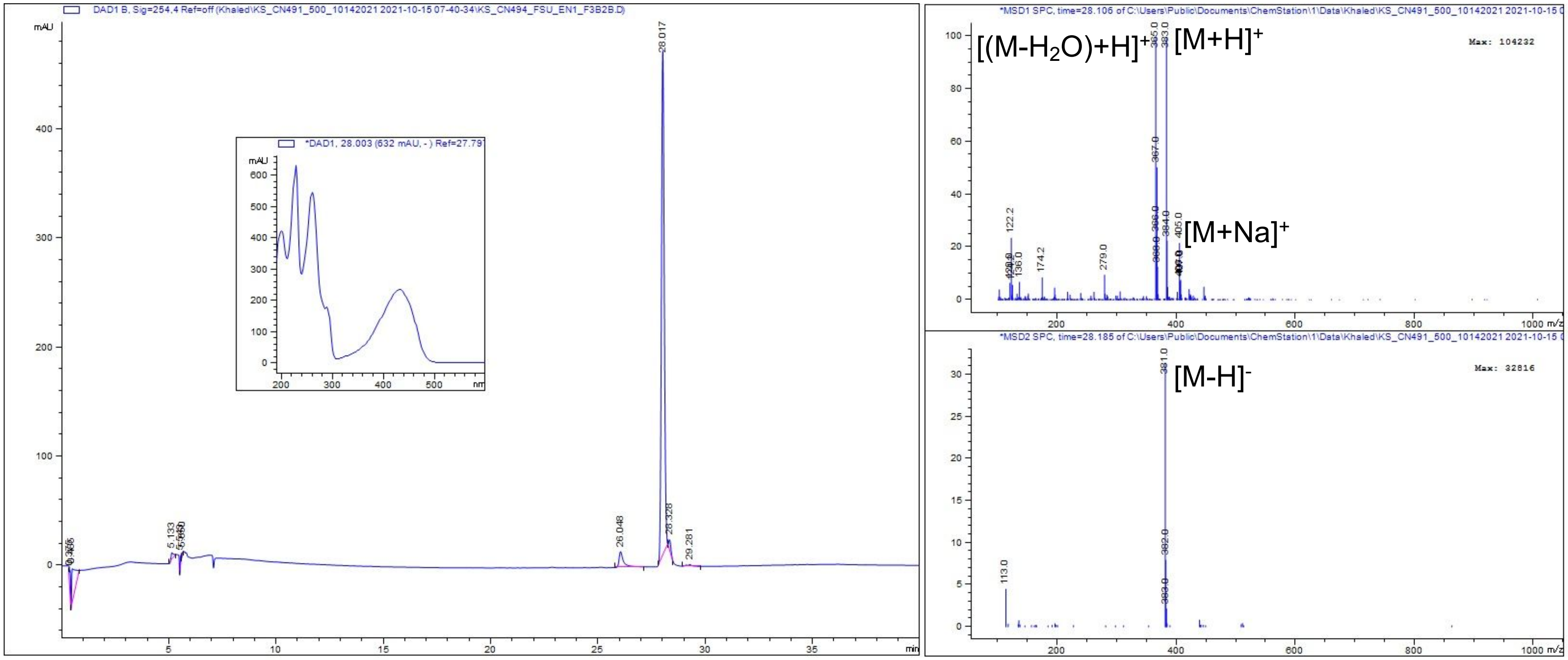

Figure S52: HPLC-MS analysis of 7-deoxyauramycinone (9-epi-7-deoxy-nogalamycinone; **4**). HPLC-conditions: solvent A: H2O/0.1% FA; solvent B: CH3CN; flow rate: 0.5 mL min-1; 0-30 min, 5-100% B; 30-35 min, 100% B; 35-36 min, 100-5% B; 36-40 min, 5% B; Phenomenex NX-C18 column (250 × 4.6 mm, 5 µm); 254 nm. UV-vis inset of full wavelength scan (190-600 nm).

**Sample Name**	FSU_EN1_F3B2B	**Position**	P2-A4	**Instrument Name**	Instrument 1
**User Name**		**Inj Vol**	4	**InjPosition**	
**Sample Type**	Sample	**IRM Calibration Status**	Success	**Data Filename**	FSU_EN1_F3B2B.d
**ACQ Method**	Zheng_AQC ACC short_Neg.m	**Comment**		**Acquired Time**	9/22/2021 12:20:58 AM (UTC-04:00)

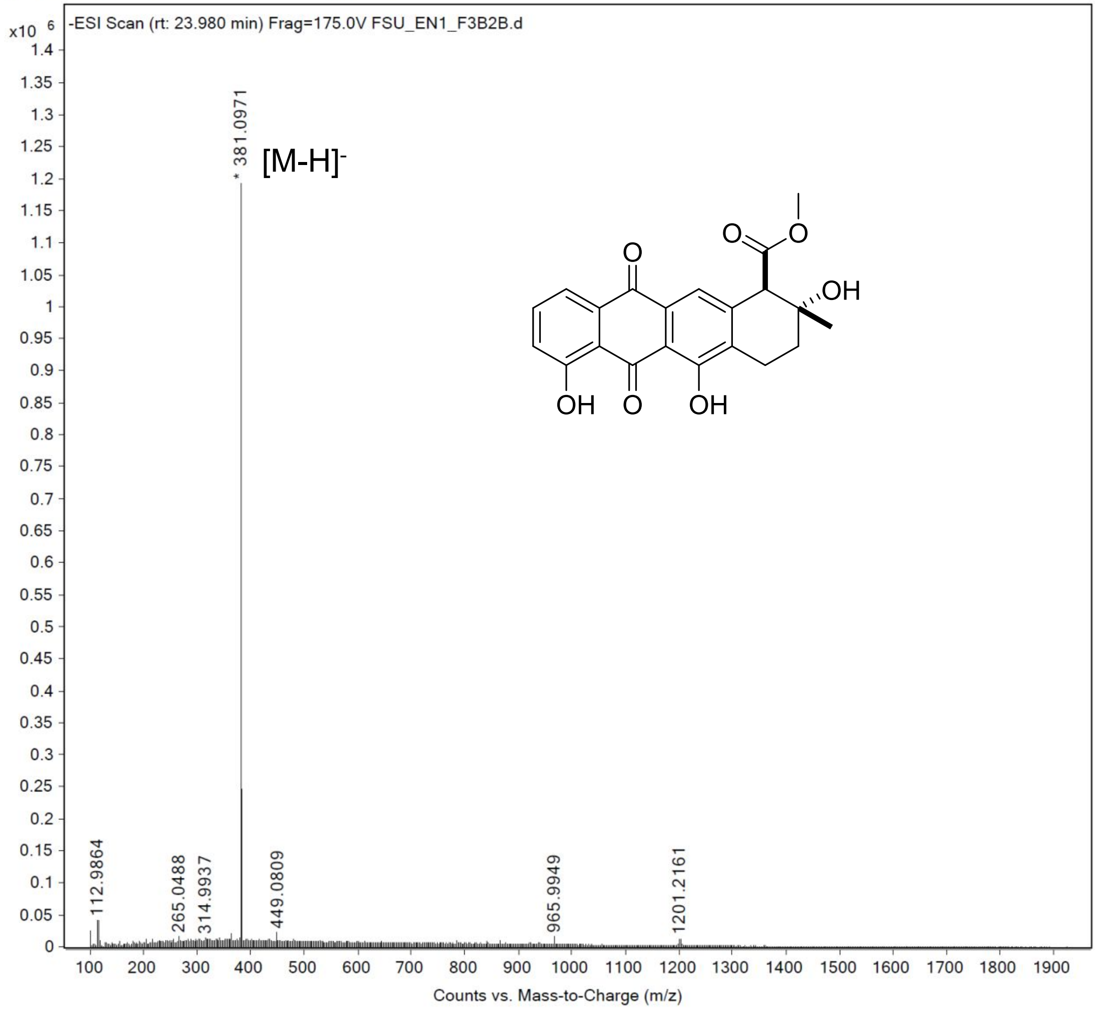

Figure S53. (–)-HRESI-MS spectrum of 7-deoxyauramycinone (9-epi-7-deoxy-nogalamycinone; **4**).

**Sample Name**	FSU_EN1_F3B2B	**Position**	P2-A4	**Instrument Name**	Instrument 1
**User Name**		**Inj Vol**	4	**InjPosition**	
**Sample Type**	Sample	**IRM Calibration Status**	Success	**Data Filename**	FSU_EN1_F3B2B.d
**ACQ Method**	Zheng_AQC ACC short_Pos.m	**Comment**		**Acquired Time**	9/22/2021 9:44:42 PM (UTC-04:00)

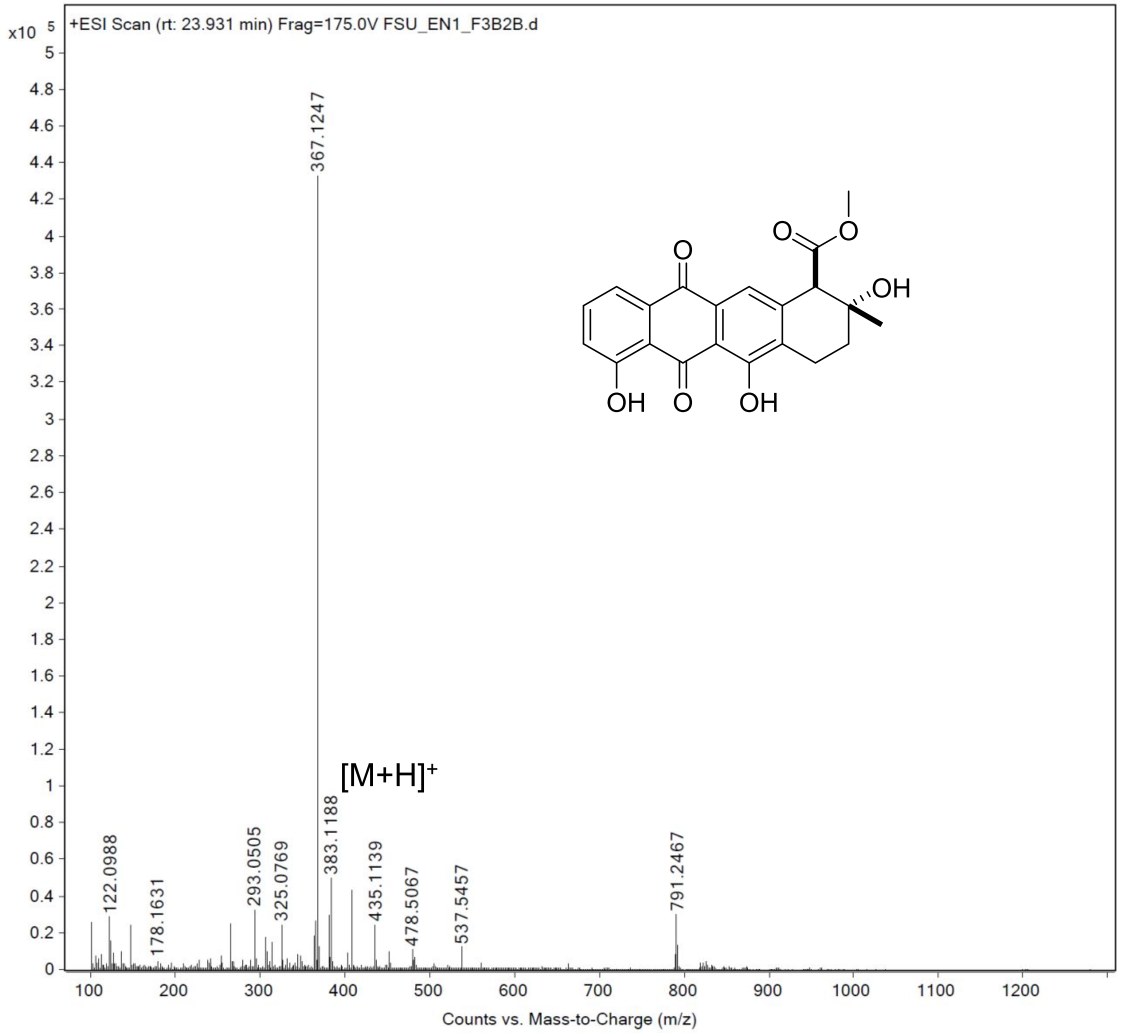

Figure S54. (+)-HRESI-MS spectrum of 7-deoxyauramycinone (9-epi-7-deoxy-nogalamycinone; **4**).

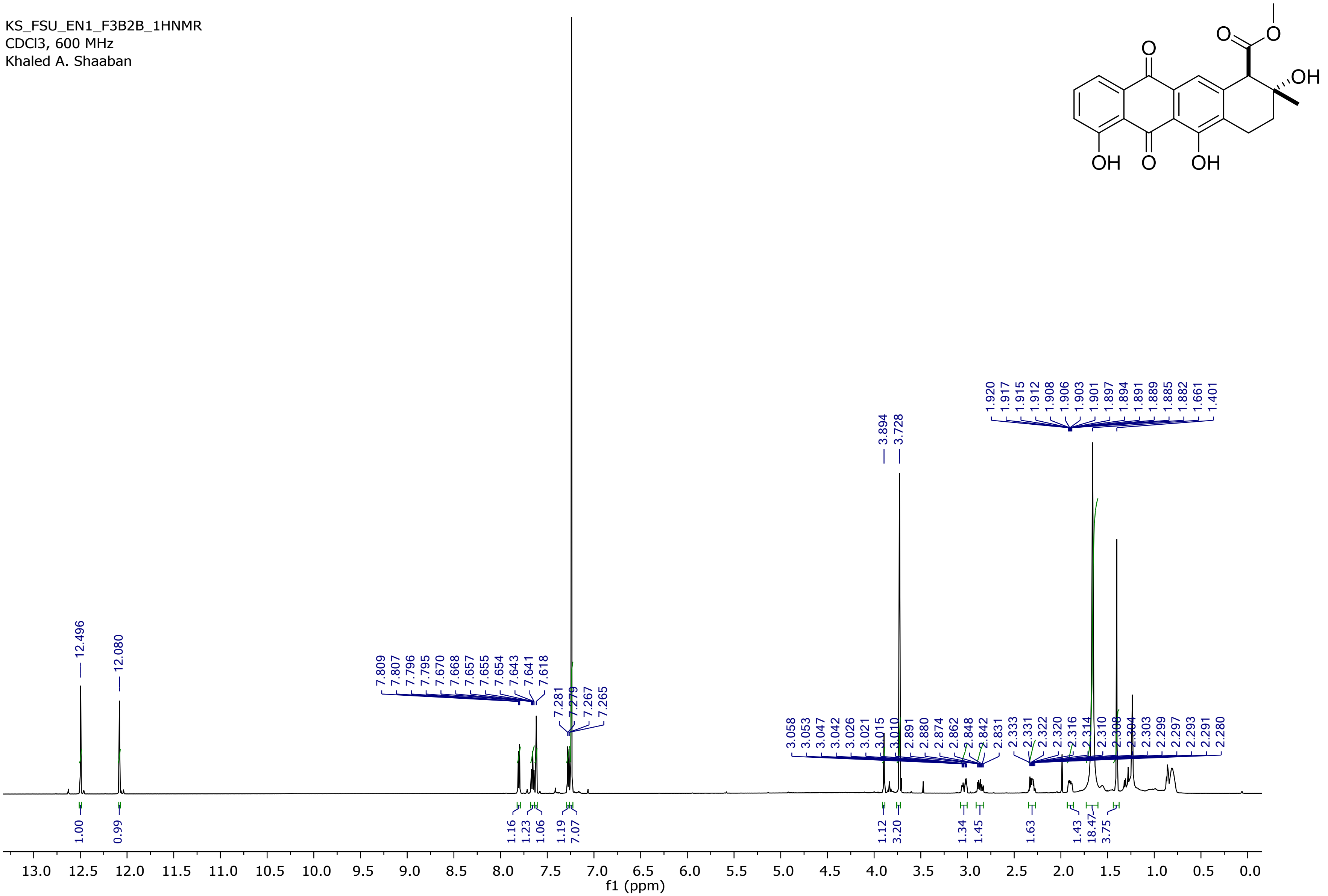

Figure S55. ^{1}H NMR spectrum ($CDCl_3$, 600 MHz) of 7-deoxyauramycinone (9-epi-7-deoxy-nogalamycinone; **4**).

KS_FSU_EN1_F3B2B_13CHNMR
CDCl3, 150 MHz
Khaled A. Shaaban

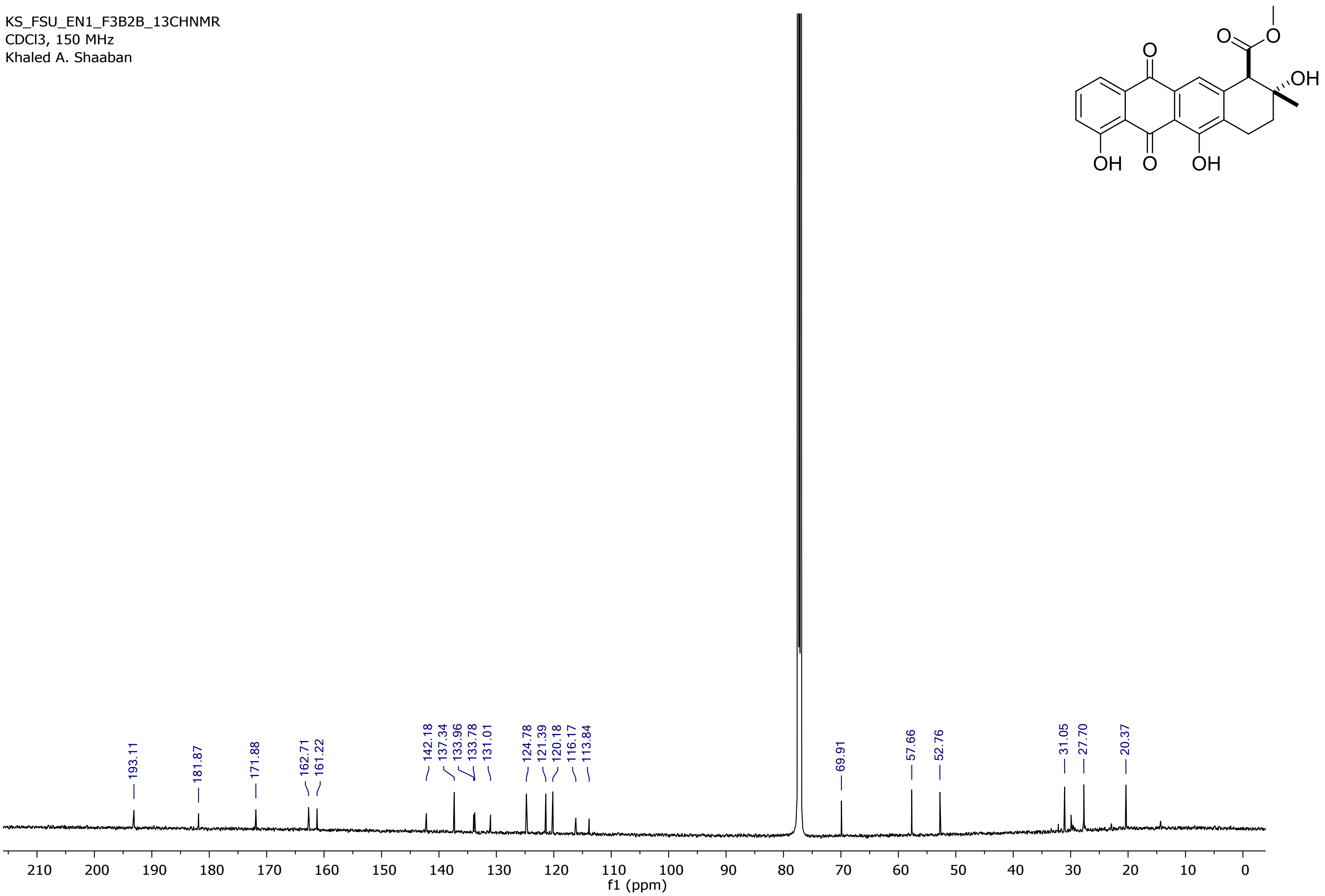

Figure S56. ^{13}C NMR spectrum ($CDCl_3$, 150 MHz) of 7-deoxyauramycinone (9-epi-7-deoxy-nogalamycinone; **4**).

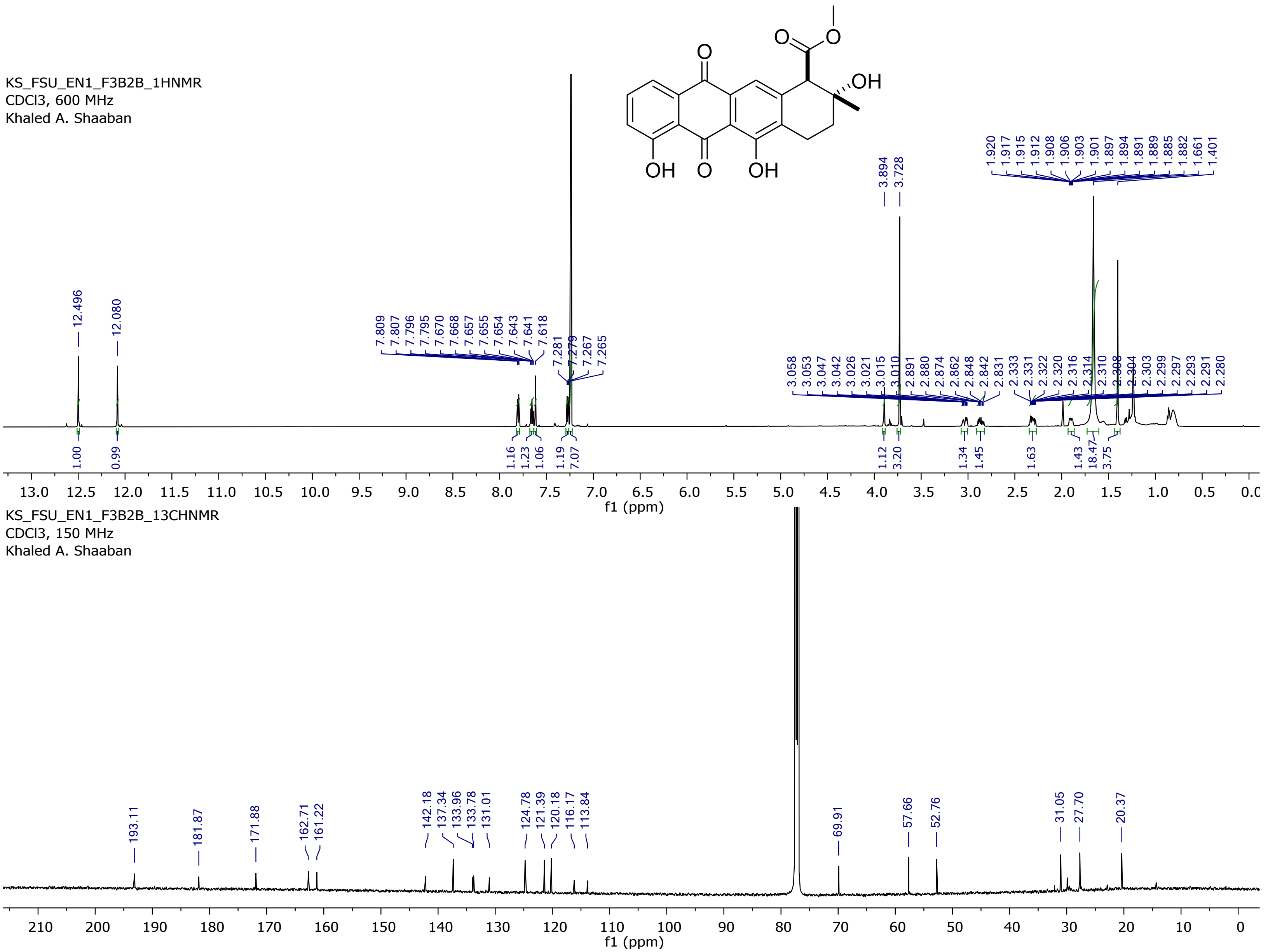

Figure S57. ^{1}H ($CDCl_3$, 600 MHz) and ^{13}C ($CDCl_3$, 150 MHz) NMR spectra of 7-deoxyauramycinone (9-epi-7-deoxy-nogalamycinone; **4**).

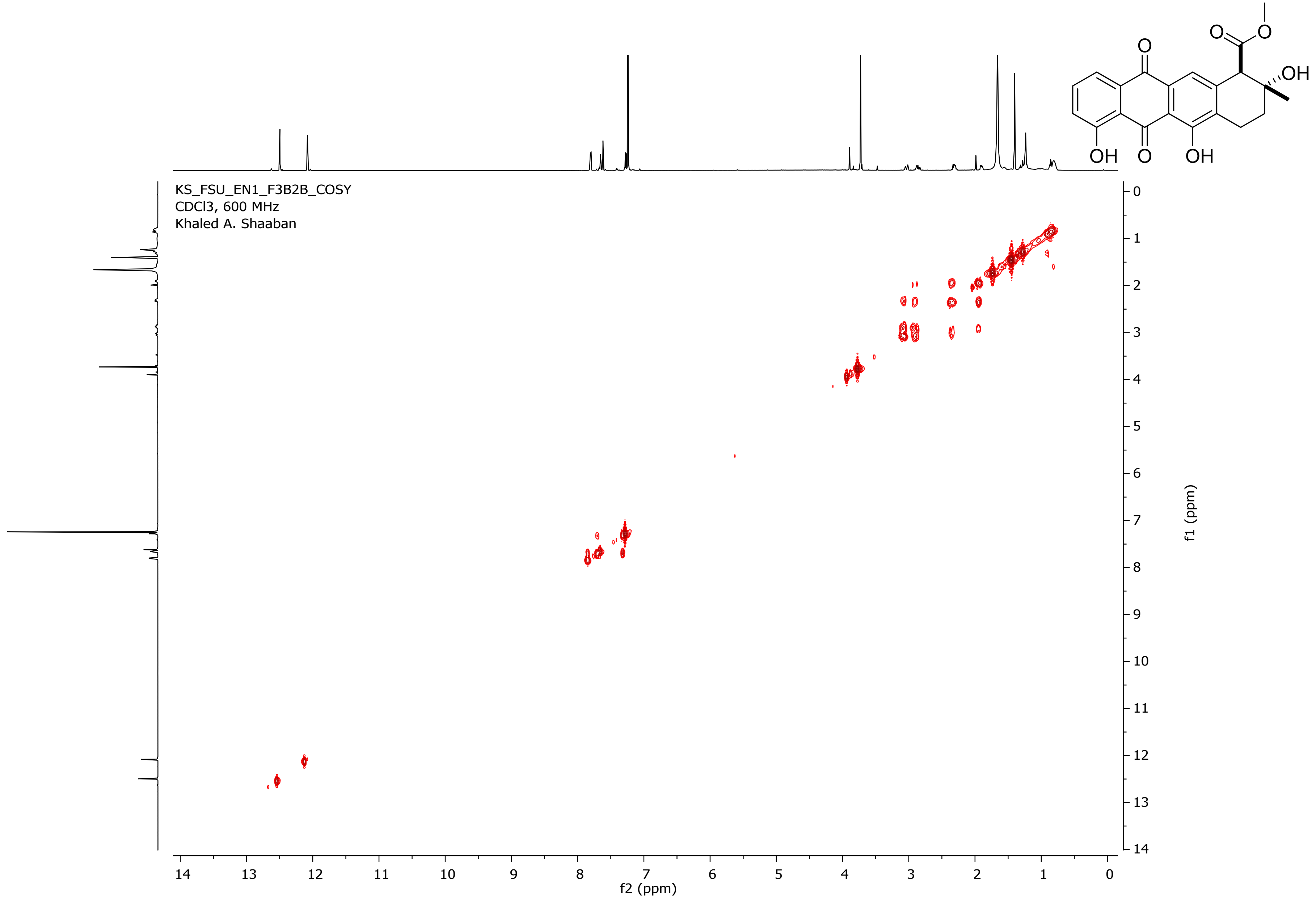

Figure S58. $^{1}H,^{1}H$-COSY spectrum ($CDCl_3$, 600 MHz) of 7-deoxyauramycinone (9-epi-7-deoxy-nogalamycinone; **4**).

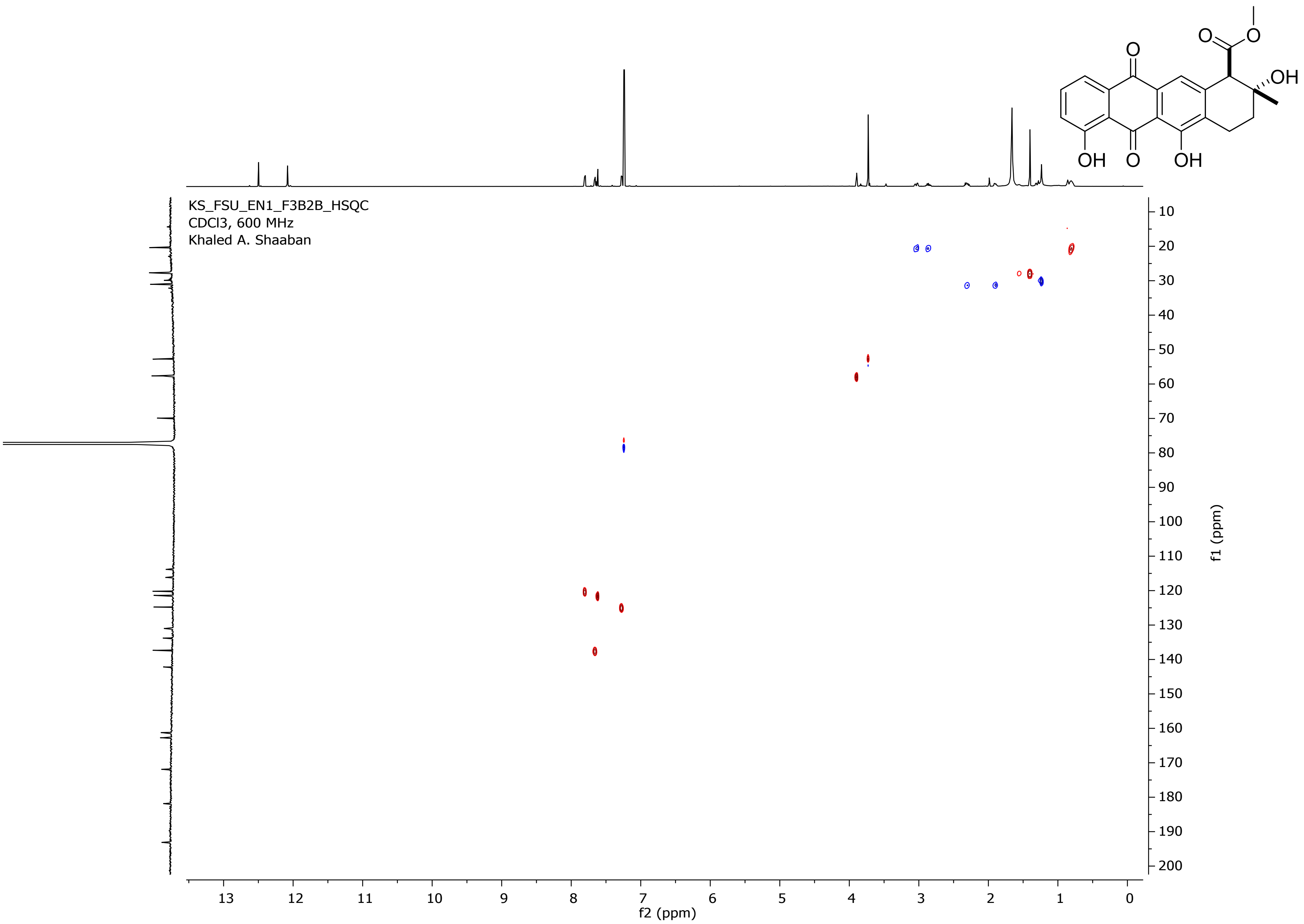

Figure S59. HSQC spectrum ($CDCl_3$, 600 MHz) of 7-deoxyauramycinone (9-epi-7-deoxy-nogalamycinone; **4**).

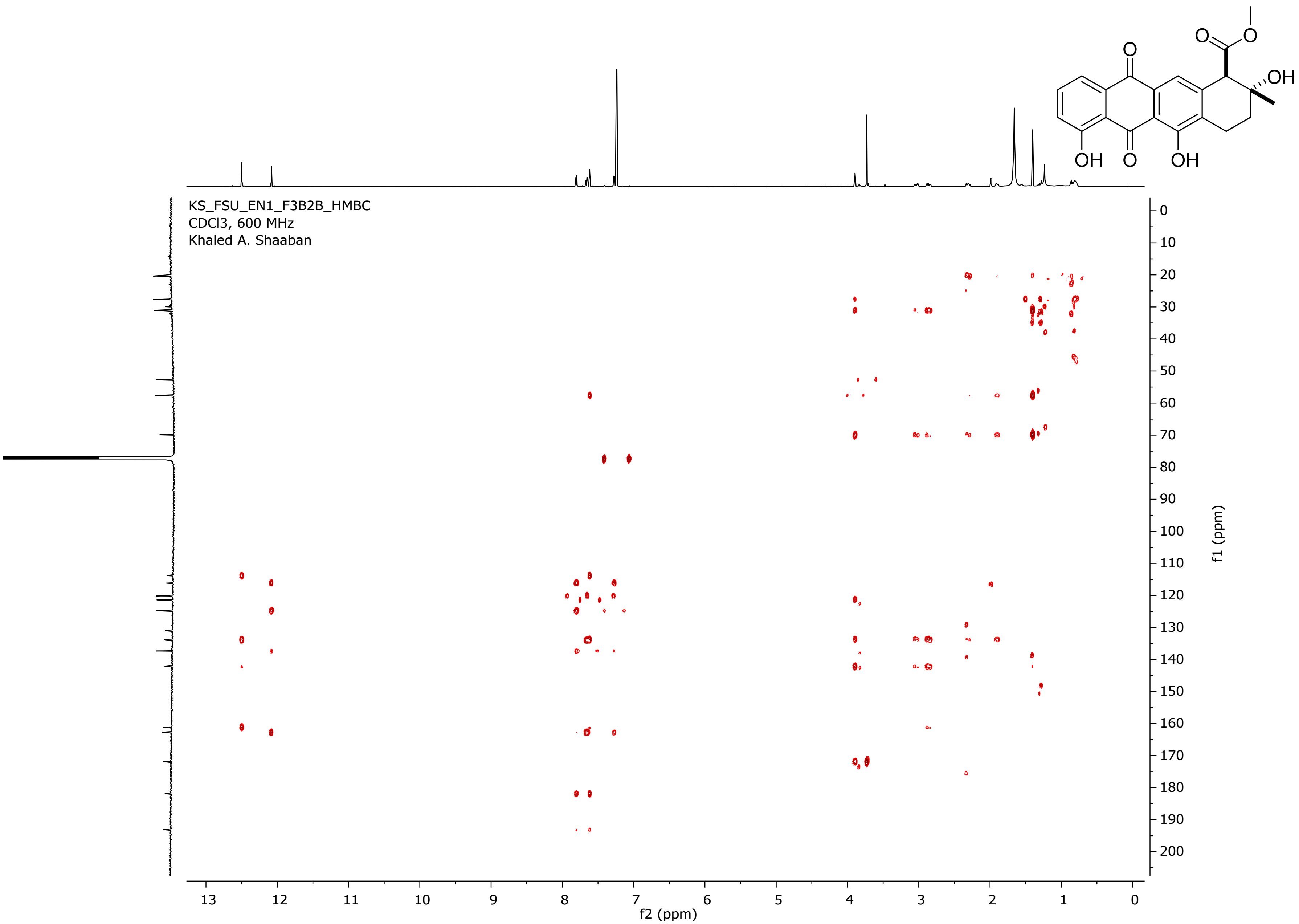

Figure S60. HMBC spectrum ($CDCl_3$, 600 MHz) of 7-deoxyauramycinone (9-epi-7-deoxy-nogalamycinone; **4**).

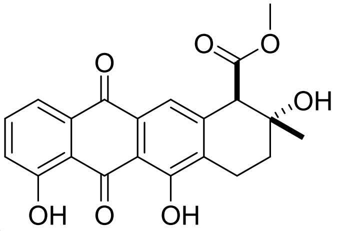

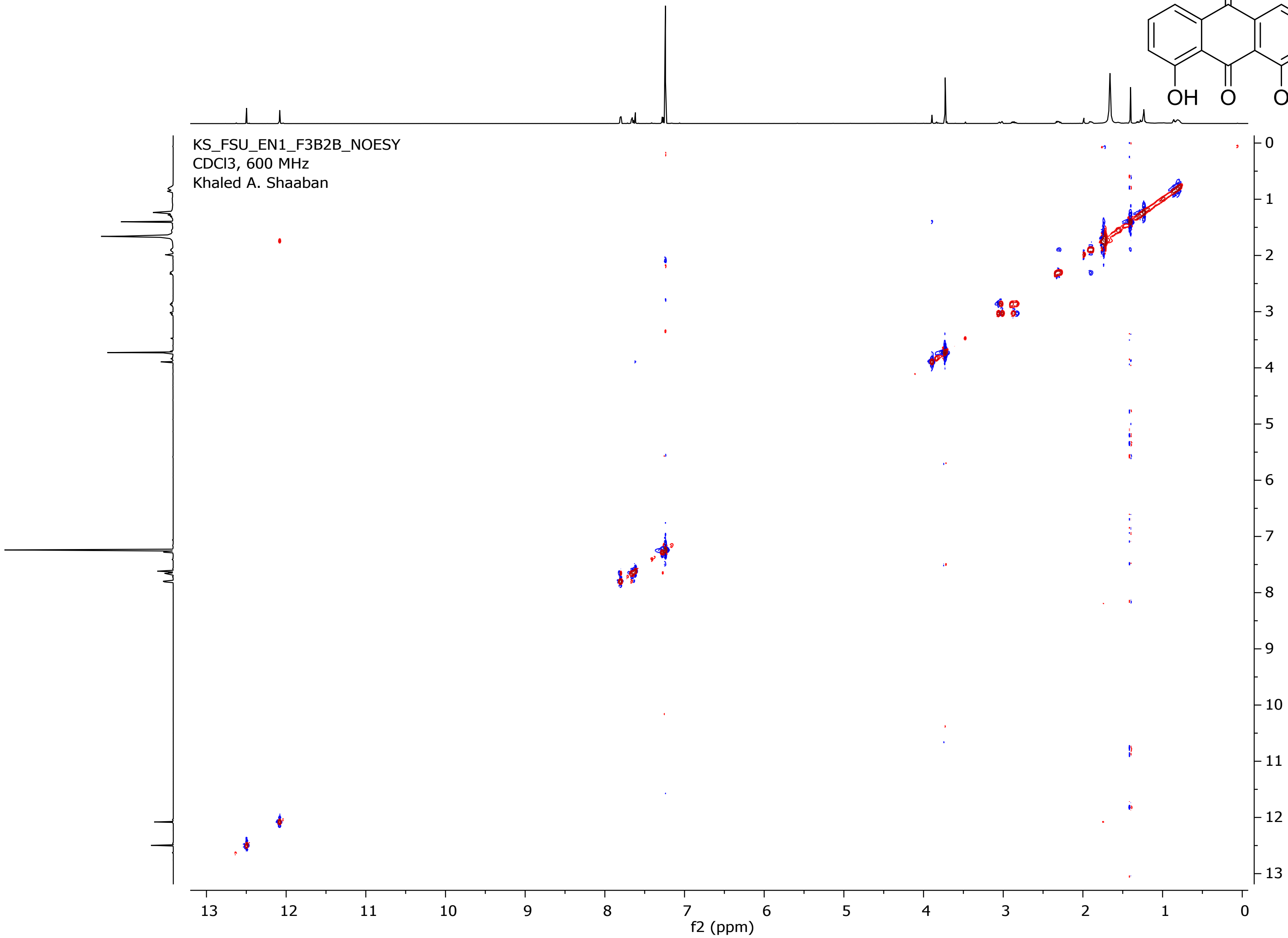

Figure S61. NOESY spectrum ($CDCl_3$, 600 MHz) of 7-deoxyauramycinone (9-epi-7-deoxy-nogalamycinone; **4**).

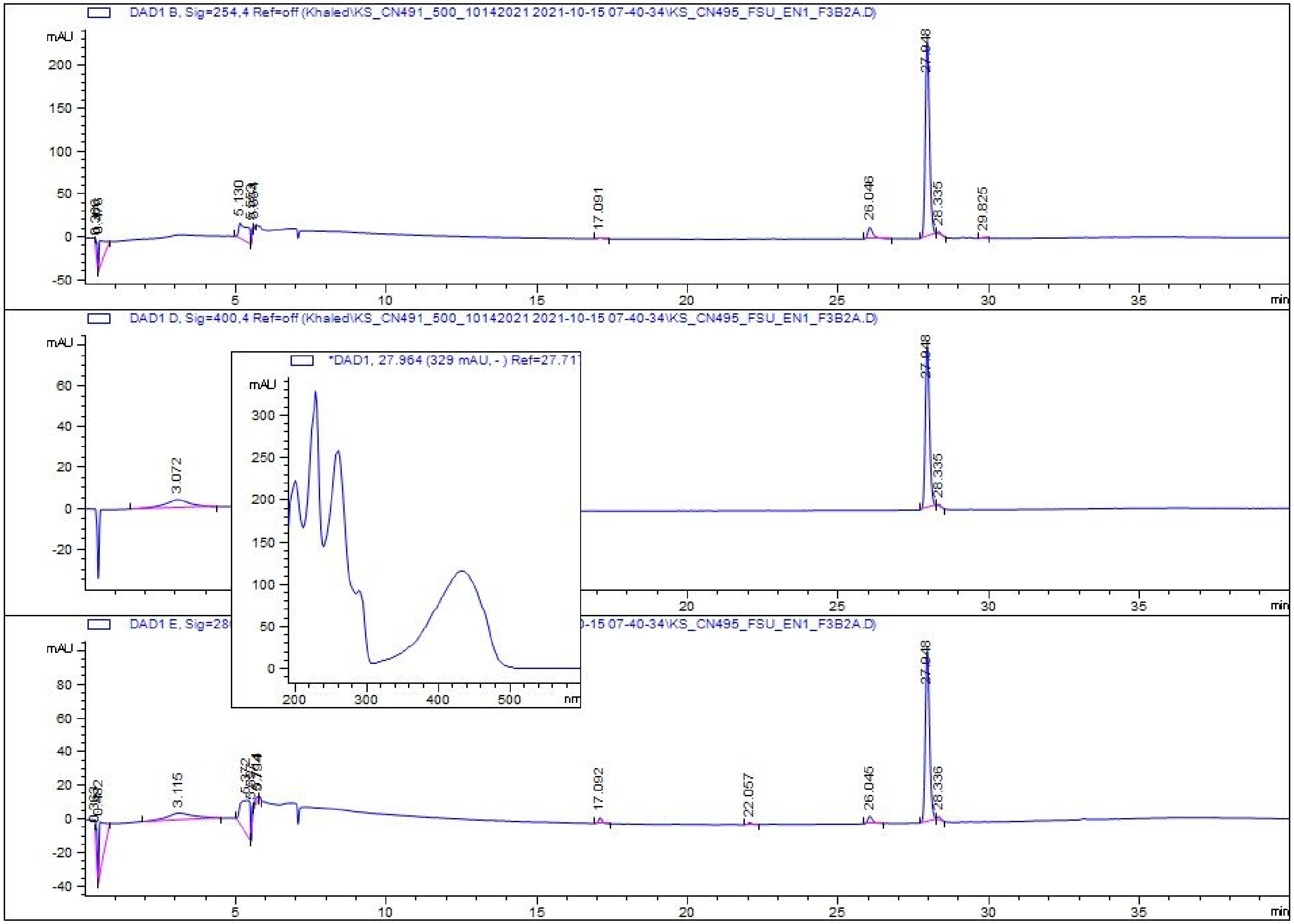

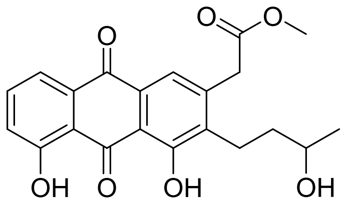

Figure S62: HPLC-UV/vis analysis of 9,10-seco-7-deoxy-nogalamycinone (**5**). HPLC-conditions: solvent A: H2O/0.1% FA; solvent B: CH3CN; flow rate: 0.5 mL min-1; 0-30 min, 5-100% B; 30-35 min, 100% B; 35-36 min, 100-5% B; 36-40 min, 5% B; Phenomenex NX-C18 column (250 × 4.6 mm, 5 µm); 254 nm, 280 nm, 400 nm. UV-vis inset of full wavelength scan (190-600 nm).

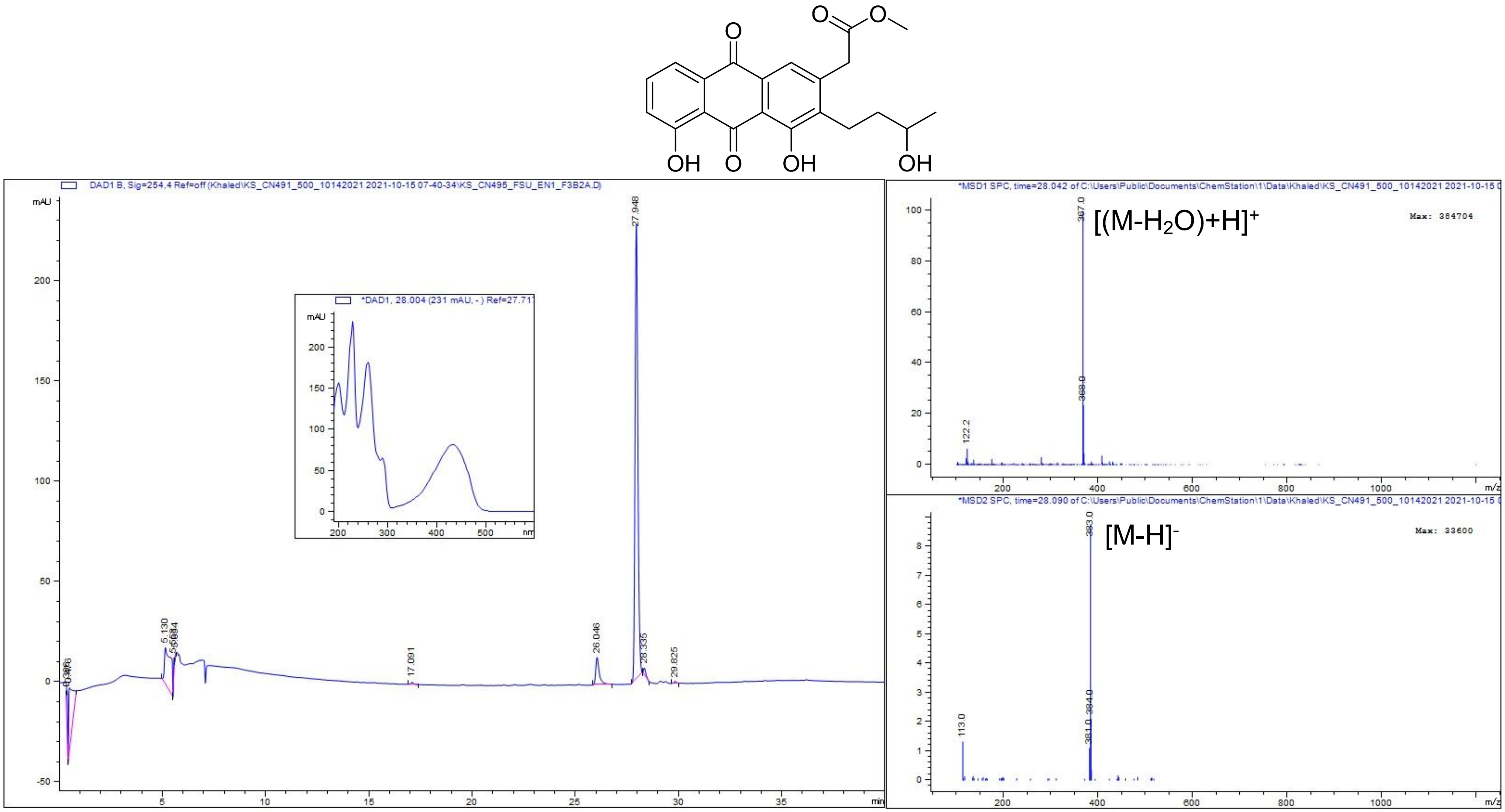

Figure S63: HPLC-MS analysis of 9,10-seco-7-deoxy-nogalamycinone (**5**). HPLC-conditions: solvent A: H2O/0.1% FA; solvent B: CH3CN; flow rate: 0.5 mL min-1; 0-30 min, 5-100% B; 30-35 min, 100% B; 35-36 min, 100-5% B; 36-40 min, 5% B; Phenomenex NX-C18 column (250 × 4.6 mm, 5 µm); 254 nm. UV-vis inset of full wavelength scan (190-600 nm).

**Sample Name**	FSU_EN1_F3B2A	**Position**	P2-A3	**Instrument Name**	Instrument 1
**User Name**		**Inj Vol**	4	**InjPosition**	
**Sample Type**	Sample	**IRM Calibration Status**	Success	**Data Filename**	FSU_EN1_F3B2A.d
**ACQ Method**	Zheng_AQC ACC short_Neg.m	**Comment**		**Acquired Time**	9/21/2021 11:52:01 PM (UTC-04:00)

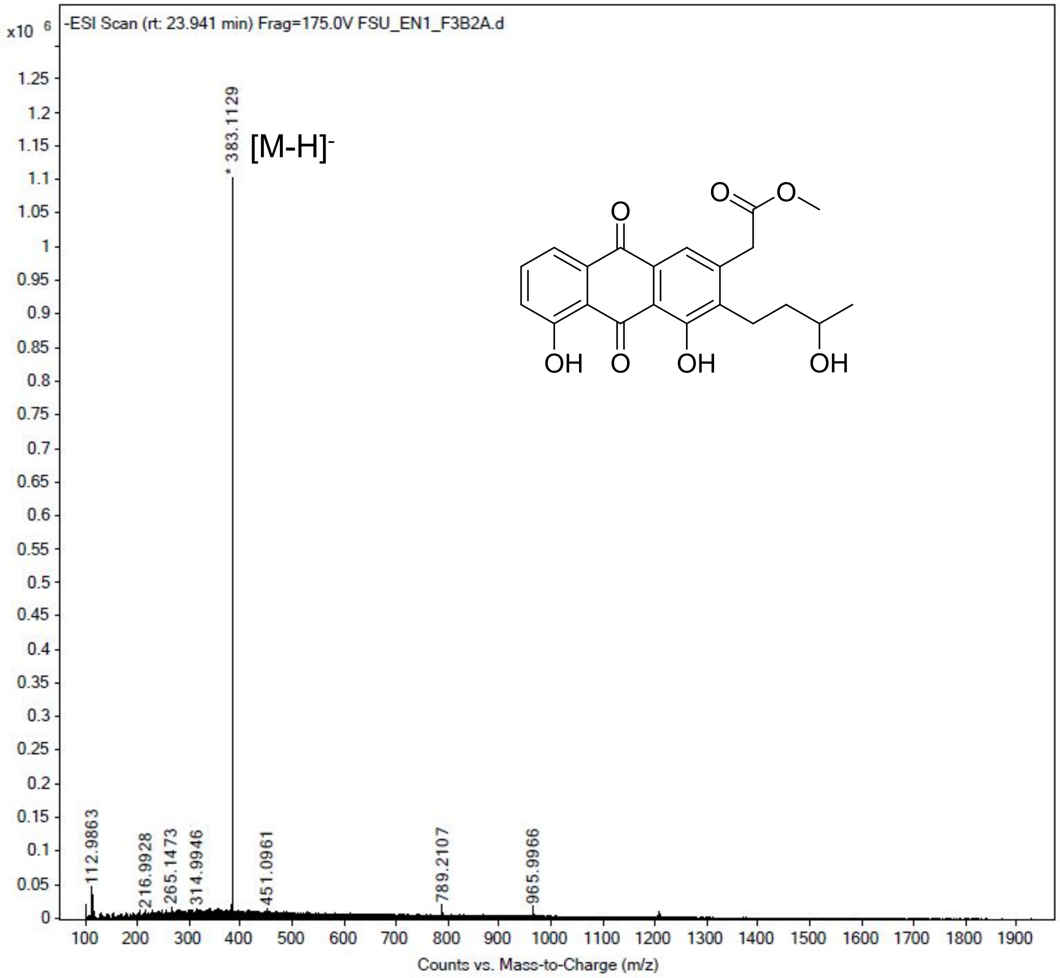

Figure S64. (–)-HRESI-MS spectrum of 9,10-seco-7-deoxy-nogalamycinone (**5**).

**Sample Name**	FSU_EN1_F3B2A	**Position**	P2-A3	**Instrument Name**	Instrument 1
**User Name**		**Inj Vol**	4	**InjPosition**	
**Sample Type**	Sample	**IRM Calibration Status**	Success	**Data Filename**	FSU_EN1_F3B2A.d
**ACQ Method**	Zheng_AQC ACC short_Pos.m	**Comment**		**Acquired Time**	9/22/2021 9:15:44 PM (UTC-04:00)

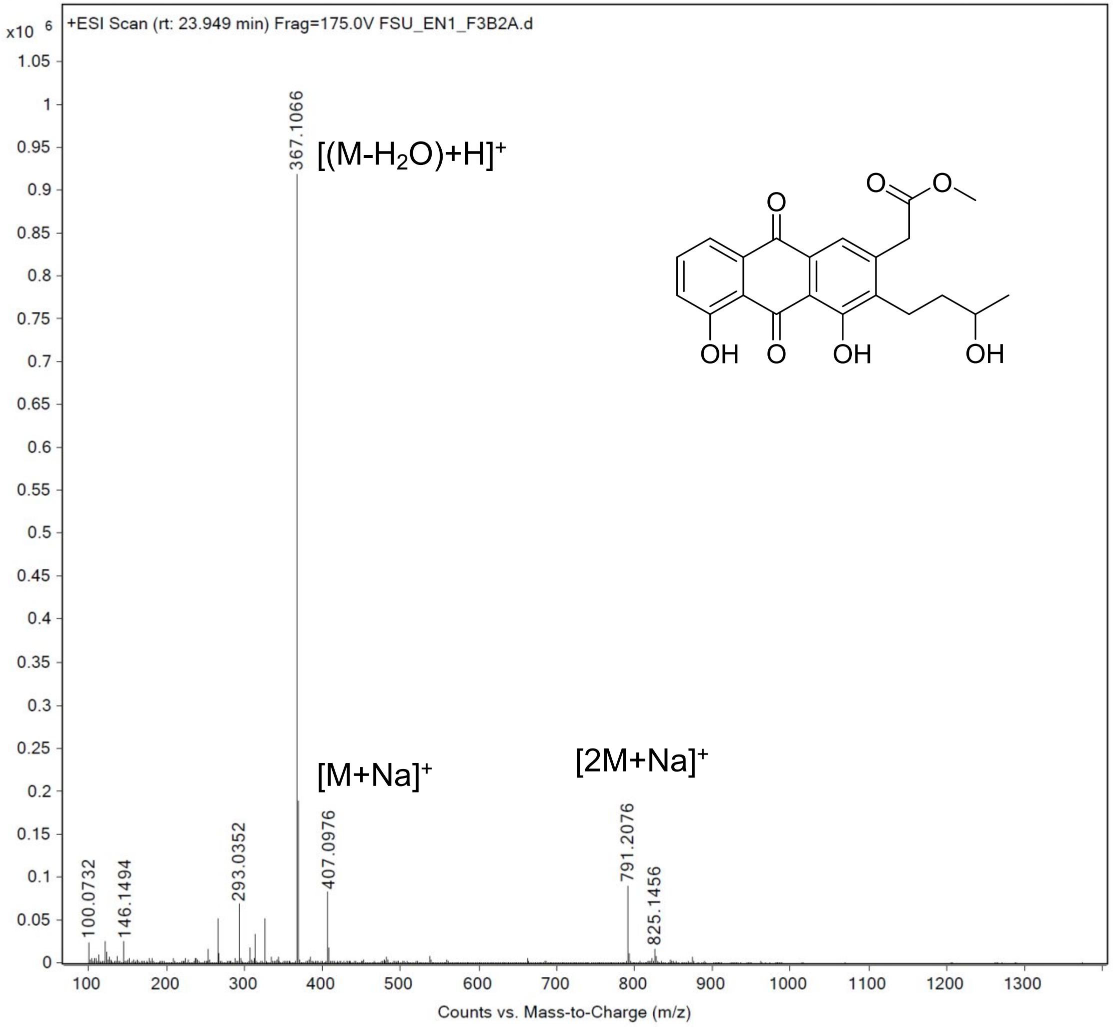

Figure S65. (+)-HRESI-MS spectrum of 9,10-seco-7-deoxy-nogalamycinone (**5**).

KS_FSU_EN1_F3B2A_1HNMR
CDCl3, 600 MHz
Khaled A. Shaaban

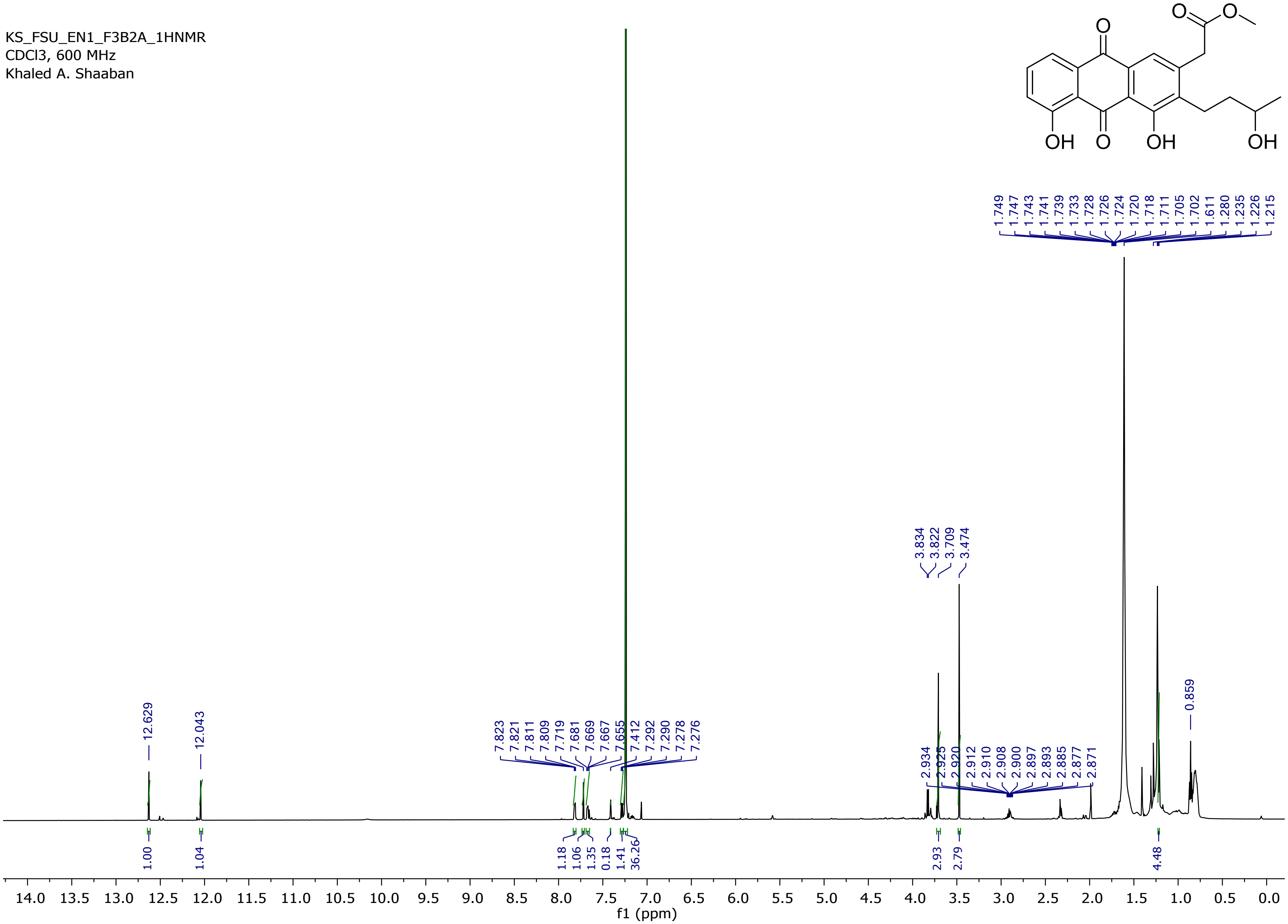

Figure S66. ^{1}H NMR spectrum ($CDCl_3$, 600 MHz) of 9,10-seco-7-deoxy-nogalamycinone (**5**).

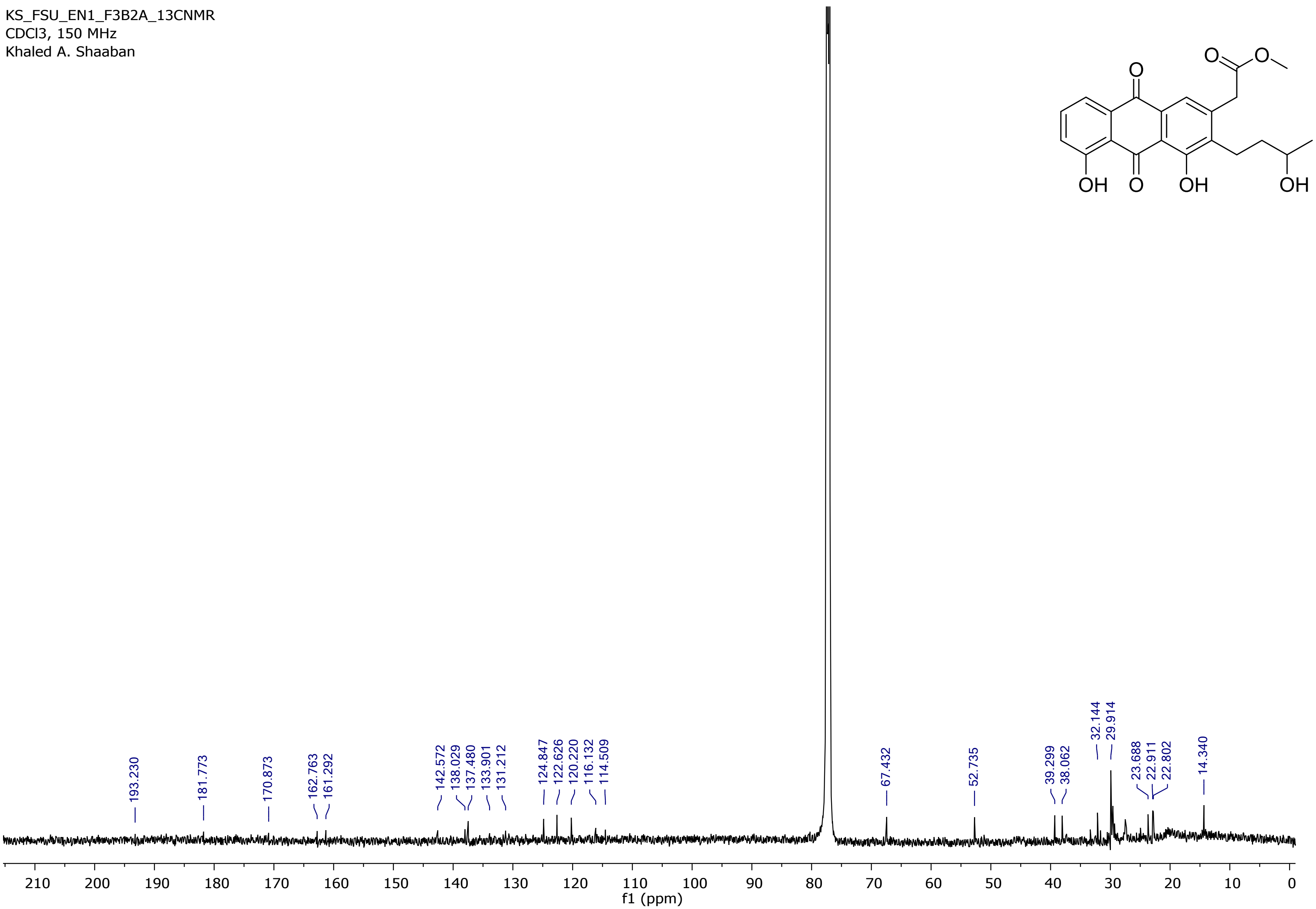

Figure S67. ^{13}C NMR spectrum ($CDCl_3$, 150 MHz) of 9,10-seco-7-deoxy-nogalamycinone (**5**).

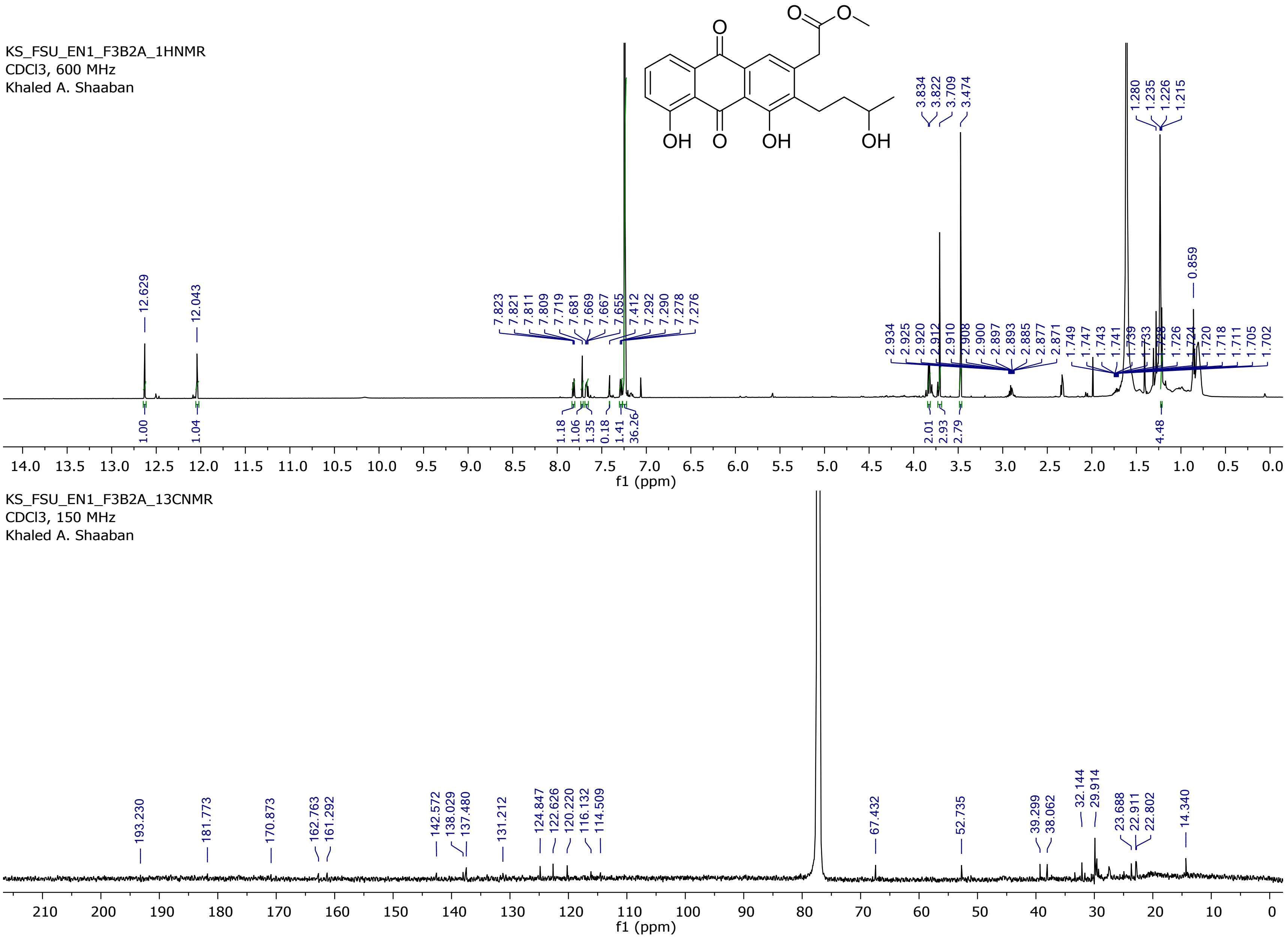

Figure S68. ^{1}H ($CDCl_3$, 600 MHz) and ^{13}C ($CDCl_3$, 150 MHz) NMR spectra of 9,10-seco-7-deoxy-nogalamycinone (**5**).

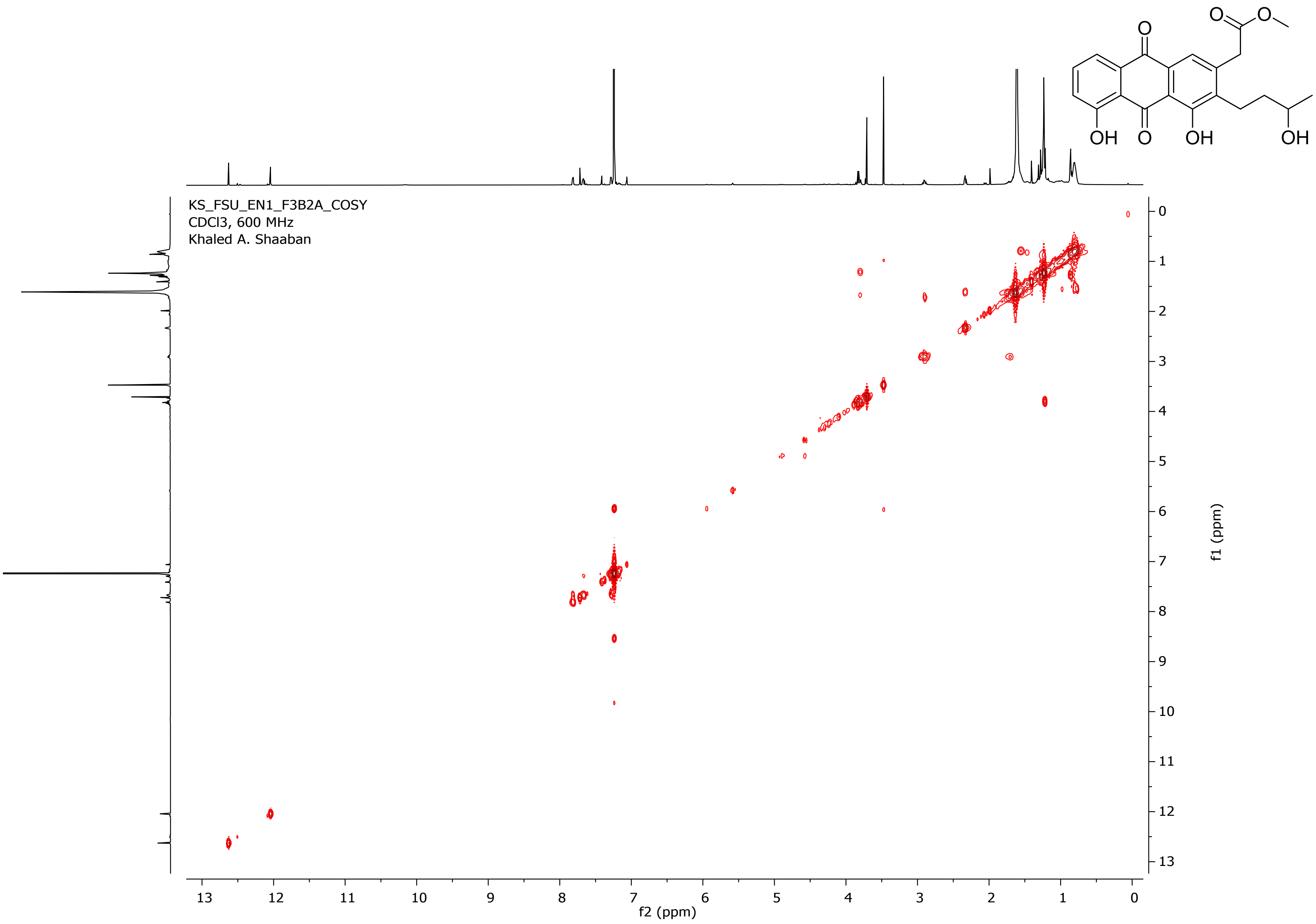

Figure S69. $^1H,^1H$-COSY spectrum ($CDCl_3$, 600 MHz) of 9,10-seco-7-deoxy-nogalamycinone (**5**).

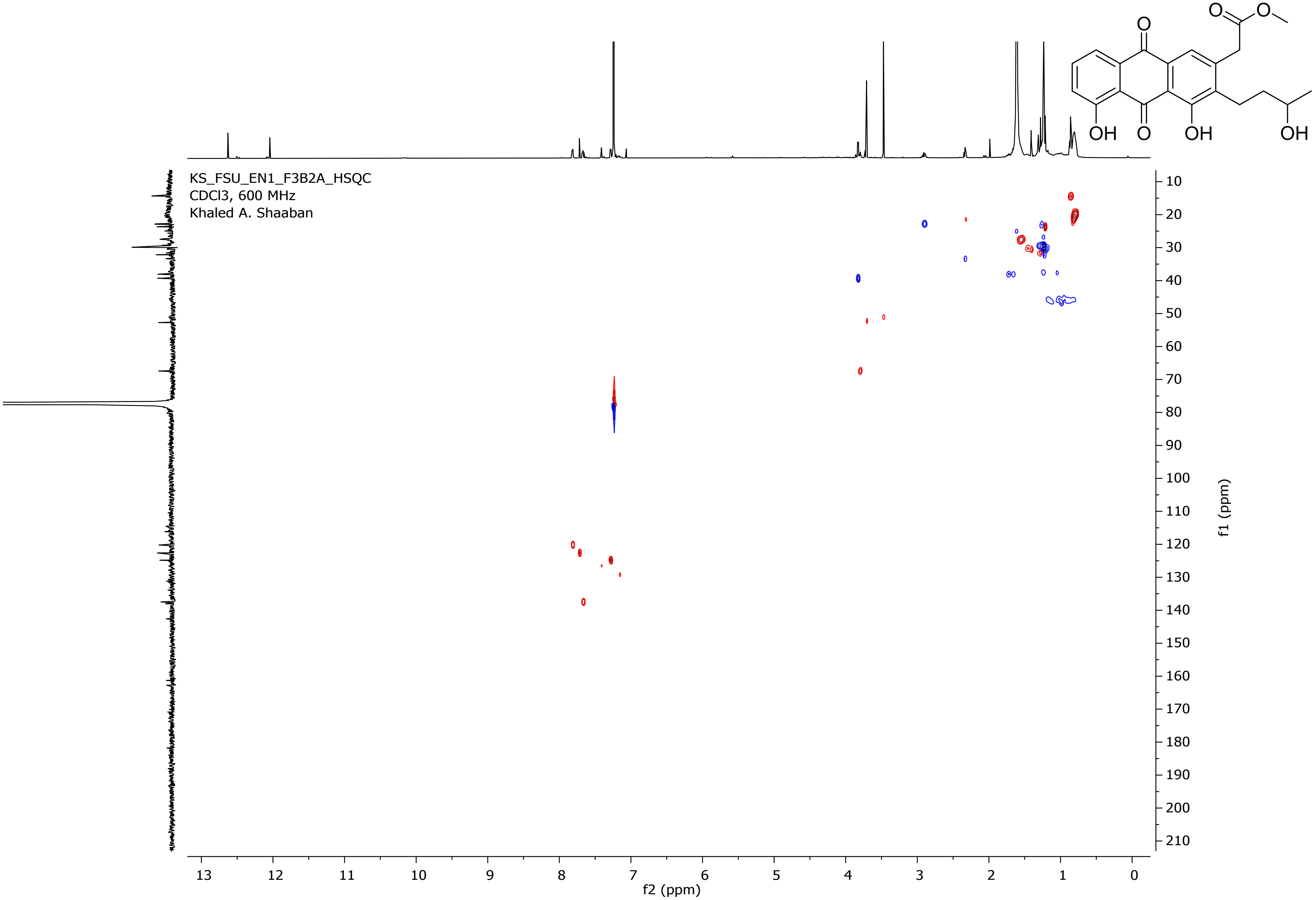

Figure S70. HSQC spectrum ($CDCl_3$, 600 MHz) of 9,10-seco-7-deoxy-nogalamycinone (**5**).

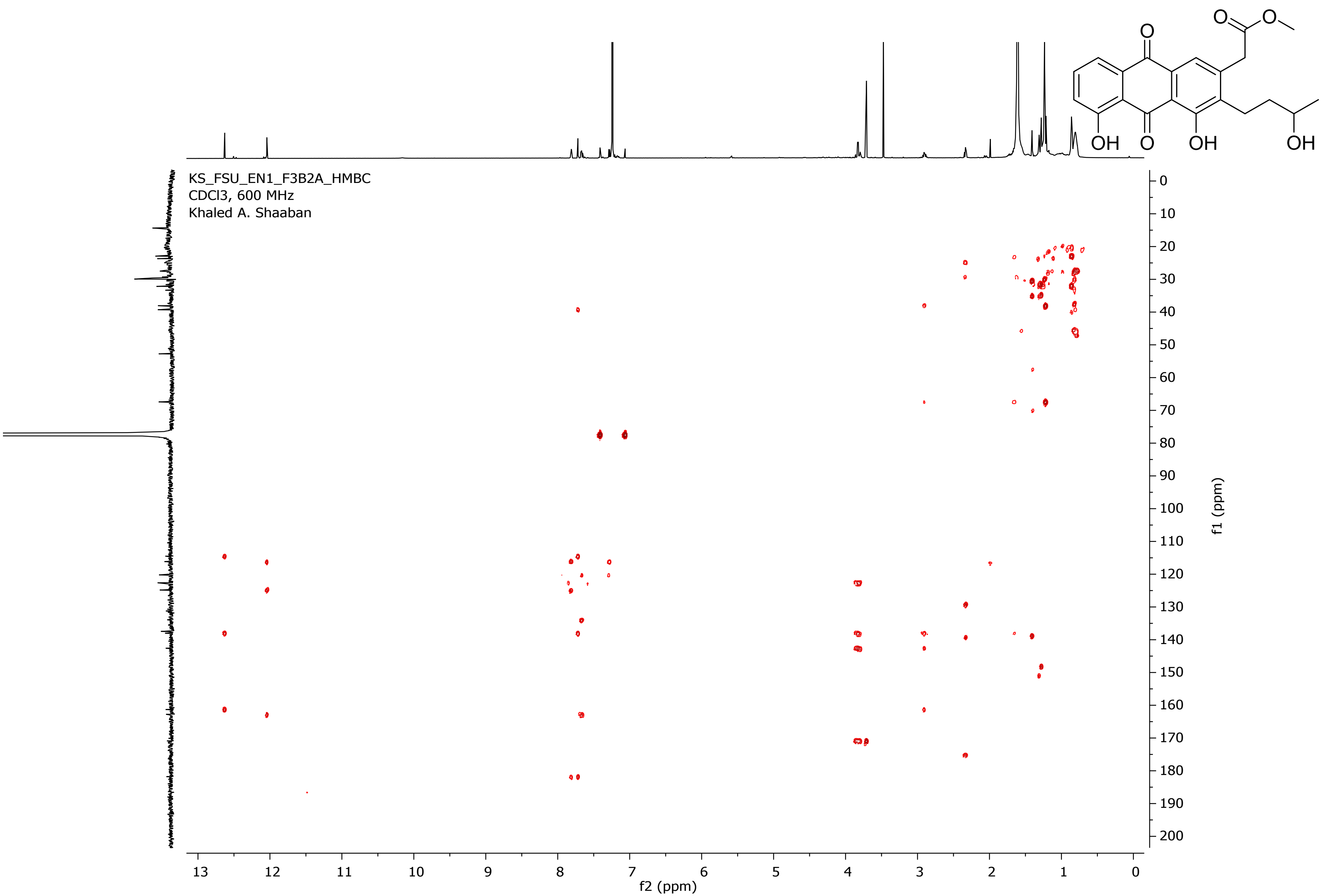

Figure S71. HMBC spectrum ($CDCl_3$, 600 MHz) of 9,10-seco-7-deoxy-nogalamycinone (5).

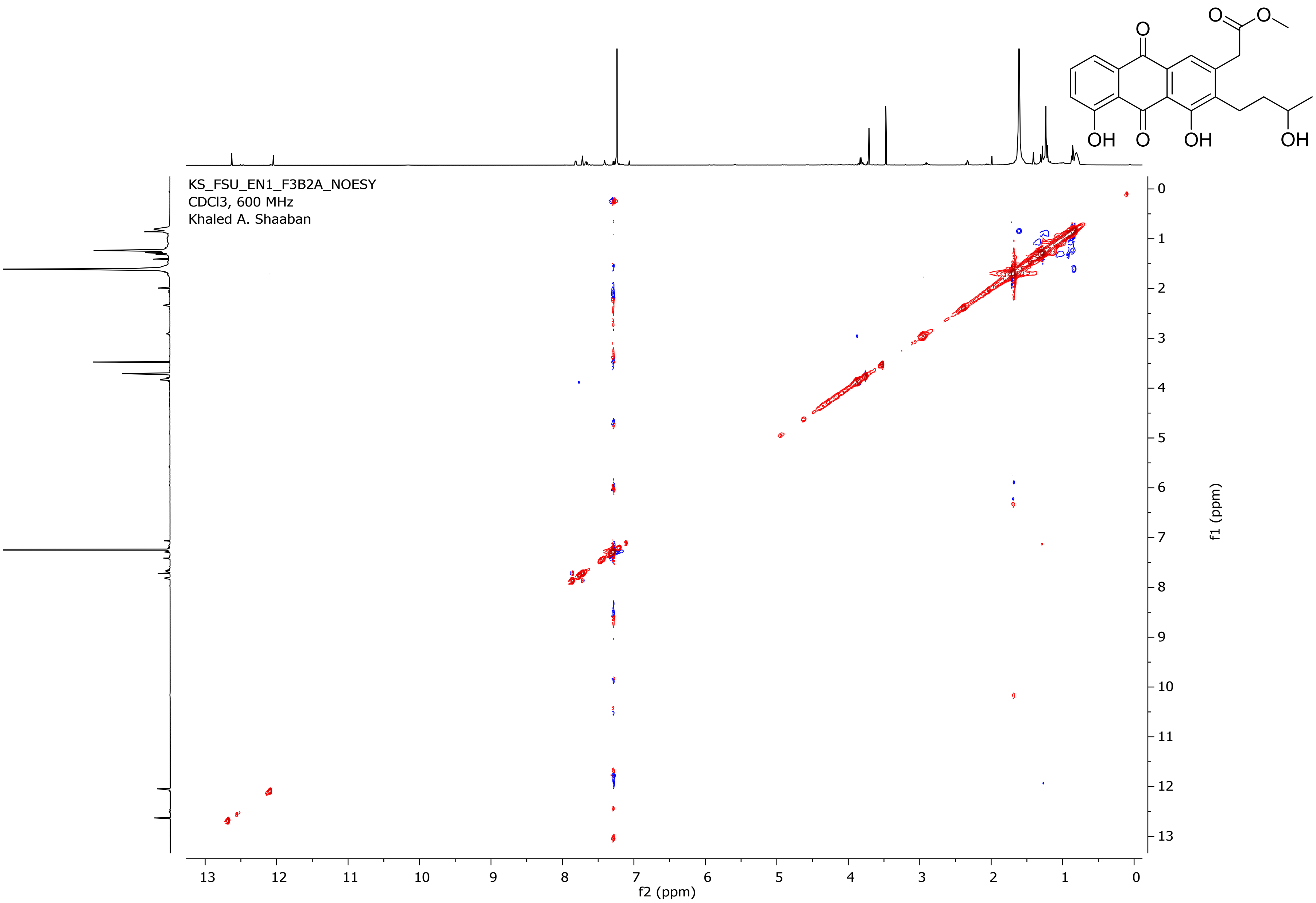

Figure S72. NOESY spectrum ($CDCl_3$, 600 MHz) of 9,10-seco-7-deoxy-nogalamycinone (**5**).

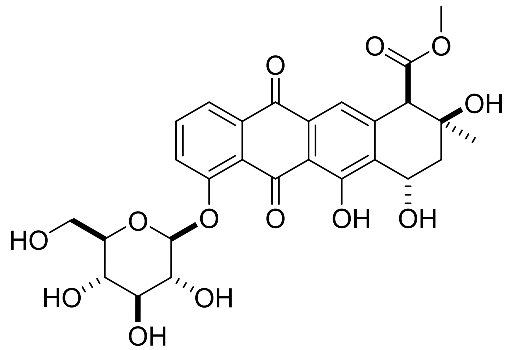

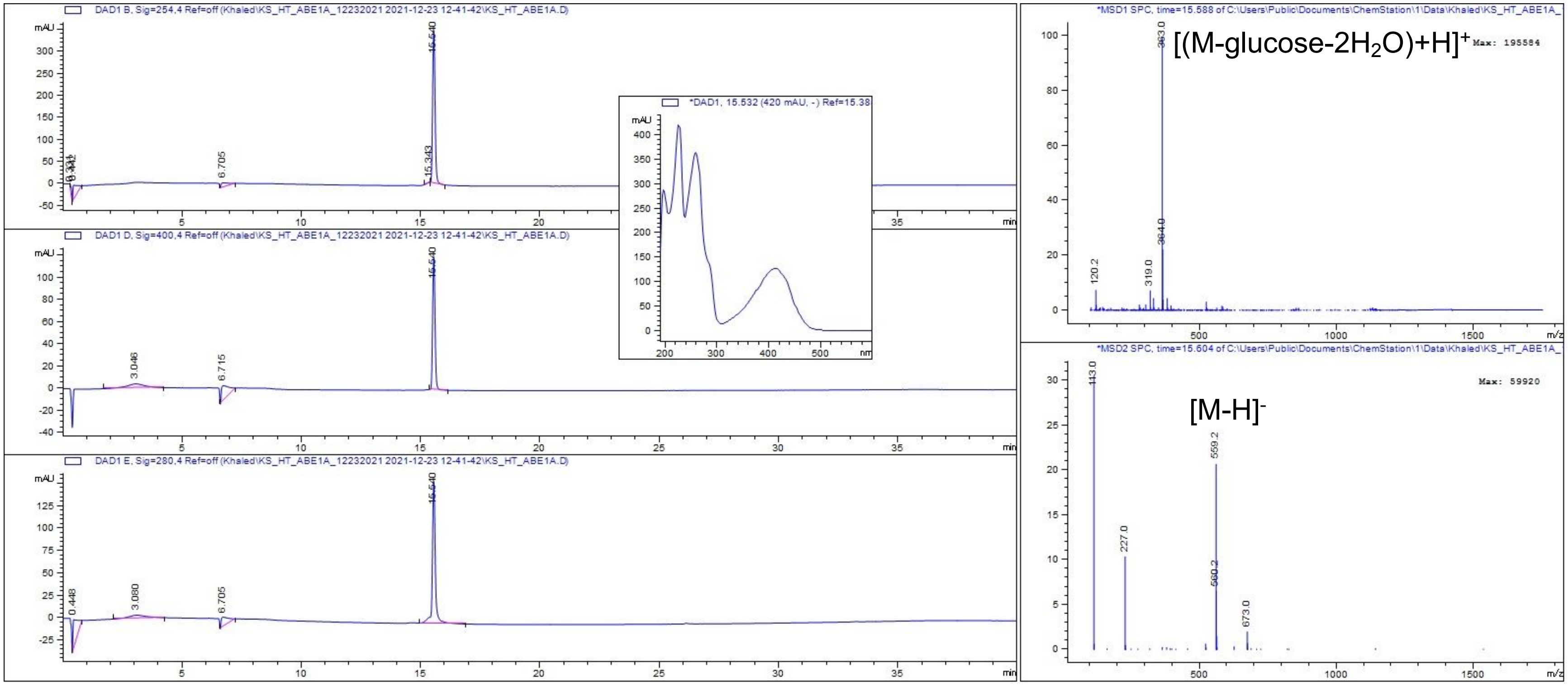

Figure S73: HPLC-MS analysis of 4-β-D-glucosyl-nogalamycinone (6). HPLC-conditions: solvent A: H2O/0.1% FA; solvent B: CH3CN; flow rate: 0.5 mL min-1; 0-30 min, 5-100% B; 30-35 min, 100% B; 35-36 min, 100-5% B; 36-40 min, 5% B; Phenomenex NX-C18 column (250 × 4.6 mm, 5 µm); 254 nm, 280 nm, 400 nm. UV-vis inset of full wavelength scan (190-600 nm).

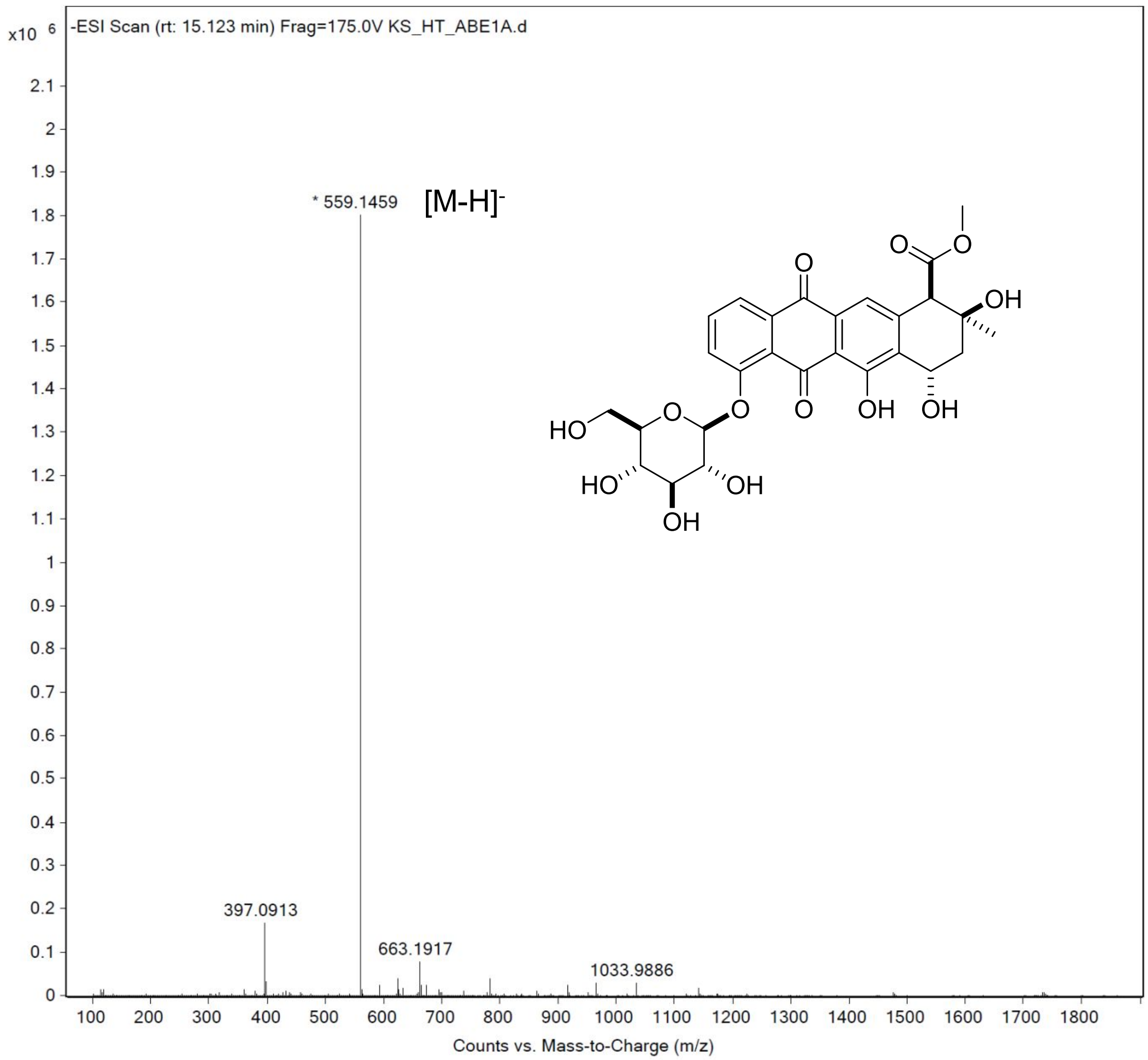

Figure S74. (–)-HRESI-MS spectrum of **4-β-D-glucosyl-nogalamycinone (6).**

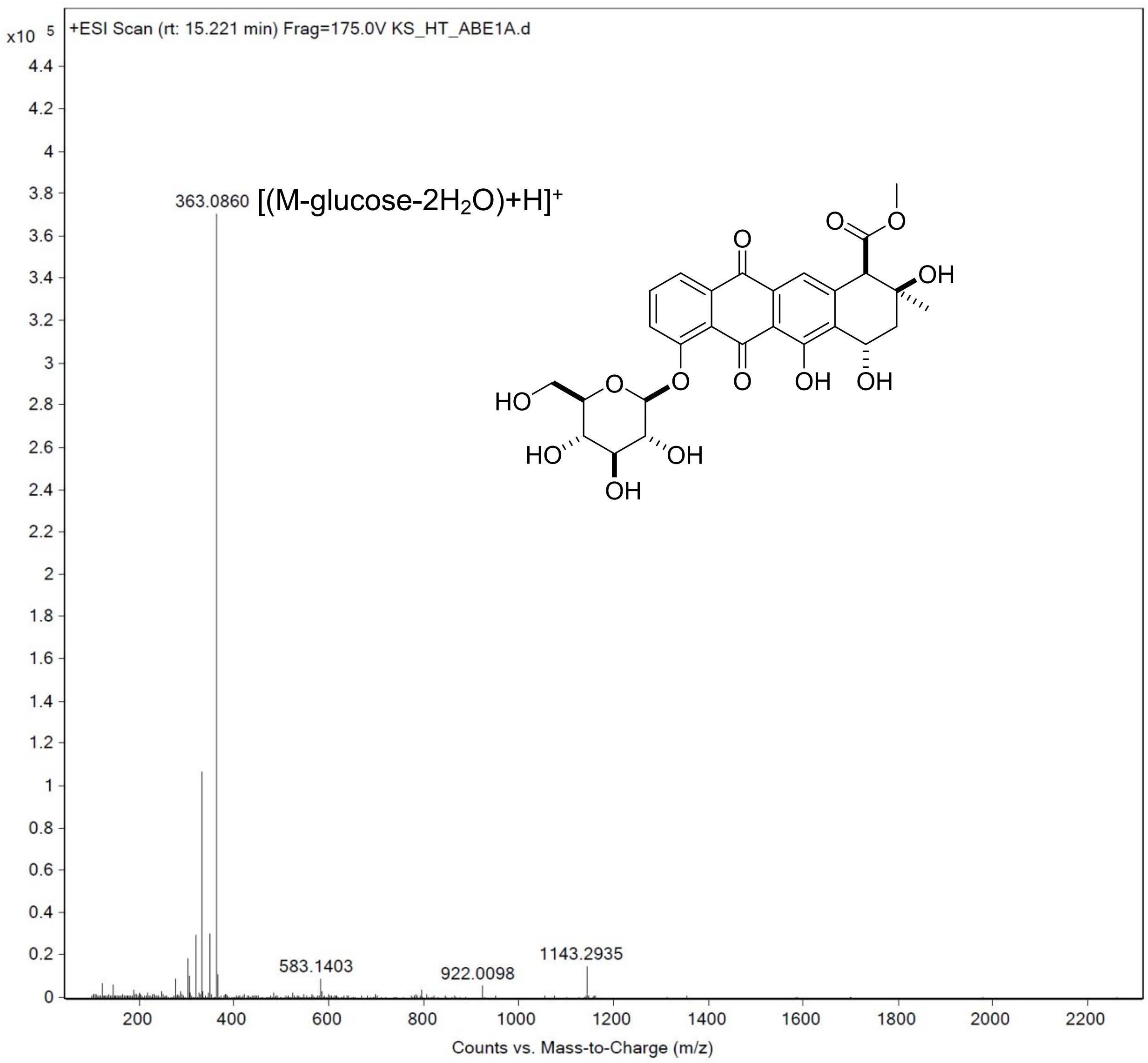

Figure S75. (+)-HRESI-MS spectrum of 4-β-D-glucosyl-nogalamycinone (**6**).

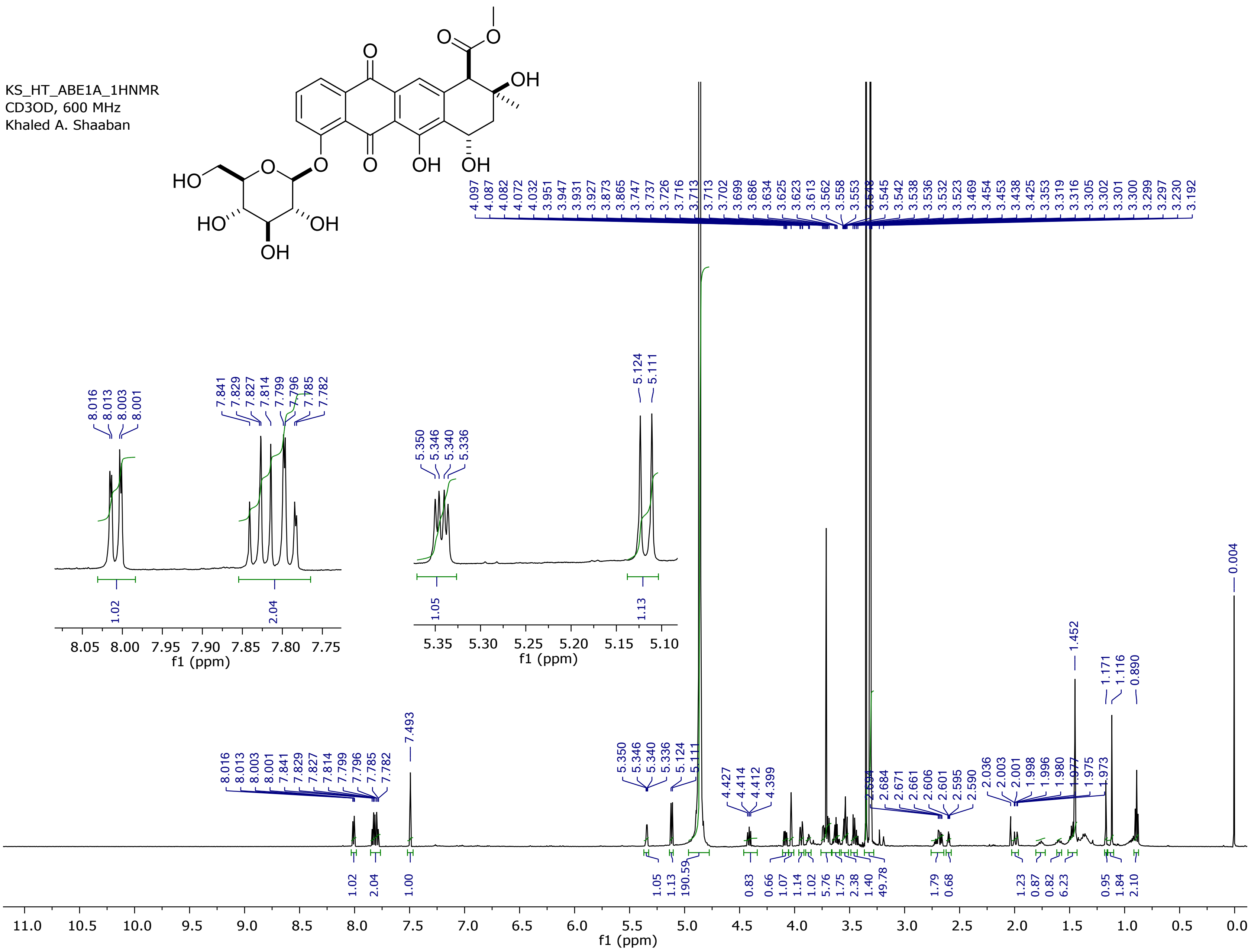

Figure S76. ^{1}H NMR spectrum (CD_3OD, 600 MHz) of **4-β-D-glucosyl-nogalamycinone** (**6**).

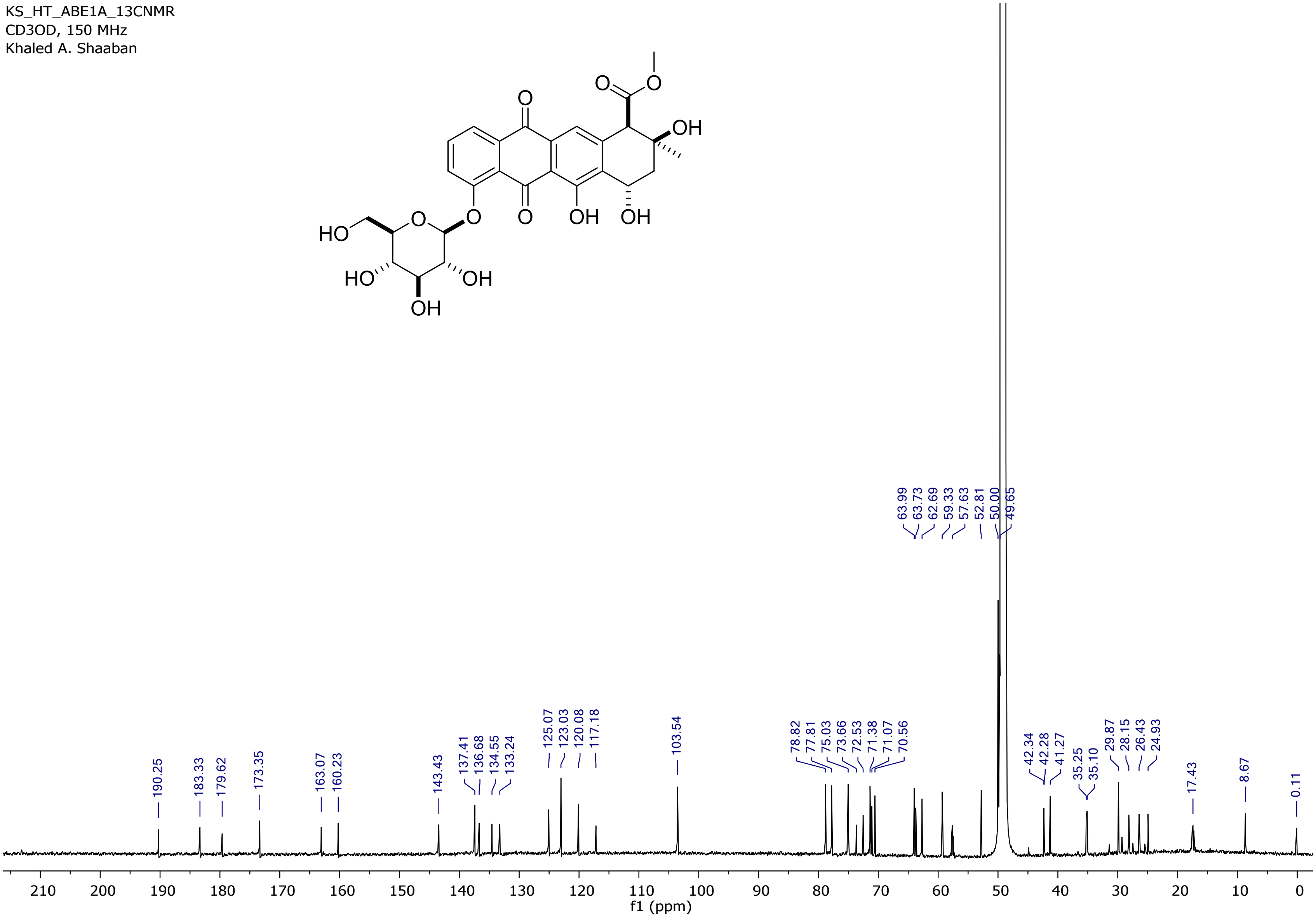

Figure S77. ^{13}C NMR spectrum (CD_3OD, 150 MHz) of 4-β-D-glucosyl-nogalamycinone (**6**).

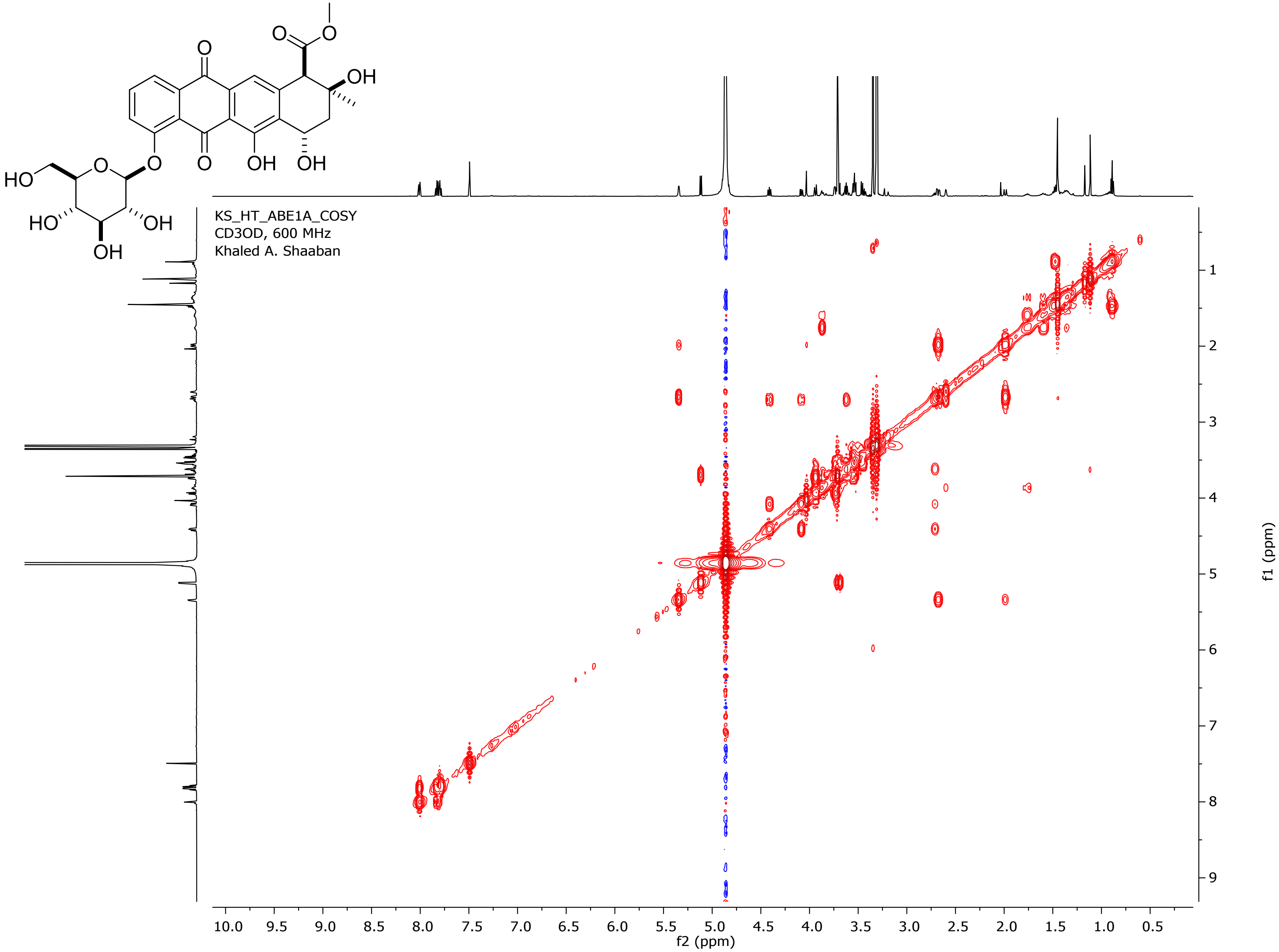

Figure S78. ^{1}H,^{1}H-COSY spectrum (CD_3OD, 600 MHz) of **4-β-D-glucosyl-nogalamycinone** (**6**).

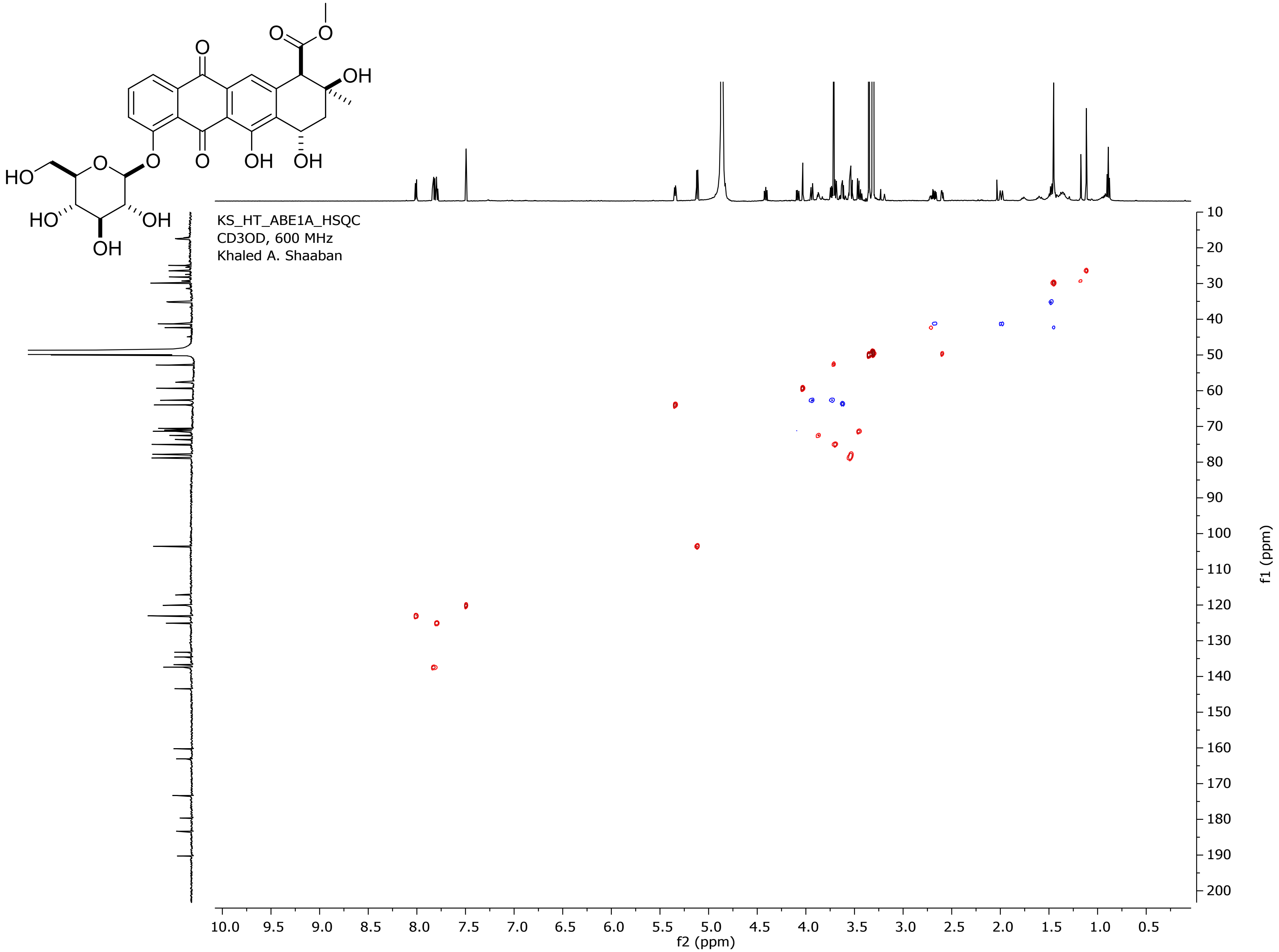

Figure S79. HSQC spectrum (CD_3OD, 600 MHz) of 4-β-D-glucosyl-nogalamycinone (**6**).

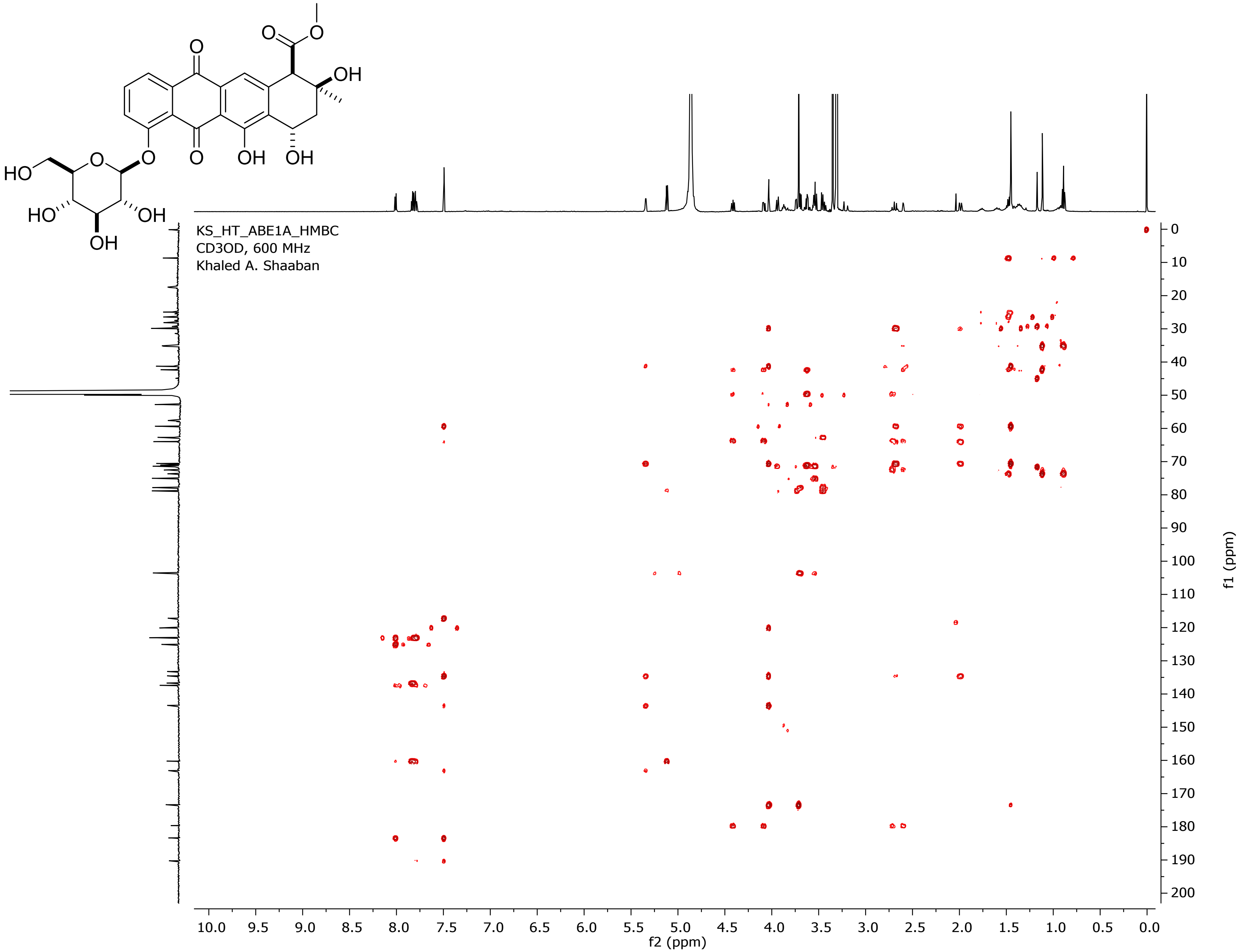

Figure S80. HMBC spectrum (CD_3OD, 600 MHz) of **4-β-D-glucosyl-nogalamycinone** (**6**).

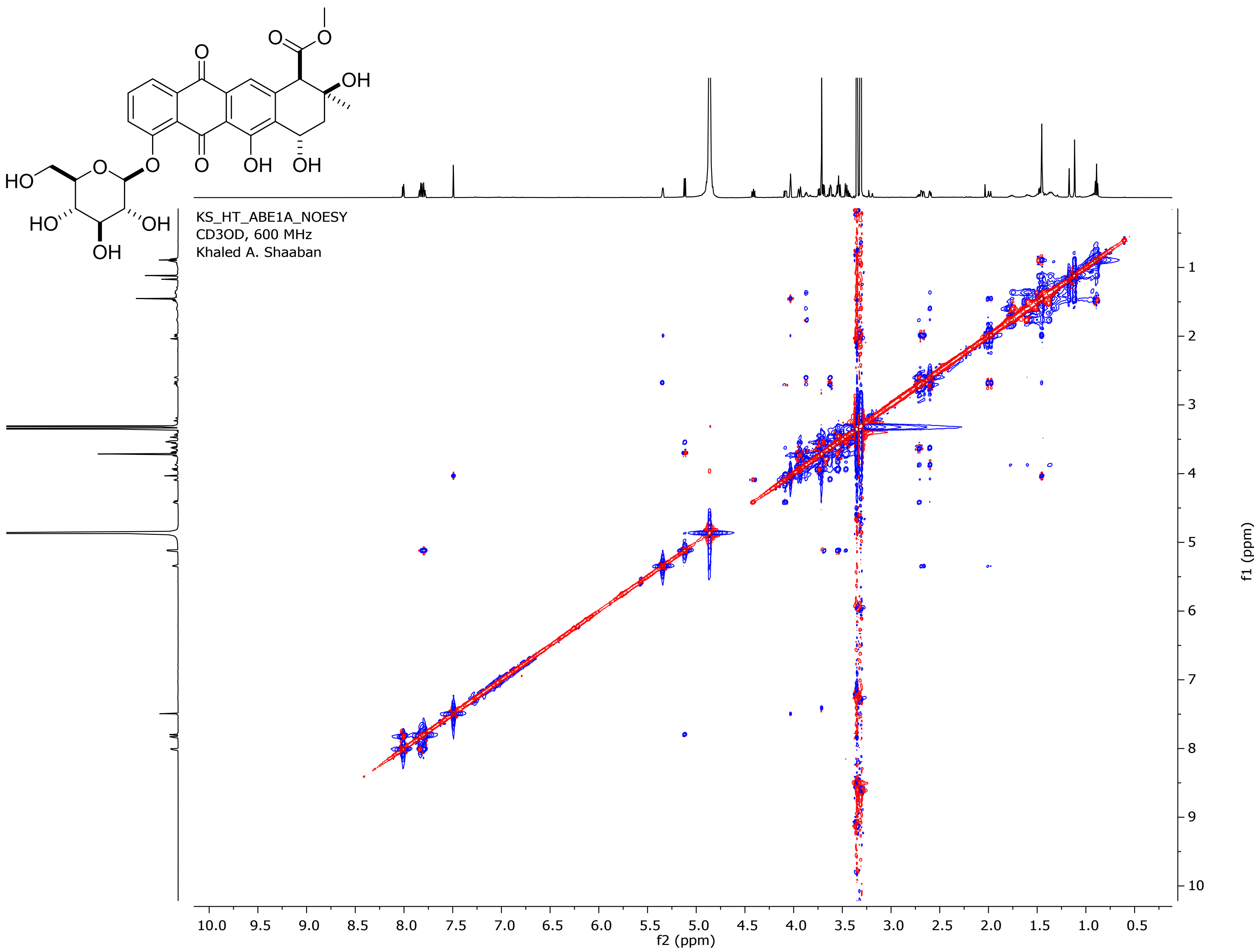

Figure S81. NOESY spectrum (CD_3OD, 600 MHz) of **4-β-D-glucosyl-nogalamycinone** (**6**).

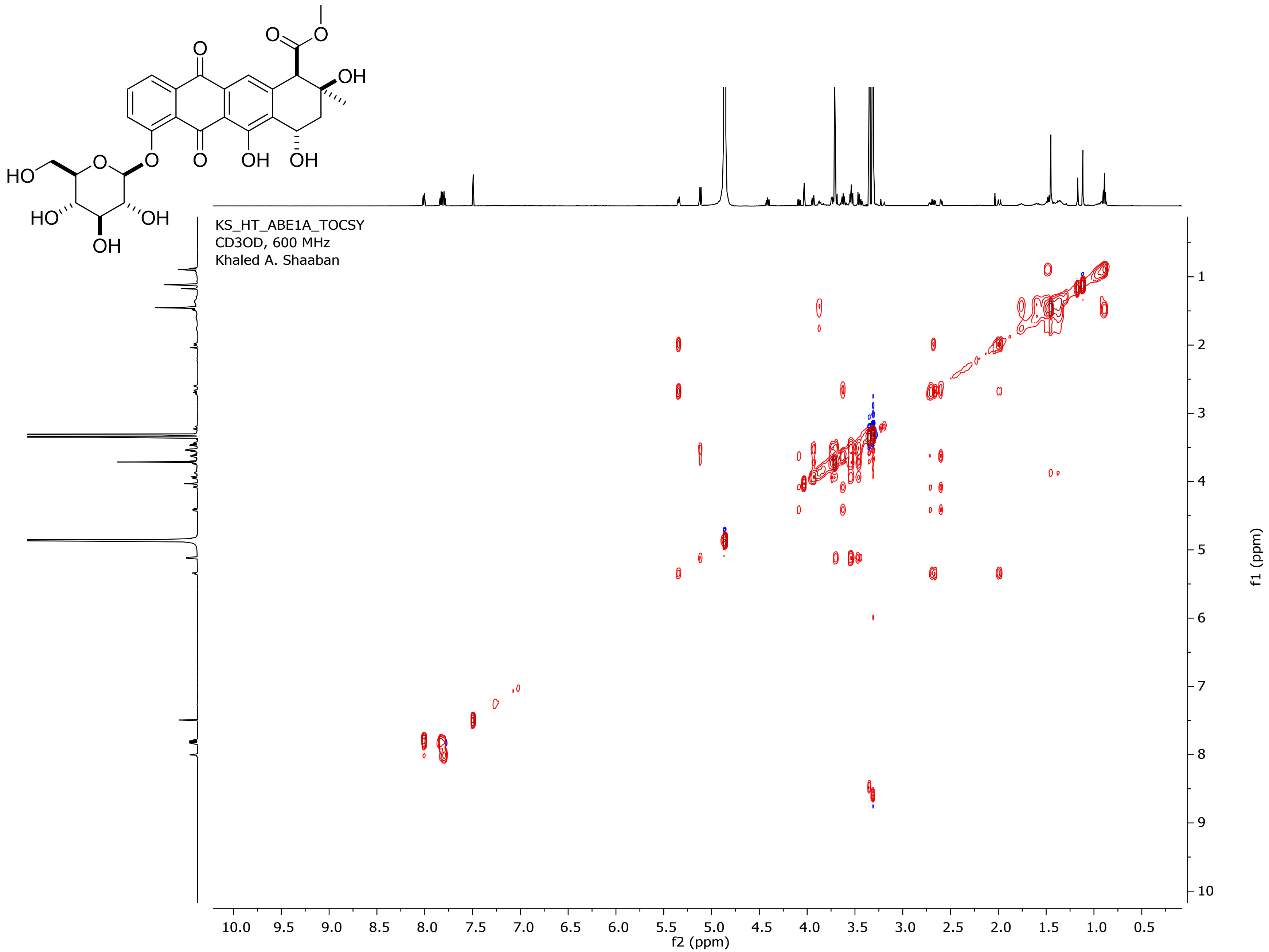

Figure S82. TOCSY spectrum (CD_3OD, 600 MHz) of **4-β-D-glucosyl-nogalamycinone** (**6**).

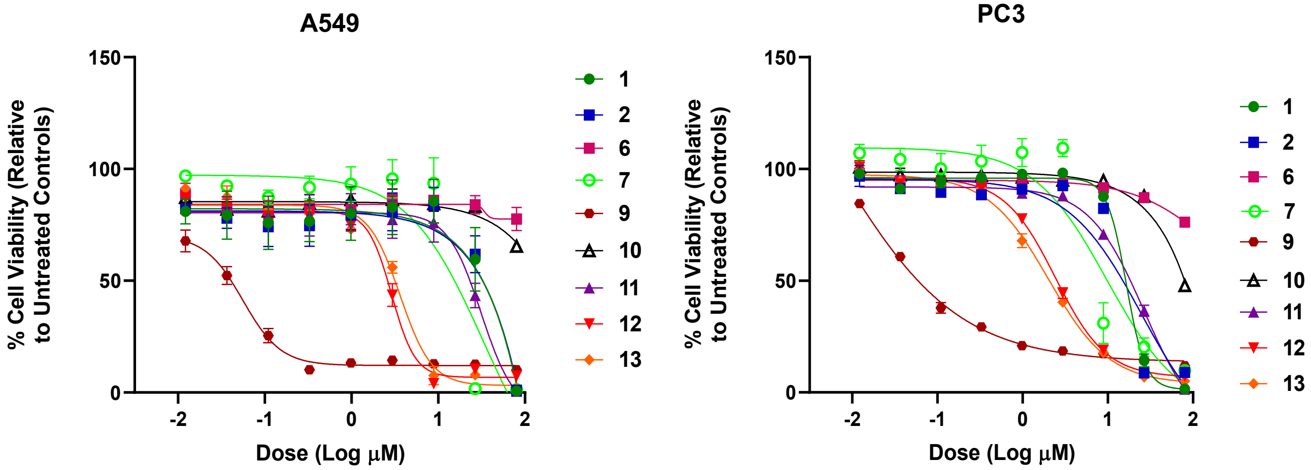

Figure S83: A) Dose-response of compounds 1, 2, 6, 7 and 9-13 against A549 (non-small cell lung) human cancer cell line (72 h). B) Dose-response of compounds 1, 2, 6, 7 and 9-13 against PC3 (prostate) human cancer cell line (72 h).

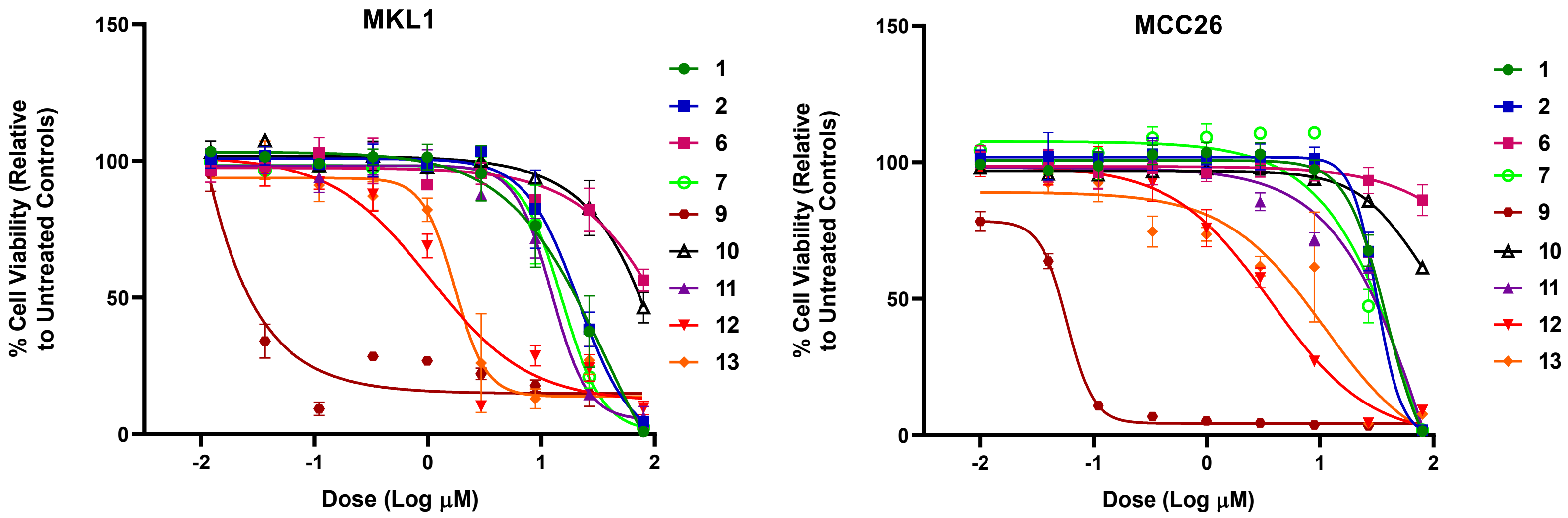

Figure S84: Dose-response of compounds 1, 2, 6, 7 and 9-13 against Merkel cells (MKL1 and MCC26), (72 h).

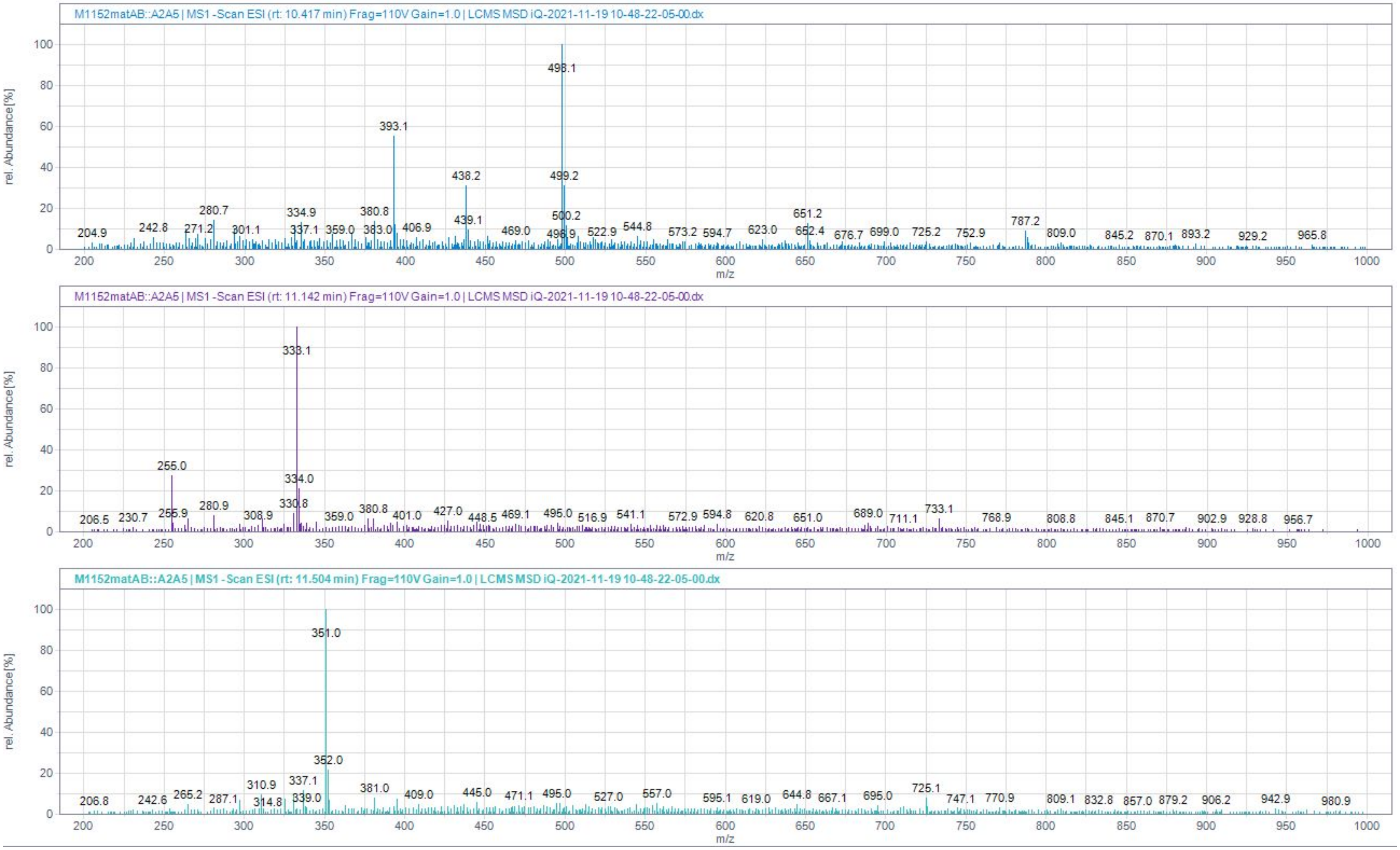

Figure S85: ESI-MS negative mode mass spectrum of aklanonic acid shunt products AA-1, AA-2, and AA-3 from *S. coelicolor* M1152Δ*matAB*::pSET-A2A5.

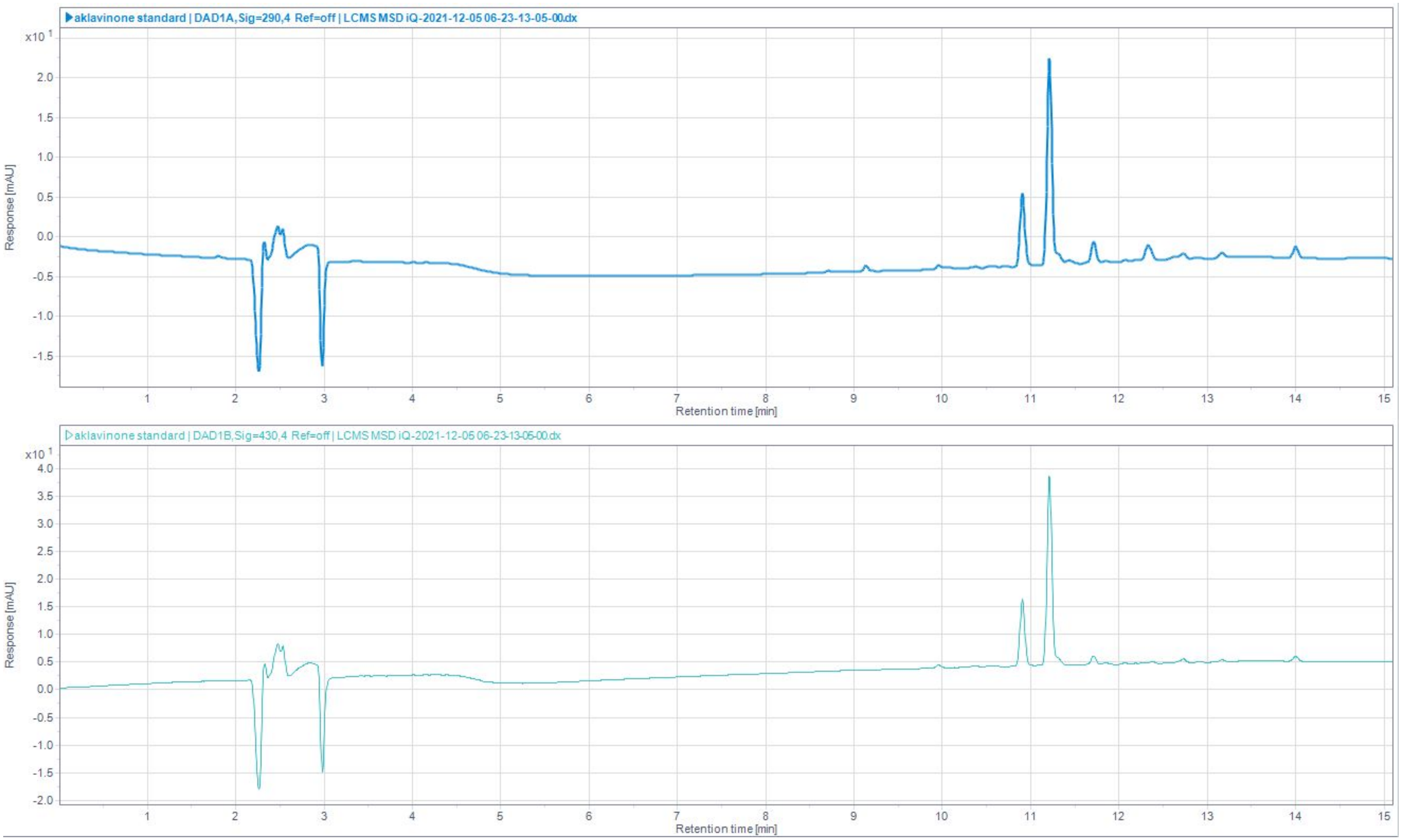

Figure S86: HPLC-UV/vis analysis of aklavinone standard.

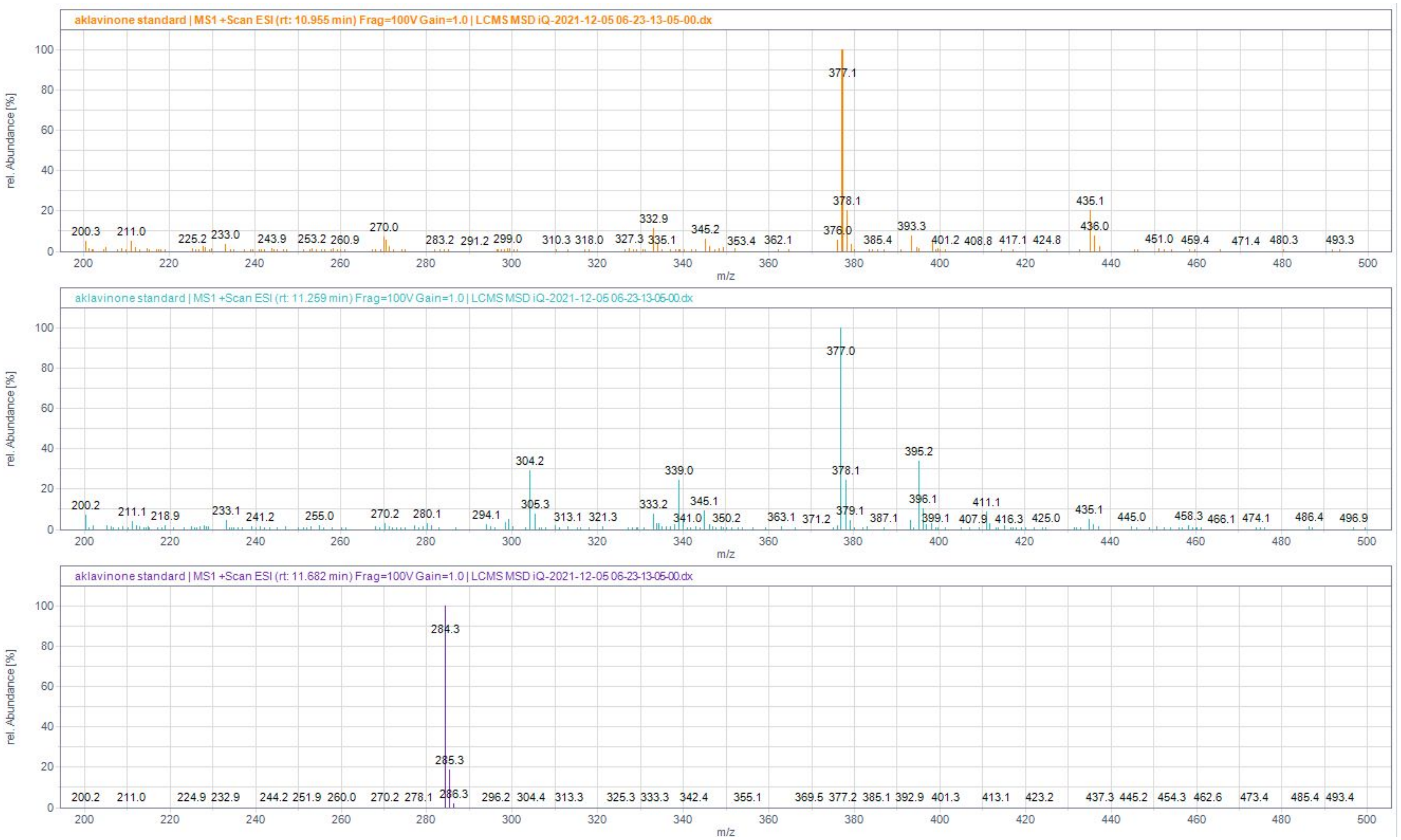

Figure S87. ESI-MS negative mode mass spectrum of aklavinone standard $[M - H]^-$ = 411 *m/z*.

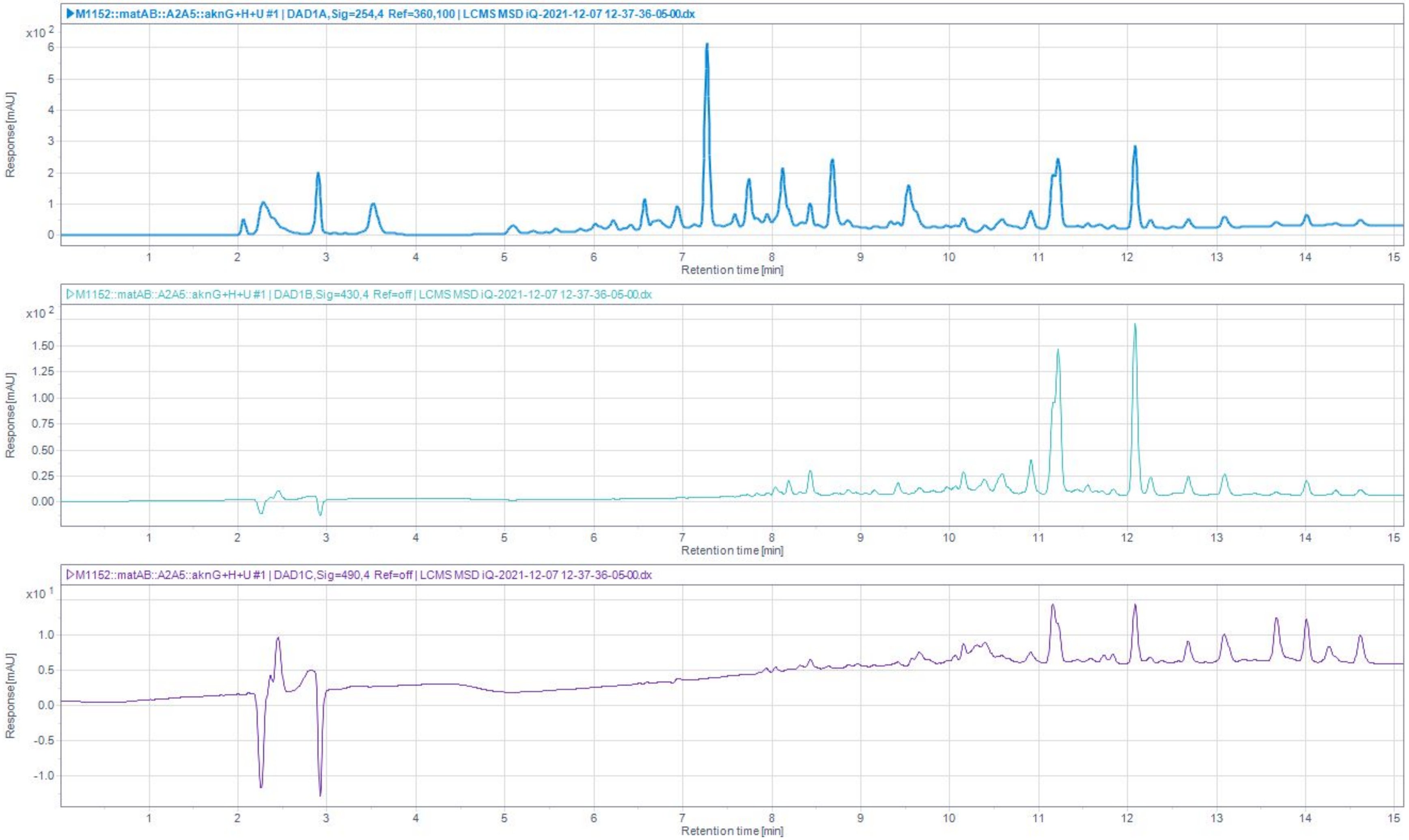

Figure S88: HPLC-UV/vis analysis of aklavinone produced from *S. coelicolor* M1152Δ*matAB*::pSET-A2A5::pTG-A6.

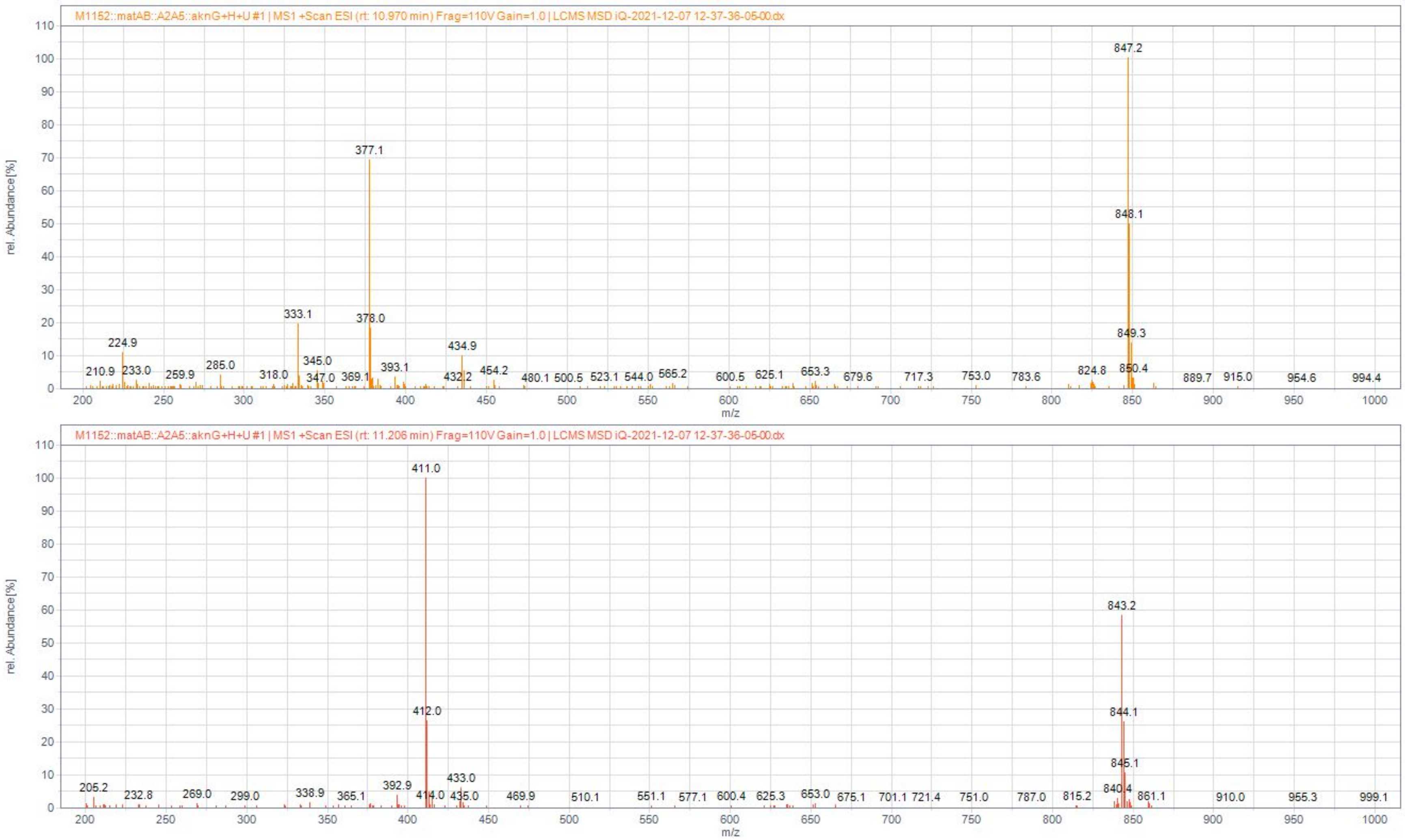

Figure S89. ESI-MS negative mode mass spectrum of aklavinone produced from *S. coelicolor* M1152Δ*matAB*::pSET-A2A5::pTG-A6. $[M - H]^-$ = 411 *m/z*.

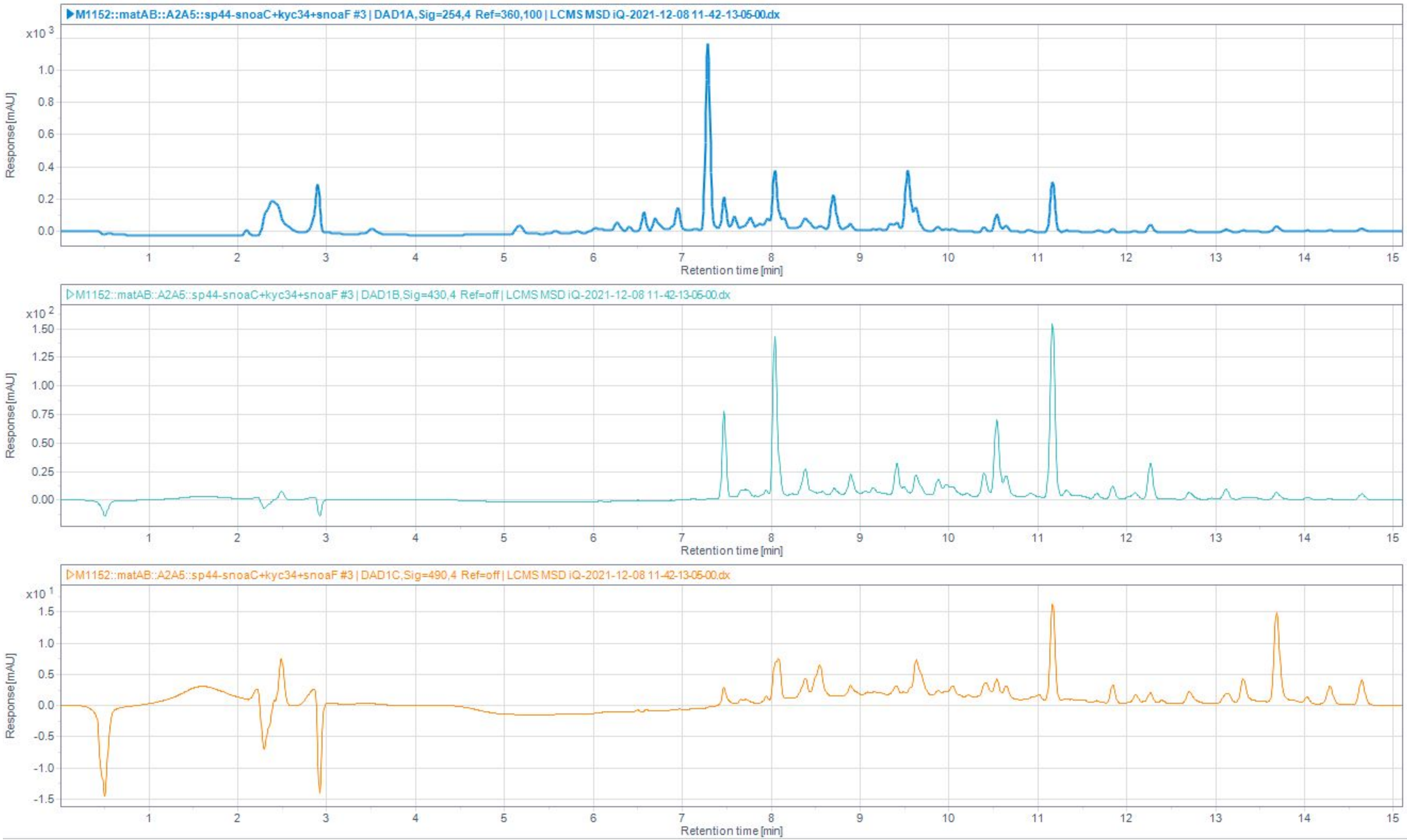

Figure S90. HPLC-UV/vis analysis of 9-*epi*-aklavinone produced from *S. coelicolor* M1152Δ*matAB*::pSET-A2A5::pTG-S7.

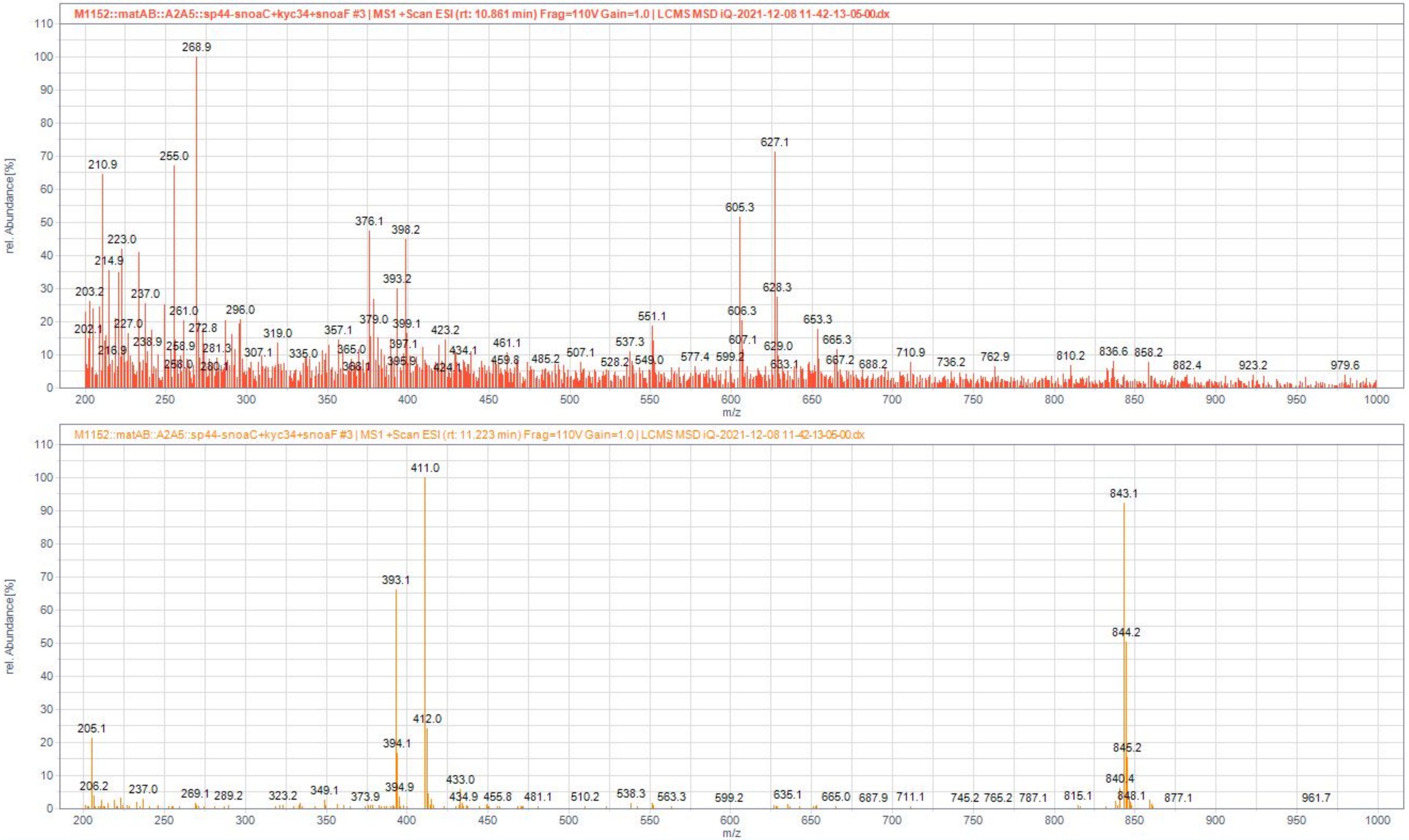

Figure S91. ESI-MS negative mode mass spectrum of 9-*epi*-aklavinone produced from S. coelicolor M1152ΔmatAB::pSET-A2A5::pTG-S6. $[M - H]^-$ = 411 *m/z*.

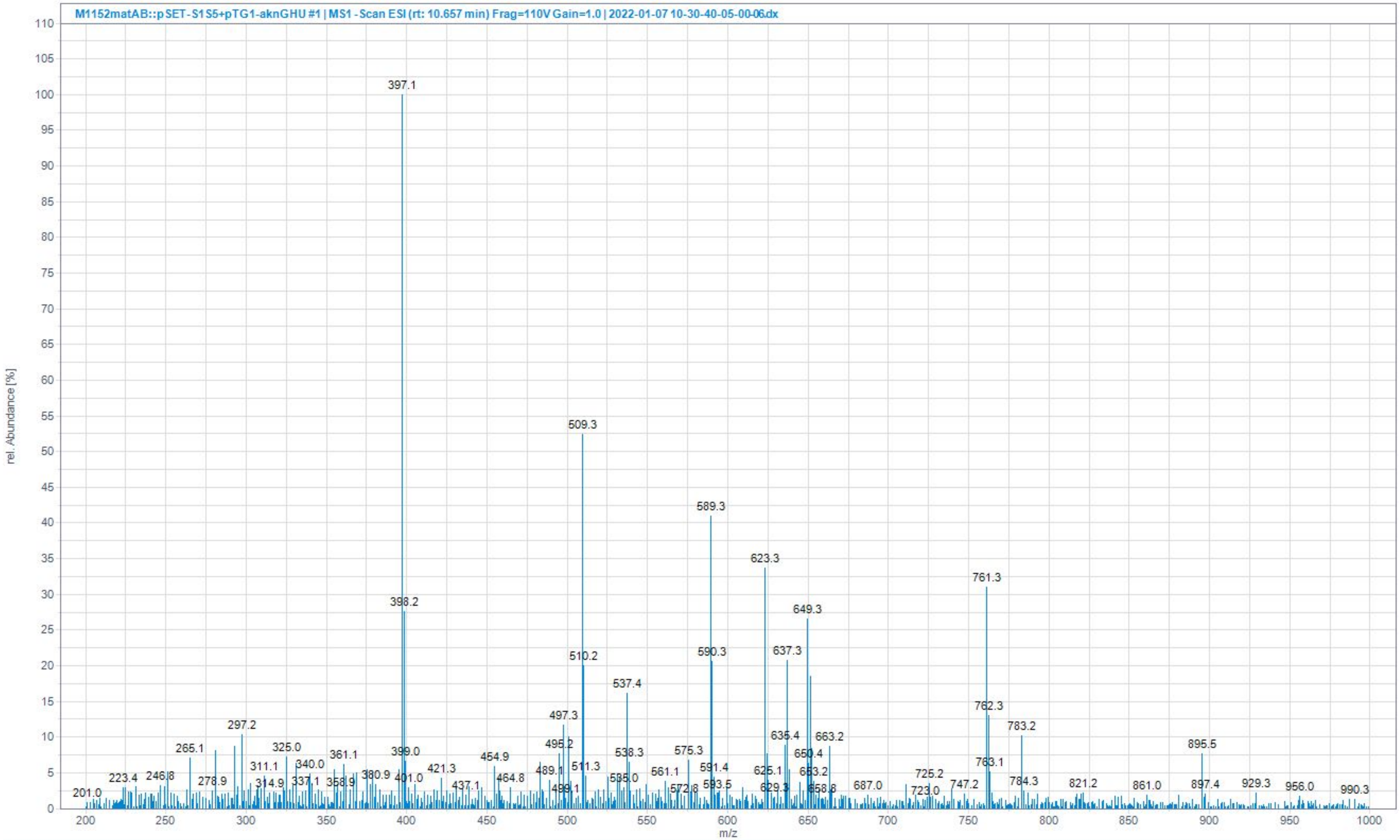

Figure S92. ESI-MS negative mode mass spectrum of auramycinone produced from S. coelicolor M1152ΔmatAB::pSET-S2S5::pTG-A6. $[M - H]^-$ = 397 *m/z.*

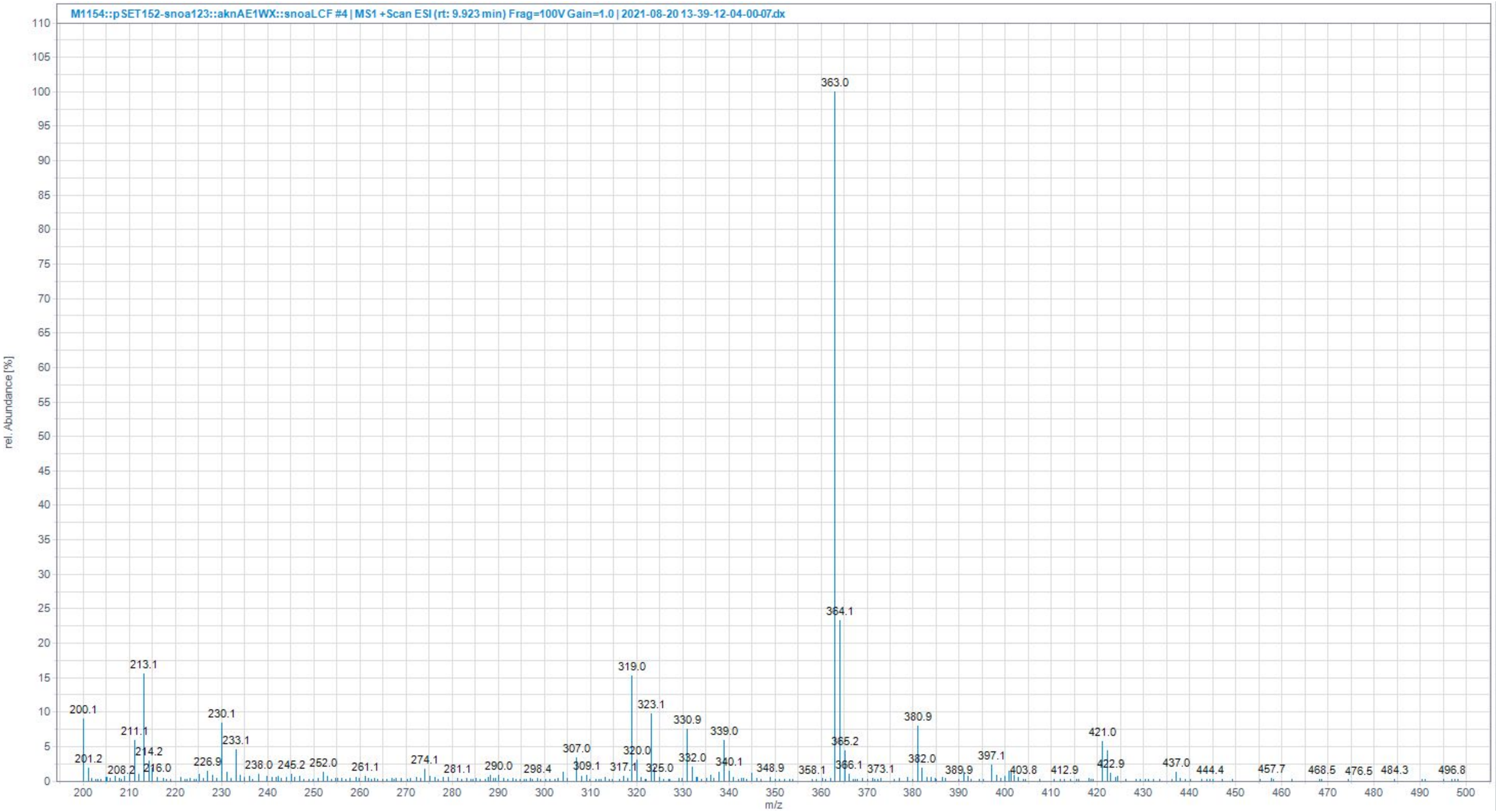

Figure S93 ESI-MS positive mode mass spectrum of nogalamycinone produced from S. coelicolor M1152ΔmatAB::pRW10000. $[M + Na]^+$ = 421 *m/z*.

Supplementary References

(1) MacNeil, D. J.; Gewain, K. M.; Ruby, C. L.; Dezeny, G.; Gibbons, P. H.; MacNeil, T. Gene 1992, 111 (1), 61–68.

(2) Ziermann, R.; Betlach, M. C. Biotechniques 1999, 26 (1), 106–110.

(3) McDaniel, R.; Ebert-Khosla, S.; Hopwood, D. A.; Khosla, C. Science 1993, 262 (5139), 1546–1550.

(4) Gomez-Escribano, J. P.; Bibb, M. J. Microb Biotechnol 2011, 4 (2), 207–215. h

(5) Bierman, M.; Logan, R.; O'Brien, K.; Seno, E. T.; Nagaraja Rao, R.; Schoner, B. E. Gene 1992, 116 (1), 43–49.

(6) Nguyen, J. T.; Riebschleger, K. K.; Brown, K. v.; Gorgijevska, N. M.; Nybo, S. E. Biotechnol J 2021, 2100371.

(7) Aubry, C.; Pernodet, J. L.; Lautru, S. Appl Environ Microbiol 2019, 85 (16).

(8) Otsuka, J, and Kunisawa T. Journal of theoretical biology 97.3 (1982): 415-436.

(9) Huff J, Czyz A, Landick R, Niederweis M. Gene. 2010 Nov 15;468(1-2):8-19.

(10) Chen YJ, Liu P, Nielsen AA, Brophy J. A. N., Clancy K, Peterson T, Voigt C. A. Nat Methods. 2013;10(7):659-664.

(11) Shaaban, K. A.; Wang, X.; Elshahawi, S. I.; Ponomareva, L. V.; Sunkara, M.; Copley, G. C.; Hower, J. C.; Morris, A. J.; Kharel, M. K.; Thorson, J. S. J. Nat. Prod. 2013, 76, 1619–1626.

(12) Wang, X.; Shaaban, K. A.; Elshahawi, S. I.; Ponomareva, L. V.; Sunkara, M.; Zhang, Y.; Copley, G. C.; Hower, J. C.; Morris, A. J.; Kharel, M. K.; Thorson, J. S. J. Nat. Prod. 2013, 76, 1441–1447.

(13) Shaaban, K. A.; Elshahawi, S. I.; Wang, X.; Horn, J.; Kharel, M. K.; Leggas, M.; Thorson, J. S. J. Nat. Prod. 2015, 78, 1723–1729.

(14) Savi, D. C.; Shaaban, K. A.; Gos, F.; Ponomareva, L. V.; Thorson, J. S.; Glienke, C.; Rohr, J. Sci. Rep. 2018, 8, 3122.

(15) van Dissel, D., Claessen, D., Roth, M. and van Wezel, G.P., 2015. A novel locus for mycelial aggregation forms a gateway to improved Streptomyces cell factories. Microbial Cell Factories, 14(1), pp.1-10.

(16) Wu, C., van der Heul, H.U., Melnik, A.V., Lübben, J., Dorrestein, P.C., Minnaard, A.J., Choi, Y.H. and van Wezel, G.P., 2019. Lugdunomycin, an Angucycline-Derived Molecule with Unprecedented Chemical Architecture. Angewandte Chemie, 131(9), pp.2835-2840.
